# Beyond the clinic: improving child health through evidence-based community development

**DOI:** 10.1186/1471-2431-13-172

**Published:** 2013-10-21

**Authors:** Kelli A Komro, Amy L Tobler, Alexis L Delisle, Ryan J O’Mara, Alexander C Wagenaar

**Affiliations:** 1 Department of Health Outcomes and Policy, Institute for Child Health Policy, University of Florida, 1329 SW 16th St, Rm 5285, PO Box 100177, Gainesville, FL 32610-01, USA

**Keywords:** Child, Adolescent, Health promotion, Policy, Evidence-based practice, Review

## Abstract

**Background:**

Promoting child wellbeing necessarily goes beyond the clinic as risks to child health and development are embedded in the social and physical environmental conditions in which children live. Pediatricians play a vital role in promoting the health of children in the communities they serve and can maximize their impact by advocating for and supporting efficacious, evidence-based strategies in their communities.

**Methods:**

To provide a succinct guide for community pediatric efforts to advance the wellbeing of all children and particularly disadvantaged children in a community, we conducted a theory-driven and structured narrative review to synthesize published systematic and meta-analytic reviews of policy-relevant, local-level strategies addressing potent and malleable influences on child health and development. An exhaustive list of policy-relevant, local-level strategies for improving child health was used to conduct a comprehensive search of recent (1990–2012), English language peer-reviewed published meta-analyses and systematic reviews in the 10 core databases of scientific literature. Our review of the literature encompassed six key conceptual domains of intervention foci, including distal influences of child health (i.e., income and resources, social cohesion, and physical environment) and proximal influences (i.e., family, school and peer). We examined intervention effects on four key domains of child health and development: cognitive development, social and emotional competence, psychological and behavioral wellbeing, and physical health.

**Results:**

Published reviews were identified for 98 distinct policy-relevant community interventions, evaluated across 288 outcomes. We classified 46 strategies as meeting scientific criteria for efficacy by having consistent, positive outcomes from high-quality trials (e.g., tenant-based rental assistance, neighborhood watch programs, urban design and land use policies, access to quality childcare services, class size reductions, after-school programs that promote personal/social skills). Another 21 strategies were classified as having consistent evidence of positive outcomes from high-quality observational studies only, while 28 strategies had insufficient evidence available to assess their effectiveness based on published reviews. We did not limit the review to studies conducted in the United States, but the vast majority of them were U.S.-based, and the results therefore are most applicable to the U.S. context.

**Conclusions:**

Based on our synthesis of published literature on community development strategies, we provide an illustration combining a comprehensive set of evidence-based strategies to promote child health and development across a wide-range of child health outcomes.

## Background

A mounting body of research has documented the social gradient in child health [1]. Children of low socioeconomic status (SES) [2,3], racial and ethnic minorities [4,5], and residents of disadvantaged neighborhoods [6,7] have substantially increased risk for deleterious health outcomes. Poverty raises risk for infant mortality, low birth weight, poor diet, limited physical activity, teenage childbearing, depression, obesity, unintentional injuries, homicide, suicide, coronary heart disease, diabetes, and overall ill health [3,8]. In the United States, the poverty rate is three times higher for African American and Hispanic/Latino children than Caucasian (non-Hispanic) children (39% of African American children and 35% of Hispanic children are below the federal poverty line, compared with 12% of Caucasian children) [9]. In addition to increased risk associated with poverty, ethnic and minority populations experience even greater health detriments, apparently due to more concentrated disadvantage and experiences related to racial bias [3]. Furthermore, large geographic variations in health outcomes—independent of individual-level attributes—underscore that “place” is an important contextual determinant of health [6].

Given the multiplicity of health risks associated with social disadvantage, strategies to promote child health and wellbeing necessarily go beyond the clinic and involve community-wide policy strategies that change the broader environments in which children live. There is a growing understanding that health disparity populations (i.e., low SES groups, racial/ethnic minorities, and residents of distressed neighborhoods) are overlapping populations suffering avoidable health inequities resulting from unequal distribution of health-damaging and health-protecting exposures in daily life [10,11]. Policies, programs and practices of the public sector, private sector and civil society largely shape the economic, social and physical environmental conditions in which individuals live, and these structural determinants and conditions of daily life constitute the social determinants of health which account for a large part of health disparities [12].

Child-focused community development initiatives offer great potential to prevent the numerous, potentially life-long and generationally transmitted, health inequities related to SES, race/ethnicity, and neighborhood. Recent efforts in the United States include the Promise Neighborhoods program [13] modeled after the Harlem Children’s Zone [14], as well as other community development initiatives seeking to build health-enhancing communities through comprehensive strategies such as improving access to quality education and providing family supports. Such “place-based” initiatives aim to improve the developmental trajectories of children residing in areas of concentrated disadvantage by providing a continuum of supports from “cradle to career.” These initiatives are promising because evidence shows that prevention is most effective when it occurs early in life, addresses root causes at multiple ecological levels, and is sustained over time [8,15].

Despite sizeable investments in child-focused community development initiatives and the tremendous potential of such efforts to improve social and health inequities, extant enacted policies and programs often do not reflect effective practices based on scientific studies [8,16,17]. In recent decades, prevention science has made significant progress in determining effective community-level strategies (such as quality preschool education, parent education and support, and improvements to the built environment) that influence potent risk and protective factors affecting child development and health outcomes from infancy through adolescence [8]. Despite progress in identifying effective community practices and policies, significant challenges remain on how to coherently combine and adapt evidence-based strategies to unique local settings, foster community support for change, bring these comprehensive efforts to scale within communities, and sustain preventive effects over time to permanently improve child health outcomes and reduce health disparities [15].

**Figure 1:**
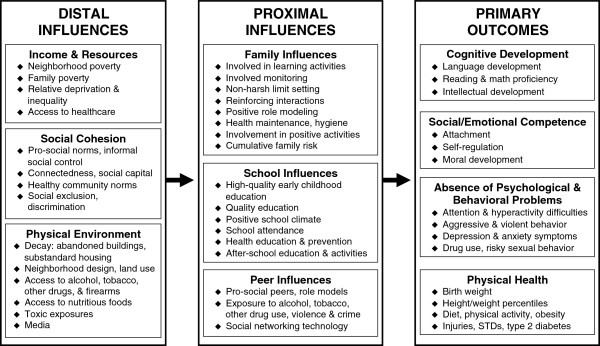
**A framework for creating nurturing environments.** Note. This figure is adapted with kind permission from Springer Science + Business Media: Clinical Child and Family Psychology Review, Creating Nurturing Environments: A Science-Based Framework for Promoting Child Health and Development Within High-Poverty Neighborhoods, 14(2), 2011, 114, Komro, K.A., Flay, B.R., Biglan, A. & PNRC, Figure 1.

The Promise Neighborhoods Research Consortium (PNRC), a network of prevention scientists in the U.S., created a science-based framework to guide comprehensive, community-level efforts promoting development and health among socially disadvantaged children living in distressed neighborhoods [15]. This framework (Figure 1) identifies distal influences (i.e., income and resources, social cohesion, and physical environment) and proximal influences (i.e., family, school and peer) that are critical to children’s cognitive development, social and emotional competence, psychological and behavioral wellbeing, and physical health. Guided by this framework, and in collaboration with PNRC, we sought to identify evidence-based, policy-relevant strategies that local governments and institutions could enact to facilitate lasting, population-level improvements in child outcomes within distressed neighborhoods. As pediatricians play a vital and long-standing role in promoting the health and well-being of all children in the communities they serve [18], pediatricians can maximize their impact by advocating for and supporting efficacious, evidence-based strategies in their communities.

In this paper, we present the results of a synthesis of published systematic reviews or meta-analyses of six intervention domains for protecting and promoting child health. We did not limit the review to studies conducted in the United States, but the vast majority of them were U.S.-based, and the results therefore are most applicable to U.S. context. Based on our synthesis of the published literature, we provide an assessment of the current state-of-the-science regarding community development strategies that influence child health. Finally, we present an illustration of a comprehensive set of evidence-based strategies for child health as guidance for pediatricians and other child health advocates advancing local community development efforts. Scientific evidence on health promotion and risk prevention strategies is diverse, complicated, inconsistent in quantity and quality, and often inaccessible to policymakers, health care providers and other community stakeholders [19]. Our goal is to provide practitioners with a useable summary of a wide-ranging set of scientific literature relevant to community development strategies for child health.

## Methods

We conducted a theory-driven and structured narrative review of systematic reviews of a comprehensive set of community development strategies for child health. We limited the scope of our narrative review to those strategies that have been assessed in peer-reviewed, published meta-analyses or systematic reviews for three reasons. First, the scope of the conceptual framework guiding our work precludes exhaustive review of individual studies or newly emerging reports on prevention strategies that have yet to warrant systematic review. Second, systematic reviews represent the pinnacle of the “levels of evidence” hierarchy and provide the best practical method for identifying effective interventions [20]. Third, systematic reviews play an integral role in decision-making about community prevention strategies because they help synthesize bodies of potentially-conflicting research results, appropriately weight smaller versus larger studies, and provide more accurate estimates of effect size than individual studies or non-quantitative reviews [21].

### Literature search

Guided by our conceptual framework (Figure 1), scholarly literature, and the collective expertise of a sizable network of prevention scientists involved with the Promise Neighborhoods Research Consortium (PNRC), we developed an exhaustive list of local-level, policy-relevant strategies that community leaders could implement to improve child health and development. We then conducted a systematic, comprehensive search for recent (1990–2012), peer-reviewed published meta-analyses and systematic reviews in several databases: The Cochrane Database of Systematic Reviews; The Campbell Library; Wolters Kluwer; Ovid; Evidence Based Medicine Reviews; JSTOR; MEDLINE; PubMed; Wiley; and Google™ Scholar Beta. Search terms for each database included any combination of the relevant terms comprising a particular policy subject or title, followed by the terms: review, systematic, systematic review, meta-analysis, or meta-analytic. For example, search terms for reviews of alcoholic beverage excise taxes were: [(tax OR taxes OR taxation OR cost* OR price OR prices) AND (alcohol* OR drinking OR liquor OR drunk* OR beer OR wine OR spirits OR malt beverage*) AND (review* OR systematic OR systematic review OR meta-analysis OR meta-analytic)]. We identified any record with the search terms in the title, keywords, subject heading, or abstract field. We further identified relevant reviews in the reference lists of articles from the original database search. Identified papers were sorted and examined for relevance and content. We excluded (1) publications of single studies, (2) legal reviews, commentaries, or narrative literature reviews with no systematic review or meta-analytic methods, and (3) publications not written in English from our summary.

### Classification criteria

#### Outcomes

We include summaries of effects on both primary and intermediate child health outcomes. We define primary outcomes to include any measures of cognitive development, social/emotional competence, absence of psychological and behavioral problems, and physical health among children or adolescence. We define intermediate outcomes to include measures of any of the proximal and distal influences on primary child outcomes (Figure 1). For example, a decrease in youth alcohol use is a primary outcome, whereas a reduction in alcohol sales to underage youth is an intermediate outcome.

#### Level of evidence

Policy-relevant strategies were classified into six categories that influence child health outcomes consistent with our framework (Figure 1). Based on published systematic reviews and meta-analyses, we evaluated the *level of evidence* on a scale of 1 to 3 for each strategy following the standards of evidence for efficacious interventions laid out by the Society for Prevention Research [22]. Level 1 classification represents strategies that meet criteria for an efficacious intervention by having consistent, positive outcomes from at least two high-quality experimental or quasi-experimental trials using a comparison group or interrupted time-series design. Level 2 classification represents strategies with consistent evidence of positive outcomes from high-quality observational studies only. Level 3 represents strategies with insufficient evidence available to determine effectiveness. Generally, each systematic review or meta-analysis reported sufficient information to determine the number of high-quality experimental or quasi-experimental trials. In cases where this information was not presented, individual studies were examined to determine the appropriate level of evidence.

#### Magnitude of effect

Since quantitative outcomes were reported in a variety of ways in the meta-analytic studies we reviewed, we standardized the effect estimation into an ordinal *magnitude of effect*. If a study reported an odds ratio ≤ 0.5 or ≥ 1.5, Cohen’s *d* ≥ 0.7, or percent change ≥ 70%, the effects were classified as “large.” We classified effect estimates as “medium” for outcomes with an odds ratio between 1.20-1.49 or 0.51-0.79, Cohen’s *d* = 0.3-0.69, or percent change between 30-69%. “Small” effect estimates were assigned to outcomes with an odds ratio between 0.80-1.19, Cohen’s *d* < 0.3, or percent change < 30%. If a study did not conduct a meta-analysis or report quantitative estimates, and the authors did not make any indication of the magnitude of effects observed, we indicated that effect sizes were not reported, “NR.”

## Results

We identified published systematic reviews for 98 policy-relevant community strategies with 288 studied outcomes. Based on the standards of evidence for efficacious interventions laid out by the Society for Prevention Research [22], 46 strategies meet scientific criteria for efficacy by having consistent, positive outcomes from at least two high-quality experimental or quasi-experimental trials using a comparison group or interrupted time-series design (Additional file 1). An additional 21 community intervention strategies had consistent evidence of positive outcomes from high-quality observational studies only (Additional file 1), and 28 strategies had insufficient evidence available to assess effectiveness based on published systematic reviews (Additional file 1). The Additional files 1 and 2 stratify policy-relevant community strategies by quality of evidence (i.e., Level 1–3) and intervention domain (i.e., income and resources, social cohesion, physical environment, family influences, school influences, and peer influences), and display each strategy’s studied outcomes, including direction and magnitude of effect.

Three strategies were found with sufficient evidence that they were not effective or had deleterious effects (excluded from Additional files): increased college tuition resulted in lower attendance among low income and ethnic minority populations; [23] zero tolerance policy in schools showed no improvements in school safety; [24] and transfer of juvenile offenders to adult criminal court had harmful consequences [25].

**Figure 2:**
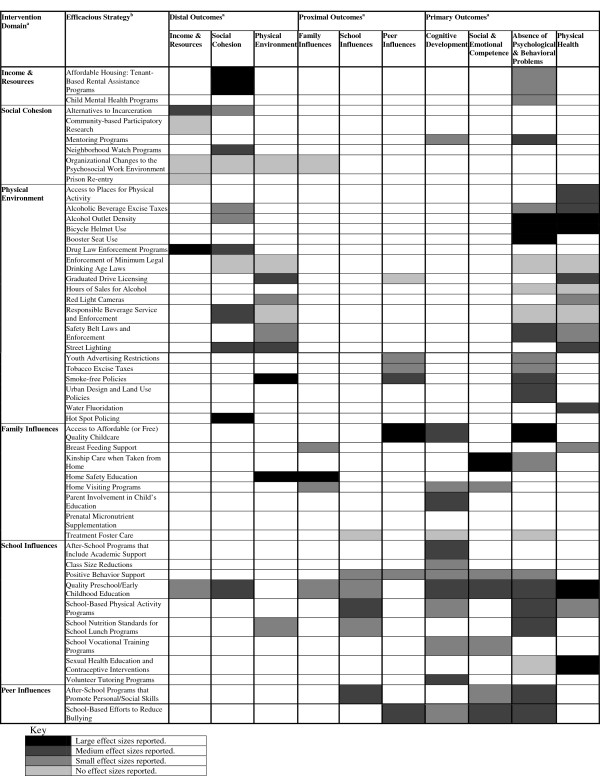
Policy-relevant community strategies for improving child health and development. Note. ^a^Intervention domains and outcome categories are defined by science-based framework for promoting child health and development within distressed neighborhoods (Figure 1). ^b^Strategy meets criteria for efficacy based on a minimum of 2 high-quality trials (Level 1).

Figure 2 summarizes the 46 most efficacious (Level 1) strategies and their studied outcomes. Ten of these efficacious strategies showed medium to large effect sizes in improving child physical health: access to places for physical activity, alcoholic beverage excise taxes, alcohol outlet density, bicycle helmet use, graduated driver licensing, prenatal micronutrient supplementation, quality preschool/early childhood education, sexual health education and contraceptive interventions, street lighting, and water fluoridation. An additional 14 strategies showed medium to large effect sizes in improving primary child outcomes other than physical health (i.e., cognitive development, social/emotional competence, and absence of psychological and behavioral problems): access to affordable (or free) child health care, after school programs that include academic support, after-school programs that promote personal/social skills, booster seat use, kinship care when taken from home, mentoring programs, parent involvement in child’s education, safety belt laws and enforcement, school nutrition standards for school lunch programs, school-based efforts to reduce bullying, school-based physical activity programs, smoke-free policies, urban design and land use, and volunteer tutoring programs. Two promising strategies (i.e., after school programs that promote personal/social skills, quality preschool/early childhood education) showed small to large effect sizes across a wide range of distal, proximal, and primary child health outcomes.

### Income and resources

In the Income and Resources domain, published systematic reviews identify 2 policy-relevant strategies (i.e., tenant-based rental assistance programs, child mental health programs) that meet criteria for an efficacious intervention (i.e., Level 1) on 5 outcomes. Most of the effects are small on primary outcomes specific to children or adolescents (e.g., experiences of victimization within the neighborhood, diagnoses of conduct disorder and depression). Published reviews show 5 strategies (e.g., active labor market policies, living wage ordinances) with consistent evidence from observational studies only (i.e., Level 2) on 14 outcomes, most of which are intermediate and not specific to children or adolescents (e.g., job placement, urban poverty). Most of the effects are small, but medium-sized effects were found for policies related to need-based grants for higher education and child health care access. Finally, 7 strategies (e.g., conditional cash transfer, welfare-to-work) with 17 studied outcomes (e.g., utilization of health services, employment and earnings of welfare recipients) are found in published systematic reviews to have insufficient evidence to determine effects (i.e., Level 3).

### Social cohesion

In the Social Cohesion domain, published systematic reviews identify 6 strategies (e.g., neighborhood watch programs, restorative justice programs) that meet the criteria for an efficacious intervention (i.e., Level 1) on 23 outcomes, most of which are intermediate and not specific to children or adolescents (e.g., crime, recidivism). Published reviews show 3 strategies (e.g., community-based arts programs, employee share ownership and profit-sharing) with consistent evidence from observational studies (i.e., Level 2) on 12 intermediate outcomes (e.g., community unity, worker productivity), none specific to children or adolescents. Sizes of these effects are in the small to medium range. Four strategies (e.g., collective or community kitchens, enterprise zones) with 9 studied outcomes (e.g., social cohesion, increases employment and business in distressed areas) are found in published reviews to have insufficient evidence to determine effects (i.e., Level 3).

### Physical environment

In the Physical Environment domain, published reviews identify 18 strategies (e.g., smoke-free policies, urban design and land use policies) that meet the criteria for an efficacious intervention on 58 outcomes, most measuring primary outcomes not specific to youth (e.g., environmental tobacco smoke, physical activity). The largest effects were found for policies mandating bicycle helmet and car booster seat use, and reducing the density of retail beverage alcohol outlets. Reviews show 5 strategies (e.g., point of purchase nutrition strategies, pool fencing) with consistent evidence from observational studies on 15 outcomes (e.g., health foods/snacks sales, drowning risk). Finally, reviews identify 11 strategies (e.g., condom availability in schools, firearm policies) with insufficient evidence on 17 outcomes (e.g., safe sex practices in teens, violence).

### Family influences

In the Family domain, published reviews identify 8 strategies (e.g., prenatal micronutrient supplementation, parent involvement in child’s education) that meet criteria for an efficacious intervention on 30 outcomes, most specific to children or adolescents (e.g., low birth weight infants, academic performance). The largest effects were seen for access to affordable quality childcare services, child kinship care when taken from home, and home safety education. Reviews identify one strategy (early childhood health promotion) with consistent evidence from observational studies on 4 outcomes specific to youth (e.g., parents’ and children’s safety behaviors), and one strategy (mental health services for parents) with insufficient evidence on 2 outcomes (e.g., child social adjustment).

### School influences

In the School Influences domain, published reviews identify 9 strategies (e.g., class size reductions, school nutrition standards for school lunch programs) that meet efficacious intervention criteria on 43 outcomes, mostly specific to children and adolescents (e.g., academic performance, saturated fat intake). The largest estimated effects are for quality preschool/early childhood education, as well as sexual health education and contraceptive interventions. Reviews identify 7 strategies with consistent evidence from observational studies (e.g., school-based health centers, school funding) on 13 youth-specific outcomes (e.g., health services accessibility, academic achievement). Reviews identify 6 strategies (e.g., early college programs, vaccination for daycare) on 10 studied outcomes (e.g., college enrollment and success, illness in school-children) with insufficient evidence to estimate effects.

### Peer influences

Strategies related to Peer Influences overlap with other domains, such as social cohesion and school influences. Nevertheless, 2 strategies are primarily peer influence interventions: after-school programs that promote personal/social skills, and school-based efforts to reduce bullying. Both strategies meet criteria for efficacious interventions with mostly medium to large effects on 13 youth-specific outcomes (e.g., positive psychosocial environment in the classroom, victimization).

## Discussion

A theory- and evidence-based conceptual framework for comprehensive, localized efforts shows how to best promote child health and development within distressed high-poverty neighborhoods (Figure 1) [15]. Guided by a theory-based understanding of the main domains of influence on child health (i.e., income and resources, social cohesion, physical environment, family, school, peer), we conducted a comprehensive search for systematic or meta-analytic reviews of local-level policies or policy-relevant strategies that may amend those influences. To facilitate dissemination of efficacious, evidence-based community strategies to pediatricians and other child health advocates, we classified strategies by level of evidence and summarized the results in Figure 2. To further facilitate the dissemination and translation of evidence-based strategies, we packaged this evidence in a manner useful for pediatricians, local-level policymakers and other stakeholders [26]. We developed policy briefs (available in Additional file 2) and at http://promiseneighborhoods.org) that provide: (1) rationale for the policy, (2) outline of targeted outcomes, (3) summary and appraisal of the available evidence, and (4) real examples of the policy in action in community settings.

Published systematic reviews address 98 policy-relevant strategies with 288 studied outcomes ranging from primary indicators of child health to measures of important physical and social contextual influences on child health. Less than half (46 of 98) intervention strategies meet scientific criteria for efficacy, as defined by the Society of Prevention Research [22] by having consistent, positive outcomes from at least two high-quality experimental or quasi-experimental trials using a comparison group or interrupted time-series design. Another 21 strategies have consistent evidence of beneficial outcomes from high-quality observational studies.

Our primary objective was to organize a very large and diverse literature into a conceptually logical framework that reflects the best of current science, and succinctly summarize large bodies of scientific literature that makes clear to end-users the strength of evidence and size of estimated effects across the whole range of community changes thought to improve child health and wellbeing. Additionally, for each intervention, we created policy briefs to provide pediatricians, local-level policymakers, and other stakeholders with accessible science-based summaries to help identify policies that may effectively address child health needs in their community and facilitate community action around those issues. The Promise Neighborhoods Research Consortium website (http://promiseneighborhoods.org) displays all policy briefs in a searchable format organized by domain of influence on child health and development. The Consortium is also working actively to disseminate this information to the recent Promise Neighborhoods grantees and others planning community development initiatives.

**Figure 3:**
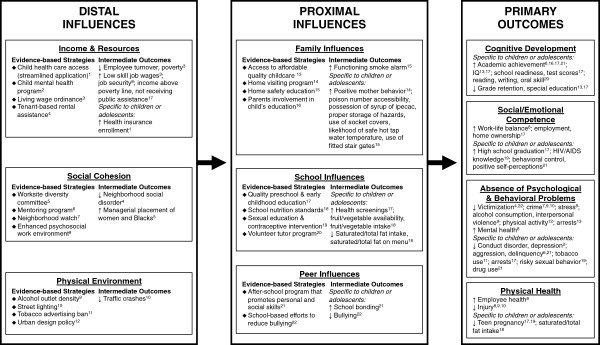
**An illustration of combining efficacious community strategies to improve child health and development.** Note. Superscript numbers link evidence-based strategies with corresponding studied outcomes. For example, quality preschool & early childhood education (superscript 17 under School Influences) has been shown to improve multiple primary outcomes in children (i.e., cognitive development, social/emotional competence, absence of psychological & behavioral problems) as well as affect distal and proximal outcomes (i.e., income & resources, school influences) that may positively impact child health and development.

In addition to helping select individual efficacious policies to improve child outcomes, another important application of our framework and policy briefs is to facilitate combining multiple strategies across domains of influence. This comprehensive approach contrasts with the narrow and isolated prevention efforts often implemented by local leaders and institutions. Ameliorating the many, and diverse, deleterious consequence of children living in high-poverty neighborhoods requires the mutually reinforcing benefits of multiple effective policy strategies across what have traditionally been viewed as separate areas of action. To illustrate a comprehensive approach to supporting child health and development (Figure 3), we selected 2–4 strategies per intervention domain based on the quality of the evidence (Level 1 or 2), magnitude of effects, and breadth of outcomes. This illustrative combination of evidence-based policy strategies is intended to improve income and resources for disadvantaged families; develop social cohesion within work, school and neighborhood environments; alter the physical environment to protect against risk and to promote physical activity and safety; encourage nurturing and safe home environments; provide high-quality education and educational environments; and support positive, health-promoting peer influences. Combining effective strategies across key intervention domains that influence child health produces multiple, positive reinforcing beneficial effects on intermediate and primary child health outcomes.

Our review of evidence-based policy strategies highlights several priority areas for further research. First, prevention scientists should conduct rigorous experimental or quasi-experimental studies of the strategies categorized level 2, listed in Additional file 1, because currently only observational studies have found positive effects of these strategies. Second, additional strategies need to be developed and rigorously evaluated in the income and resource domain. This domain is particularly challenging for neighborhood-based initiatives to change, given the complex socio-political influences on concentrated poverty. Related to this, prevention scientists should increasingly investigate the child health effects of these distal influences, as rigorous evaluations of social and economic policies’ effects on health outcomes is lacking despite the tremendous potential of these policies to influence population health [27,28]. Third, prevention scientists could work with pediatricians and other local child health advocates to better examine and understand the processes by which child health polices become disseminated and implemented in real community settings in order to evaluate the rollout of policy strategies under natural conditions [29].

## Conclusions

Our synthesis of published systematic reviews of local-level strategies to improve child outcomes yielded the creation of 95 policy briefs to guide community prevention efforts and future research. Summarizing the scientific evidence across such an expansive collection of prevention strategies has its limitations. We did not conduct a review of the primary scientific evidence; rather, we relied on published systematic reviews given the breadth of the extant literature. Therefore, this review potentially overlooks new, innovative and highly effective approaches to child health and wellbeing. Also, there was variation in the methodological rigor of the search and summarization methods employed in the included reviews. We did not conduct a formal assessment (e.g., AMSTAR) of the quality of the reviews. However, narrative literature reviews with no systematic review or meta-analytic methods were excluded from our summary. We critically evaluated the studies included in each published review, classified the level of evidence, and provided a summary of findings. No individual study can definitively answer the question of a policy’s effectiveness across populations and time. Therefore, the accumulation of evidence and meta-analysis of outcomes across studies provides a more accurate assessment of effectiveness than a single study. Finally, the vast majority of studies were U.S.-based, and the results therefore are most applicable to U.S. context. Therefore, one should generalize to other countries with care, especially when considering countries that are quite dissimilar to the U.S.

We present a current state of the science in order to (1) inform pediatricians and other child health advocates about policy-relevant community strategies with the most evidence demonstrating efficacy at improving primary child health outcomes, proximal influences of child health and development (i.e., family, school, peer), and distal influences (i.e., income and resources, social cohesion, physical environment); as well as to (2) inform scientists of community interventions that require more empirical attention to determine their efficacy at improving child health and reducing health disparities. It is our hope that continued evaluation and systematic review of child health policies and policy-relevant strategies, in conjunction with translation of the most evidence-based strategies into effective polices and practices, will lead to optimal child outcomes and minimal health disparities.

## Competing interests

The authors declare that they have no competing interests.

## Authors’ contributions

KK, AT and AW participated in the conceptualization of the study concept and design. AD and AT participated in the acquisition of data. AT, RO, AD, KK and AW participated in the analysis and interpretation of data. AT, RO, AD and KK drafted the manuscript. KK, AW, RO and AT participated in critical revision of the manuscript for important intellectual content. KK and AT supervised the study. All authors read and approved the final manuscript.

## Authors’ information

Kelli A. Komro, MPH, PhD is Professor of Health Outcomes and Policy in the College of Medicine, Associate Director of the Institute for Child Health Policy, and University of Florida Research Foundation Professor.

Amy L. Tobler, MPH, PhD is Assistant Professor of Health Outcomes and Policy in the College of Medicine and Institute for Child Health Policy at the University of Florida.

Alexander C. Wagenaar, PhD, is Professor of Health Outcomes and Policy in the College of Medicine and Institute for Child Health Policy at the University of Florida and Associate Director of Public Health Law Research, a national program of the Robert Wood Johnson Foundation, housed at the Temple University Beasley School of Law.

Alexis L. Delisle, MS, was an Institute for Child Health Policy predoctoral fellow at the time of this work.

Ryan J. O’Mara, MS, is an MD/PhD student at the University of Florida and was a Research Assistant in the Institute for Child Health Policy at the time of this work.

## Pre-publication history

The pre-publication history for this paper can be accessed here:

http://www.biomedcentral.com/1471-2431/13/172/prepub

## Supplementary Material

Additional file 1 Policy-relevant community strategies by level of evidence. Click here for file

Additional file 2 Policy briefs for strategies by level of evidence. Click here for file

## References

[B1] Feudtner C Noonan KG Poorer health: the persistent and protean connections between poverty, social inequality, and child well-being Arch Pediatr Adolesc Med 2009 13 7 668 670 10.1001/archpediatrics.2009.118 19581554

[B2] Marmot M The influence of income on health: views of an epidemiologist Health Aff (Millwood) 2002 13 2 31 46 10.1377/hlthaff.21.2.31 11900185

[B3] Braveman P Cubbin C Egerter S Williams DR Pamuk E Socioeconomic disparities in health in the United States: what the patterns tell Us Am J Public Health 2010 13 1 s186 s196 20147693 10.2105/AJPH.2009.166082 PMC2837459

[B4] Levine R Foster J Fullilove R Black-white inequalities in mortality and life expectancy Public Health Rep 2001 13 477 483 10.1093/phr/116.5.474 PMC1497364 12042611

[B5] Flores G Fuentes-Afflick E Barbot O The health of Latino children: urgent priorities, unanswered questions, and a research agenda Am Med Assoc 2002 13 1 82 90 10.1001/jama.288.1.82 12090866

[B6] Diez Roux AV Investigating neighborhood and area effects on health Am J Public Health 2001 13 11 1783 1789 10.2105/AJPH.91.11.1783 11684601 PMC1446876

[B7] Pickett K Pearl M Multilevel analyses of neighbourhood socioeconomic context and health outcomes: a critical review J Epidemiol Comm Health 2001 13 2 111 122 10.1136/jech.55.2.111 PMC1731829 11154250

[B8] National Research Council, Institute of Medicine Preventing mental, emotional and behavioral disorders among young people: progress and possibilities 2009 Washington, DC: National Academies Press 20662125

[B9] Federal Interagency Forum on Child and Family Statistics America’s Children in Brief: Key National Indicators of Well-being, 2012 2012 Washington, DC: U.S. Government Printing Office

[B10] Adler NE Rehkopf DH US disparities in health: descriptions, causes, and mechanisms Annu Rev Public Health 2008 13 235 252 10.1146/annurev.publhealth.29.020907.090852 18031225

[B11] Commission to Build a Healthier America Breaking Through on the Social Determinants of Health and Health Disparities: An approach to message translation 2009 Princeton, New Jersey: Robert Wood Johnson Foundation

[B12] Commission on Social Determinants of Health Closing the gap in a generation: Health equity through action on the social determinants of health. Final Report of the Commission on Social Determinants of Health 2008 Geneva, Switzerland: World Health Organization

[B13] US Department of Education Promise Neighborhoods 2012 http://www2.ed.gov/programs/promiseneighborhoods/index.html. Accessed 12/14/12, 2012

[B14] Harlem Children’s Zone Harlem Children’s Zone 2012 http://www.hcz.org. Accessed December 14, 2012, 2012

[B15] Komro KA Flay BR Biglan A the Promise Neighborhoods Research Consoritum Creating nurturing environments: a science-based framework for promoting child health and development within high-poverty neighborhoods Clin Child Fam Psychol Rev 2011 13 2 111 10.1007/s10567-011-0095-2 21468644 PMC3686471

[B16] Baron J Applying evidence to social programs The New York Times 2012 http://economix.blogs.nytimes.com/2012/11/29/applying-evidence-to-social-programs/

[B17] Haskins R Baron J Building the Connection between Policy and Evidence: The Obama evidene-based initiatives 2011 London, England: Nesta

[B18] American Academy of Pediatrics The Pediatrician’s role in community pediatrics Pediatrics 2005 13 1092 1094 15805396 10.1542/peds.2004-2680

[B19] Brownson RC Chriqui JF Stamatakis KA Understanding evidence-based public health policy Am J Public Health 2009 13 9 1576 1587 10.2105/AJPH.2008.156224 19608941 PMC2724448

[B20] Oxford Centre for Evidence-Based Medicine The Oxford 2011 Levels of Evidence 2011 Oxford, England: Oxford Centre for Evidence-Based Medicine

[B21] Briss PA Zaza S Pappaioanou M Developing an evidence-based guide to community preventive services–methods Am J Prev Med 2000 13 35 43 10.1016/S0749-3797(99)00119-1 10806978

[B22] Flay BR Biglan A Boruch RF Standards of evidence: criteria for efficacy, effectiveness and dissemination Prev Sci 2005 13 3 151 173 10.1007/s11121-005-5553-y 16365954

[B23] Heller DE Student price response in higher education: an update to Leslie and brinkman J High Educ 1997 13 6 624 659 10.2307/2959966

[B24] American Psychological Association Zero Tolerance Task Force Are zero tolerance policies effective in the schools? An evidentiary review and recommendations Am Psychol 2008 13 9 852 862 19086747 10.1037/0003-066X.63.9.852

[B25] McGowan A Hahn R Liberman A Effects on violence of laws and policies facilitating the transfer of juveniles from the juvenile justice system to the adult justice system: a systemic review Am J Prev Med 2007 13 4 Suppl S7 S28 17386331 10.1016/j.amepre.2006.12.003

[B26] Lavis JN How can we support the use of systematic reviews in policymaking? PLoS Med 2009 13 11 e1000141 10.1371/journal.pmed.1000141 19936223 PMC2777391

[B27] Berkman LF Unintended consequences of social and economic policies for population health: towards a more intentional approach Eur J Public Health 2011 13 5 547 548 10.1093/eurpub/ckr124 21953945

[B28] Stahl T Wismar M Ollila E Lahtinen E Leppo K Health in All Policies: Prospects and potentials 2006 Helsinki, Finland: Finland Ministry of Social Affairs and Health

[B29] Rabin BA Brownson RC Haire-Joshu D Kreuter MW Weaver NL A glossary for dissemination and implementation research in health J Public Health Manag Pract 2008 13 117 123 10.1097/01.PHH.0000311888.06252.bb 18287916

