# Supplementary material for: Beyond the clinic: improving child health through evidence-based community development
Source: BMC Pediatr. 2013 Oct 21;13:172. doi: 10.1186/1471-2431-13-172 (PMC4016148; doi:10.1186/1471-2431-13-172)
Supplement: Additional file 1 — Policy-relevant community strategies by level of evidence. [file 1471-2431-13-172-S1.docx]

| **Additional file 1a.** Policy-relevant community strategies meeting criteria for efficacy based on a minimum of 2 high-quality trials (Level 1). | | | | | | |
| --- | --- | --- | --- | --- | --- | --- |
|  |  |  |  |  |  | |
| **Domain** | **Policy^a^** | **Reference** | **Policy Component** | **Direction** | **Outcome** | **Magnitude of Effect^b^** |
|  |  |  |  |  |  |  |
| Income & Resources | Affordable Housing: Tenant-Based Rental Assistance Programs | Anderson[^1^](#_ENREF_1) et al, 2003a |  | ↓ | Neighborhood social disorder | Small to large |
|  |  |  |  | ↓ | Experience of victimization within the neighborhood | Small |
|  |  |  |  |  |  |  |
|  | Child Mental Health Programs | Waddell[^2^](#_ENREF_2) et al, 2007 |  | ↓ | Conduct disorder symptoms | Small |
|  |  |  |  | ↓ | Diagnosed conduct disorder | Small |
|  |  |  |  | ↓ | Diagnostic measures for depression | Small |
|  |  |  |  |  |  |  |
| Social Cohesion | Alternatives to Incarceration | Latimer[^3^](#_ENREF_3) et al, 2005 | Restorative justice programs | ↑ | Restitution compliance | Medium |
|  |  |  |  | ↓ | Recidivism | Small |
|  |  |  |  | ↑ | Victim & offender satisfaction | Small |
|  |  |  | Drug courts | ↓ | Costs related to prison or jail sentences | NR |
|  |  |  |  | ↓ | Re-arrest of drug offenses | NR |
|  |  |  |  |  |  |  |
|  | Community-Based Participatory Research | Viswanathan^[4](#_ENREF_4)^ et al, 2004 |  | ↑ | Community capacity, grant funding, and job creation | NR |
|  |  |  |  | ↑ | Intervention quality | NR |
|  |  |  |  |  |  |  |
|  | Mentoring Programs | Tolan^[5](#_ENREF_5)^ et al, 2008 |  | ↓ | Aggression | Medium |
|  |  |  |  | ↑ | Academic achievement | Small |
|  |  |  |  | ↓ | Delinquency | Small |
|  |  |  |  |  |  |  |
|  | Neighborhood Watch Programs | Bennett[^6^](#_ENREF_6) et al, 2008 |  | ↓ | Crime | Small to medium |
|  |  |  |  |  |  |  |
|  | Organizational Changes to the Psychosocial Work Environment | Bambra^[7](#_ENREF_7)^ et al, 2009 | Increasing employee control | ↑ | Employee health | NR |
|  |  |  | Changes to shift work | ↑ | Work-life balance | NR |
|  |  |  |  | ↑ | Mental health | NR |
|  |  |  | Government management of public agencies and industries | ↑ | Job security | NR |
|  |  |  |  | ↓ | Job stress | NR |
|  |  |  | Greater regulation and inspection of workplace safety | ↓ | Injury rates | NR |
|  |  |  |  |  |  |  |
|  |  |  |  |  |  |  |
|  | Prisoner Re-entry | Seiter & Kadela,[^8^](#_ENREF_8) 2003 | Vocational training and work release programs | ↓ | Recidivism and re-arrest | NR |
|  |  |  |  | ↑ | Job readiness | NR |
|  |  |  | Prerelease programs | ↓ | Recidivism | NR |
|  |  |  | Drug rehabilitation | ↑ | Ease of transition from prison to community | NR |
|  |  |  |  |  |  |  |
| Physical Environment | Access to Places for Physical Activity | Kahn[^9^](#_ENREF_9) et al, 2002 |  | ↑ | Frequency of physical activity | Medium |
|  |  |  |  | ↑ | Aerobic capacity | Small |
|  |  |  |  | ↑ | Energy expenditure | Small |
|  |  |  |  | ↑ | Leisure-time physical activity | Small |
|  |  |  |  |  |  |  |
|  | Alcoholic Beverage Excise Taxes | Wagenaar^[10](#_ENREF_10)^ et al, 2009; Wagenaar^[11](#_ENREF_11)^ et al, 2010 |  | ↓ | Alcohol-related morbidity/mortality | Medium |
|  |  |  |  | ↓ | Consumption of alcohol by heavy drinkers | Small |
|  |  |  |  | ↓ | Consumption of alcohol in general | Small |
|  |  |  |  | ↓ | Consumption of beer, wine, and spirits | Small |
|  |  |  |  | ↓ | Crime | Small |
|  |  |  |  | ↓ | Sexually transmitted infections | Small |
|  |  |  |  | ↓ | Traffic crash deaths | Small |
|  |  |  |  | ↓ | Violence | Small |
|  |  |  |  |  |  |  |
|  | Alcohol Outlet Density | Campbell[^12^](#_ENREF_12) et al, 2009 |  | ↓ | Alcohol consumption | Large |
|  |  |  |  | ↓ | Interpersonal violence | Large |
|  |  |  |  | ↓ | Unintentional injury | Large |
|  |  |  |  | ↓ | Crime | Small |
|  |  |  |  |  |  |  |
|  | Bicycle Helmet Use | Karkhaneh^[13](#_ENREF_13)^ et al, 2006; Thompson[^14^](#_ENREF_14) et al, 1999 |  | ↑ | Bicycle helmet use | Large |
|  |  |  |  | ↓ | Bicycle-related head injuries | Large |
|  |  |  |  |  |  |  |
|  | Booster Seat Use | Ehiri^[15](#_ENREF_15)^ et al, 2006 | Booster seat distribution and education | ↑ | Ownership or use of booster seats | Large |
|  |  |  | Booster seat intervention | ↑ | Booster seat use | Large |
|  |  |  | Education intervention | ↑ | Ownership or use of booster seats | Small |
|  |  |  |  |  |  |  |
|  | Drug Law Enforcement Programs | Mazerolle^[16](#_ENREF_16)^ et al, 2007 | Problem-oriented and community-wide policing | ↓ | Total calls for service | Large |
|  |  |  |  | ↓ | Drug-related calls for service | Medium |
|  |  |  | Hot spot policing | ↓ | Total offenses | Small |
|  |  |  |  |  |  |  |
|  | Enforcement of Minimum Legal Drinking Age Laws | Wagenaar & Toomey,[^17^](#_ENREF_17) 2002 |  | ↓ | Alcohol consumption among underage youth | NR |
|  |  |  |  | ↓ | Rates of alcohol-related health and social problems | NR |
|  |  |  |  | ↓ | Traffic crashes | NR |
|  |  |  |  |  |  |  |
|  | Graduated Driver Licensing | Hartling^[18](#_ENREF_18)^ et al, 2005 |  | ↓ | Injury crash rates among 16-year-old drivers | Medium |
|  |  |  |  | ↓ | Overall crash rate among 16-year-old drivers | Medium |
|  |  |  |  |  |  |  |
|  | Hours of Sale for Alcohol | Popova^[19](#_ENREF_19)^ et al, 2009; Stockwell & Chikritzhs,[^20^](#_ENREF_20) 2009 |  | ↓ | Alcohol consumption and related harm | NR |
|  |  |  |  |  |  |  |
|  | Red Light Cameras | Aeron-Thomas & Hess,[^21^](#_ENREF_21) 2005 |  | ↓ | Casualty crashes at intersections with traffic lights | Small |
|  |  |  |  | ↓ | Right-angle casualty crashes at intersections with traffic lights | Small |
|  |  |  |  |  |  |  |
|  | Responsible Beverage Service and Enforcement | Wagenaar & Tobler,[^22^](#_ENREF_22) 2007 | RBS enforcement | ↓ | Sales to underage youth | Medium |
|  |  |  | RBS training | ↓ | Alcohol consumption and blood alcohol concentrations among patrons | NR |
|  |  |  |  | ↓ | Alcohol-related traffic crashes | NR |
|  |  |  |  | ↑ | Responsible service practices | NR |
|  |  |  |  | ↑ | Server knowledge and beliefs | NR |
|  |  |  |  |  |  |  |
|  | Safety Belt Laws and Enforcement | Dinh-Zarr^[23](#_ENREF_23)^ et al, 2001 | Enhanced enforcement programs | ↑ | Observed safety belt use | Medium |
|  |  |  | Safety belt laws & enhanced enforcement programs | ↓ | Fatal injuries | Small |
|  |  |  | Safety belt laws | ↓ | Non-fatal injuries | Small |
|  |  |  | Safety belt laws | ↑ | Self-reported safety belt use | Small |
|  |  |  |  |  |  |  |
|  | Street Lighting | Beyer & Ker,[^24^](#_ENREF_24) 2009; Welsh & Farrington,[^25^](#_ENREF_25) 2008 |  | ↓ | Crime | Medium |
|  |  |  |  | ↓ | Injury crashes | Medium |
|  |  |  |  | ↓ | Traffic crashes | Medium |
|  |  |  |  |  |  |  |
|  | Tobacco Advertising Restrictions | Saffer & Chaloupka,[^26^](#_ENREF_26) 2000; Willemsen & De Zwart,[^27^](#_ENREF_27) 1999 | Comprehensive bans | ↓ | Youth tobacco use | Small |
|  |  |  |  |  |  |  |
|  | Tobacco Excise Taxes | Hopkins[^28^](#_ENREF_28) et al, 2001 |  | ↓ | Quantity of product consumed by adolescent users | Small |
|  |  |  |  | ↓ | Tobacco use in both adolescents and young adults | Small |
|  |  |  |  | ↓ | Tobacco use participation among young adults | Small |
|  |  |  |  | ↓ | Overall prevalence of tobacco product use and consumption of tobacco products | NR |
|  |  |  |  |  |  |  |
|  | Smoke-Free Policies | Fichtenberg & Glantz,[^29^](#_ENREF_29) 2002; Hopkins[^28^](#_ENREF_28) et al, 2001 | Smoke-free workplace policies | ↓ | Self-reported and measured environmental tobacco smoke presence | Large |
|  |  |  |  | ↓ | Consumption of cigarettes per day per smoker | Medium |
|  |  |  |  | ↓ | Likelihood of teenagers who worked in smoke-free worksites to ever smoke | Medium |
|  |  |  |  | ↓ | Total cigarette consumption per employee | Medium |
|  |  |  |  | ↓ | Smoking prevalence | Small |
|  |  |  | Legislation restricting smoking in public and in workplaces | ↓ | Consumption of cigarettes per day per capita | Small |
|  |  |  |  | ↓ | Smoking prevalence | Small |
|  |  |  |  |  |  |  |
|  | Urban Design and Land Use Policies | Heath[^30^](#_ENREF_30) et al, 2006 |  | ↑ | Physical activity | Medium |
|  |  |  |  |  |  |  |
|  | Water Fluoridation | Richmond,[^31^](#_ENREF_31) 1985 |  | ↓ | Dental cavities | Medium |
|  |  |  |  |  |  |  |
|  | Hot Spot Policing | Braga,[^32^](#_ENREF_32) 2007 |  | ↓ | Citizens calls for services | Small to Large |
|  |  |  |  | ↓ | Citizen disorder calls | Small |
|  |  |  |  |  |  |  |
| Family Influences | Access to Affordable (or Free) Quality Childcare Services | Zoritch^[33](#_ENREF_33)^ et al, 2000 |  | ↓ | Arrests for drug deals | Large |
|  |  |  |  | ↓ | Five or more arrests | Large |
|  |  |  |  | ↓ | Grade retention | Medium |
|  |  |  |  | ↓ | Special education | Medium |
|  |  |  |  | ↑ | IQ at ages 3 & 5 | Small |
|  |  |  |  |  |  |  |
|  | Breast Feeding Support | Dennis[^34^](#_ENREF_34) 2002 | Peer support groups | ↑ | Exclusive breastfeeding and mother confidence | Small |
|  |  |  | Professional support | ↑ | Duration of breastfeeding (better results for face to face support vs. telephone contact) | Small |
|  |  |  |  |  |  |  |
|  | Child Placement when Taken from Home | Winkour[^35^](#_ENREF_35)et al, 2009 | Kinship care | ↑ | Reporting of positive emotional health | Large |
|  |  |  |  | ↑ | Competence | Medium |
|  |  |  |  | ↓ | Behavior problems | Small |
|  |  |  | Non-kinship (foster) care | ↑ | Likelihood of receiving mental health services | Large |
|  |  |  |  | ↑ | Mental illness | Large |
|  |  |  |  | ↑ | Placement settings | Large |
|  |  |  |  |  |  |  |
|  | Home Safety Education | Kendrick[^36^](#_ENREF_36) et al, 2007 |  | ↑ | Likelihood of owning a functioning smoke alarm | Large |
|  |  |  |  | ↑ | Poison control number accessibility | Large |
|  |  |  |  | ↑ | Possession of syrup of ipecac | Large |
|  |  |  |  | ↑ | Proper storage of cleaning products, medicines, and sharp objects | Large |
|  |  |  |  | ↑ | Use of socket covers | Large |
|  |  |  |  | ↑ | Likelihood of having a safe hot tap water temperature | Medium |
|  |  |  |  | ↑ | Use of fitted stair gates | Small |
|  |  |  |  |  |  |  |
|  | Home Visiting Programs | Nievar & van Egeren,[^37^](#_ENREF_37) 2005; Sweet & Appelbaum,[^38^](#_ENREF_38) 2004 |  | ↑ | Positive parenting behavior | Small |
|  |  |  |  | ↑ | Child cognitive and social-emotional development | Small |
|  |  |  |  | ↑ | Risks for potential child abuse | Small |
|  |  |  |  |  |  |  |
|  | Parent Involvement in Child's Education | Nye[^39^](#_ENREF_39) et al, 2006 |  | ↑ | Academic performance among elementary school-aged children (greatest benefits occur in reading) | Medium |
|  |  |  |  |  |  |  |
|  | Prenatal Micronutrient Supplementation | Prakesh & Ohlsson,[^40^](#_ENREF_40) 2009 |  | ↓ | Low birth weight infants | Small to medium |
|  |  |  |  | ↑ | Birth weight | Small |
|  |  |  |  |  |  |  |
|  | Treatment Foster Care | MacDonald & Turner,[^41^](#_ENREF_41) 2007 |  | ↓ | Antisocial behavior, number of days children abscond from placement, number of criminal referrals, and time spent in locked setting | NR |
|  |  |  |  | ↑ | School attendance and homework completion | NR |
|  |  |  |  |  |  |  |
| School Influences | After-School Programs that Include Academic Support Services | Lauer,[^42^](#_ENREF_42) 2006 |  | ↑ | Math achievement in at-risk students | Medium |
|  |  |  |  | ↑ | Reading achievement in at-risk students | Small |
|  |  |  |  |  |  |  |
|  | Class Size Reductions | Shin & Chung,[^43^](#_ENREF_43) 2009 |  | ↑ | Students' academic performance | Small |
|  |  |  |  |  |  |  |
|  | Positive Behavior Support | Wilson[^44^](#_ENREF_44) et al, 2001 |  | ↓ | Alcohol and drug use | Small |
|  |  |  |  | ↓ | Problem behaviors (rebelliousness, antisocial or disrespectful behaviors) | Small |
|  |  |  |  | ↓ | School dropout /truancy | Small |
|  |  |  |  |  |  |  |
|  | Quality Preschool/Early Childhood Education | Anderson[^45^](#_ENREF_45) et al, 2003b; Camilli^[46](#_ENREF_46)^ et al, 2010 |  | ↑ | Child health screenings | Medium to large |
|  |  |  |  | ↑ | Socio/emotional and anti-social health | Small |
|  |  |  |  | ↑ | Academic, school readiness, and IQ test scores | Medium |
|  |  |  |  | ↓ | Negative social outcomes, including teen pregnancy, teen arrests and welfare use | Medium |
|  |  |  |  | ↑ | Positive social outcomes, including high school graduation, employment and home ownership | Medium |
|  |  |  |  | ↓ | Grade retention and special education | Small |
|  |  |  |  | ↑ | Positive family outcomes, including parental high school graduation, income above the poverty line, parental employment, and not receiving public assistance | Small |
|  |  |  |  |  |  |  |
|  | School-Based Physical Activity Programs | Kahn[^9^](#_ENREF_9) et al, 2002; Strong[^47^](#_ENREF_47) et al, 2005 |  | ↑ | Class time spent in moderate-vigorous physical activity | Medium |
|  |  |  |  | ↑ | Academic performance | Small |
|  |  |  |  | ↑ | Aerobic capacity | Small |
|  |  |  |  | ↓ | Body fat | Small |
|  |  |  |  | ↑ | Muscular endurance, flexibility, health-related knowledge, and physical activity self-efficacy | NR |
|  |  |  |  |  |  |  |
|  | School Nutrition Standards for School Lunch Programs | Jaime & Lock,[^48^](#_ENREF_48) 2009 |  | ↓ | Saturated fat intake | Medium |
|  |  |  |  | ↓ | Total fat intake | Medium |
|  |  |  |  | ↑ | Fruit/vegetable availability | Small |
|  |  |  |  | ↑ | Fruit/vegetable intake | Small |
|  |  |  |  | ↓ | Total and saturated fat on school menu | NR |
|  |  |  |  |  |  |  |
|  | School Vocational Training Programs | Kulik,[^49^](#_ENREF_49) 1994 |  | ↓ | High School dropout | Small |
|  |  |  |  | ↑ | Job satisfaction | Small |
|  |  |  |  |  |  |  |
|  | Sexual Health Education and Contraceptive Interventions | Oringanje et al, 2009[^50^](#_ENREF_50); Underhill[^51^](#_ENREF_51) et al, 2009 | Combined education and contraceptive interventions | ↓ | Unintentional pregnancies | Large |
|  |  |  | Abstinence-plus programs | ↑ | HIV/AIDS knowledge | NR |
|  |  |  |  | ↓ | Risky sexual behavior | NR |
|  |  |  |  |  |  |  |
|  | Volunteer Tutoring Programs | Ritter[^52^](#_ENREF_52) et al, 2006 |  | ↑ | Overall reading | Medium |
|  |  |  |  | ↑ | Reading letters and words | Medium |
|  |  |  |  | ↑ | Reading oral fluency | Medium |
|  |  |  |  | ↑ | Writing skill | Medium |
|  |  |  |  | ↑ | Global domain reading | Small |
|  |  |  |  |  |  |  |
| Peer Influences | After-School Programs that Promote Personal and Social Skills | Durlak & Weissberg,[^53^](#_ENREF_53) 2007; Durlak^[54](#_ENREF_54)^ et al, 2010 |  | ↓ | Self-reported drug use | Small |
|  |  |  |  | ↑ | Positive social behaviors | Small |
|  |  |  |  | ↑ | Behavioral control | Medium |
|  |  |  |  | ↑ | Child self-perceptions (self-esteem, self-concept, self-efficacy) | Medium |
|  |  |  |  | ↑ | School bonding | Medium |
|  |  |  |  | ↑ | High grades in school | Small |
|  |  |  |  | ↑ | Performance on standardized school achievement tests | Small |
|  |  |  |  | ↓ | Problem behaviors (non-compliance, aggression, delinquent acts, disciplinary referrals, rebellion) | Medium |
|  |  |  |  |  |  |  |
|  |  |  |  |  |  |  |
|  |  |  |  |  |  |  |
|  |  |  |  |  |  |  |
|  | School-Based Efforts to Reduce Bullying | Farrington & Ttofi,[^55^](#_ENREF_55) 2009 |  | ↓ | Bullying | Small to medium |
|  |  |  |  | ↓ | Victimization | Small to medium |
|  |  |  |  |  |  |  |

**Note.** ^a^Policy briefs (available in Additional File 1b or at <http://promiseneighborhoods.org>) provide: (1) rationale for the policy, (2) outline of targeted outcomes, (3) summary and appraisal of the available evidence, and (4) real examples of the policy in action in community settings. ^b^NR = effect estimates not reported in published review.

| **Additional file 2a.** Policy-relevant community strategies with evidence of positive outcomes from high-quality observational studies (Level 2). | | | | | | |
| --- | --- | --- | --- | --- | --- | --- |
|  |  |  |  |  |  | |
| **Domain** | **Policy^a^** | **Reference** | **Policy Component** | **Direction** | **Outcome** | **Magnitude of Effect^b^** |
|  |  |  |  |  |  |  |
| Income & Resources | Active Labor Market Policies | Card[^56^](#_ENREF_56) et al, 2009 | Classroom and on-the-job training | ↑ | Job placement | Small |
|  |  |  | Job search assistance programs | ↑ | Job placement | Small |
|  |  |  |  |  |  |  |
|  |  |  |  |  |  |  |
|  | Child Health-Care Access | Zambrana & Carter-Porkras,[^57^](#_ENREF_57) 2004 | Streamlined application process | ↓ | Incomplete applications for the State Children’s Health Insurance Program (SCHIP) | Medium |
|  |  |  | Simplified application form | ↑ | SCHIP enrollment | Small |
|  |  |  | Expanded coverage to parents | ↑ | SCHIP enrollment | Small |
|  |  |  |  |  |  |  |
|  | College Grants & Financial Aid | Leslie & Brinkman,[^58^](#_ENREF_58) 1987 |  | ↑ | Lower-income student college enrollment | Medium |
|  |  |  |  | ↑ | Middle-income student college enrollment | Small |
|  |  |  |  |  |  |  |
|  | Condom Subsidies & Social Marketing Programs | Harvey,[^59^](#_ENREF_59) 1994 |  | ↑ | Condom use | NR |
|  |  |  |  |  |  |  |
|  | Living Wage Ordinances | Fairris & Reich,[^60^](#_ENREF_60) 2005; Neumark & Adams,[^61^](#_ENREF_61) 2003; Pollin,[^62^](#_ENREF_62) 2005 |  | ↑ | Contractor operating costs | Small |
|  |  |  |  | ↓ | Employee turnover | Small |
|  |  |  |  | ↑ | Low-skill job pay | Small |
|  |  |  |  | ↓ | Urban poverty | Small |
|  |  |  |  | ↑ | Wages of low-wage workers | Small |
|  |  |  |  |  |  |  |
| Social Cohesion | Anti-discrimination & Diversity Policies | Kalev^[63](#_ENREF_63)^ et al, 2006 | Diversity committee or full-time diversity staff | ↑ | Managerial placement of women and blacks | Small to medium |
|  |  |  |  |  |  |  |
|  | Community-Based Arts Programs | Newman[^64^](#_ENREF_64) et al, 2003 |  | ↑ | Economic-level: new jobs, increased art sales, inward community investment | NR |
|  |  |  |  | ↑ | Personal-level: making friends, happiness, creativity, confidence, and reduced isolation | NR |
|  |  |  |  | ↑ | Educational-level: improved school performance | NR |
|  |  |  |  | ↑ | Social-level: community understanding, unity | NR |
|  |  |  |  |  |  |  |
|  | Employee Share Ownership & Profit-sharing | Doucouliagos,[^65^](#_ENREF_65) 1995; Perotin & Robinson,[^66^](#_ENREF_66) 2003 | Worker participation in decision-making, worker ownership, or profit-sharing | ↑ | Worker productivity | Small |
|  |  |  |  |  |  |  |
| Physical Environment | Area-Wide Traffic Calming Devices | Bunn[^67^](#_ENREF_67) et al, 2003 |  | ↓ | Road traffic injuries | Small |
|  |  |  |  |  |  |  |
|  | Farm-to-School Programs | Carlsson & Williams,[^68^](#_ENREF_68) 2008 |  | ↑ | Building community capacity and relationships | NR |
|  |  |  |  | ↑ | Easier and less costly job for food service managers to provide healthy food to children | NR |
|  |  |  |  | ↑ | Experiential education for sustainability | NR |
|  |  |  |  | ↑ | Fresh food access at school | NR |
|  |  |  |  | ↑ | Money infusion into local economy | NR |
|  |  |  |  | ↑ | Steady income and convenient delivery for local farmers | NR |
|  |  |  |  | ↓ | Urban sprawl | NR |
|  |  |  |  |  |  |  |
|  | Point of Purchase Nutrition Strategies | Seymour[^69^](#_ENREF_69) et al, 2004 | Incentives combined with nutritional information | ↑ | Targeted food sales | NR |
|  |  |  | Increased healthy food availability | ↑ | Healthy foods/snacks sales | NR |
|  |  |  | Point of purchase nutritional information | ↑ | Targeted food sales and positive dietary patterns within workplace and university settings | NR |
|  |  |  |  |  |  |  |
|  | Pool Fencing for Preventing Drowning | Thompson & Rivara,[^70^](#_ENREF_70) 1998 |  | ↓ | Drowning risk | Large |
|  |  |  |  |  |  |  |
|  | Speed Enforcement Detection Devices | Wilson[^71^](#_ENREF_71) et al, 2006 |  | ↓ | Crashes resulting in injury | Small to medium |
|  |  |  |  | ↓ | Road traffic crashes | Small to medium |
|  |  |  |  | ↓ | Average speeds | NR |
|  |  |  |  |  |  |  |
| Family Influences | Early Childhood Health Promotion | Guyer^[72](#_ENREF_72)^ et al, 2009 | Smoking cessation for pregnant women | ↓ | Parent-reported smoking at home or household air nicotine measures | Medium |
|  |  |  | Injury prevention programs | ↑ | Parents’ and children's knowledge, safety behaviors, and safety device usage | NR |
|  |  |  |  |  |  |  |
| School Influences | Later School Day Start Time | Millman^[73](#_ENREF_73)^ et al, 2005; Wolfson & Carskadon,[^74^](#_ENREF_74) 2003 |  | ↑ | High school attendance rates | NR |
|  |  |  |  | ↓ | High school dropout rates | NR |
|  |  |  |  | ↑ | Sleep on school nights | NR |
|  |  |  |  | ↑ | Student attention and concentration | NR |
|  |  |  |  |  |  |  |
|  | Modified School Calendars | Cooper[^75^](#_ENREF_75) et al, 2003 |  | ↑ | Academic achievement | Small |
|  |  |  |  |  |  |  |
|  | School-Based Health Centers | Santelli^[76](#_ENREF_76)^ et al, 1996 |  | ↑ | Health services accessibility to students | Medium |
|  |  |  |  |  |  |  |
|  | School Funding | Hedges[^77^](#_ENREF_77) et al, 1994 |  | ↑ | Student academic achievement | Large |
|  |  |  |  |  |  |  |
|  | School Gardens | Blair,[^78^](#_ENREF_78) 2009 |  | ↑ | Elementary student preference for vegetables as snacks | NR |
|  |  |  |  | ↑ | School attitude and pride | NR |
|  |  |  |  | ↑ | Science Scores | NR |
|  |  |  |  | ↑ | Teamwork, student bonding, and interaction with adults and their community | NR |
|  |  |  |  |  |  |  |
|  | School Recess | Pellegrini & Bohn,[^79^](#_ENREF_79) 2005 |  | ↑ | Attention to classroom tasks after recess | NR |
|  |  |  |  |  |  |  |
|  | Teacher Merit Pay | Podgursky & Springer,[^80^](#_ENREF_80) 2007 |  | ↑ | Positive teacher behavior | NR |
|  |  |  |  |  |  |  |
| Peer Influences | N/A |  |  |  |  |  |
|  | | | | | | |

**Note.** ^a^Policy briefs (available in Additional File 2b or at <http://promiseneighborhoods.org>) provide: (1) rationale for the policy, (2) outline of targeted outcomes, (3) summary and appraisal of the available evidence, and (4) real examples of the policy in action in community settings. ^b^NR = effect estimates not reported in published review.

**Additional file 3a.** Policy-relevant community strategies with insufficient evidence to assess efficacy based on published reviews (Level 3).

|  |  |  |  |
| --- | --- | --- | --- |
| **Domain** | **Policy^a^** | **Reference** | **Targeted Outcome** |
| Income & Resources | Conditional Cash Transfer | Lagarde^[81](#_ENREF_81)^ et al, 2007; Lagarde^[82](#_ENREF_82)^ et al, 2009 | Increased use of health services |
|  |  |  | Improved school attendance |
|  |  |  | Reduced poverty |
|  |  |  |  |
|  | Home Nursing Services for Children | Cooper[^83^](#_ENREF_83) et al, 2006 | Reduced hospitalization and health care costs |
|  |  |  |  |
|  | Housing Improvement | Dedman^[84](#_ENREF_84)^ et al, 2001; Shaw,[^85^](#_ENREF_85) 2004; Thomson[^86^](#_ENREF_86) et al, 2001 | Improved mental and physical health |
|  |  |  |  |
|  | In-Community Alcohol and Drug Abuse Treatment Centers | Chanhatasilpa^[87](#_ENREF_87)^ et al, 2000 | Reduced recidivism and re-arrest |
|  |  |  |  |
|  | Mixed-Income Housing | Anderson[^1^](#_ENREF_1) et al, 2003 | Improved mental and physical health |
|  |  |  | Reduced neighborhood crime and segregation |
|  |  |  |  |
|  | Transportation Policies | Heath[^30^](#_ENREF_30) et al, 2006 | Increased physical activity |
|  |  |  |  |
|  | Welfare-to-Work | Smeslund^[88](#_ENREF_88)^ et al, 2006 | Increased likelihood of employment/earnings of welfare recipients |
|  |  |  | Reduced welfare payments to recipients |
|  |  |  |  |
| Social Cohesion | Collective or Community Kitchens | Engler-Stringer & Berenbaum,[^89^](#_ENREF_89) 2005 | Improved social cohesion and health |
|  |  |  |  |
|  | Community-Driven Development | Mansuri & Vijayendra,[^90^](#_ENREF_90) 2004 | Improved community infrastructure and welfare outcomes |
|  |  |  |  |
|  | Enterprise Zones | Greenbaum & Landers,[^91^](#_ENREF_91) 2009 | Increased employment and business in distressed areas |
|  |  |  |  |
|  | Independent Living Programs for Young People Leaving the Care System | Donkoh^[92](#_ENREF_92)^ et al, 2006 | Improved educational attainment and increased employment |
|  |  |  | Improved health status |
|  |  |  | Stable housing |
|  |  |  |  |
| Physical Environment | Complete Streets | Heath[^30^](#_ENREF_30) et al, 2006 | Increased traffic safety |
|  |  |  |  |
|  | Condom Availability in Schools | Kirby,[^93^](#_ENREF_93) 2002 | Improved safe sex practices in teens |
|  |  |  |  |
|  | Enhanced Enforcement Gun Laws | Koper & Mayo-Wilson,[^94^](#_ENREF_94) 2006 | Reduced gun violence at high-risk places and times |
|  |  |  |  |
|  | Firearm Policies | Hahn[^95^](#_ENREF_95) et al, 2005 | Increased ability to enforce firearms laws and trace sources of illegally possessed firearms |
|  |  |  | Reduced violence |
|  |  |  |  |
|  | Food Taxes and Subsidies | Powell & Chaloupka,[^96^](#_ENREF_96) 2009; Thow^[97](#_ENREF_97)^ et al, 2010 | Reduced food consumption, body weight, and disease incidence |
|  |  |  |  |
|  |  |  |  |
|  |  |  |  |
|  | Limiting Alcohol Advertising | Anderson[^98^](#_ENREF_98) et al, 2009; Grube,[^99^](#_ENREF_99) 2005; Smith & Foxcroft,[^100^](#_ENREF_100) 2009 | Reduced youth drinking and problem behaviors |
|  |  |  |  |
|  | Limiting Pesticide Exposures in Workers | Keifer,[^101^](#_ENREF_101) 2000 | Improved health of workers |
|  |  |  | Reduced occupational pesticide exposure |
|  |  |  |  |
|  | Social Host Liability and Keg Registration | Grube,[^99^](#_ENREF_99) 2005 | Reduced youth alcohol use and unintentional injuries |
|  |  |  | Reduced blood alcohol concentrations (BACs) among adults |
|  |  |  | Reduce alcohol-related traffic crashes and fatalities |
|  |  |  |  |
|  | Supermarket Access | Ford & Dzewaltowski,[^102^](#_ENREF_102) 2008; Giskes^[103](#_ENREF_103)^ et al, 2010; Larson[^104^](#_ENREF_104) et al, 2009 | Reduced obesity and improved diets |
|  |  |  |  |
|  | Tobacco Sales Laws Enforcement | Stead & Lancaster,[^105^](#_ENREF_105) 2005 | Improved retailer compliance |
|  |  |  | Reduced access/use of tobacco products by minors |
|  |  |  |  |
| Family Influences | Mental Health Services for Parents | Craig,[^106^](#_ENREF_106) 2004; Mayberry & Reupert,[^107^](#_ENREF_107) 2009 | Positive parent-child relationships and improved child social adjustment |
|  |  |  |  |
| School Influences | Adequate Yearly Progress | Au,[^108^](#_ENREF_108) 2007 | Improved academic performance |
|  |  |  | Reduced achievement gap |
|  |  |  |  |
|  | Charter Schools | Bulkley & Fisler,[^109^](#_ENREF_109) 2003; Miron & Nelson,[^110^](#_ENREF_110) 2001 | Improved academic achievement |
|  |  |  |  |
|  | Early College Programs | Bailey & Karp,[^111^](#_ENREF_111) 2003 | Increase college enrollment and success in otherwise non-college bound students |
|  |  |  | Give advanced students an opportunity to enter college more prepared |
|  |  |  |  |
|  | K-8 School Grade Configuration | Coladarci & Hancock,[^112^](#_ENREF_112) 2002 | Improved academic achievement |
|  |  |  |  |
|  | School Music Programs | Wolff,[^113^](#_ENREF_113) 2004 | Improved academic achievement and social/emotional growth |
|  |  |  |  |
|  | Vaccination for Daycare | Shefer^[114](#_ENREF_114)^ et al, 1999 | Increased vaccination coverage |
|  |  |  | Reduced illnesses in school-children |
| Peer Influences | N/A |  |  |
| **Note.** ^a^Policy briefs (available in Additional File 3b or at <http://promiseneighborhoods.org>) provide: (1) rationale for the policy, (2) outline of targeted outcomes, (3) summary and appraisal of the available evidence, and (4) real examples of the policy in action in community settings. | | | |

**Additional File References**

**1.** Anderson LM, St. Charles J, Fullilove MT, Scrimshaw SC, Fielding JE. Providing affordable family housing and reducing residential segregation by income: A systematic review. *Am. J. Prev. Med.* 2003;24(3S):S47-S67.

**2.** Waddell C, Hua JM, Garland OM, De V, Peters R, McEwan K. Preventing mental disorders in children: A systematic review to inform policy-making *Candian Journal of Public Health.* 2007;98(3):166-173.

**3.** Latimer J, Dowden C, Muise D. The Effectiveness of Restorative Justice Practices: A Meta-Analysis. *The Prison Journal.* 2005;85(2):127-144.

**4.** Viswanathan M, Ammerman A, Eng E, et al. *Community-based participatory research: Assessing the evidence*: AHRQ;2004.

**5.** Tolan P, Henry D, Schoeny M, Bass A. Mentoring interventions to affect juvenile delinquency and associated problems. *Campbell Systemic Reviews.* 2008;16.

**6.** Bennett T, Holloway K, Farrington D. The effectiveness of neighborhood watch. *Campbell Systemic Reviews.* 2008;18.

**7.** Bambra C, Gibson M, Sowden AJ, Wright K, Whitehead M, Petticrew M. Working for health? Evidence from systematic reviews on the effects on health and health inequalities of organizational changes to the psychosocial work environment. *Prev. Med.* 2009;48:454-461.

**8.** Seiter RP, Kadela KR. Prisoner reentry: What works, what does not, and what is promising. *Crime Delinquency.* 2003;49(3):360-388.

**9.** Kahn EB, Ramsey LT, Brownson RC, et al. The effectiveness of interventions to increase physical activity: A systematic review. *Am. J. Prev. Med.* 2002;22(4S):73-107.

**10.** Wagenaar AC, Salois MJ, Komro KA. Effects of beverage alcohol price and tax levels on drinking: a meta-analysis of 1003 estimates from 112 studies. *Addiction.* 2009;104:179-190.

**11.** Wagenaar AC, Tobler AL, Komro KA. Effects of alcohol tax price policies on morbidity and mortality: A systematic review. *Am. J. Public Health.* 2010.

**12.** Campbell CA, Hahn RA, Elder R, et al. The effectiveness of limiting alcohol outlet density as a means of reducing excessive alcohol consumption and alcohol-related harms. *Am. J. Prev. Med.* 2009;37(6):556-569.

**13.** Karkhaneh M, Kalenga JC, Hagel BE, Rowe BH. Effectiveness of bicycle helmet legislation to increase helmet use: A systematic review. *Inj. Prev.* 2006;12:76-82.

**14.** Thompson D, Rivara F, Thompson R. Helmets for preventing head and facial injuries in bicyclists. *Cochrane Database Syst. Rev.* 1999(3).

**15.** Ehiri JE, Ejere HOD, Mangnussen L, Emusu D, King W, Osberg SJ. Effects of seating position and appropriate restraint use on the risk of injury to children in motor vehicle crashes. *Pediatrics.* 2006;115:E305-E309.

**16.** Mazerolle L, Soole DW, Rombouts S. Street-level drug law enforcement: A meta-analytic review. *Campbell Systemic Reviews.* 2007;2.

**17.** Wagenaar AC, Toomey TL. Effects of minimum drinking age laws: Review and analyses of the literature from 1960-2000. *J. Stud. Alcohol.* 2002;114:206-225.

**18.** Hartling L, Wiebe N, Russell KR, Petruk J, Spinola C, Klassen TP. Graduated driver licensing for reducing motor vehicle crashes among young drivers. *Cochrane Database Syst. Rev.* 2005;2(CD003300).

**19.** Popova S, Giesbrecht N, Bekmuradov D, Patra J. Hours and days of sale and density of alcohol outlets: Impacts on alcohol consumption and damage: A systematic review. *Alcohol Alcohol.* 2009;44(5):500-516.

**20.** Stockwell T, Chikritzhs T. Do relaxed trading hours for bars and clubs mean more relaxed drinking? A review of international research on the impacts of changes to permitted hours of drinking. *Crime Prevention and Community Safety.* 2009;11:153-170.

**21.** Aeron-Thomas A, Hess S. Red-light cameras for the prevention of road traffic crashes. *Cochrane Database Syst. Rev.* 2005;2(CD003862).

**22.** Wagenaar AC, Tobler AL. Alcohol sales and service to underage youth and intoxicated patrons: Effects of responsible beverage service training and enforcement interventions. *Transportation Research Circular.* 2007;E-C123:141-163.

**23.** Dinh-Zarr TB, Sleet DA, Shults RA, et al. Reviews of Existing Evidence Regarding Interventions to Increase the Use of Safety Belts. *Am. J. Prev. Med.* 2001;21(4S):48-65.

**24.** Beyer FR, Ker K. Street lighting for preventing road traffic injuries. *Cochrane Database Syst. Rev.* 2009;1(CD004728).

**25.** Welsh BP, Farrington DC. Effects of improved street lighting on crime. *Campbell Systemic Reviews.* 2008;1(CD004728).

**26.** Saffer H, Chaloupka F. The Effect of Tobacco Advertising Bans on Tobacco Consumption. *J. Health Econ.* 2000;19(1117):1137.

**27.** Willemsen MC, De Zwart WM. The Effectiveness of Policy and Health Education Strategies for Reducing Adolescent Smoking: A Review of the Evidence. *J. Adolesc.* 1999;22(5):587-599.

**28.** Hopkins DP, Briss PA, Ricard CJ, et al. Reviews of Evidence Regarding Interventions to Reduce Tobacco Use and Exposure to Environmental Tobacco Smoke. *Am. J. Prev. Med.* 2001;20(2 Suppl):16-66.

**29.** Fichtenberg CM, Glantz SA. Effect of Smoke-Free Workplaces on Smoking Behavior: Systematic Review. *Br. Med. J.* 2002;325(7357):188-195.

**30.** Heath GW, Brownson RC, Kruger J, et al. The effectiveness of urban design and land use and transport policies and practices to increase physical activity: a systematic review. *J. Phys. Act. Health.* 2006;3(S1):S55-S76.

**31.** Richmond VL. Thrity years of fluoridation: A review. *Am. J. Clin. Nutr.* 1985;41:129-138.

**32.** Braga AA. The effects of hot spots policing on crime. 2007; Campbell Collaboration systematic review, available at: <http://www.aic.gov.au/campbellcj/reviews/titles.html>.

**33.** Zoritch B, Roberts I, Oakley A. Day care for pre-school children. *Cochrane Database Syst. Rev.* 2000;3(CD00564).

**34.** Dennis CL. Breastfeeding initiation and duration: A 1990-2000 literature review. *J. Obstet. Gynecol. Neonatal Nurs.* 2002;31:12-32.

**35.** Winkour M, Holtan A, Valentine D. Kinship care for the safety, permanency, and well-being of children removed from the home for maltreatment. *Cochrane Database Syst. Rev.* 2009;1(CD006546).

**36.** Kendrick D, Coupland C, Mulvaney C, et al. Home safety education and provision of safety equipment for injury prevention. *Cochrane Database Syst. Rev.* 2007;1(CD005014).

**37.** Nievar MA, van Egeren L. More is better: A meta-analysis of home visiting programs for at-risk families. *Biennial Conference of the Society for Research in Child Development*. Tampa, FL: <http://www.eric.ed.gov;> 2005.

**38.** Sweet MA, Appelbaum MI. Is home visiting an effective strategy? A meta-analytic review of home visiting programs for families with young children. *Child Dev.* 2004;75(5):1435-1456.

**39.** Nye C, Turner H, Schwartz J. Approaches to parent involvement for improving the academic performance of elementary school age children. *Campbell Systemic Reviews.* 2006;4.

**40.** Prakesh SS, Ohlsson A. Effects of prenatal multimicronutrient supplementation on pregnancy outcomes: A meta-analysis. *Candian Medical Association Journal.* 2009;180(12):99-108.

**41.** MacDonald GM, Turner W. Treatment foster care for improving outcomes in children and young people. *Campbell Systemic Reviews.* 2007;9.

**42.** Lauer PA, Akiba, M., Wilderson, S.B., Apthorp, H.S., Snow, D., & Martin-Glenn, M.L. Out-of-school-time programs: A mete-analysis of effects for at-risk students. *Review of Educational Research.* 2006;76(2):275-313.

**43.** Shin IS, Chung JY. Class size and student achievement in the United States: A meta-analysis. *KEDI Journal of Educational Policy.* 2009;6(2):3-19.

**44.** Wilson DB, Gottfredson D, Najaka SS. School-based prevention of problem behaviors: A meta-analysis. *Journal of Quantitative Criminology.* 2001;17:247-272.

**45.** Anderson LM, Shinn C, Fullilove MT, et al. The effectiveness of early childhood development programs: A systematic review. *Am. J. Prev. Med.* 2003;24(3S):32-46.

**46.** Camilli G, Vargas S, Ryan S, Barnett SW. Meta-Analysis of the Effects of Early Education Interventions on Cognitive and Social Development. *Teachers College Record.* 2010;112(3):579-620.

**47.** Strong WB, Malina RM, Bumkie CJR, et al. Evidence based physical activity for school-age youth. *J. Pediatr.* 2005;146:732-737.

**48.** Jaime PC, Lock K. Do school based food and nutrition policies improve diet and reduce obesity? *Prev. Med.* 2009;48:45-53.

**49.** Kulik JA. *Curricular tracks and high school vocational education.* Washington, DC1994.

**50.** Oringanje C, Meremikwu MM, Eko H, Esu E, Meremikwu A, Ehirj JE. Interventions for preventing unintended pregnancies among adolescents. *Cochrane Database Syst. Rev.* 2009;4(CD005215).

**51.** Underhill K, Montgomery P, Operario D. Abstinence-plus programs for HIV infection prevention in high-income countries. *Cochrane Database Syst. Rev.* 2009;1(CD007006).

**52.** Ritter G, Denny G, Albin G, Barnett J, Blankenship V. The effectiveness of bolunteer tutoring programs: A systematic review. *Campbell Systemic Reviews.* 2006;7.

**53.** Durlak JA, Weissberg RP. *The impact of after-school programs that seek to promote personal and social skills.* Chicago, IL: The Collaborative for Academic, Social and Emotional Learning;2007.

**54.** Durlak JA, Weissberg, R.P., Pachan, M. A meta-analysis of after-school programs that seek to promote personal and social skills in children and adolescents. *Am. J. Community Psychol.* 2010;45(3-4):294-309.

**55.** Farrington DP, Ttofi MM. School-Based Programs to Reduce Bullying and Victimization. *Campbell Systemic Reviews.* 2009;2009:6.

**56.** Card D, Kluve J, Weber A. *Active labor market policy evaluations: A meta-analysis*2009.

**57.** Zambrana RE, Carter-Porkras O. Improving health insurance coverage for Latino children: A review of barriers, challenges, and state strategies. *J. Natl. Med. Assoc.* 2004;96(4):508-523.

**58.** Leslie LL, Brinkman PT. Student Price Response in Higher Education. *Journal of Higher Education.* 1987;58:181-204.

**59.** Harvey PD. The impact of condom prices on sales in social marketing programs. *Stud. Fam. Plann.* 1994;25(1):52-58.

**60.** Fairris D, Reich M. The impacts of living wage policies: Introduction to the special issue. *Industrial Relations.* 2005;44(1):1-13.

**61.** Neumark D, Adams S. Do living wage ordinances reduce urban poverty? . *J. Hum. Resour.* 2003;38(3):490-521.

**62.** Pollin R. Evaluating living wage laws in the United States. . *Economic Development Quarterly.* 2005;19(1):3-24.

**63.** Kalev A, Dobbin F, Kelly E. Best practices or best guess? Assessing the efficacy of corporate affirmative action diversity policies. *Am. Sociol. Rev.* 2006;71:589-617.

**64.** Newman T, Curtis K, Stephens J. Do Community-Based Arts Projects Result in Social Gains? A Review of the Literature. *Community Development Journal.* 2003;38(4):310-322.

**65.** Doucouliagos C. Worker participation and productivity in labor-managed and participatory capitalist firms: A meta-analysis. *Ind. Labor Relat. Rev.* 1995;49(1):58-77.

**66.** Perotin V, Robinson A. *Employee participation in profit and ownership: A review of the issues and evidence*2003.

**67.** Bunn F, Collier T, Frost C, et al. Area-wide traffic calming for preventing traffic related injuries. *Cochrane Database Syst. Rev.* 2003;1(CD003110).

**68.** Carlsson L, Williams PL. New approaches to the health promoting school: Participation in sustainable food systems. *Journal of Hunger and Environmental Nutrition.* 2008;3(4):400-417.

**69.** Seymour JD, Yarock AL, Serdula M, Blanck HM, Khan LK. Impact of Nutrition Environmental Interventions on Point-of-Purchase Behavior in Adults: A Review. *Prev. Med.* 2004;39:S108-S136.

**70.** Thompson DC, Rivara F. Pool fencing for preventing drowning of children. *Cochrane Database Syst. Rev.* 1998;1(CD001047).

**71.** Wilson C, Willis C, Hendrikz JK, Bellamy N. Speed enforcement detection devices for preventing road traffic injuries. *Cochrane Database Syst. Rev.* 2006;2(CD004607).

**72.** Guyer B, Ma S, Grason H, et al. Early childhood health promotion and its life course health consequences. *Academic Pediatrics.* 2009;9:142-149.

**73.** Millman RP, American Academy of Pediatrics Committee on Adolescence. Excessive Sleepiness in Adolescents and Young Adults: Causes, Consequences, and Treatment Strategies. *Pediatrics.* 2005;115(6):1774.

**74.** Wolfson AR, Carskadon MA. Understanding Adolescents' Sleep Patterns and School Performance: A Critical Appraisal. *Sleep Medicine Reiews.* 2003;7(6):491-506.

**75.** Cooper H, Valentine JC, Charlton K, Melson A. The effects of modified school calendars on student academic achievement and on school and community attitudes. *Review of Educational Research.* 2003;73(1):1-52.

**76.** Santelli J, Morreale M, Wigton A, Grason H. School health centers and primary care for adolescents: A review of the literature. *J. Adolesc. Health.* 1996;18:357-366.

**77.** Hedges LV, Laine RD, Greenwald R. An exchange: Part I: Does money matter? A meta-analysis of studies ofthe effects of differential school inputs on student outcomes. *Educational Researcher.* 1994;23(3):5-14.

**78.** Blair D. The Child in the Garden: An Evaluative Review of the Benefits of School Gardening. *The Journal of Environmental Education.* 2009;40(2):15-38.

**79.** Pellegrini AD, Bohn CM. The Role of Recess in Children's Cognitive Performance and School Adjustment. *Educational Researcher.* 2005;34:13-19.

**80.** Podgursky M, Springer MG. Teacher preformance pay: A review. *J. Policy Anal. Manage.* 2007;26(4):909-949.

**81.** Lagarde M, Haines A, Palmer N. Conditional cash transfers for improving uptake of health interventions in low- and middle-income countries: A systematic review. *JAMA.* 2007;298(16):1899-1910.

**82.** Lagarde M, Haines A, Palmer N. The impact of conditional cash transfers on health outcomes and use of health services in low adn middle income countries. *Cochrane Database Syst. Rev.* 2009;4(CD008137).

**83.** Cooper C, Wheeler DM, Woolfenden S, Boss T, Piper S. Specialist home-based nursing services for children with acute and chronic illnesses. *Cochrane Database Syst. Rev.* 2006;4(CD004383).

**84.** Dedman D, Gunnell D, Davey-Smith G, Frankel S. Childhood housing conditions and later mortality in the Boyd Orr cohort. *J. Epidemiol. Community Health.* 2001;55:10-15.

**85.** Shaw M. Housing and public health. *Annual Reviews in Public Health.* 2004;25:379-418.

**86.** Thomson H, Petticrew M, Morrison D. Health effects of housing improvement: Systematic review of intervention studies. *Br. Med. J.* 2001;323:187-190.

**87.** Chanhatasilpa C, MacKenzie DL, Hickman LJ. The effectiveness of community-based programs for chemically dependent offenders: A review and assessment of the research. *J. Subst. Abuse Treat.* 2000;19:383-393.

**88.** Smeslund G, Hagen KB, Steiro A, Johme T, Dalsbo K, Rud G. Work programs for welfare recipients. *Campbell Collaboration Library* 2006.

**89.** Engler-Stringer R, Berenbaum S. Collective kitchens in Canada: A review of the literature. *Candian Journal of Dietetic Practice and Research.* 2005;66(4):246-251.

**90.** Mansuri G, Vijayendra R. Community-based and -driven development: A critical review. *The World Bank Observer.* 2004;19(1):1-39.

**91.** Greenbaum RT, Landers J. Why are state policy-makers still proponents of enterprise zones? What explains their action in the face of a preponderance of the research? *International Regional Science Review.* 2009;32:466-479.

**92.** Donkoh C, Underhill K, Montgomery P. Independent living programmes for improving outcomes for young people leaving the care system. *Campbell Systemic Reviews.* 2006;8.

**93.** Kirby D. The impact of schools and school programs upon adolescent sexual behavior. *The Journal of Sex Research.* 2002;39(1):27-33.

**94.** Koper CS, Mayo-Wilson E. Police Crackdowns on Illegal Gun Carrying: A Systemic Review of Their Impact on Gun Crime. *Journal of Experimental Criminology.* 2006;2:227-261.

**95.** Hahn RA, Bilukha O, Crosby A, et al. Firearm Laws and the Reduction of Violence: A Systemic Review. *Am. J. Prev. Med.* 2005;28(2S1):40-71.

**96.** Powell LM, Chaloupka FJ. Food prices and obesity: Evidence and policy implications for taxes and subsidies. *Milbank Q.* 2009;87(1):229-257.

**97.** Thow AM, Jan S, Leeder S, Swinburn B. The effect of fiscal policy on diet, obesity and chronic disease: A systematic review. *Bull. World Health Organ.* 2010;88:609-614.

**98.** Anderson P, de Bruijn A, Angus K, Gordon R, Hastings G. Impact of alcohol advertising and media exposure on adolescent alcohol use: A systematic review of longitudinal studies. *Alcohol Alcohol.* 2009;44(3):229-243.

**99.** Grube JW. *Preventing alcohol-related problems: Public policy strategies*2005.

**100.** Smith LA, Foxcroft DR. The effect of alcohol advertising, marketing, and portrayal on drinking behavior in young people: Systematic review of prospective cohort studies. *BMC Public Health.* 2009;9.

**101.** Keifer MC. Effectiveness of interventiosn in reducing pesticide overexposure and poisonings. *Am. J. Prev. Med.* 2000;18(4S):80-89.

**102.** Ford PB, Dzewaltowski DA. Disparities in obesity prevalence due to variation in the retail food environment: Three testable hypotheses. *Nutr. Rev.* 2008;66(4):216-228.

**103.** Giskes K, van Lenthe F, Avendano-Pabon M, Brug J. A systematic review of environmental factors and obesogenic dietary intakes among adults: Are we getting closer to undertanding obesogenic environments? *Obes. Rev.* 2010;12:e95-e106.

**104.** Larson NI, Story M, Nelson MC. Neighbhorhood environments: Disparities in access to healthy foods in the U.S. *Am. J. Prev. Med.* 2009;36(1):74-81.

**105.** Stead LF, Lancaster T. Interventions for preventing tobacco sales to minors. *Cochrane Database Syst. Rev.* 2005;1(CD001497).

**106.** Craig EA. Parenting programs for women with mental illness who have young children: A review. *Aust. N. Z. J. Psychiatry.* 2004;38:923-928.

**107.** Mayberry D, Reupert A. Parental mental illness: A review of barriers and issues for working with families and children. *J. Psychiatr. Ment. Health Nurs.* 2009;16:784-791.

**108.** Au W. High-stakes testing and curricular control: A qualitative metasynthesis. *Educational Researcher.* 2007;36:258-268.

**109.** Bulkley K, Fisler J. A decade of charter schools: From theory to practice. *Educational Policy.* 2003;17(3):3170342.

**110.** Miron G, Nelson C. Student academic achievement in charter schools: What we know and why we know so little. *Occasional Paper*. Columbia, NY: National Center for the Study of Privatization in Education, Teacher's College, Columbia University; 2001.

**111.** Bailey T, Karp MM. *Promoting college access and success: a review of credit-based transition programs.* New York: Teachers College, Columbia University, Community College Research Center;2003.

**112.** Coladarci T, Hancock J. Grade-span configuration. *Journal of Research in Rural Education.* 2002;17(3):189-192.

**113.** Wolff KL. The Nonmusical Outcomes of Music Education: A Review of the Literature. *Bulletin of the Council for the Research in Music Education.* 2004;159:74-91.

**114.** Shefer A, Briss P, Rodewald L, et al. Improving immunization coverage rates: A review of the literature. *Epidemiol. Rev.* 1999;21(1):96-142.
