# Supplementary material for: Beyond the clinic: improving child health through evidence-based community development
Source: BMC Pediatr. 2013 Oct 21;13:172. doi: 10.1186/1471-2431-13-172 (PMC4016148; doi:10.1186/1471-2431-13-172)
Supplement: Additional file 2 — Policy briefs for strategies by level of evidence. [file 1471-2431-13-172-S2.docx]

Additional file 1b.  Policy briefs for strategies meeting criteria for efficacy based on a minimum of 2 high-quality trials (Level 1).

Affordable Housing: Tenant-Based Rental Assistance Programs

Domain: Income & Resources

Reasons for Policy:

- The inadequate supply of affordable housing for low-income families is among the most prevalent community health concerns related to family housing^1^
- Lack of affordable housing can result in residential instability, overcrowding, or homelessness^1^
- Nearly one million children in the U.S. experience homelessness each year^2^
- Socioeconomic housing segregation leads to high poverty neighborhoods, which may have serious negative consequences for the well-being and life chances of children^3^
- Tenant-based rental assistance programs give subsidized households expanded choice in where they live^1^
- Because housing mobility policies target very low-income families, they may have the potential to reduce health disparities by improving the health of these disadvantaged groups^4^

Community Group:

- Local government
- Local rental property owners/landlords
- Local housing authorities

Policy Components:

- Tenant-based rental assistance programs which subsidize the cost of housing secured by low-income households within the private rental market through the use of vouchers or direct cash subsidies
- Housing search counseling
- Community networking
- Landlord outreach
- Post-placement services (ex- employment and transportation services)

Desired Outcomes:

- Decreased socioeconomic residential housing segregation
- Decreased exposure to crimes against person and property
- Decreased neighborhood social disorder
- Improved mental and physical health

Level of Evidence Available to Evaluate Effectiveness of Policy (1 = strong evidence to 3 = insufficient evidence*):

1 = Meets Criteria for Effectiveness

Achievable Results:

The following summary of achievable results is based on a published review of the scientific evidence.^5^

- 6%-22% reductions in experience of victimization within the neighborhood^1^
- 3%-89% reductions in neighborhood social disorder, including public drinking and drug use, seeing individuals carrying weapons, and hearing gunfire.^1^

Tenant-based rental assistance programs may also have effects on youth behavioral problems and mental and physical health, with improvement ranging from 4% to 12%.^1^  However, the evidence for these outcomes is insufficient.

Community Examples:

- Seattle Washington passed a 2009 levy that funds rental assistance programs for low income families.
  - <http://www.seattle.gov/housing/levy/TBRA_program_whitepaper.pdf>
  - http://www.cityofseattle.net/housing/levy/docs/Draft_2010-2011_Levy_A&F_Plan.pdf
- Montgomery County Maryland provides rental assistance program to low-income families in need.
  - http://www.montgomerycountymd.gov/hhstmpl.asp?url=/content/hhs/SpecialNeedsHousing/RAPOHEP.asp

Links to Policy Examples†:

- Seattle Washington, City **Ordinance Number:** **123013**
  - http://clerk.ci.seattle.wa.us/~scripts/nph-brs.exe?s1=&s3=&s4=+123013&s2=&s5=&Sect4=AND&l=20&Sect2=THESON&Sect3=PLURON&Sect5=CBORY&Sect6=HITOFF&d=ORDF&p=1&u=%2F%7Epublic%2Fcbory.htm&r=1&f=G
- Montgomery County, Maryland County Code Chapter 41.A § 05.01
  - http://www.amlegal.com/nxt/gateway.dll?f=templates&fn=default.htm&vid=amlegal:montgomeryco_md_mc

*Note: 1 = meets criteria for policy effectiveness (consistent, positive outcomes from at least two high-quality experimental or quasi-experimental trials using a comparison group or interrupted time series design)^5^; 2 = consistent evidence available linking policy with positive outcomes from high-quality observational studies only; 3 = insufficient evidence available for policy or policy components.

† Be sure to check with your state, county, and municipal governments regarding potential existing laws that may impede any new policy development.

References

^1^ Anderson, LM, St. Charles, J, Fullilove, MT, Scrimshaw, SC, Fielding, JE, Normand, J, & the Task Force on Community Preventive Services (2003). Providing affordable family housing and reducing residential segregation by income: A systematic review. American Journal of Preventive Medicine, 24(3S), S47-67.

^2^ The Urban Institute. A new look at homelessness in America. 2000. Available at: [www.urban.org/Template.cfm?Section](http://www.urban.org/Template.cfm?Section)=Home&Nav MenuID=141&template=/TaggedContent/ViewPublication.cfm&PublicationID=7476.

^3^ Ellen, IG & Turner, MA (2003). Do neighborhoods matter and why? : Choosing a better life? Evaluating the moving to opportunity social experiment. (pg 313-338). Washington, DC: Urban Institute Press.

^4^ Acevedo-Garcia, D, Osypuk, TL, & Werbel, RE (2004). Does housing mobility policy improve health? Housing Policy Debate, 15(1), 49-98.

^5^ Flay, BR, Biglan, A, Boruch, RF, Ganzalez Castro, F, Gottfredson, D, Kellam, S, Moscicki, EK, Schinke, S, Valentine, JC, & Ji, P (2005). Standards of evidence: Criteria for efficacy, effectiveness and dissemination. Prevention Science, 6(3), 151-175.

Child Mental Health Programs

Domain: Income & Resources

Reasons for Policy:

- Between 20% and 30% of children have mental health problems.^1^
- Untreated mental disorders can be costly to the children, their families, and society as a whole. Costs are estimated to exceed $14 billion annually.^1, 2^
- Only 25% of children that need mental health services actually get them.^2^

Community Group:

- State government
- Local government
- School district
- Local public health department
- Non-profit organizations

Policy Components:

- Programs focused on early intervention and prevention rather than treating existing problems
- Address multiple domains (family, school, and community)
- Periodic follow-ups
- Uses interactive activities
- Targets at-risk children in early years

Desired Outcomes:

- Lower prevalence rates of anxiety, depression, and conduct disorders
- Higher levels of self-esteem
- Higher levels of pro-social behavior
- Lower rates of dropout, unemployment, and crime due to untreated mental disorders

Level of Evidence Available to Evaluate Effectiveness of Policy (1 = strong evidence to 3 = insufficient evidence*):^3^

1 = Meets Criteria for Effectiveness

Achievable Results:

The following summary of achievable results is based on a published review of the scientific evidence.

On average, prevention programs targeting mental health can achieve:

- Small reductions in conduct disorder symptoms (Effect Size: 0.12-0.39)^2^
- 10% reduction of diagnosed conduct disorder^2^
- 11-17% reductions in diagnostic measures for depression^2^

There is some evidence that these programs can achieve moderate reductions in anxiety among at-risk children. However, more research is needed. ^2^

Community Examples:

- Berkeley, CA
  - <http://www.ci.berkeley.ca.us/ContentDisplay.aspx?id=15654>
- Santa Cruz, CA Health Services Agency provides mental health services through a variety of programs
  - <http://www.santacruzhealth.org/cmhs/2children.htm>

Links to Policy Examples†:

- Berkeley, CA
  - Resolution to increase funding for the mental health workforce
    - <http://www.ci.berkeley.ca.us/citycouncil/2005citycouncil/packet/041905/2005-04-19%20Item%2005.pdf>
- Santa Cruz, CA
  - Chapter 2.104 Establishes a Mental Health Advisory Board which is responsible for the services that the county provides
  - SEE PDF

*Note: 1 = meets criteria for policy effectiveness (consistent, positive outcomes from at least two high-quality experimental or quasi-experimental trials using a comparison group or interrupted time series design)^3^; 2 = consistent evidence available linking policy with positive outcomes from high-quality observational studies only; 3 = insufficient evidence available for policy or policy components.

† Be sure to check with your state, county, and municipal governments regarding potential existing laws that may impede any new policy development.

References

^1^Browne G, Gafni A, Roberts J, Byrne C, Majumdar B (2004). Effective/efficient mental health programs for school-age children: A synthesis of reviews. *Social Science & Medicine,* 58, 1367-1384.

^2^Waddell C, Hua JM, Garland OM, DeV. Peters R, McEwan K (2007). Preventing mental disorders in children: A systematic review to inform policy-making. *Canadian Journal of Public Health,* 98(3), 166-173.

^3^Flay, BR, Biglan, A, Boruch, RF, Ganzalez Castro, F, Gottfredson, D, Kellam, S, Moscicki, EK, Schinke, S, Valentine, JC, & Ji, P (2005). Standards of evidence: Criteria for efficacy, effectiveness and dissemination. Prevention Science, 6(3), 151-175.

Alternatives to Incarceration

Domain: Social Cohesion

Reasons for Policy:

- Incarceration policies and programs have a disproportionate impact on low-income, racial/ethnic minorities.^1^
- Incarceration is expensive and, in many cases, ineffective.^2^
- Incarceration alone cannot satisfy the long-term needs of the criminal justice system^2^

Community Group:

- State/local government
- State/local justice system
- State/local law enforcement
- Community agencies

Policy Components:

- Inclusion of restorative justice policies
- Community service programs
- Use of day reporting centers
- Use of drug courts
- Use of electronic monitoring
- Forfeiture programs
- Home detention programs
- Intensive supervision probation
- Substance abuse treatment for offenders
- Work release programs
- Tailoring each policy component to the community and the offender

Desired Outcomes:

- Lower rates of recidivism
- Easier transition into the community for offenders
- Greater social cohesion
- Improved outcomes for ex-offenders, including more education and job opportunities
- Lower crime rates in the community

Level of Evidence Available to Evaluate Effectiveness of Policy (1 = strong evidence to 3 = insufficient evidence*):^3^

1 = Meets Criteria for Effectiveness

Achievable Results:

The following summary of achievable results is based on a published review of the scientific evidence.

On average, restorative justice programs can achieve:

- Small improvements in victim satisfaction (Effect Size: 0.19, 95% CI: 0.08-0.30)^4^
- Small improvements in offender satisfaction (Effect Size: 0.17)^4^
- Moderate improvements in restitution compliance (Effect Size: 0.33)^4^
- Small improvements in recidivism (Effect Size: 0.07, 95% CI: 0.02-0.12)^4^

On average, drug courts can achieve:

- Reductions in costs related to prison or jail sentences^2^
- Reductions in re-arrests of drug offenders^2^

There is limited evidence available for community service/work crew programs, day reporting centers, electronic monitoring, forfeiture programs, home detention, intensive supervision probation, and work release programs. Initial work suggests these may have positive effects, however more research is needed.

Community Examples:

- Michigan Department of Corrections reduced its prison population by adopting the Michigan Prisoner Re-Entry Initiative (MPRI)
  - <http://www.michigan.gov/corrections/0,1607,7-119-9741_33218---,00.html>
  - <http://www.michpri.com/>
- El Paso, Texas, West Texas Community Supervision and Corrections Department, provides probation and community corrections services which include; residential programs, rehabilitative programs, drug court, and Re-Entry court.
  - <http://www.co.el-paso.tx.us/wtc/>

Links to Policy Examples:

- Michigan House Bill No. 4538 amends MCL, §791.234a (12) placement in special alternative incarceration unit
  - See pdf
- Texas Statutes, §76.017 Treatment Alternative to Incarceration Program
  - See pdf

*Note: 1 = meets criteria for policy effectiveness (consistent, positive outcomes from at least two high-quality experimental or quasi-experimental trials using a comparison group or interrupted time series design)^3^; 2 = consistent evidence available linking policy with positive outcomes from high-quality observational studies only; 3 = insufficient evidence available for policy or policy components.

† Be sure to check with your state, county, and municipal governments regarding potential existing laws that may impede any new policy development.

‡Local governments and organizations may check existing state statutes and administrative codes for the authority to implement local policies.

References

^1^Freudenberg, N (2001). Jails, prisons, and the health of urban populations: A review of the impact of the correctional system on community health. *Journal of Urban Health: Bulletin of the New York Academy of Medicine,* 78(2), 214-235.

^2^Patchin JW, & Keveles GN (2004). Alternatives to incarceration: An evidence-based research review: A summary of finding. *Northwest Wisconsin Criminal Justice Management Conference,* Lakewoods Resort, Cable, Wisconsin.

^3^Flay, BR, Biglan, A, Boruch, RF, Ganzalez Castro, F, Gottfredson, D, Kellam, S, Moscicki, EK, Schinke, S, Valentine, JC, & Ji, P (2005). Standards of evidence: Criteria for efficacy, effectiveness and dissemination. Prevention Science, 6(3), 151-175.

^4^Latimer J, Dowden C, & Muise D (2005). The effectiveness of restorative justice practices: A meta-analysis. *The Prison Journal,* 85(2), 127-144.

Community-based Participatory Research

Domain: Social cohesion

Reasons for Policy:

- Researchers and practitioners have called for greater community involvement and control through partnerships among academic, health practice and community organizations.^1^
- Community–based participatory research (CBPR) enhances the relevance, usefulness, and use of research data by all partners involved.^1^
- Knowledge gained through research should be used by all partners involved to direct resources and influence policies that will benefit the community.^1^
- CBPR is an approach to health and environmental research meant to increase the value of studies that address the problems of health care disparities in a variety of populations.^2^

Community Group:

- Universities and colleges
- Government agencies
- Community organizations

Policy Components:

- Active involvement of community members, organizational representatives, and researchers in all aspects of the research process.
- Emphasis on the participation and influence of nonacademic researchers in the process of creating knowledge.
- Partnerships among representatives from health and human service organizations, academia, community-based organizations, and the community-at-large.

Desired Outcomes:

- Improved research quality outcomes
- Improved behavioral and physiological health outcomes
- Improved community capacity for development
- Better informed and more effective practice

Level of Evidence Available to Evaluate Effectiveness of Policy (1 = strong evidence to 3 = insufficient evidence*):^3^

1 = Meets Criteria for Effectiveness

Achievable Results:

The following summary of achievable results is based on a published review of the scientific evidence.

On average, community-based participatory research can achieve:

- Improved community capacity, including additional grant funding obtained by the community and job creation due to the collaboration.^2^
- Enhanced intervention quality related to community development.^2^

Note: There is insufficient evidence regarding CBPR’s effect on health outcomes. Further research is needed to assess the impact of CBPR on health outcomes.^2^

Community Examples:

- Atlanta, Georgia, Morehouse School of Medicine, Prevention Research Center engages in interdisciplinary applied prevention research in collaboration with community partners, government agencies and universities.
  - <http://www.msm.edu/research/research_centersandinstitutes/research_cni_PRC/overview.aspx>
  - Prevention research programs: <http://www.msm.edu/research/research_centersandinstitutes/research_cni_PRC/preventionPrograms.aspx>
- Trenton, New Jersey, Isles, Inc. a non-profit community development organization, has adopted a community based approach to conducting research.
  - <http://www.isles.org/>
- Seattle, Washington, University of Washington, School of Public Health conducts community based research.
  - <http://sph.washington.edu/research/>

Links to Policy Examples:

- Morehouse School of Medicine, Prevention Research Center, Bylaws.
  - <http://depts.washington.edu/ccph/pdf_files/bylaws%2002.99.pdf>
- Isles, Inc., Isles Research Principles
  - <http://depts.washington.edu/ccph/pdf_files/Isles%20Research%20Principles%202003.pdf>
- University of Washington, School of Public Health Community-Based Research Principles
  - <http://sph.washington.edu/research/community.asp>

*Note: 1 = meets criteria for policy effectiveness (consistent, positive outcomes from at least two high-quality experimental or quasi-experimental trials using a comparison group or interrupted time series design)^3^; 2 = consistent evidence available linking policy with positive outcomes from high-quality observational studies only; 3 = insufficient evidence available for policy or policy components.

† Be sure to check with your state, county, and municipal governments regarding potential existing laws that may impede any new policy development.

References

1 Israel, BA, Schulz, AJ, Parker, EA, Becker, AB (1998). Review of Community-Based Research: Assessing Partnership Approaches to Improve Public Health. *Annual Review of Public Health*, 19, 173-202.

2 Viswanathan, M, et al. (2004). Community-based Participatory Research: Assessing the Evidence. Evidence Report/Technology Assessment No. 99. Agency for Healthcare Research and Quality, AHRQ Publication 04-E022-2.

3Flay, BR, Biglan, A, Boruch, RF, Ganzalez Castro, F, Gottfredson, D, Kellam, S, Moscicki, EK, Schinke, S, Valentine, JC, & Ji, P (2005). Standards of evidence: Criteria for efficacy, effectiveness and dissemination. Prevention Science, 6(3), 151-175.

Mentoring Programs

Domain: Social cohesion

Reasons for Policy:

- The relationship between a mentor and mentee can help foster healthy relationships.^1^
- Mentors can provide support, advocacy, advice, and a positive model to at-risk youth.^1^
- Studies have found a positive effect on youth in mentorship programs.^1^

Community Group:

- Local government
- Local school board
- Local justice system
- Local non-profit organizations

Policy Components:

- Pairing of a responsible, caring adult with an at-risk youth
- Regular meeting of the mentor and mentee

Desired Outcomes:

- Positive mentor/mentee relationship
- Positive role models for at-risk youth
- Fewer occurrences of problem or delinquent behavior
- Greater academic achievement
- Lower levels of aggression
- Less drug use

Level of Evidence Available to Evaluate Effectiveness of Policy (1 = strong evidence to 3 = insufficient evidence*):^2^

1 = Meets Criteria for Effectiveness

Achievable Results:

The following summary of achievable results is based on a published review of the scientific evidence.

On average, mentoring programs can achieve:

- Small reductions in delinquency (Effect Size: -0.25)^1^
- Moderate reductions in aggression (Effect Size: -0.40)^1^
- Small improvements in academic achievement (Effect Size: 0.14)^1^
- Small reductions in drug use have been observed (Effect Size: -0.13). However, these reductions were not statistically significant.^1^

Community Examples:

- The Dubuque Community School District (Iowa) created a mentoring program to enrich the lives an education of their students.
  - http://www.dubuque.k12.ia.us/studentmentoring/Studentmentoringinformation.htm
- The DeKalb County School System (Georgia) offers various mentoring programs to improve student achievement
  - http://www.dekalb.k12.ga.us/support/counseling/mentoring.html

Links to Policy Examples: †

- The Dubuque Community School District: Chapter 4 §4614 and Chapter 6 § 6203
  - http://www.dubuque.k12.ia.us/schoolboard/policies/TOCTable06-18-04.htm
- The DeKalb County School System: Administrative Regulation
  - https://eboard.eboardsolutions.com/ePolicy/policy.aspx?PC=IG-R&Sch=4054&S=4054&RevNo=1.01&C=I&Z=R

*Note: 1 = meets criteria for policy effectiveness (consistent, positive outcomes from at least two high-quality experimental or quasi-experimental trials using a comparison group or interrupted time series design)^2^; 2 = consistent evidence available linking policy with positive outcomes from high-quality observational studies only; 3 = insufficient evidence available for policy or policy components.

† Be sure to check with your state, county, and municipal governments regarding potential existing laws that may impede any new policy development.

References

^1^Tolan P, Henry D, Schoeny M, Bass A. Mentoring interventions to affect juvenile delinquency and associated problems. Campbell Systematic Reviews 2008:16

^2^ Flay, BR, Biglan, A, Boruch, RF, Ganzalez Castro, F, Gottfredson, D, Kellam, S, Moscicki, EK, Schinke, S, Valentine, JC, & Ji, P (2005). Standards of evidence: Criteria for efficacy, effectiveness and dissemination. Prevention Science, 6(3), 151-175.

Neighborhood Watch Programs

Domain: Social cohesion

Reasons for Policy:

- Neighborhood watch can deter offenders if they know local residents are likely to report suspicious activity.^1^
- Neighborhood watch may also reduce criminal opportunities, such as creating signs of occupancy when neighbors are away from home.^1^

Community Group:

- Local government
- Local law enforcement
- Local community organizations

Policy Components:

- Organized block watches, usually run by a block captain
- Use of property-marking and home security surveys
- Use of a neighborhood liaison to the local police department

Desired Outcomes:

- Reduction in neighborhood crime
- Greater social cohesion
- Reduction in the opportunity for crime to occur
- Improved relationship with local law enforcement

Level of Evidence Available to Evaluate Effectiveness of Policy (1 = strong evidence to 3 = insufficient evidence*):^2^

1 = Meets Criteria for Effectiveness

Achievable Results:

The following summary of achievable results is based on a published review of the scientific evidence.

On average, neighborhood watch programs can achieve:

- 16-26% reductions in crime^1^

Community Stories:

- Wake Village, Texas has a blog used to communicate with members of the local community about events and other activities
  - http://wakevillagewatchgroup.blogspot.com/Community example
- Palm Springs, California Police Department has a Neighborhood Watch program
  - <http://www.pspd.com/crimeprevention.html>

Policy Examples: †

- Wake Village, Texas Code of Ordinances, Title 3 Chapter 32.15
  - See pdf file
- Palm Springs, California Ordinance 1666. Section 2.55.130 Establishes a Neighborhood Empowerment Program in which Neighborhood Watch programs are included
  - <http://www.themoviecolony.org/docs/Ordinance1666.pdf>

*Note: 1 = meets criteria for policy effectiveness (consistent, positive outcomes from at least two high-quality experimental or quasi-experimental trials using a comparison group or interrupted time series design)^2^; 2 = consistent evidence available linking policy with positive outcomes from high-quality observational studies only; 3 = insufficient evidence available for policy or policy components.

† Be sure to check with your state, county, and municipal governments regarding potential existing laws that may impede any new policy development.

References

^1^Bennett Trevor, Holloway Katy, and Farrington David. (2005). “The Effectiveness of Neighborhood Watch”. In: The Campbell Collaboration Reviews of Intervention and Policy Evaluations (C2-RIPE).

^2^Flay, BR, Biglan, A, Boruch, RF, Ganzalez Castro, F, Gottfredson, D, Kellam, S, Moscicki, EK, Schinke, S, Valentine, JC, & Ji, P (2005). Standards of evidence: Criteria for efficacy, effectiveness and dissemination. Prevention Science, 6(3), 151-175.

Organizational Changes to the Psychosocial Work Environment

Domain: Social Cohesion

Reasons for Policy:

- The work environment is an important determinant of health and health inequalities^1^
- The “demand control (support)” model hypothesizes that physical and mental health is negatively associated with job demands and positively associated with control and social support in the workplace^1^

Community Group:

- Local government
- Local public health department
- Local non-profit organizations
- Local business organizations

Policy Components:

- Increase employee control by including employees in decision making for the workplace
- Changes to shift work:
  - Compressed work weeks (4 days w/12 hour shifts instead of 5 days w/8 hour shifts)
  - Switching from slow to fast shift rotation (max of 3-4 consecutive shifts of the same type)
  - Changing from backward to forward shift rotation (morning, afternoon, night rather than night afternoon, morning)
  - Self-scheduling shifts
- Government management of public agencies and industries
- Increased regulation and inspections for employee safety legislation

Desired Outcomes:

- Improved social support in workplace
- Improved physical and mental health of employees
- Healthier and safer work environments
- Improved employee satisfaction and productivity
- Reduced health inequalities amongst workers
- Improved work-life balance

Level of Evidence Available to Evaluate Effectiveness of Policy (1 = strong evidence to 3 = insufficient evidence*)^2^:

1 = Meets Criteria for Effectiveness

Achievable Results:

The following summary of achievable results is based on a published review of the scientific evidence.

On average, increasing employee control can achieve:

- Mixed, but generally positive effects on employee health^1^

On average, changes to shift work can achieve:

- Positive impacts on work-life balance^1^
- Positive effects on mental health^1^

On average, government management of public agencies and industries can achieve:

- Increase in job security^1^
- Decrease in job stress^1^

On average, greater regulation and inspection of workplace safety can achieve:

- Decrease in injury rates^1^

Community Examples:

- Santa Barbara, California allows city employees to participate in alternative work schedules
  - See pdf
- Lake County, Florida city government has an extensive program to ensure the safety of their workforce <http://www.lakecountyfl.gov/departments/employee_services/health/wellness_safety.aspx#sect_safety>

Links to Policy Examples†:

- Santa Barbara, California city council agenda detailing the aspects of the alternative work schedule <http://www.santabarbaraca.gov/NR/rdonlyres/CD6AEB3A-06A2-47C1-A54F-6CCC1988630D/0/20070515Citywide980.pdf>
- Lake County, Florida policy detailing the elements of the safety program <http://www.lakecountyfl.gov/documents/employee_services/policies_and_procedures/safety_policy_LCC68.pdf>

*Note: 1 = meets criteria for policy effectiveness (consistent, positive outcomes from at least two high-quality experimental or quasi-experimental trials using a comparison group or interrupted time series design)^2^; 2 = consistent evidence available linking policy with positive outcomes from high-quality observational studies only; 3 = insufficient evidence available for policy or policy components.

† Be sure to check with your state, county, and municipal governments regarding potential existing laws that may impede any new policy development.

References

^1^Bambra C, Gibson M, Sowden AJ, Wright K, Whitehead M, Petticrew M (2009). Working for health? Evidence from systematic reviews on the effects on health and health inequalities of organizational changes to the psychosocial work environment. *Preventive Medicine,* 48, 454-461.

^2^Flay, BR, Biglan, A, Boruch, RF, Ganzalez Castro, F, Gottfredson, D, Kellam, S, Moscicki, EK, Schinke, S, Valentine, JC, & Ji, P (2005). Standards of evidence: Criteria for efficacy, effectiveness and dissemination. Prevention Science, 6(3), 151-175.

Prisoner Re-entry

Domain: Social cohesion

Reasons for Policy:

- Failure to reintegrate ex-offenders can lead to many negative effects for the individual, families, and communities.^1^
- Every year, over half a million inmates are released from prison, many of which are ill-prepared to reenter their communities.^1,2^
- Incarceration policies and programs have a disproportionate impact on low-income communities due to disproportionate incarceration of low-income, racial/ethnic minorities.^1^

Community Group:

- Local government
- Local justice and correctional departments
- Local law enforcement
- Local non-profit organizations
- Local faith-based organizations

Policy Components:

- Vocational training and work release programs
- Drug rehabilitation while incarcerated
- Half-way house programs
- Prerelease programs

Desired Outcomes:

- Reduced recidivism
- Improved job placement
- Improved community cohesion
- Easier transition into community life
- Lower rates of drug use and risky sexual behavior

Level of Evidence Available to Evaluate Effectiveness of Policy (1 = strong evidence to 3 = insufficient evidence*):^3^

1 = Meets Criteria for Effectiveness

Achievable Results:

The following summary of achievable results is based on a published review of the scientific evidence.

On average, vocational training and work release programs can achieve:

- Reduction in recidivism^2^
- Improved job readiness^2^

On average, drug rehabilitation while incarcerated and half-way houses can:

- Ease the transition from prison to the community^2^

On average, prerelease programs can achieve:

- Reduced recidivism^2^

Community Examples:

- Philadelphia, Pennsylvania created the prisoner re-entry employment program (PREP) to encourage business owners to hire former inmates in order to reduce recidivism rates. PREP guide is attached pdf
  - <http://www.phila.gov/reentry/>
  - See pdf
- Tarrant County, Texas created a prisoner reentry program to aid prisoners in re-integrating into the local community.
  - http://www.tarrantcountytx.gov/etcri/site/default.asp

Links to Policy Examples: †

- Philadelphia, PA Title 20, Chapter 20-1700
  - See pdf
- Tarrant County, TX Reentry Initiative Charter
  - http://www.tarrantcountytx.gov/etcri/lib/etcri/council_charter_%282%29.pdf

*Note: 1 = meets criteria for policy effectiveness (consistent, positive outcomes from at least two high-quality experimental or quasi-experimental trials using a comparison group or interrupted time series design)^3^; 2 = consistent evidence available linking policy with positive outcomes from high-quality observational studies only; 3 = insufficient evidence available for policy or policy components.

† Be sure to check with your state, county, and municipal governments regarding potential existing laws that may impede any new policy development.

References

^1^Freudenberg, N (2001). Jails, prisons, and the health of urban populations: A review of the impact of the correctional system on community health. *Journal of Urban Health: Bulletin of the New York Academy of Medicine,* 78(2), 214-235.

^2^Seiter, RP, Kadela, KR (2003). Prisoner reentry: What works, what does not, and what is promising. *Crime Delinquency,* 49(3), 360-388.

^3^Flay, BR, Biglan, A, Boruch, RF, Ganzalez Castro, F, Gottfredson, D, Kellam, S, Moscicki, EK, Schinke, S, Valentine, JC, & Ji, P (2005). Standards of evidence: Criteria for efficacy, effectiveness and dissemination. Prevention Science, 6(3), 151-175.

Access to Places for Physical Activity

Domain: Physical environment

Reasons for Policy:

- Regular physical activity is associated with enhanced health and reduced risk of all-cause mortality.^1^
- Despite the benefits of regular physical activity, only 25% of adults in the United States report engaging in the recommended amounts of physical activity.^2^
- Low-income and racial/ethnic minority populations have less access to facilities for physical activity, which may contribute to disparities in regular physical activity and obesity.^3^

Community Group:

- Worksites, coalitions, agencies, and communities
- Parks and Recreation Departments
- Local Government
- Gyms and Fitness Facilities

Policy Components:

- Improve access to existing facilities or create new places (e.g., walking trails)
- Informational outreach activities, such as training on equipment, health behavior education and techniques, seminars, counseling, risk screening, health forums, and workshops

Desired Outcomes:

- Create healthful physical and organizational environments
- Increase physical activity in the community
- Increase physical fitness
- Reduce body fat or weight in individuals

Level of Evidence Available to Evaluate Effectiveness of Policy (1 = strong evidence to 3 = insufficient evidence*):^4^

1 = Meets Criteria for Effectiveness

Achievable Results:

The following summary of achievable results is based on a published review of the scientific evidence.

On average, increasing access to places for physical activity can achieve:

- 5.1% increase in aerobic capacity^5^
- 8.2% increase in energy expenditure^5^
- 2.9% increase in leisure-time physical activity^5^
- 48.4% increase in frequency of physical activity^5^

Community Examples:

- Utah, the Governor of Utah implemented an initiative, WorkWell, which encourages public agencies to provide a healthy workplace. Healthy Utah, a free program for state and local government employees and their spouses, offers wellness programs, wellness councils, seminars and webinars. Forty-three state agency sites have implemented worksite wellness councils.
  - <http://www.healthyutah.org/workwell/>
  - <http://www.healthyutah.org/>
- Ohio, Healthy Ohioans Business Council is a group of Ohio companies promoting best practices for a healthy workforce. The Governor appoints members to the executive committee from companies providing exemplary health and wellness programs for their employees. The Council, working with the Ohio Department of Health, promotes health and wellness programs and encourages Ohio business to adopt wellness practices.
  - <http://healthyohioprogram.org/healthpromotion/business/businesses.aspx>
  - <http://www.healthyohioans.org/businesses/winwin.aspx>
- Boston, Healthworks Foundation, a non-profit organization, operates fitness centers for low-income women.
  - <http://www.healthworksfitness.com/>
  - <http://www.healthworksfoundation.org/>

Links to Policy Examples:

- The Governor and Director of the Utah Department of Health, through their administrative powers, encourage and implement the Work Well and Healthy Utah programs.
  - <http://www.healthyutah.org/downloads/ww/WorkWell_Memo.pdf>
  - <http://www.healthyutah.org/downloads/Governors_Recommendations.pdf>
- Healthy Ohio Business Council, Articles of Operation
  - See pdf

*Note: 1 = meets criteria for policy effectiveness (consistent, positive outcomes from at least two high-quality experimental or quasi-experimental trials using a comparison group or interrupted time series design)^4^; 2 = consistent evidence available linking policy with positive outcomes from high-quality observational studies only; 3 = insufficient evidence available for policy or policy components.

† Be sure to check with your state, county, and municipal governments regarding potential existing laws that may impede any new policy development.

References

^1^ U.S. Department of Health and Human Services, Physical activity and health: A report of the Surgeon General (1996). U.S. Department of Health and Human Services, Centers for Disease Control and Prevention, National Center for Chronic disease Prevention and Health Promotion, 1996.

^2^ U.S. Department of Health and Human Services, Public Health Service, Centers for Disease Control and Prevention, National Center for Chronic Disease Prevention and Health Promotion, Division of Nutrition and Physical Activity (1999). Promoting physical activity; A guide to community action. Champaign, IL: Human Kinetics, 1999.

^3^ Gordon-Larsen, P, Nelson, MC, Page, P, & Popkin, BM (2006). Inequality in the built environment underlies key health disparities in physical activity and obesity. Pediatrics, 117(2), 417-424.

^4^ Flay, BR, Biglan, A, Boruch, RF, Ganzalez Castro, F, Gottfredson, D, Kellam, S, Moscicki, EK, Schinke, S, Valentine, JC, & Ji, P (2005). Standards of evidence: Criteria for efficacy, effectiveness and dissemination. Prevention Science, 6(3), 151-175.

^5^ Kahn, EB, Ramsey, LT, Brownson, RC, Heath, GW, Howze, EH, Powell, KE, Stone, EJ, Rajab, MW, Corso, P, & The Task Force on Community Preventive Services (2002). The effectiveness of interventions to increase physical activity: A systematic review. American Journal of Preventive Medicine, 22(4S), 73-107.

Alcoholic Beverage Excise Taxes

Domain: Physical environment

Reasons for Policy:

- Heavy alcohol consumption contributes to approximately 79,000 deaths per year in the United States.^1^
- Alcohol is the 3^rd^ leading cause of preventable death in the United States.^1^
- Alcohol consumption is a risk factor for injuries, diseases, and social problems.^2^

Community Group:

- Local government
- Local alcohol distributors
- Local alcohol retailers

Policy Components:

- Increase in excise taxes on alcoholic drinks (e.g. beer, wine, and spirits)

Desired Outcomes:

- Reduce alcohol consumption
- Reduce alcohol-related morbidity and mortality
- Reduce alcohol-related unintentional injuries

Level of Evidence Available to Evaluate Effectiveness of Policy (1 = strong evidence to 3 = insufficient evidence*):^3^

1 = Meets Criteria for Effectiveness

Achievable Results:

The following summary of achievable results is based on a published review of the scientific evidence.

Doubling the tax on alcoholic beverages would be associated with a(n):

- 35% reduction in alcohol-related morbidity and mortality^2^
- 11% reduction in traffic crash deaths^2^
- 6% reduction in sexually transmitted disease^2^
- 2% reduction in violence^2^
- 1% reduction in crime^2^
- 6% reduction in general alcohol consumption^4^
- 17% reduction in beer consumption^4^
- 25% reduction in wine consumption^4^
- 25% reduction in distilled spirits consumption^4^
- 1% reduction in consumption among heavy drinkers^4^

Community Examples:

- Dawson County (Georgia) Planning and Development Department implemented an excise tax on all distilled beverages and wine sold in the county.
  - http://www.dawsoncounty.org/page.php?id=239
- Cook County (Illinois) Department of Revenue implemented a tax on the retail sale of all alcoholic beverages within the county
  - <http://www.cookcountygov.com/portal/server.pt?open=514&objID=452&parentname=CommunityPage&parentid=1&mode=2&in_hi_userid=2&cached=true>

Links to Policy Examples**†**:

- Consolidated Alcohol Ordinance of Dawson County Article III, Section 300
  - See Attached PDF
- Cook County, IL Alcohol Beverage Tax Ordinance Chapter 74 Article IX
  - <http://www.cookcountygov.com/Agencies/CC_LIQUOR_ORD.pdf>

*Note: 1 = meets criteria for policy effectiveness (consistent, positive outcomes from at least two high-quality experimental or quasi-experimental trials using a comparison group or interrupted time series design)^3^; 2 = consistent evidence available linking policy with positive outcomes from high-quality observational studies only; 3 = insufficient evidence available for policy or policy components.

† Be sure to check with your state, county, and municipal governments regarding potential existing laws that may impede any new policy development.

References

^1^Campbell CA, et al. (2009). The effectiveness of limiting alcohol outlet density as a means of reducing excessive alcohol consumption and alcohol-related harms. *American Journal of Preventive Medicine,* 37(6). 556-569.

^2^Wagenaar AC, Tobler AL, Komro KA (in press). Effects of alcohol tax and price policies on morbidity and mortality: A systematic review. *American Journal of Public Health.*

^3^Flay, BR, Biglan, A, Boruch, RF, Ganzalez Castro, F, Gottfredson, D, Kellam, S, Moscicki, EK, Schinke, S, Valentine, JC, & Ji, P (2005). Standards of evidence: Criteria for efficacy, effectiveness and dissemination. Prevention Science, 6(3), 151-175.

^4^Wagenaar AC, Salois MJ, Komro KA (2009). Effects of beverage alcohol price and tax levels on drinking: A meta-analysis of 1,003 estimates from 112 studies. *Addiction,* 104, 179-190.

Alcohol Outlet Density

Domain: Physical environment

Reasons for Policy:

- Alcohol is ranked 3^rd^ out of 26 risk factors that contribute to disease, disability, or mortality^1^
- Approximately 79,000 deaths per year in the United States are due to binge drinking or heavy daily drinking^2^
- In 2009, approximately 29% of adult drinkers in the U.S. reported binge drinking in the last 30 days^2^
- In 2009, 67% of high school students who drank reported binge drinking in the last 30 days^2^

Community Group:

- Local government
- Local businesses
- Local department that issues liquor licenses (different depending on state)
- Local zoning boards

Policy Components:

- Restrict the number of alcohol licenses allowed in a particular area
- Restrict the types of locations that are allowed to sell alcohol (e.g. grocery stores)

Desired Outcomes:

- Lower rates of problem drinking behaviors
- Lower rates of violence due to drinking
- Lower rates of injuries due to drinking
- Lower rates of alcohol consumption

Level of Evidence Available to Evaluate Effectiveness of Policy (1 = strong evidence to 3 = insufficient evidence*):^3^

1 = Meets Criteria for Effectiveness

Achievable Results:

The following summary of achievable results is based on a published review of the scientific evidence.

Reducing on- and off-premise alcohol outlet density can achieve:

- Large, significant reductions in alcohol consumption (mean elasticity: 0.27)^2^
- Large. significant reductions in interpersonal violence (mean elasticity: 0.32)^2^

There is also consistent evidence from observational studies that reducing on- and off-premise alcohol outlet density can achieve:

- Large reductions in unintentional injury (mean elasticity: 0.23)^2^
- Small reductions in crime (mean elasticity: 0.04)^2^

Community Examples: †

- Madison, Wisconsin created an Alcohol License Review Committee to lower the amount of retail alcohol outlets within the city.
  - <http://www.cityofmadison.com/mcc12/alrc.html>
  - <http://www.cityofmadison.com/CityHall/alcoholPolicies/documents/DensityPlan.pdf>
- The Oakland City Attorney (California) created a position to enforce the problems with areas that are highly concentrated with liquor outlets.
  - <http://www.oaklandcityattorney.org/Community/LiquorStores.html>
  - <http://www.oaklandnet.com/government/ceda/revised/planningzoning/Commission/November-18-09/CityAttorneysReport/CityAttReport_x-CEDA-Daria.pdf>

Links to Policy Examples:

- Madison, WI municipal code Chapter 38, Section 05
  - See attached pdf file (page 29, section (o))
- Oakland, CA City CodeTitle 17, Chapter 156 improves operating standards of non-conforming alcohol outlets and implements an enforcement program to monitor the operators.
  - see attached pdf

*Note: 1 = meets criteria for policy effectiveness (consistent, positive outcomes from at least two high-quality experimental or quasi-experimental trials using a comparison group or interrupted time series design)^3^; 2 = consistent evidence available linking policy with positive outcomes from high-quality observational studies only; 3 = insufficient evidence available for policy or policy components.

† Be sure to check with your state, county, and municipal governments regarding potential existing laws that may impede any new policy development.

References

^1^Popova S, Giesbrecht N, Bekmuradov D, & Patra J (2009). Hours and days of sale and density of alcohol outlets: Impacts on alcohol consumption and damage: A systematic review. *Alcohol & Alcoholism,* 44(5), 500-516.

^2^Campbell CA, et al. (2009). The effectiveness of limiting alcohol outlet density as a means of reducing excessive alcohol consumption and alcohol-related harms. *American Journal of Preventive Medicine,* 37(6). 556-569.

^3^Flay, BR, Biglan, A, Boruch, RF, Ganzalez Castro, F, Gottfredson, D, Kellam, S, Moscicki, EK, Schinke, S, Valentine, JC, & Ji, P (2005). Standards of evidence: Criteria for efficacy, effectiveness and dissemination. Prevention Science, 6(3), 151-175.

Bicycle Helmet Use

Domain: Physical Environment

Reasons for Policy:

- Bicycle-related head injuries and deaths are common and can be prevented, or reduced in severity, with helmet use.^1^
- Only 25% of the general population of bicyclists in America use helmets regularly.^2^
- Helmet legislation increases bicycle helmet use and decreases head injuries among all age groups.^3^

Community Group:

- Local or State Government Legislation
- Law Enforcement

Policy Components:

- Legislation requiring bicycle helmet use among all cyclists under 16 years old
- Enforcement of bicycle helmet laws
- Distribution of bicycle helmets (or incentives to purchase)
- Education

Desired Outcomes:

- Increase bicycle helmet use
- Decrease bicycle-related deaths
- Decrease head injury severity and occurrence

Level of Evidence Available to Evaluate Effectiveness of Policy (1= strong evidence to 3=insufficient evidence*):

- 1= Meets Criteria for Effectiveness

Achievable Results:

The following summary of achievable results is based on a published review of the scientific evidence.^4^

| Outcome | % Change | Direction |
| --- | --- | --- |
| Bicycle helmet use^3^ | 79.0% | Increase |
| Bicycle-related head injuries^1^ | 65%-88% | Decrease |

Community Examples:

- Houston Texas requires all children under 18 to wear a protective helmet when riding as a passenger or operator of a bicycle.
- St. Louis County Missouri requires anyone aged 1-17 to wear a protective helmet when riding as a passenger or operator of a bicycle.

Links to Policy Examples:†

- Houston Texas x, ARTICLE XII , § 45-326
  - http://library7.municode.com/default-test/home.htm?infobase=10123&doc_action=whatsnew
- St. Louis County, Missouri Title VI, § 602.600
  - http://library3.municode.com/default-test/home.htm?infobase=11512&doc_action=whatsnew

*Note: 1 = meets criteria for policy effectiveness (consistent, positive outcomes from at least two high-quality experimental or quasi-experimental trials using a comparison group or interrupted time series design)4; 2 = consistent evidence available linking policy with positive outcomes from high-quality observational studies only; 3 = insufficient evidence available for policy or policy components.

† Be sure to check with your state, county, and municipal governments regarding potential existing laws that may impede any new policy development.

References:

^1^ Thompson, D, Rivara, F, & Thompson, R (1999). Helmets for preventing head and facial injuries in bicyclists. Cochrane Database of Systematic Reviews, 1999, Issue 4.

^2^ Macpherson, A & Spinks, A (2008). Bicycle helmet legislation for the uptake of helmet use and prevention of head injuries. The Cochrane Database of Systematic Reviews, 2008, Issue 3.

^3^  Karkhaneh, M, Kalenga, J-C, Hagel, BE, & Rowe BH, (2006). Effectiveness of bicycle helmet legislation to increase helmet use: a systematic review. Injury Prevention, 12:76-82.

^4^ Flay, BR, Biglan, A, Boruch, RF, Ganzalez Castro, F, Gottfredson, D, Kellam, S, Moscicki, EK, Schinke, S, Valentine, JC, & Ji, P (2005). Standards of evidence: Criteria for efficacy, effectiveness and dissemination. Prevention Science, 6(3), 151-175.

Booster Seat Use

Domain: Physical Environment

Reasons for Policy:

- Motor vehicle injuries are the leading cause of death among children in the U.S.^1^
- During 2008, 968 children ages 14 years and younger died as occupants in motor vehicle crashes, and approximately 168,000 were injured.^1^
- Booster seat use among children aged four to eight years significantly lowers the risk of injuries.^2^
- Policies are effective in increasing the use of booster seat use among children.^3^
- Child safety restraint use is lower in low-income communities due to lack of access to affordable safety seats.^4^

Community Group:

- Local Government
- Local Public Health Department
- Law Enforcement

Policy Components:

- Legislation requiring booster seat use
- Enforcement of booster seat laws
- Distribution of booster seats (or incentives to purchase)
- Education

Desired Outcomes:

- Increase booster seat ownership
- Increase booster seat use
- Decrease motor vehicle crash injuries among children
- Decrease motor vehicle crash deaths among children

Level of Evidence Available to Evaluate Effectiveness of Policy:

1= Meets criteria for effectiveness

Achievable Results:

The following summary of achievable results is based on a published review of the scientific evidence.^5^

On average, any booster seat intervention can achieve:

- 100% increase in booster seat use^3^

On average, an educational intervention can achieve:

- 30% increase in ownership or use of booster seats^3^

On average, distribution of booster seats (or incentives to purchase) and education can achieve:

- 130%-175% increase in ownership or use of booster seats^3^

There is not enough evidence to evaluate the effectiveness of legislation or enforcement efforts on booster seat ownership or use.^3^

Community Examples:

- Drive Smart Colorado Springs, CO is a program to promote booster seat utilization.
  - <http://www.drivesmartcoloradosprings.com/BoosterSeat/AProjectSummary.doc>
  - <http://www.drivesmartcoloradosprings.com/BuckleBear.aspx>
- Bexley, Ohio
  - <http://www.odh.ohio.gov/ASSETS/9DBD6CE02B1D4106BCA3C327C4E39AD5/boostbroch.pdf>
  - <http://www.odh.ohio.gov/odhPrograms/hprr/cpsafe/cpsafety.aspx>

Links to Policy Examples†:

- Bexley, OH, Ordinance No. 64-09, amends city code 438.26, section 4511.01
  - <http://www.bexley.org/govt/public-docs/doc_download/1047-2009-ordinance-64-09>
- Colorado Springs, CO Chapter 10, Article 23, Ordinance 10.23.117
  - <http://www.sterlingcodifiers.com/CO/Colorado%20Springs/>

Note: 1 = meets criteria for policy effectiveness (consistent, positive outcomes from at least two high-quality experimental or quasi-experimental trials using a comparison group or interrupted time series design) ^4^; 2 = consistent evidence available linking policy with positive outcomes from high-quality observational studies only; 3 = insufficient evidence available for policy or policy components.

† Be sure to check with your state, county, and municipal governments regarding potential existing laws that may impede any new policy development.

References

^1^ Centers for Disease Control and Prevention (2009). Child Passenger Safety: Fact Sheet. Available from URL: [www.cdc.gov/MotorVehicleSafety/Child_Passenger_Safety/CPS-Factsheet.html](http://www.cdc.gov/MotorVehicleSafety/Child_Passenger_Safety/CPS-Factsheet.html). [2009 December 14].

^2^ Durbin, RD, Chen, I, Smith, R, Elliott MR, Winston, FK (2005). Effects of seating position and appropriate restraint use on the risk of injury to children in motor vehicle crashes. Pediatrics, 115, E305-309.

^3^ Ehiri, JE, Ejere, HOD, Magnussen, L, Emusu, D, King, W, Osberg, SJ (2006). Interventions for promoting booster seat use in four to eight year olds traveling in motor vehicles. Cochrane Database of Systematic Reviews (1), Art. No: CD004334. DOI: 10.1002/14651858.CD004334.pib2.

^4^ National SAFE KIDS Campaign (NSKC), Motor Vehicle Occupant Injury Fact Sheet.  Washington (DC): NSKC, 2004.

^5^ Flay, BR, Biglan, A, Boruch, RF, Ganzalez Castro, F, Gottfredson, D, Kellam, S, Moscicki, EK, Schinke, S, Valentine, JC, & Ji, P (2005). Standards of evidence: Criteria for efficacy, effectiveness and dissemination. Prevention Science, 6(3), 151-175.

Drug Law Enforcement Programs

Domain: Physical environment

Reasons for Policy:

- In 2002, the U.S. spent over 50% of the federal expenditure for drug control on domestic law enforcement.^1^
- Drug law enforcement is the primary component of national drug policies.^1^
- The “standard model,” or traditional approach of policing, involving unfocused strategies, rapid response to calls for service, routine patrol, and increasing the number of police in a jurisdiction, has little empirical support.^1^

Community Group:

- Local law enforcement
- Local government
- Community organizations

Policy Components:

- Policing approaches that include a level of focus and a diversity of approaches
- Hot-spots policing (e.g., raids, crackdowns, buy-bust operations)
- Problem-oriented policing (e.g., drug nuisance abatement, civil remedies)
- Community-wide policing (e.g., Weed and Seed)

Desired Outcomes:

- Reduce or prevent illicit drug use
- Reduce or prevent drug dealing
- Reduce drug-related calls for service and reported offenses
- Reduce drug incidents
- Reduce calls for service and reported offenses for non-drug offenses (e.g., property crime, violent offenses and disorder)

Level of Evidence Available to Evaluate Effectiveness of Policy (1 = strong evidence to 3 = insufficient evidence*):^2^

1 = Meets Criteria for Effectiveness

Achievable Results:

The following summary of achievable results is based on a published review of the scientific evidence.*

On average, problem-oriented policing can achieve:^1^

- Moderate effects on drug-related calls for service (Odds Ratio = 1.44, 95% CI: 1.16-1.77)^1^
- Large effects on total calls for service (Odds Ratio = 1.81, 95% CI: 1.21-2.72)^1^

On average, hotspots policing can achieve:

- Small effects on total offenses (Odds Ratio = 1.04, 95% CI: 1.01-1.08)^1^

Effects of community-, hotspot- and problem-oriented policing are not significant for drug offenses, offenses against the person, property offenses, crime-related calls for service, and disorder-related calls for service.^1^

Problem-oriented and community-wide policing approaches that seek to disrupt street-level drug market problems are likely to be a more productive approach to reducing drug problems than directed law enforcement focused on hotspots.^1^

Community Examples:

- Napa County, California, Napa County Sheriff’s Office, Operations Division includes a Problem Oriented Policing Program.
  - <http://www.countyofnapa.org/Pages/DepartmentContent.aspx?id=4294971229>
  - See pdf
- St. Louis, Missouri, employs Weed & Seed, a community-based strategy that aims to prevent, control, and reduce violent crime, drug abuse, and gang activity.
  - <http://www.weedandseedstl.org/default.asp?V_DOC_ID=843>
  - See pdf

Links to Policy Examples:

- Cal. Gov. Code §26600, Preservation of peace
  - See pdf
- 42 U.S.C.A §§ 3712a. Office of Weed and Seed strategies, 3712b. Weed and Seed strategies (Pub.L. 109-162, Title XI, § 1121(a))
  - See pdf

*Note: 1 = meets criteria for policy effectiveness (consistent, positive outcomes from at least two high-quality experimental or quasi-experimental trials using a comparison group or interrupted time series design)^2^; 2 = consistent evidence available linking policy with positive outcomes from high-quality observational studies only; 3 = insufficient evidence available for policy or policy components.

† Be sure to check with your state, county, and municipal governments regarding potential existing laws that may impede any new policy development.

‡Local governments and organizations may check existing state and federal statutes and administrative codes for the authority to implement local policies.

References

1 Mazerolle, L, Soole, DW, Rombouts, S (2007). Street-level drug law enforcement: A meta-analytic review. Campbell Systematic Reviews 2007, 2, DOI:10.4073/csr.2007.2.

2 Flay, BR, Biglan, A, Boruch, RF, Ganzalez Castro, F, Gottfredson, D, Kellam, S, Moscicki, EK, Schinke, S, Valentine, JC, & Ji, P (2005). Standards of evidence: Criteria for efficacy, effectiveness and dissemination. Prevention Science, 6(3), 151-175.

Enforcement of Minimum Legal Drinking Age Laws

Domain: Physical environment

Reasons for Policy:

- Traffic crashes are the leading cause of death among adolescents and 1/3 of those deaths involve alcohol.^1^
- Studies have shown that teens that start drinking before they are 21 are more likely to drink heavier as adults, compared to those that wait until they are 21.^1^
- The majority of alcohol outlets regularly sell alcohol to minors.^1^

Community Group:

- Local government
- Local law enforcement
- Local justice system

Policy Components:

- Greater enforcement of minimum age sales laws
- Efforts to reduce use of false identification
- Increased restrictions on home delivery of alcohol

Desired Outcomes:

- Lower rates of underage drinking
- Lower rates of traffic crashes involving alcohol
- Lower rates of other alcohol-related health and social problems (e.g., crime, unintentional injuries)

Level of Evidence Available to Evaluate Effectiveness of Policy (1 = strong evidence to 3 = insufficient evidence*):^2^

1 = Meets Criteria for Effectiveness

Achievable Results:

The following summary of achievable results is based on a published review of the scientific evidence.

On average, higher legal drinking ages can achieve:

- Significant reductions in alcohol consumption among underage youth^1^
- Significant reductions in traffic crashes^1^
- Lower rates of alcohol-related health and social problems (e.g., unintentional injuries, crime); although evidence is inconsistent across some outcomes^1^

Community Examples:

- Jefferson Parish, Louisiana reviews all liquor license holders regularly and revokes licenses of business who serve to underage patrons.
  - <http://www.nola.com/politics/index.ssf/2010/04/fat_city_bar_loses_alcohol_lic.html>
- Thief River Falls County (Minnesota) uses compliance checks to enforce the MLDA.
  - <http://www.nhtsa.gov/nhtsa/whatsup/SAFETEAweb/images/FY05/AnnRpts/Minnesota_2005AnnRpt.pdf> page 39

Links to Policy Examples: †

- Jefferson Parish, LA Ordinance Chapter 4 Article II section 4-32 to 4-34
  - <http://library.municode.com/html/14447/level3/PII_C4_AII.html#PII_C4_AII_s4-34>
- Thief River Falls, MN Title XI Business Regulations Chapter 111 Alcoholic Beverages § 111.100
  - <http://www.citytrf.net/CityCode/Ch%20110%20%20Busness%20Regulations.pdf>

*Note: 1 = meets criteria for policy effectiveness (consistent, positive outcomes from at least two high-quality experimental or quasi-experimental trials using a comparison group or interrupted time series design)^2^; 2 = consistent evidence available linking policy with positive outcomes from high-quality observational studies only; 3 = insufficient evidence available for policy or policy components.

† Be sure to check with your state, county, and municipal governments regarding potential existing laws that may impede any new policy development.

References

^1^Wagenaar AC & Toomey TL (2002). Effects of minimum drinking age laws: Review and analyses of the literature from 1960 to 2000. *Journal of Studies on Alcohol,* Supplement No. 14, 206-225.

^2^Flay, BR, Biglan, A, Boruch, RF, Ganzalez Castro, F, Gottfredson, D, Kellam, S, Moscicki, EK, Schinke, S, Valentine, JC, & Ji, P (2005). Standards of evidence: Criteria for efficacy, effectiveness and dissemination. Prevention Science, 6(3), 151-175.

Graduated Driver Licensing

Domain: Physical environment

Reasons for Policy:

- Motor vehicle crashes account for 40% of fatalities among adolescents aged 16 to 19 years.^1^
- Sixteen-year-old drivers have an especially high risk of crash involvement. Per mile driven, their crash rate is approximately 10 times the rate for drivers aged 30-59 years and more than twice the rate of 18- to 19-year old drivers.^2^
- The high risk for crashes is attributed to inexperience and immaturity of teen drivers.^3^

Community Group:

- State Government
- State Department of Motor Vehicles
- State Law Enforcement Agencies
- Local Government
- Local branch of Mothers Against Drunk Driving (MADD)

Policy Components:

- Three stage GDL program: (1) requiring an adult with a valid license be present at all times, (2) allow the new driver to drive along with some restrictions (e.g., no night-time driving, limitations on extra passengers, restrictions on blood alcohol concentrations), and (3) full licensure.

Desired Outcomes:

- Reduction in crashes among teen drivers and the general population
- Reduction in crash injuries and fatalities among teen drivers and the general population
- Reduction in hospital admissions due to crashes
- Reduction in crashes occurring during curfew hours
- Reduction in alcohol-involved crashes
- Reduction in traffic violations

Level of Evidence Available to Evaluate Effectiveness of Policy (1 = strong evidence to 3 = insufficient evidence*):^4^

1 = Meets Criteria for Effectiveness

Achievable Results:

The following summary of achievable results is based on a published review of the scientific evidence.

On average, graduated driver licensing programs can achieve:

- A 31% decrease in overall crash rates among 16 year-old drivers (range 26-41%)^5^
- A 28% decrease in injury crash rates among 16-year old drivers (range 4-43%)^5^

When GDL programs are more comprehensive, thoughtfully created, and thorough, (including extended

learner periods, nighttime restrictions, and passenger restrictions),^6^ they are more effective.^7^

Community Examples:

- Illinois, Office of the Secretary of State, Division of Traffic Safety has a graduated driver’s license program called “Graduate to Safety”.
  - <http://www.cyberdriveillinois.com/departments/drivers/programs/gdl.html>
  - see pdf
- Tennessee, Department of Safety has a graduated driver’s license program.
  - <http://www.tn.gov/safety/driverlicense/gdl.htm>
  - See pdf

Links to Policy Examples:

- Illinois Statutes, § 625 ILCS 5/2-104 (2010). Powers and duties of the Secretary of State
  - See pdf
- Illinois Administrative Code, § 1030.65 Instruction Permits (92 Ill. Adm. Code 1030.65 (2010))
  - See pdf
- Tennessee Code Annotated, § 55-50-311 Learner permits; intermediate driver licenses
  - See pdf

*Note: 1 = meets criteria for policy effectiveness (consistent, positive outcomes from at least two high-quality experimental or quasi-experimental trials using a comparison group or interrupted time series design)^4^; 2 = consistent evidence available linking policy with positive outcomes from high-quality observational studies only; 3 = insufficient evidence available for policy or policy components.

† Be sure to check with your state, county, and municipal governments regarding potential existing laws that may impede any new policy development.

References

^1^ Ferguson, SA, Leaf, WA, Williams, AF, Preusser, DF (1996). Differences in young driver crash involvement in states with varying licensure practices. Accident Analysis and Prevention, 28(2), 171-180.

^2^ Chen, LH, Baker, SP, & Li, G (2005). Graduated driver licensing programs and fatal crashes of 16-year-old drivers: A national evaluation. Pediatrics, 118, 56-62.

^3^ Foss, RD & Evenson, KR (1999). Effectiveness of graduated driver licensing in reducing motor vehicle crashes. American Journal of Preventive Medicine, 16(1), 47-56.

^4^ Flay, BR, Biglan, A, Boruch, RF, Ganzalez Castro, F, Gottfredson, D, Kellam, S, Moscicki, EK, Schinke, S, Valentine, JC, & Ji, P (2005). Standards of evidence: Criteria for efficacy, effectiveness and dissemination. Prevention Science, 6(3), 151-175.

^5^ Hartling, L, Wiebe, N, Russell, KR, Petruk, J, Spinola, C, & Klassen, TP (2005). Graduated driver licensing for reducing motor vehicle crashes among young drivers. Cochrane Database of Systematic Reviews, Issue 2, Art. No.: CD003300. DOI: 10.1002/14651858.CD003300.pub2

^6^ Wiliams, AF (2007). Contribution of the components of graduated licensing to crash reductions. Journal of Safety Research, 38(2), 177-184.

^7^ Shope, JT (2007). Graduated driver licensing: Review of evaluation results since 2002. Journal of Safety Research, 38(2), 165-175.

Hours of Sale for Alcohol

Domain: Physical environment

Reasons for Policy:

- In 2002, heavy alcohol consumption contributed to approximately 10% of the total burden of disease in economically developed countries^1^
- Alcohol consumption is third out of 26 leading risk factors contributing to disease, injury , and death^2^

Community Group:

- Local government
- Local law enforcement
- Local licensed alcohol vendors
- Local department that issues liquor licenses (different depending on state)

Policy Components:

- Limiting hours and days that alcohol can be sold, thereby, reducing the availability of alcohol

Desired Outcomes:

- Lower rates of interpersonal violence
- Lower rates of heavy alcohol consumption
- Lower rates of non-vehicle related injuries due to alcohol
- Lower rates of motor vehicle crashes due to alcohol

Level of Evidence Available to Evaluate Effectiveness of Policy (1 = strong evidence to 3 = insufficient evidence*):^3^

1 = Meets Criteria for Effectiveness

Achievable Results:

The following summary of achievable results is based on a published review of the scientific evidence.

- Controls on hours or days of sale result in significant reductions in alcohol consumption and related harm (e.g., violence).^1,2^

Community Examples:

- Titusville, Florida reduced the time of alcoholic beverage sales from 2:00am to 11:00pm in order to reduce the number of negative outcome related with alcohol use.
  - <http://www.titusville.com/Files/Regular%20City%20Council%207-14-09%20minutes.pdf>
- Washington, DC reduced the number of hours that retail outlets are able to sell alcoholic beverages by one hour.
  - http://media.www.gwhatchet.com/media/storage/paper332/news/2001/06/27/News/Abc-Law.Limits.Hours.For.Alcohol.Retailers-80522.shtml

Links to Policy Examples: †

- Titusville Florida Ordinance # 25-2009
  - http://www.titusville.com/Files/Ordinance%20No%2025-2009.pdf
- District of Columbia Official Code § 25-722
  - <http://weblinks.westlaw.com/result/default.aspx?cite=UUID%28N2B90584095%2DDE11DB9BCF9%2DDAC28345A2A%29&db=1000869&findtype=VQ&fn=%5Ftop&ifm=NotSet&pbc=4BF3FCBE&rlt=CLID%5FFQRLT55359261410294&rp=%2FSearch%2Fdefault%2Ewl&rs=WEBL10%2E04&service=Find&spa=DCC%2D1000&sr=TC&vr=2%2E0>

*Note: 1 = meets criteria for policy effectiveness (consistent, positive outcomes from at least two high-quality experimental or quasi-experimental trials using a comparison group or interrupted time series design)^3^; 2 = consistent evidence available linking policy with positive outcomes from high-quality observational studies only; 3 = insufficient evidence available for policy or policy components.

† Be sure to check with your state, county, and municipal governments regarding potential existing laws that may impede any new policy development.

References

^1^Stockwell T & Chikritzhs T (2009). Do relaxed trading hours for bars and clubs mean more relaxed drinking? A review of international research on the impacts of changes to permitted hours of drinking. *Crime Prevention and Community Safety,* 11, 153-170.

^2^Popova S, Giesbrecht N, Bekmuradov D, & Patra J (2009). Hours and days of sale and density of alcohol outlets: Impacts on alcohol consumption and damage: A systematic review. *Alcohol & Alcoholism,* 44(5), 500-516.

^3^Flay, BR, Biglan, A, Boruch, RF, Ganzalez Castro, F, Gottfredson, D, Kellam, S, Moscicki, EK, Schinke, S, Valentine, JC, & Ji, P (2005). Standards of evidence: Criteria for efficacy, effectiveness and dissemination. Prevention Science, 6(3), 151-175.

Red-light Cameras

Domain: Physical environment

Reasons for Policy:

- By 2020, road traffic injury is predicted to become the third greatest cause of death and disability in the world.^1^
- In 2006, there were 42,708 motor-vehicle crash fatalities in the U.S.^2^
- Motor-vehicle traffic-related injuries accounted for 24.4% of all injury deaths.^2^
- While traditional manual enforcement methods are resource intensive and entail risk, red-light cameras can operate 24 hours a day, in a non-discriminatory and safe manner.^1^

Community Group:

- Local government
- State government
- Transportation department

Policy Components:

- Cameras used at intersections to detect red-light offenders

Desired Outcomes:

- Decrease motor-vehicle traffic injuries
- Decrease motor-vehicle traffic crashes
- Decrease red-light violations

Level of Evidence Available to Evaluate Effectiveness of Policy (1 = strong evidence to 3 = insufficient evidence*):^3^

1 = Meets Criteria for Effectiveness

Achievable Results:

The following summary of achievable results is based on a published review of the scientific evidence.

On average, red-light cameras can achieve:

- Small reductions in total casualty crashes at intersections with traffic lights (RR = 0.84, 95% CI: 0.76-0.93).^1^
- Small reductions in right-angle casualty crashes at intersections with traffic lights (RR = 0.76, 95% CI: 0.58, 0.99).^1^

Note: There is insufficient evidence to indicate whether red-light cameras reduce total crashes and traffic

violations.^1^

Community Examples:

- City of Plano, Texas implements a Red Light Camera Program
  - <http://www.planotx.org/Departments/Police/RedLightCameras/Pages/default.aspx>
  - See pdf
- Hillsborough County, Florida implements a Red Light Camera Enforcement Ordinance.
  - <http://www.hillsboroughcounty.org/redforreason/home.cfm>
  - See pdf

Links to Policy Examples:

- Texas Transportation Code, Chapter 707 Photographic Traffic Signal Enforcement System, allows local authority to install red light cameras, requires local ordinance.
  - See pdf
- Plano, Texas, Code of Ordinances, Chapter 12, Article X Automated Traffic Signal Enforcement, §§ 12-260-269.
  - See pdf
- Florida House Bill 325, “Mark Wandall Traffic Safety Act.”, authorizes counties and municipalities to use traffic infraction detectors. See also F.S. § 316.008(8) Powers of local authorities.
  - See pdf
- Hillsborough County Red Light Camera Enforcement Ordinance, Ordinance 09-64.
  - See pdf

*Note: 1 = meets criteria for policy effectiveness (consistent, positive outcomes from at least two high-quality experimental or quasi-experimental trials using a comparison group or interrupted time series design)^3^; 2 = consistent evidence available linking policy with positive outcomes from high-quality observational studies only; 3 = insufficient evidence available for policy or policy components.

† Be sure to check with your state, county, and municipal governments regarding potential existing laws that may impede any new policy development.

‡Local governments and organizations may check existing state and federal statutes and administrative codes for the authority to implement local policies.

References

1 Aeron-Thomas, A, Hess, S (2005). Red-light cameras for the prevention of road traffic crashes. *Cochrane Database of Systematic Reviews* 2005, 2, CD003862.

2 Heron, HP, Hoyert, DL, Murphy, SL, Xu, JQ, Kochanek, KD, Tejada-Vera, B (2009). Deaths: Final data for 2006. National Center for Health Statistics. National Vital Statistics Report, 57(14).

3 Flay, BR, Biglan, A, Boruch, RF, Ganzalez Castro, F, Gottfredson, D, Kellam, S, Moscicki, EK, Schinke, S, Valentine, JC, & Ji, P (2005). Standards of evidence: Criteria for efficacy, effectiveness and dissemination. Prevention Science, 6(3), 151-175.

Responsible Beverage Service and Enforcement

Domain: Physical environment

Reasons for Policy:

- Pseudo-intoxicated patrons are successful in purchasing alcohol in 62%-90% of purchase attempts.^1^
- Approximately 1/3 of patrons leaving bars have blood alcohol concentrations above the legal limit.^1^
- 45%-50% of alcohol outlets sell alcohol to people under the minimum legal drinking age.^1^
- Alcohol is involved in a high percentage of health problems, such as fatal traffic accidents, rapes, drownings, assaults, and suicides^1^

Community Group:

- State government
- Local government
- Local law enforcement
- Local alcohol outlets

Policy Components:

- Responsible beverage service (RBS) programs that educate alcohol servers and clerks on strategies to avoid selling alcohol to underage and intoxicated patrons
- Training owners and managers of alcohol outlets on appropriate policies to monitor and teach staff
- Graduated administrative penalties for reported violations and incentives for voluntarily attending RBS training.
- Active, visible, and swift enforcement of RBS laws

Desired Outcomes:

- Reduce service to intoxicated patrons
- Reduce unlawful alcohol sales to patrons under the minimum legal drinking age
- Reduce alcohol consumption
- Reduce alcohol-related harm

Level of Evidence Available to Evaluate Effectiveness of Policy (1 = strong evidence to 3 = insufficient evidence*):^2^

1 = Meets Criteria for Effectiveness

Achievable Results:

The following summary of achievable results is based on a published review of the scientific evidence.

On average, studies on RBS training programs saw:

- Improvements in server knowledge and beliefs^1^
- Modest increases in responsible service practices (e.g. refusing to serve alcohol, asking about driving, giving food with beverages)^1^
- Modest reductions in alcohol consumption and blood alcohol concentrations among patrons^1^
- Modest reductions in alcohol-related traffic crashes^1^

On average, visible enforcement of RBS laws can achieve:

- 30% - 50% reductions in sales to underage youth^1^

RBS programs alone may not be sufficient to produce large, sustained effects on server/clerk sales to underage and intoxicated patrons. However, provision of training programs may be a prerequisite to implementation of more intense, successful enforcement efforts, which produce larger, and longer-lasting effects.

Community Examples:

- Danville, Kentucky requires all servers of alcoholic beverages to participate in a responsible beverage service program
  - <http://www.danvilleky.org/index.aspx?NID=247>
- Pinole, California requires employees who sell or serve alcohol to receive responsible beverage service training
  - <http://www.abc.ca.gov/programs/RBS.html>
  - <http://www.abc.ca.gov/forms/abc800.pdf>

Links to Policy Examples: †

- Danville, Kentucky art. XII, § 12.1-12.4
  - <http://www.danvilleky.org/DocumentView.aspx?DID=394>
- Pinole, California, Municapal Code Title 17, Chapter 60.040 Standard Condition
  - <http://www.ci.pinole.ca.us/planning.docs/CUP_Alcohol%20Sales.pdf>

*Note: 1 = meets criteria for policy effectiveness (consistent, positive outcomes from at least two high-quality experimental or quasi-experimental trials using a comparison group or interrupted time series design)^2^; 2 = consistent evidence available linking policy with positive outcomes from high-quality observational studies only; 3 = insufficient evidence available for policy or policy components.

† Be sure to check with your state, county, and municipal governments regarding potential existing laws that may impede any new policy development.

References

^1^Wagenaar AC, Tobler AL (2007). Alcohol sales and service to underage youth and intoxicated patrons: Effects of responsible beverage service training and enforcement interventions. *Transportation Research Circular: Traffic Safety and Alcohol Regulation,* E-C123, 141-163.

^2^Flay, BR, Biglan, A, Boruch, RF, Ganzalez Castro, F, Gottfredson, D, Kellam, S, Moscicki, EK, Schinke, S, Valentine, JC, & Ji, P (2005). Standards of evidence: Criteria for efficacy, effectiveness and dissemination. Prevention Science, 6(3), 151-175.

Safety Belt Laws and Enforcement

Domain: Physical environment

Reasons for Policy:

- The use of safety belts is the single most effective means of reducing fatal and nonfatal injuries in motor vehicle crashes.^1^
- Safety belts are approximately 45% effective in reducing fatalities in passenger cars and 60% effective in light trucks. ^2^
- Overall, 71% of motor vehicle occupants in 2000 wore safety belts, but teenagers consistently report lower than average usage rates.^3^
- Safety belt use is estimated to have saved 123,000 lives between 1975 and 1999, an estimated 9553 additional deaths would have been prevented in 1999 alone if all motor vehicle occupants consistently wore safety belts.^4^

Community Group:

- Law Enforcement Agencies
- Local and State Government
- Local Media

Policy Components:

- Extend safety belt laws to rear seat coverage and all ages groups
- Include fines for violation of laws and make into a primary enforcement law which allows a police officer to stop a motorist solely for not wearing a safety belt
- Enhance enforcement (i.e., increasing the number of officers on patrol, increasing citations for safety belt checkpoints).
- Increase public awareness of enforcement through media campaigns.

Desired Outcomes:

- Increase use of safety belts
- Reduce fatal and nonfatal injuries

Level of Evidence Available to Evaluate Effectiveness of Policy (1 = strong evidence to 3 = insufficient evidence*):^5^

1 = Meets Criteria for Effectiveness

Achievable Results:

The following summary of achievable results is based on a published review of the scientific evidence.

On average, safety belt laws can achieve:

- 9% decrease in fatal injuries (Range: 2-18%).^1^
- 8% decrease in fatal and nonfatal injuries combined (Range: 3-20%).^1^
- 16% increase in self-reported safety belt use (Range: 13-19%).^1^

On average, primary enforcement relative to secondary enforcement safety belt laws can achieve incremental effectiveness of:

- 8% decrease in fatal injuries (Range: 3-14%).^1^

On average, enhanced enforcement programs can achieve:^1^

- 16% increase in observed safety belt use (Range: 8-24%).^1^

Community Examples:

- 31 states, the District of Columbia, American Samoa, Guam, the Northern Mariana Islands, Puerto Rico and the Virgin Islands have primary seat belt laws
  - <http://www.ghsa.org/html/stateinfo/laws/seatbelt_laws.html>
- Minnesota’s primary seat belt law requires any age passenger, in all seating positions, must be buckled up or in a correct child restraint.
  - <http://www.dps.state.mn.us/ots/topic_areas/belts/belts_default.asp>
- Tampa, Florida, Hillsborough County Sheriff’s Office conducted “Operation Belts or El$e” to enforce Florida’s new enhanced seat belt law.
  - <http://www2.tbo.com/content/2009/jun/30/301801/na-seat-belt-no-longer-optional/news-metro/>

Links to Policy Examples:

- Minnesota Statutes §§ 169.684 Seat Belt; Declaration of Policy, 169.685 Seat Belt; Passenger Restraint System for Children, 169.686 Seat Belt Use Required; Penalty
  - <https://www.revisor.mn.gov/statutes/?id=169.684>
  - <https://www.revisor.mn.gov/statutes/?id=169.685>
  - <https://www.revisor.mn.gov/statutes/?id=169.686>
- Florida Statutes § 316.614 Safety belt usage, “Florida Safety Belt Law”
  - <http://www.leg.state.fl.us/statutes/index.cfm?App_mode=Display_Statute&Search_String=&URL=0300-0399/0316/Sections/0316.614.html>

*Note: 1 = meets criteria for policy effectiveness (consistent, positive outcomes from at least two high-quality experimental or quasi-experimental trials using a comparison group or interrupted time series design)^5^; 2 = consistent evidence available linking policy with positive outcomes from high-quality observational studies only; 3 = insufficient evidence available for policy or policy components.

† Be sure to check with your state, county, and municipal governments regarding potential existing laws that may impede any new policy development.

References

^1^ Dinh-Zarr, TB, Sleet, DA, Shults, RA, Zaza, S, Elder, RW, Nichols, JL, Thompson, RS, Sosin, DM, & the Task Force on Community Preventive Services (2001). Reviews of existing evidence regarding interventions to increase the use of safety belts. American Journal of Preventive Medicine, 21(4S), 48-65.

^2^ Evans, L (1986). The effectiveness of safety belts in preventing fatalities. Accident Analysis & Prevention, 18, 229-241.

^3^ National Highway Traffic Safety Administration (2000). Observed safety belt use from December 1999 and June 2000. MiniNOPUS. Washington, DC: U.S. Department of Transportation, National Highway Traffic Safety Administration.

^4^ National Highway Traffic Safety Administration (2000). Traffic safety facts 1999: occupant protection. Washington, DC: U.S. Department of Transportation, National Highway Traffic Safety Administration, 2000. DOT HS 809 090.

^5^ Flay, BR, Biglan, A, Boruch, RF, Ganzalez Castro, F, Gottfredson, D, Kellam, S, Moscicki, EK, Schinke, S, Valentine, JC, & Ji, P (2005). Standards of evidence: Criteria for efficacy, effectiveness and dissemination. Prevention Science, 6(3), 151-175.

Street Lighting

Domain: Physical Environment

Reasons for Policy:

- Street lighting could reduce crime through improved visibility and investment in neighborhood conditions.^1^
- Street lighting may prevent traffic crashes by improving a driver’s visual capabilities and ability to detect road hazards.^2^

Community Group:

- Local Government
- State Department of Transportation

Policy Components:

- Improved street lighting in neighborhood public areas
- New or improved street lighting on un- or under-lit roads and intersections

Desired Outcomes:

- Reduced neighborhood crime
- Reduced community traffic injuries
- Improved community connectedness and cohesion

Level of Evidence Available to Evaluate Effectiveness of Policy (1 = strong evidence to 3 = insufficient evidence*):^3^

1 = Meets Criteria for Effectiveness

Achievable Results:

The following summary of achievable results is based on a published review of the scientific evidence.

On average, street lighting can achieve:

- 21% decrease in crime (Relative Effect Size = 1.27, 95% CI 1.09-1.47)^1^
- 55% decrease in traffic crashes (RR = 0.45, 95% CI 0.29-0.69, pg. 10)^2^
- 22% decrease in injury crashes (RR=0.78, 95% CI 0.63-0.97, pg. 13)^2^

Community Examples:

- Arlington, Virginia the Department of Environmental Services, Transportation Engineering Division is responsible for the street lights in the county
  - <http://www.arlingtonva.us/departments/EnvironmentalServices/dot/traffic/streetlights/EnvironmentalServicesHb14.aspx>
- Minneapolis, Minnesota city website provides a way for residents to report street light trouble and to request a new street light
  - <http://www.ci.minneapolis.mn.us/streetlighting/issues.asp>

Links to Policy Examples:

- Arlington, Virginia Arlington County Street Light Policy and Planning Guide
  - <http://www.arlingtonva.us/departments/EnvironmentalServices/dot/traffic/streetlights/images/file60881.pdf>
- Minneapolis, Minnesota Public Works Department is in charge of maintaining local street lights
  - http://www.ci.minneapolis.mn.us/streetlighting/
  - http://www.ci.minneapolis.mn.us/streetlighting/docs/mpls-street-lighting-policy.pdf

*Note: 1 = meets criteria for policy effectiveness (consistent, positive outcomes from at least two high-quality experimental or quasi-experimental trials using a comparison group or interrupted time series design)^3^; 2 = consistent evidence available linking policy with positive outcomes from high-quality observational studies only; 3 = insufficient evidence available for policy or policy components.

† Be sure to check with your state, county, and municipal governments regarding potential existing laws that may impede any new policy development.

References

1 Welsh BP, Farrington DC (2008). Effects of improved street lighting on crime. Campbell Systematic Reviews, 2008:13.

2 Beyer FR, Ker K (2009). Street lighting for preventing road traffic injuries. Cochrane Database of Systematic Reviews, 2009, Issue 1. Art. No.: CD004728.

3 Flay, BR, Biglan, A, Boruch, RF, Ganzalez Castro, F, Gottfredson, D, Kellam, S, Moscicki, EK, Schinke, S, Valentine, JC, & Ji, P (2005). Standards of evidence: Criteria for efficacy, effectiveness and dissemination. Prevention Science, 6(3), 151-175.

Tobacco Advertising Restrictions

Domain: Physical Environment

Reasons for Policy:

- In the U.S. there are over 400,000 premature deaths per year due to smoking.^1^
- Tobacco advertising increases tobacco consumption.^1^
- Youth experimenting with cigarettes develop a more positive attitude toward smoking and smokers after exposure to tobacco advertisements.^2^

Community Group:

- Local health department/board
- Local government
- State government
- Federal government

Policy Components:

- Tobacco product advertising bans for television advertising, radio advertising, print media advertising, internet advertising, billboard advertising, point of purchase advertising, movie advertising, and sponsorship bans.
- Stricter advertising restrictions (greater degree of government control on tobacco advertising)
- Comprehensive or total advertising bans

Desired Outcomes:

- Decrease in youth tobacco use
- Decrease in youth exposure to tobacco products

Level of Evidence Available to Evaluate Effectiveness of Policy (1 = strong evidence to 3 = insufficient evidence*):^3^

1 = Meets Criteria for Effectiveness (Comprehensive Bans only)

Achievable Results:

The following summary of achievable results is based on a published review of the scientific evidence.

On average, comprehensive or total advertising bans can achieve: ^1,2^

- Small, but significant reductions in tobacco use.

The evidence from the highest-quality studies indicates that comprehensive or total advertising bans can significantly reduce tobacco consumption. Limited or isolated sets of advertising bans have not shown a significant effect in reducing youth smoking. Limited bans result in tobacco advertising in the remaining non-banned media. However, local limited bans may have an effect in reducing tobacco consumption when combined with national or state bans and educational programs.^1,2^

On June 22, 2009 President Obama signed the Family Smoking and Prevention and Tobacco Control Act, giving the FDA the authority to regulate tobacco products and tobacco advertising.^4^

Community Examples:

- Buffalo, New York, Buffalo Common Council to draft an ordinance regulating tobacco ads.
  - <http://www.buffalonews.com/2010/07/06/1105240/council-backs-plan-to-regulate.html>
- Island County, Washington, Island County Board of Health proposed a tobacco advertising resolution to reduce marketing of tobacco products to children.
  - <http://www.islandcounty.net/health/Envh/tobacco_outdoor.htm>

Links to Policy Examples:

- Buffalo, New York drafts The Responsible Tobacco Retailing Act, Chapter 197 of the Code of the City of Buffalo.
  - See pdfs
- Island County, Washington, Island County Board of Health can enact local rules and regulations pursuant to Revised Code of Washington, §70.05.060(3) Powers and duties of local board of health
  - See pdf

*Note: 1 = meets criteria for policy effectiveness (consistent, positive outcomes from at least two high-quality experimental or quasi-experimental trials using a comparison group or interrupted time series design)^3^; 2 = consistent evidence available linking policy with positive outcomes from high-quality observational studies only; 3 = insufficient evidence available for policy or policy components.

† Be sure to check with your state, county, and municipal governments regarding potential existing laws that may impede any new policy development.

References

1 Saffer H, Chaloupka F (2000). The effect of tobacco advertising bans on tobacco consumption. *Joural of Health Economics*, 19, 1117-1137.

2 Willemsen MC, De Zwart WM (1999) The effectiveness of policy and health education strategies for reducing adolescent smoking: a review of the evidence. *Journal of Adolescence*, 22, 587-599.

3 Flay, BR, Biglan, A, Boruch, RF, Ganzalez Castro, F, Gottfredson, D, Kellam, S, Moscicki, EK, Schinke, S, Valentine, JC, & Ji, P (2005). Standards of evidence: Criteria for efficacy, effectiveness and dissemination. Prevention Science, 6(3), 151-175.

4 Family Smoking Prevention and Tobacco Control Act, Pub. L. No. 111-31 (2009). Library of Congress, Retrieved July 19, 2010, from <http://thomas.loc.gov/home/LegislativeData.php>

Tobacco Excise Taxes

Domain: Physical environment

Reasons for Policy:

- Excise taxes on tobacco products make the use of tobacco less attractive to adolescents and young adults who have limited resources and a variety of options for spending available money.^1^
- Tax increases for cigarettes are increasingly acceptable.^2^
- An effect of higher price on cigarettes may reduce the number of smokers.^2^
- Taxes on cigarettes may be justified because smokers impose costs on others which may exceed tax levels.^2^

Community Group:

- Local government
- State government

Policy Components:

- Imposing an excise tax on tobacco products
- Increasing an already existing excise tax on tobacco products

Desired Outcomes:

- Decrease in tobacco use among adolescents and young adults
- Reduction in the number of smokers

Level of Evidence Available to Evaluate Effectiveness of Policy (1 = strong evidence to 3 = insufficient evidence*):^3^

1 = Meets Criteria for Effectiveness

Achievable Results:

The following summary of achievable results is based on a published review of the scientific evidence.

On average, an increase in the price of tobacco products can achieve:

- A decrease in both overall prevalence of tobacco product use and consumption of tobacco products.^1^
- Reductions in tobacco use in both adolescents and young adults.^1^

On average, a 10% increase in tobacco product price can achieve:

- 2.3% decrease in the quantity of product consumed by adolescent users.^1^
- 3.7% decrease in tobacco use participation among young adults.^1^

Note: Effect sizes were measured by changes in price elasticity of demand estimates (i.e. a negative price elasticity of demand estimate reflects a decrease in tobacco use in response to an increase in tobacco product price).

Community Examples:

- Anchorage, Alaska, imposes an excise tax on cigarettes and other tobacco products.
  - <http://www.muni.org/departments/finance/treasury/programtaxes/tobaccotax/Pages/default.aspx>
- Cook County, Illinois, imposes a cigarette tax. In addition, there is an Illinois state tax on cigarettes
  - <http://www.usatoday.com/news/health/2004-04-02-chicago-tax_x.htm>
  - <http://www.chicagobreakingnews.com/2010/05/illinois-house-panel-oks-extra-1-cigarette-tax.html>

Note: High tobacco excise taxes may encourage avoidance:

- <http://www.huffingtonpost.com/2010/05/20/chicago-cigarette-tax-stu_n_583398.html>
- <http://articles.chicagotribune.com/2010-09-12/business/ct-biz-0912-tax-stamps--20100912_1_cigarette-tax-stamps-tax-collection>

Links to Policy Examples:

- Anchorage Municipal Code, Chapter 12.40 Excise Tax on Cigarettes and Tobacco (specifically §§ 12.40.05, .010, .020)
  - See Municode pdf
- Cook County Tobacco Tax Ordinance (Ord No. 09-O-15); Cook County, Illinois, Code of Ordinances, Chapter 74, Article XI – Tobacco Tax (§§ 74.430-.448)
  - See pdf
- Illinois Statutes, § 35 ILCS 130 Cigarette Tax Act
  - See pdf

*Note: 1 = meets criteria for policy effectiveness (consistent, positive outcomes from at least two high-quality experimental or quasi-experimental trials using a comparison group or interrupted time series design)^3^; 2 = consistent evidence available linking policy with positive outcomes from high-quality observational studies only; 3 = insufficient evidence available for policy or policy components.

† Be sure to check with your state, county, and municipal governments regarding potential existing laws that may impede any new policy development.

‡Local governments and organizations may check existing state and federal statutes and administrative codes for the authority to implement local policies.

References

1 Hopkins DP, et al. (2001). Reviews of Evidence Regarding Interventions to Reduce Tobacco Use and Exposure to Environmental Tobacco Smoke *American Journal of Preventive Medicine,* 20(2S), 16-66.

2 Grossman, M, Sindelar, JL, Mullahy, J, Anderson, R (1993). Alcohol and Cigarette Taxes. *Journal of Economic Perspectives,* 7(4), 211-222

3 Flay, BR, Biglan, A, Boruch, RF, Ganzalez Castro, F, Gottfredson, D, Kellam, S, Moscicki, EK, Schinke, S, Valentine, JC, & Ji, P (2005). Standards of evidence: Criteria for efficacy, effectiveness and dissemination. Prevention Science, 6(3), 151-175.

Smoke-Free Policies

Domain: Physical environment

Reasons for Policy:

- Secondhand smoke exposure is linked with cancer, heart disease, respiratory illness and is the leading source of indoor air pollution.^1^
- In the United States, secondhand smoke exposure causes approximately 46,000 heart disease deaths among adult nonsmokers per year.^2^
- In the U.S, secondhand smoke exposure causes 3,400 lung cancer deaths among adult nonsmokers per year.^2^

Community Group:

- Local government
- State government
- Local businesses
- Universities and colleges

Policy Components:

- Totally smoke-free workplaces (private or corporate business workplace, government workplace)
- Smoke-free public places (restaurants and bars)
- Smoke-free campuses
- Legislation restricting smoking in public and in workplaces

Desired Outcomes:

- Decreased prevalence of smoking
- Decreased daily cigarette consumption
- Decreased exposure to second-hand smoke

Level of Evidence Available to Evaluate Effectiveness of Policy (1 = strong evidence to 3 = insufficient evidence*):^3^

1 = Meets Criteria for Effectiveness

Achievable Results:

The following summary of achievable results is based on a published review of the scientific evidence.

On average, smoke-free workplace policies can achieve:

- 29% reduction in total cigarette consumption per employee (95% CI: 11%, 53%).^1^
- 3.8% reduction in smoking prevalence (95% CI: 2.8%, 4.7%).^1^
- Decrease in consumption of 3.1 cigarettes per day per smoker (95% CI: 2.4, 3.8).^1^
- Teenagers who worked in totally smoke-free worksites were 32% less likely to ever smoke than those who worked in less restricted work sites.^1^
- 60-70% reduction in self-reported and measured environmental tobacco smoke presence^4^

On average, local and state legislation restricting smoking in public and in workplaces can achieve:

- Decreases in consumption of 0.16 -0.73 cigarettes per day per capita.^1^
- 3.7% -4.5% reduction in smoking prevalence.^1^

Community Examples:

- University of Florida is a tobacco-free campus and prohibits tobacco use on all university-owned properties
  - <http://news.ufl.edu/2010/06/23/no-tobacco/>
- Triangle Area, North Carolina, Duke University Health System, UNC Health Care, and WakeMed Health & Hospitals are tobacco-free health systems.
  - <http://www.unchealthcare.org/site/aboutus/tobaccoannounce>
  - <http://www.unchealthcare.org/site/aboutus/tobaccofree>

Links to Policy Examples:

- Regulations of the University of Florida, UF-2.022; No Smoking and Tobacco Use
  - <http://regulations.ufl.edu/chapter2/2022.pdf>
- University of North Carolina, UNC Health Safety Policy Manual, #52 Smoking Policy
  - <http://www.unchealthcare.org/site/healthpatientcare/patient/other/smoking.html>
- Duke University, Duke Human Resources, Workplace Health and Safety, 10.02 Smoking Policy
  - <http://www.hr.duke.edu/policies/health_safety/smoking.php>

*Note: 1 = meets criteria for policy effectiveness (consistent, positive outcomes from at least two high-quality experimental or quasi-experimental trials using a comparison group or interrupted time series design)^3^; 2 = consistent evidence available linking policy with positive outcomes from high-quality observational studies only; 3 = insufficient evidence available for policy or policy components.

† Be sure to check with your state, county, and municipal governments regarding potential existing laws that may impede any new policy development.

‡Local governments and organizations may check existing state statutes and administrative codes for the authority to implement local policies.

References

1 Fichtenberg, CM, Glantz, SA (2002) Effect of smoke-free workplaces on smoking behavior: systematic review. *British Medical Journal*, 325(7357), 188-195.

2 Centers for Disease Control and Prevention. Smoking-Attributable Mortality, Years of Potential Life Lost, and Productivity Losses- United States, 2000-2004. *Morbidity and Mortality Weekly Report*,57(45), 1226–1228.

3 Flay, BR, Biglan, A, Boruch, RF, Ganzalez Castro, F, Gottfredson, D, Kellam, S, Moscicki, EK, Schinke, S, Valentine, JC, & Ji, P (2005). Standards of evidence: Criteria for efficacy, effectiveness and dissemination. Prevention Science, 6(3), 151-175.

4 Hopkins, D.P., Briss, P.A., Ricard, C.J. et al. (2001). Reviews of evidence regarding interventions to reduce tobacco use and exposure to environmental tobacco smoke. American Journal of Preventive Medicine, 20(2S), 16-66.

Urban Design and Land Use Policies

Domain: Physical Environment

Reasons for Policy:

- Only 27% of students in grades 9-12 get the recommended amount of physical activity per week^1^
- The direct costs of inactivity each year in the US are approximately $24 billion^1^
- Approximately 200,000-300,000 premature deaths each year are due to physical inactivity^1^
- Approximately 40% of children are inhibited from walking or cycling to school because of perceived traffic dangers^2^

Community Group:

- Local government
- Local builder’s associations

Policy Components:

- Build commercial areas within walking or biking distance from residential areas (mixed land use)
- Safe, clean, and continuous sidewalks
- Use of landscaping to make walking more attractive
- Improved street lighting
- Adding bicycle lanes
- Use of traffic calming strategies (e.g. center islands or raised crosswalks)

Desired Outcomes:

- Increased physical activity
- Improved safety for pedestrians/fewer pedestrian injuries
- Improved health
- Reduced obesity
- Cleaner air

Level of Evidence Available to Evaluate Effectiveness of Policy (1 = strong evidence to 3 = insufficient evidence*):^3^

1 = Meets Criteria for Effectiveness

Achievable Results:

The following summary of achievable results is based on a published review of the scientific evidence.

On average, changing community- and street-scale urban design and land use policies can achieve:

- 35% median increase in physical activity^1^

Community Stories:

- Lewiston, Maine implemented a comprehensive street light improvement campaign in the city.
  - http://www.ci.lewiston.me.us/energysaving/streetlights/index.htm
- Penfield (New York) has made substantial progress in adding sidewalks to major areas of the city in order to link residential and commercial areas.
  - http://www.penfield.org/index.php?pr=dpt-engineer-sidewalks

Policy Examples: †

- Lewiston, ME Master Policy #82
  - http://www.ci.lewiston.me.us/clerk/masterpolicies/082-StreetLightingPolicy.PDF
- Penfield, NY IV-7
  - http://www.penfield.org/media/dpt_engineering_Sidewalk_Policy.pdf

*Note: 1 = meets criteria for policy effectiveness (consistent, positive outcomes from at least two high-quality experimental or quasi-experimental trials using a comparison group or interrupted time series design)^3^; 2 = consistent evidence available linking policy with positive outcomes from high-quality observational studies only; 3 = insufficient evidence available for policy or policy components.

† Be sure to check with your state, county, and municipal governments regarding potential existing laws that may impede any new policy development.

References

^1^Heath G, et al. The effectiveness of urban design and land use and transport policies and practices to increase physical activity: a systematic review. Journal of Physical Activity and Health. 2006;3(Suppl 1):S55-S76.

^2^Giles-Corti B, Kelty SF, Zubrick SR, Villanueva KP (2009). Encouraging walking for transport and physical activity in children and adolescents: How important is built environment? Sports Medicine, 39(12), 995-1009.

^3^Flay, BR, Biglan, A, Boruch, RF, Ganzalez Castro, F, Gottfredson, D, Kellam, S, Moscicki, EK, Schinke, S, Valentine, JC, & Ji, P (2005). Standards of evidence: Criteria for efficacy, effectiveness and dissemination. Prevention Science, 6(3), 151-175.

Water Fluoridation

Domain: Physical Environment

Reasons for Policy:

- Tooth decay is the most common chronic disease of childhood^1^ and disproportionately affects low income and minority children^2,3^
- More than 50% of children and 80% of adolescents have dental caries^4^
- Water fluoridation is the most effective measure in preventing oral disease on a large scale^5^
- Less than 65% of the US population served by public water systems have access to water fluoridated at the optimal levels^6^

Community Group:

- Local Water Department
- Local Public Health Department
- Local Oral Health Professionals
- Dental Public Health Advocates
- Local Government

Policy Components:

- Fluoridate public water supplies to an optimal level of 1mg/L
- Educate public about proven safety and benefits of fluoridated water

Desired Outcomes:

- Improvements in oral health of children and adolescents
- Reduced costs of pediatric dental care

Level of Evidence Available to Evaluate Effectiveness of Policy (1 = strong evidence to 3 = insufficient evidence*):

1 = Meets Criteria for Effectiveness

The following summary of achievable results is based on a published review of the scientific evidence.^7^

On average, water fluoridation can achieve:

| Outcome | % Change | Direction |
| --- | --- | --- |
| Dental cavities^8^ | 50%-60% | Decrease |

Community Examples:

- Seattle, Washington uses water fluoridation to reduce the occurrence of dental cavities.
  - http://www.kingcounty.gov/healthservices/health/personal/oralhealth/fluoridation.aspx
- New York, New York in conjunction with its state health department has included fluoride in its water supply for its oral health benefits.
  - http://www.health.state.ny.us/prevention/dental/fluoridation/benefits.htm

Links to Policy Examples: †

- Seattle Washington Municipal Code, Title 10 chapter 10.22.010
  - http://library.municode.com/index.aspx?clientId=13857&stateId=47&stateName=Washington
- New York, New York Municipal Code, Title 24 §141.05
  - http://24.97.137.100/nyc/RCNY/Title24_141_05.asp?zoom_highlight=fluoridation

*Note: 1 = meets criteria for policy effectiveness (consistent, positive outcomes from at least two high-quality experimental or quasi-experimental trials using a comparison group or interrupted time series design)^7^; 2 = consistent evidence available linking policy with positive outcomes from high-quality observational studies only; 3 = insufficient evidence available for policy or policy components.

† Be sure to check with your state, county, and municipal governments regarding potential existing laws that may impede any new policy development.

References

^1^ Nelson, WE, ed. Textbook of Pediatrics. 15^th^ ed. Philadelphia, PA: WB Saunders; 1996, 628.

^2^ General Accounting Office. Oral health: Dental disease is a chronic problem among low-income populations. Report GAO/HEHS-00-72. Available at <http://www.gao.gov>.

^3^ US Inspector General. Children’s Dental Services under Medicaid: Access and Utilization*.* San Francisco, CA: US Department of Health and Human Services; 1996. Publication 09-93-00240.

^4^ Mouradian, WE, Wehr, E, & Crall, JJ (2000). Disparities in Children’s Oral Health and Access to Dental Care. JAMA 2000;284(20), 2625-2631.

^5^ Centers for Disease Control and Prevention: Public Health Service report on fluoride benefits and risks. JAMA 2001;266(8), 1061-1067.

^6^ US Public Health Service. Healthy People 2000 Progress Report on Oral Health. Washington, DC: US Dept of Health and Human Services; 1995.

^7^ Flay, BR, Biglan, A, Boruch, RF, Ganzalez Castro, F, Gottfredson, D, Kellam, S, Moscicki, EK, Schinke, S, Valentine, JC, & Ji, P (2005). Standards of evidence: Criteria for efficacy, effectiveness and dissemination. Prevention Science, 6(3), 151-175.

^8^ Richmond, VL (1985). Thirty years of fluoridation: A review. American Journal of Clinical Nutrition; (41)129-138.

Hot-Spot Policing

Domain: Physical Environment

Reasons for Policy:

- Crime is concentrated in small geographic areas, called “hot spots.”^1^
- Hot spots account for half of all criminal activity.^1^
- Physical and social characteristics of neighborhoods are associated with crime hot spots.^1^

Community Group:

- Local government
- Local law enforcement

Policy Components:

- Use of crime mapping to determine hot spots
- Focused intervention and patrolling in hot spots (e.g. crack house raids, targeted beat patrols)

Desired Outcomes:

- Reduce crime
- Improve neighborhood safety
- Reduce citizen calls for police services

Level of Evidence Available to Evaluate Effectiveness of Policy (1 = strong evidence to 3 = insufficient evidence*):^2^

1 = Meets Criteria for Effectiveness

Achievable Results:

The following summary of achievable results is based on a published review of the scientific evidence.

On average, hot spot policing can achieve:

- Small to large effects on all citizen calls for services (Effect Size: 0.37-0.67)^1^
- Small effects on citizen disorder calls (Effect Size: 0.16-0.28)^1^

Community Examples:

- Rockland County, NY police department targets specific areas in the city to improve public safety
  - <http://criminaljustice.state.ny.us/crimnet/ojsa/impact/index.htm>
- San Diego, CA police department uses “community oriented policing” and “problem oriented policing” to target and fight crime in specific neighborhoods
  - <http://www.sandiego.gov/police/about/problem.shtml>
- Green Bay, WI police department uses community policing to proactively fight crime in specific neighborhood “hot spots”
  - <http://www.gbpolice.org/comm/>

Links to Policy Examples†:

- County of Rockland, NY Resolution 33 in 2007 accepts funds to support Project I.MP.A.C.T.
  - See pdf in the helpful brief links folder titled Rockland Hot Spot Policing Resolution
  - <http://library6.municode.com:80/nonfolio/template.htm?view=browse&doc_action=setdoc&doc_keytype=tocid&doc_key=c5365d29a42f98772b4f3ae4d2984dd5&infobase=30200>
- San Diego, CA Chapter 2 art. 6 of the municipal code created a Citizens Advisory Board on Police/Community relations to aid police by developing and recommending strategies for crime prevention
  - <http://docs.sandiego.gov/municode/MuniCodeChapter02/Ch02Art06Division08.pdf>
- Green Bay, WI Ordinance Chapter 28 allows the city to fine and bill for police services if a property is a “chronic” nuisnace
  - <http://www.gbpolice.org/landlords/overview28.html>

*Note: 1 = meets criteria for policy effectiveness (consistent, positive outcomes from at least two high-quality experimental or quasi-experimental trials using a comparison group or interrupted time series design)^2^; 2 = consistent evidence available linking policy with positive outcomes from high-quality observational studies only; 3 = insufficient evidence available for policy or policy components.

† Be sure to check with your state, county, and municipal governments regarding potential existing laws that may impede any new policy development.

References

^1^Braga AA. The effects of hot spots policing on crime. Campbell Systematic Reviews 2007:1

^2^Flay, BR, Biglan, A, Boruch, RF, Ganzalez Castro, F, Gottfredson, D, Kellam, S, Moscicki, EK, Schinke, S, Valentine, JC, & Ji, P (2005). Standards of evidence: Criteria for efficacy, effectiveness and dissemination. Prevention Science, 6(3), 151-175.

Access to Affordable (or Free) Quality Childcare Services

Domain: Family Influences

Reasons for Policy:

- Early interventions targeting low-income children have higher success rates than late interventions.^1^
- There is a high need for affordable early childcare for working parents; 54% of mothers with children under age of one are in the labor force and 70% of mothers with children under age of six work at least 35 hours a week.^1^
- Racial and ethnic gaps in school readiness can widen if childcare quality is not high.^2^

Community Group:

- Local government
- Non-profits/Community organizations
- Businesses

Policy Components:

- Early childcare that offers a range of operating hours.
- Childcare from infancy to school-age.
- Childcare that addresses child’s developmental needs and parent’s employment needs.
- Demand-side subsidies to help parents pay for childcare.
- Quality standards, ratings, and tiered reimbursement strategies to promote high-quality centers.
- Paid family leave and flexible work schedules.

Desired Outcomes:

- Higher IQ
- Higher cognitive abilities
- Lower delinquency rates
- Less time spent in special education or grade retention
- Increased parental productivity in the workforce
- Increased parental wages

Level of Evidence Available to Evaluate Effectiveness of Policy (1 = strong evidence to 3 = insufficient evidence*):

1 = Meets Criteria for Effectiveness

Achievable Results:

The following summary of achievable results is based on a published review of the scientific evidence.

| Outcome | % Change | Direction |
| --- | --- | --- |
| IQ at age 3^3^ | 4.2 | Increase |
| IQ at age 5^3^ | 4.1 | Increase |
| Grade retention^3^ | 41.5 | Decrease |
| Special education^3^ | 59.6 | Decrease |
| 5 or more arrests^3^ | 74.8 | Decrease |
| Arrests for drug deals^3^ | 72.1 | Decrease |

Positive effects have also been achieved in parental workplace productivity, stress, and missed days of work.^1^

Community Examples:

- Los Angeles County, California, Office of Child Care facilitates high quality childcare and provides subsidies to parents who are unable to afford childcare.
  - http://ceo.lacounty.gov/ccp/cel.htm
- Brown County, Minnesota provides financial assistance through childcare subsidies to low-income families.
  - http://www.co.brown.mn.us/Departments/CSB/FinAssist/finassist.htm

Links to Policy Examples†:

- Los Angeles County, CA
  - http://ceo.lacounty.gov/ccp/pdf/policy/State%20Agenda%202009-10%20Legislative%20Session.pdf
- Brown county, MN
  - http://www.co.brown.mn.us/Departments%5CCSB%5CFinAssist%5Cbcfschildcarepolproc1011.pdf

*Note: 1 = meets criteria for policy effectiveness (consistent, positive outcomes from at least two high-quality experimental or quasi-experimental trials using a comparison group or interrupted time series design)^4^; 2 = consistent evidence available linking policy with positive outcomes from high-quality observational studies only; 3 = insufficient evidence available for policy or policy components.

† Be sure to check with your state, county, and municipal governments regarding potential existing laws that may impede any new policy development.

References

^1^ Morrissey, TW, Warner, ME (2007). Why early care and education deserves as much attention, or more, than prekindergarten alone. Applied Development Science, 11(2), 47-70.

^2^ Magnuson, KA, Waldfogel, J (2005). Early childhood care and education: Effects on ethnic and racial gaps in school readiness. The Future of Children, 15(1), 169-196.

^3^ Zoritch, B, Roberts, I, Oakley, A (2000). Day care for pre-school children. Cochrane Database of Systematic Reviews, Issue 3. Art. No.: CD000564.

^4^ Flay, BR, Biglan, A, Boruch, RF, Ganzalez Castro, F, Gottfredson, D, Kellam, S, Moscicki, EK, Schinke, S, Valentine, JC, & Ji, P (2005). Standards of evidence: Criteria for efficacy, effectiveness and dissemination. Prevention Science, 6(3), 151-175.

Promoting Breastfeeding

Domain: Family Influences

Reasons for Policy:

- Breastfeeding is related to many infant and maternal health benefits including, decreased infant infection and disease and decreased maternal postpartum bleeding and possibly ovarian cancer.^1^
- Only 29% of mothers in the United States continue breastfeeding to the recommended 6-12 months postpartum, with lower rates occurring in socially disadvantaged women.^1^
- A number of factors influence breastfeeding initiation and continuation, including personal characteristics and attitudes, hospital policies and intrapartum experience, and sources of support.^1^

Community Group:

- State government
- Local government
- Local hospitals
- Local public health department
- Local businesses

Policy Components:

- Instruction to new mothers that is interactive in nature
- Group or personal modalities for instruction
- Facilitating peer support
- Workplace policies that support continuation of breastfeeding (e.g., leave policies, tax incentives, cultural involvement)
- Hospital policies that support initiation and continuation of breastfeeding (e.g., rooming-in, early discharge, supplementary feedings)

Desired Outcomes:

- Higher rates of breastfeeding to the recommended 6-12 months postpartum
- Higher rates of breastfeeding-related health benefits for both infant and mother
- Greater social support for new mothers

Level of Evidence Available to Evaluate Effectiveness of Policy (1 = strong evidence to 3 = insufficient evidence*):^2^

1 = Meets Criteria for Effectiveness

Achievable Results:

The following summary of achievable results is based on a published review of the scientific evidence.

On average, peer support groups for new mothers can achieve:

- Small, beneficial effects on exclusively breastfeeding for up to 3 months postpartum.^1^
- Small beneficial effects on mother confidence.^1^
- Effects are especially promising among socially disadvantaged mothers.^1^

On average, professional support (e.g., nurses, lactation consultants) can achieve:

- A small beneficial effect on the duration of breastfeeding (RR = 0.90, 95% CI = 0.82-0.97).^1^
- Face-to-face support can provide larger benefits (RR = 0.85, 95% CI = 0.74-0.97) than telephone contact (RR = 0.98, 95% CI = 0.88-1.09). ^1^
- There is no clear advantage between antenatal support as to only postnatal support.^1^
- Effects are limited among low-income populations (RR = 0.82, 95% CI = 0.62-1.08). ^1^

There is limited evidence that hospital policies, such as early breast feeding initiation, rooming-in, supplementary feedings, provision of hospital discharge packs (with and without formula), early hospital discharge, and intrapartum experience, influence initiation and continuation of breastfeeding. More research is needed.^1^

There is also limited evidence that workplace strategies are effective in supporting breastfeeding initiation and continuation. Such strategies may include maternal and paternal leave policies and tax incentives, public awareness of rights of women to work and breastfeed, cultural practices that are supportive of breastfeeding, community leader involvement, employer education, increasing awareness of national legislation protecting breastfeeding rights, and recognition of workplaces that are mother and baby friendly. More research is needed.^1^

Community Examples:

- Sonoma County, CA, Department of Health Services and the Sonoma County Breastfeeding Coalition promote breastfeeding-friendly policies, and provide breastfeeding education and support services.
  - <http://www.sonoma-county.org/health/ph/mcah/breastfeeding.htm>
  - <http://www.sonoma-county.org/health/ph/mcah/breastfeedingcoalition.htm>
- Texas Department of State Health Services promotes “mother-friendly” worksite program and provides a list of “mother-friendly” designated businesses.
  - <http://www.dshs.state.tx.us/wichd/lactate/mother-worksites.shtm#item4>

Links to Policy Examples^†^:

- California and Sonoma County Departments of Health Services maintain a maternal and child health program that provides breastfeeding support services. California also requires employers to provide paid rest periods for breastfeeding mothers^‡^.
  - §123255 of the California Health and Safety Code provides that the Department of Health Services may maintain a maternal and child health program in each county.
  - §123365 of the California Health and Safety Code provides that all general acute care hospitals and hospitals providing maternity care must make available a breast feeding consultant, or provide information to the mother on where to receive breast feeding services.
  - §1030 of the California Labor Code requires an employer to provide paid rest period for lactation purposes.
    - See pdfs for above policy examples
- Texas Statutes Health and Safety Code, §165.003 establishes the requirements for a business to use the designation “mother-friendly.”
  - <http://www.dshs.state.tx.us/wichd/lactate/mother.shtm#item1>
- Note: Twenty-four states, the District of Columbia, and Puerto Rico have laws related to breastfeeding in the workplace. <http://www.ncsl.org/IssuesResearch/Health/BreastfeedingLaws/tabid/14389/Default.aspx>

*Note: 1 = meets criteria for policy effectiveness (consistent, positive outcomes from at least two high-quality experimental or quasi-experimental trials using a comparison group or interrupted time series design)^2^; 2 = consistent evidence available linking policy with positive outcomes from high-quality observational studies only; 3 = insufficient evidence available for policy or policy components.

† Be sure to check with your state, county, and municipal governments regarding potential existing laws that may impede any new policy development.

‡Local governments and organizations may check existing state statutes and administrative codes for the authority to implement local policies.

References

^1^Dennis, CL (2002). Breastfeeding initiation and duration: A 1990-2000 literature review. *Journal of Obstetric, Gynecologic, & Neonatal Nursing*, 31, 12-32.

^2^Flay, BR, Biglan, A, Boruch, RF, Ganzalez Castro, F, Gottfredson, D, Kellam, S, Moscicki, EK, Schinke, S, Valentine, JC, & Ji, P (2005). Standards of evidence: Criteria for efficacy, effectiveness and dissemination. Prevention Science, 6(3), 151-175.

Child Placement when Taken from Home

Domain: Family Influences

Reasons for Policy:

- Over 500,000 children live in out-of-home care.^1^
- Mental health problems, maladaptive behaviors, school dropout, criminal involvement, homelessness, and dependency on welfare programs are common in these children.^1^
- Kinship care allows the child to live with people he/she all ready knows and trusts, which can positively affect the child’s wellbeing.^2^

Community Group:

- Local government
- Justice system
- Local social services departments

Policy Components:

- Placement with kin when appropriate accommodations are available
- Possible licensing standards for kin caregivers
- Additional financial resources and services for kin caregivers

Desired Outcomes:

- Improved behavioral development
- Improved mental health functioning
- Placement stability for children in out-of-home care

Level of Evidence Available to Evaluate Effectiveness of Policy (1 = strong evidence to 3 = insufficient evidence*):^3^

1 = Meets Criteria for Effectiveness

Achievable Results:

The following summary of achievable results is based on a published review of the scientific evidence.

On average, kinship care can:

- Achieve small reductions in behavior problems (Effect Size (*g*): -0.24)^2^
- Achieve moderate improvements in competence (Effect Size (*g*): 0.45)^2^
- Double the report of positive emotional health^2^

On average, children in non-kinship (e.g. foster) care were:

- 2.2 times more likely to experience mental illness^2^
- 2.6 times more likely to experience 3 or more placement settings^2^
- 1.7 times more likely to receive mental health services^2^

Kinship care may improve educational attainment and family relations, while non-kinship care may improve permanency. However, aggregate estimates of effect are not statistically significant.^2^

Community Stories:

- San Bernardino, CA
  - Kinship Guardianship Assistance Program
    - <http://hss.co.san-bernardino.ca.us/dcs/departmentinfo/New_Initiatives/Kinship/default.htm>
    - <http://www.co.san-bernardino.ca.us/budget0809/PDF/HSS/o_kinship_guardianship_assistance_program.pdf>
- Montgomery County, MA
  - Kinship care
    - <http://www.montgomerycountymd.gov/hhstmpl.asp?url=/content/hhs/ads/disabilityseniorservices/childrensenior.asp>

Policy Examples: †

- San Bernardino, CA
  - Human Services Group Established § 12.3501
    - Included the Department Of Child Support, Children, and Family Services which runs the Kinship Support Services Program
    - SEE PDF
- Montgomery, MD, Chapter 1, Section 1A-104
  - Sets up a position of Chief of Children, Youth, and Family Services within the Department of Health and Human Services
  - SEE PDF

*Note: 1 = meets criteria for policy effectiveness (consistent, positive outcomes from at least two high-quality experimental or quasi-experimental trials using a comparison group or interrupted time series design)^3^; 2 = consistent evidence available linking policy with positive outcomes from high-quality observational studies only; 3 = insufficient evidence available for policy or policy components.

† Be sure to check with your state, county, and municipal governments regarding potential existing laws that may impede any new policy development.

References

^1^Trout AL, Hagaman J, Casey K, Reid R, & Epstein MH (2008). The academic status of children and youth in out-of-home care: A review of the literature. *Children and Youth Services Review,* 30, 979-994.

^2^Winokur M, Holtan A, Valentine D. Kinship care for the safety, permanency, and well-being of children removed from the home for maltreatment. Cochrane Database of Systematic Reviews 2009, Issue 1. Art.No.: CD006546.

^3^Flay, BR, Biglan, A, Boruch, RF, Ganzalez Castro, F, Gottfredson, D, Kellam, S, Moscicki, EK, Schinke, S, Valentine, JC, & Ji, P (2005). Standards of evidence: Criteria for efficacy, effectiveness and dissemination. Prevention Science, 6(3), 151-175.

Home Safety Education

Domain: Family Influences

Reasons for Policy:

- Unintentional injuries are the leading cause of death among children aged 1 to 14 years.^1^
- For children under 5, the majority of injuries occur in the home; for children 5-9, 40% of injuries occur in the home; and for children 10-14, 25% of injuries occur in the home.^1^
- Children in families of lower socioeconomic status or young mothers are at a higher risk of injury than children of a higher socioeconomic status or older mothers.^1^

Community Group:

- State government
- Local government
- Local public health department
- Local non-profit organizations
- Local school district

Policy Components:

- Provision of free, low cost, or discounted safety equipment
- Home safety education taught in the home, schools, or health care settings
- Can be individual or group education

Desired Outcomes:

- Improved parenting skills
- Lower incidence of in-home injuries
- Lower incidence of deaths due to in-home injuries
- Lower incidence of childhood poisonings
- Improved usage of home safety equipment

Level of Evidence Available to Evaluate Effectiveness of Policy (1 = strong evidence to 3 = insufficient evidence*):^2^

1 = Meets Criteria for Effectiveness

Achievable Results:

The following summary of achievable results is based on a published review of the scientific evidence.

On average, home safety education and the provision of safety equipment can achieve:

- Moderate increase in the likelihood of having a safe hot tap water temperature (OR 1.34, 95% CI 1.00-1.80) evidence^1^
- Large increase in the likelihood of owning a functioning smoke alarm (OR 1.85, 95% CI 1.24-2.75) significant^1^
- Large improvements in proper storage of medicines (OR 1.58, 95% CI 1.18-2.13)^1^
- Large improvements in proper storage of cleaning products (OR 1.63, 95% CI 1.22-2.17)^1^
- Large improvements in possession of syrup of ipecac (OR 3.34, 95% CI 1.50-7.44)^1^
- Large improvements in poison control number accessibility (OR 3.66, 95% 1.84-7.27)^1^
- Small improvements in use of fitted stair gates (OR 1.26, 95% 1.05-1.51)^1^
- Large improvements in use of socket covers (OR 3.73, 95% CI 1.48-9.39)^1^
- Large improvements in proper storage of sharp objects (OR 1.52, 95% CI 1.01-2.29)^1^
- Beneficial effects tend to be larger for efforts that provide safety equipment than for those providing education alone.^1^
- There is limited evidence of effects on child injury rates. More research is needed that examines these outcomes specifically.^1^

Community Examples:

- Riverside County, California, Injury Prevention Services offers education services to parents and caregivers of young children. The program provides free safety supplies to county residents.
  - <http://www.rivcoips.org/home/index.php?option=com_content&view=article&id=32&Itemid=53>
  - <http://www.rivcoips.org/home/index.php?option=com_content&view=article&id=36&Itemid=59>
- Concord, North Carolina, Concord Fire Department offers educational presentations in home fire hazards and injury prevention. The Department provides opportunities for children to practice home fire education.
  - <http://www.ci.concord.nc.us/Departments/Fire/LifeSafetyEducation/tabid/110/Default.aspx>

Links to Policy Examples†:

- Riverside County Children and Families First Commission provides funding for Injury Prevention Services pursuant to Riverside County Municipal Code and California Health and Safety Code^‡^.
  - Riverside County Municipal Code Title 2, Chapter 2.48 Children and Families First Commission
    - See pdf
    - <http://library2.municode.com/default-now/home.htm?infobase=16320&doc_action=whatsnew>
  - California Health and Safety Code §130140
    - <http://codes.lp.findlaw.com/cacode/HSC/1/d108/s130140>
- Concord, North Carolina, Chapter 34 Fire Prevention And Protection, Article II Fire Department, §34-44 Duties of fire chief
  - <http://library1.municode.com/default-test/home.htm?infobase=10986&doc_action=whatsnew>

*Note: 1 = meets criteria for policy effectiveness (consistent, positive outcomes from at least two high-quality experimental or quasi-experimental trials using a comparison group or interrupted time series design)^2^; 2 = consistent evidence available linking policy with positive outcomes from high-quality observational studies only; 3 = insufficient evidence available for policy or policy components.

† Be sure to check with your state, county, and municipal governments regarding potential existing laws that may impede any new policy development.

‡Local governments and organizations may check existing state statutes and administrative codes for the authority to implement local policies.

References

^1^Kendrick D, Coupland C, Mulvaney C, Simpson J, Smith S, Sutton A, Watson M. Home safety education and provision of safety equipment for injury prevention. Cochrane Database of Systematic Reviews 2007, Issue 1. Art. No.: CD005014.

^2^Flay, BR, Biglan, A, Boruch, RF, Ganzalez Castro, F, Gottfredson, D, Kellam, S, Moscicki, EK, Schinke, S, Valentine, JC, & Ji, P (2005). Standards of evidence: Criteria for efficacy, effectiveness and dissemination. Prevention Science, 6(3), 151-175.

Home Visiting Programs

Domain: Family Influences

Reasons for Policy:

- Socially disadvantaged children are more likely to be exposed to multiple stressors in the home such as, noisy and poor quality homes, chaotic family life, have fewer books, and less stimulating home environments.^1^
- Socially disadvantaged parents are more likely to have mental health problems, less consistent, less stimulating, and more punitive parenting behaviors, less likely to show affection to their children, and more likely to feel like they have less social support.^1^
- These social and developmental deficits have been shown to have effects on children throughout their lifetime.^1^

Community Group:

- Local government
- Local public health department
- Non-profits/community organizations

Policy Components:

- Frequent visits (e.g. weekly or bi-weekly)
- Provide age appropriate information about child development and care
- Provide age appropriate resources (e.g. books, puzzles, art materials)
- Provide general support about parenting or child development to the parent

Desired Outcomes:

- Greater maternal sensitivity
- Create and maintain an academically stimulating home
- Improved cognitive development of children
- Improved parent/child relationship
- Reduction in home stressors and punitive parenting behaviors

Level of Evidence Available to Evaluate Effectiveness of Policy (1 = strong evidence to 3 = insufficient evidence*):

1 = Meets Criteria for Effectiveness

Achievable Results:

The following summary of achievable results is based on a published review of the scientific evidence.

- On average, home visitation programs can achieve small improvement in parenting behaviors and attitudes (effect sizes 0.11-0.14).^2,4^
- Small improvements in child cognitive and socio-emotional development (effect sizes 0.10-0.18)^4^
- Small improvements in risks for potential child abuse (effect size 0.24)^4^

Community Examples:

- The Best Beginnings Nurse Family Partnership in Seattle, Wasington provide home visiting to low income, first time pregnant women.
  - <http://www.seattle.gov/humanservices/foodhealth/publichealth/pregnant_adolescent.htm>
- Help Us Grow Successfully (HUGS) program in Nashville, Tennessee is a home visiting program for new mothers.
  - <http://health.nashville.gov/CH/ch_help_us.htm>

Links to Policy Examples: †

- Seattle, WA, Ordinance 120732, Council Bill 114093
  - <http://clerk.ci.seattle.wa.us/~scripts/nph-brs.exe?s1=&s3=&s4=&s2=&s5=pregnant&Sect4=AND&l=20&Sect2=THESON&Sect3=PLURON&Sect5=CBORY&Sect6=HITOFF&d=ORDF&p=1&u=%2F~public%2Fcbory.htm&r=8&f=G>
- Nashville, TN
  - Resolution detailing a grant from the state to begin a home visitation program
    - <http://www.nashville.gov/mc/resolutions/term_2007_2011/rs2009_927.htm>
  - Resolution detailing a grant from the state to begin a HUGS program in the area
    - <http://www.nashville.gov/mc/resolutions/term_2007_2011/rs2009_936.htm>
  - Resolution RS2002-996 approving annual grant for home visitation counseling
    - <http://www.nashville.gov/council/docs/analysis/020402.pdf>

*Note: 1 = meets criteria for policy effectiveness (consistent, positive outcomes from at least two high-quality experimental or quasi-experimental trials using a comparison group or interrupted time series design)^3^; 2 = consistent evidence available linking policy with positive outcomes from high-quality observational studies only; 3 = insufficient evidence available for policy or policy components.

† Be sure to check with your state, county, and municipal governments regarding potential existing laws that may impede any new policy development.

References

^1^Miller S, Eakin A. Home based child development interventions for pre-school children

from socially disadvantaged families. Cochrane Database of Systematic Reviews 2009, Issue 4. Art. No.: CD008131.

^2^Nievar, M. A., & Van Egeren, L. (2005). More is better: A meta-analysis of home visiting programs for at-risk families. *Online Submission, Paper presented at the Biennial Conference of the Society for Research in Child Development (SRCD) (Tampa, FL, Apr 24-27, 2003).* Retrieved from www.eric.ed.gov.

^3^Flay, BR, Biglan, A, Boruch, RF, Ganzalez Castro, F, Gottfredson, D, Kellam, S, Moscicki, EK, Schinke, S, Valentine, JC, & Ji, P (2005). Standards of evidence: Criteria for efficacy, effectiveness and dissemination. Prevention Science, 6(3), 151-175.

^4^Sweet, M.A. & Appelbaum, M.I. (2004). Is home visiting an effective strategy? A meta-analytic review of home visiting programs for families with young children. Child Development, 15(5), 1435-1456.

Parent Involvement in Child’s Education

Domain: Family Influences

Reasons for Policy:

- Parent involvement has a positive effect on children’s academic performance.^1^

Community Group:

- School district/local school board
- Parent-Teacher Association
- Local government
- State government

Policy Components:

- Active parent engagement with children outside of the school day
- Parent involvement with children in academic activities to support school-based academic instruction
- Program implementation in which the parent has a direct interaction with the child or direct monitoring of the child

Desired Outcomes:

- Parent involvement in learning-related activities
- Improvement in academic performance
- Prevention of child behavior problems
- Promotion of pro-social norms

Level of Evidence Available to Evaluate Effectiveness of Policy (1 = strong evidence to 3 = insufficient evidence*):^2^

1 = Meets Criteria for Effectiveness

Achievable Results:

The following summary of achievable results is based on a published review of the scientific evidence.

On average, parent involvement in academic activities can achieve:

- Moderate improvements in academic performance among elementary school-aged children (d=0.45, 95% CI=0.025-0.66)^1^
- The greatest benefits occurred in reading (Medium Effect Size, d=0.42, 95% CI=0.18-0.66)^1^

Community Examples:

- Union Public Schools, Oklahoma PTA organization provides multiple ways for parents to become involved in their child’s academic achievement
  - <http://www.unionps.org/index.cfm?id=371>
- Audubon Charter School, Louisiana parental involvement webpage
  - <http://www.auduboncharter.com/parentinfo.htm>

Links to Policy Examples†:

- Union Public School Code details appropriate booster club and PTA guidelines
  - <http://www.unionps.org/filesBoardBook/1005_Booster_Club_PTA_Sanctioning2.pdf>
- Audubon Charter School policy for increasing parental involvement
  - <http://www.auduboncharter.com/documents/ACS%20School-Level%20Parental%20Involvement%20Policy%2009-10.pdf>

*Note: 1 = meets criteria for policy effectiveness (consistent, positive outcomes from at least two high-quality experimental or quasi-experimental trials using a comparison group or interrupted time series design)^2^; 2 = consistent evidence available linking policy with positive outcomes from high-quality observational studies only; 3 = insufficient evidence available for policy or policy components.

† Be sure to check with your state, county, and municipal governments regarding potential existing laws that may impede any new policy development.

References

1 Nye C, Turner H, Schwartz J. Approaches to parent involvement for improving the academic performance of elementary school age children. Campbell Systematic Reviews 2006:4.

2 Flay, BR, Biglan, A, Boruch, RF, Ganzalez Castro, F, Gottfredson, D, Kellam, S, Moscicki, EK, Schinke, S, Valentine, JC, & Ji, P (2005). Standards of evidence: Criteria for efficacy, effectiveness and dissemination. Prevention Science, 6(3), 151-175.

Prenatal Micronutrient Supplementation

Domain: Family Influences

Reasons for Policy:

- Prenatal nutrition plays an important role in the growth and development of the fetus^1^
- Overall, the diet of pregnant women is deficient in calories and micronutrients^1^
- In the United States, over 350,000 babies (8.3% of all births) a year are born with low birth weight^2^

Community Group:

- Local health department
- State health department
- State government
- Federal Government

Policy Components:

- Providing multi-micronutrient supplementation for pregnant women

Desired Outcomes:

- Reduction of low birth weight infants
- Increase in birth weight among infants

Level of Evidence Available to Evaluate Effectiveness of Policy (1 = strong evidence to 3 = insufficient evidence*):^3^

1 = Meets Criteria for Effectiveness

Achievable Results:

The following summary of achievable results is based on a published review of the scientific evidence.

On average, prenatal multi-micronutrient supplementation can achieve:

- 17% reduction in low birth weight infants.^1^
- 54 gram higher birth weight among infants whose mothers were given micronutrients compared to those whose mothers were given iron-folic acid supplementation.^1^

Note: Prenatal micronutrient supplementation programs are commonly employed by NGO’s in developing countries.

Community Examples:

- USAID supports micronutrient supplementation interventions in developing countries in South Asia, and Africa.
  - <http://www.usaid.gov/our_work/global_health/nut/techareas/micro.html>
- Micronutrient Initiative, an international not-for-profit organization, will provide pregnant women in sub-Saharan Africa with iron supplements. The intervention is funded by the Canadian International Development Agency.
  - <http://www.micronutrient.org/English/view.asp?x=656&id=43>

*Note: 1 = meets criteria for policy effectiveness (consistent, positive outcomes from at least two high-quality experimental or quasi-experimental trials using a comparison group or interrupted time series design)^3^; 2 = consistent evidence available linking policy with positive outcomes from high-quality observational studies only; 3 = insufficient evidence available for policy or policy components.

† Be sure to check with your state, county, and municipal governments regarding potential existing laws that may impede any new policy development.

References

1 Prakesh SS, Ohlsson A (2009). Effects of prenatal multimicronutrient supplementation on pregnancy outcomes: a meta-analysis. Canadian Medical Association Journal. 180(12), 99-108.

2 Martin JA, et al. (2006). Births: Final Data for 2006. National Vital Statistics Reports, National Center for Health Statistics, 57(7), retrieved from, <http://www.cdc.gov/nchs/fastats/birthwt.htm>.

3 Flay, BR, Biglan, A, Boruch, RF, Ganzalez Castro, F, Gottfredson, D, Kellam, S, Moscicki, EK, Schinke, S, Valentine, JC, & Ji, P (2005). Standards of evidence: Criteria for efficacy, effectiveness and dissemination. Prevention Science, 6(3), 151-175.

Treatment Foster Care

Domain: Family Influences

Reasons for Policy:

- Treatment foster care (TFC) is a foster family-based intervention that aims to provide young people and families with an individually tailored program designed to help bring about positive change in their lives.^1^
- TFC may be a useful intervention for children with complex emotional, psychological, and behavioral need, who are at risk of placements in non-family settings that restrict their liberty and opportunities for social inclusion.^1^
- Compared to youth discharged from institutional care, youth discharged from TFC are more likely to go to less restrictive settings and are less likely to be subsequently institutionalized.^1^
- Comparatively, TFC programs may be less costly than institutional and group home alternatives.^1^

Community Group:

- Non-profit organizations
- State child services department
- State government

Policy Components:

- Any Treatment Foster Care program that provides individualized, therapeutic, community and foster family-based intensive services to children and adolescents that includes:
  - Foster carers trained to provide therapeutic care to youths who have special needs (emotional, development, or medical).
  - Care provided in a family setting, in a home controlled by foster carers.
  - Number of children placed in a home is ≤ 2.
  - Foster carers receive support, consultation, and supervision from professionals with crisis intervention services available.
  - Foster carers are regarded as professional members of the service.
  - Program is administered by specialist agencies or unit identified as a TFC.

Desired Outcomes:

- Positive change in behavioral outcomes (i.e., reduced anti-social behavior, rates of delinquency, rates of arrest, rates or conviction, rates of incarceration, reduced drug and substance abuse, increased compliance with medication, treatment, symptom management.)
- Decreased risk of institutional placement
- Prevent multiple placements (placement stability)
- Positive psychological functioning, mental health status
- Positive interpersonal functioning
- Positive educational outcomes
- Improved physical health

Level of Evidence Available to Evaluate Effectiveness of Policy (1 = strong evidence to 3 = insufficient evidence*):^2^

1 = Meets Criteria for Effectiveness

Achievable Results:

The following summary of achievable results is based on a published review of the scientific evidence.

On average, treatment foster care can achieve:

- Improvements in school attendance and homework completion^1^
- Decreases in antisocial behavior, the number of days children abscond from placement, the number of criminal referrals, and time spent in locked settings.^1^

Community Examples:

- Washington State Department of Social and Health Services oversees the Behavior Rehabilitation Services (BRS) an intensive support and treatment program for children with behavioral/emotional disturbances as well as developmental disabilities, designed to assist them in transitioning to a less restrictive environment.
  - <http://www.dshs.wa.gov/ca/fosterparents/be_FosterIntro.asp>
  - see BRS handbook pdf
- Alabama MENTOR is a home and community-based services provider that provides a therapeutic foster care program. Youth are referred to the program from the Alabama Department of Human Resources.
  - <http://www.al-mentor.com/standard/services.aspx?guid=728b2af1-23e8-478a-afdb-cfe4bc88c273>

Links to Policy Examples:

- Revised Code of Washington, Chapter 74.13 Child Welfare Services
  - Washington Administrative Code, Chapter 388-25 Child welfare services-foster care governs foster care
- Alabama Administrative Code, Chapter 660-5-28 Foster Care for Children
  - See also Alabama Department of Human Resources, Therapeutic Foster Care manual pdf.

*Note: 1 = meets criteria for policy effectiveness (consistent, positive outcomes from at least two high-quality experimental or quasi-experimental trials using a comparison group or interrupted time series design)^2^; 2 = consistent evidence available linking policy with positive outcomes from high-quality observational studies only; 3 = insufficient evidence available for policy or policy components.

† Be sure to check with your state, county, and municipal governments regarding potential existing laws that may impede any new policy development.

‡Local governments and organizations may check existing state and federal statutes and administrative codes for the authority to implement local policies.

References

1MacDonald, GM, Turner, W (2007). Treatment foster care for improving outcomes in children and young people. Campbell Systematic Reviews 2007, 9, DOI:10.4073/csr.2007.9.

2 Flay, BR, Biglan, A, Boruch, RF, Ganzalez Castro, F, Gottfredson, D, Kellam, S, Moscicki, EK, Schinke, S, Valentine, JC, & Ji, P (2005). Standards of evidence: Criteria for efficacy, effectiveness and dissemination. Prevention Science, 6(3), 151-175.

After-School Programs that Include Academic Support Services

Domain: School Influences

Reasons for Policy:

- More than 7 million children in the US are without adult supervision after school.^1^
- In comparison with middle-income children, low-income children are more in need of after-school opportunities and more likely to benefit from them.^2^
- Unstructured, unsupervised after-school time is associated with increased violence, delinquency, sexual intercourse, smoking, alcohol and drug use, and poor academic outcomes.^3^

Community Group:

- Local School Board
- Local Government
- Community-based organizations
- Universities

Policy Components:

- Provide positive adult supervision while offering academic, youth development, and recreational activities
- Employ evidence-based training methods for the program that incorporate a planned set of activities which use active forms of learning to target positive youth development
- Involve partnerships between community-based organizations (and/or universities) and schools

Desired Outcomes:

- Reduced maladaptive behaviors such as crime and drug abuse
- Provide enriching experiences that can improve children’s socialization
- Enhanced academic achievement

Level of Evidence Available to Evaluate Effectiveness of Policy (1 = strong evidence to 3 = insufficient evidence*):

1 = Meets Criteria for Effectiveness

Achievable Results:

The following summary of achievable results is based on a published review of the scientific evidence.^4^

Significant improvements have been observed for:

- Reading achievement among students at risk for school failure (small effect size of 0.07)^5^
- Mathematics achievement among students at risk for school failure (moderate effect size of 0.26)^5^

Community Examples:

- Berkeley, CA Unified School District offers multiple after school programs
  - <http://www.berkeley.net/after-school-programs/>
- Broward, Florida host before and after school programs for elementary and middle school children.
  - <http://www.broward.k12.fl.us/k12programs/bascc/>

Links to Policy Examples†:

- Berkeley, CA Unified School District Policy details supplemental instruction for students
  - See PDF
- Broward, FL, School board policy 6000.3 Before and After Care Student Programs outlines the rules for providers
  - See PDF

*Note: 1 = meets criteria for policy effectiveness (consistent, positive outcomes from at least two high-quality experimental or quasi-experimental trials using a comparison group or interrupted time series design)^4^; 2 = consistent evidence available linking policy with positive outcomes from high-quality observational studies only; 3 = insufficient evidence available for policy or policy components.

† Be sure to check with your state, county, and municipal governments regarding potential existing laws that may impede any new policy development.

References

^1^ Durlak, JA & Weissberg, RP (2007). The impact of after-school programs that seek to promote personal and social skills. Chicago, IL: The Collaborative for Academic, Social and Emotional Learning.

^2^ Miller, BM (2003). Critical hours: Afterschool programs and educational success. Quincy, MA: Mellie Mae Education Foundation. Retrieved November 10, 2011, from <http://www.nmefdn.org/CriticalHours.htm>

- - ^3^ U.S. Department of Education & U.S. Department of Justice (2000). Working for children and families: Safe and smart after-school programs. Washington, DC: Government Printing Office. Retrieved from <http://www.ed.gov>.

^4^ Flay, BR, Biglan, A, Boruch, RF, Ganzalez Castro, F, Gottfredson, D, Kellam, S, Moscicki, EK, Schinke, S, Valentine, JC, & Ji, P (2005). Standards of evidence: Criteria for efficacy, effectiveness and dissemination. Prevention Science, 6(3), 151-175.

^5^ Lauer, PA, Akiba, M, Wilderson, SB, Apthorp, HS, Snow, D, & Martin-Glenn, ML (2006). Out-of-school-time programs: A meta-analysis of effects for at-risk students. Review of Educational Research, 76(2), 275-313.

Class Size Reductions

Domain: School Influences

Reasons for Policy:

- Smaller class sizes could positively affect student’s academic performance, especially for disadvantaged or minority students.^1^
- Individualized attention in the elementary school years can have a positive effect on students that persists throughout their schooling.^1^

Community Group:

- State government
- Local government
- Local school district

Policy Components:

- Class sizes of 17-20 students in the elementary school years, especially grades K-3
- Consideration of ideal or reasonable class sizes for each school district
- Use in conjunction with other school policy reforms

Desired Outcomes:

- Individualized attention for each student
- Improved academic performance
- Improved attitude towards school

Level of Evidence Available to Evaluate Effectiveness of Policy (1 = strong evidence to 3 = insufficient evidence*):^2^

1 = Meets Criteria for Effectiveness

Achievable Results:

The following summary of achievable results is based on a published review of the scientific evidence.

On average, a smaller class size can achieve:

- Small improvements in students’ academic performance (Effect Size: 0.20).^1^

Community Examples:

- St. Louis Missouri Ladue School District has a small class policy that mandates the class size meets or is lower than the state standards
  - <http://beta.ladue.k12.mo.us/district/content/our-district/class-size-policies.shtml>
- Bonneville Joint School District (Idaho) implements a small class size policy to facilitate student achievement.
  - http://www3.d93.k12.id.us/k-12-resources.aspx

Links to Policy Examples: †

- St. Louis Missouri Ladue School District sets a desirable standard of 20 students for K-2 grades, 22 for grades 3-4, 25 students in grades 5-6, and 28 students in grades 7-12
  - See PDF
- Bonneville Joint School District: #2240
  - http://www3.d93.k12.id.us/media/CMSImport/6AD6B57E1C1E44E18D2C9AD928AC76C4.pdf

*Note: 1 = meets criteria for policy effectiveness (consistent, positive outcomes from at least two high-quality experimental or quasi-experimental trials using a comparison group or interrupted time series design)^2^; 2 = consistent evidence available linking policy with positive outcomes from high-quality observational studies only; 3 = insufficient evidence available for policy or policy components.

† Be sure to check with your state, county, and municipal governments regarding potential existing laws that may impede any new policy development.

References

^1^Shin, IS, Chung JY (2009). Class size and student achievement in the United States: A meta-analysis. *KEDI Journal of Educational Policy,* 6(2), 3-19.

^2^Flay, BR, Biglan, A, Boruch, RF, Ganzalez Castro, F, Gottfredson, D, Kellam, S, Moscicki, EK, Schinke, S, Valentine, JC, & Ji, P (2005). Standards of evidence: Criteria for efficacy, effectiveness and dissemination. Prevention Science, 6(3), 151-175.

Positive Behavior Support

Domain: School Influences

Reasons for Policy:

- Zero-tolerance policies may actually increase the incidence and severity of aggressive and delinquent behaviors.^2^
- Positive behavior support (PBS) is meant to stop a problem behavior before it occurs or escalates and teach appropriate alternatives.^2^

Community Group:

- Local government
- Local school district
- Schools

Policy Components:

- Have a multidisciplinary team creating strategies (e.g. teachers, administrators, and special service personnel)
- Design strategies targeting behavioral problems individual schools are having
- Train teachers in use of positive behavioral support
- Use applied behavioral analysis techniques to modify problem behaviors (e.g. token economies, positive reinforcement, active supervision, precorrective strategies)

Desired Outcomes:

- Improved school environment
- Reduced criminal behavior
- Reduced drug use
- Higher rates of positive behavior
- Improved performance in school
- Reduced special education placement

Level of Evidence Available to Evaluate Effectiveness of Policy (1 = strong evidence to 3 = insufficient evidence*)^3^:

1 = Meets Criteria for Effectiveness

Achievable Results:

The following summary of achievable results is based on a published review of the scientific evidence.

On average, behavior modification in schools can achieve:

- Small effects on alcohol and other drug use (d=0.04, 95% CI: 0.02-0.07)^1^
- Small effects on school dropout/truancy (d=0.13, 95% CI: 0.06-0.20)^1^
- Small effects on other problem behaviors (e.g., rebellious, antisocial or disrespectful behaviors (d=0.15, 95% CI: 0.10-0.19)^1^

Community Examples:

- East Baton Rouge Parish School, Westdale Middle, uses positive behavior support
  - <http://westdalemiddle.ebrschools.org/explore.cfm/positivebehavior/>
- Austin Independent School District, Texas, incorporates positive behavior support into its schools
  - <http://www.austinisd.org/academics/sss/pbs/model.phtml>

Links to Policy Examples†:

- East Baton Rouge Parish School System’s policy manual uses “positive intervention and behavior modification” for discipline
  - <http://www.ebrschools.org/eduWEB1/1000437/docs/policy_manual_japp101608.pdf> (use ctrl+F to find “positive,” it is the second highlighted portion, page 76)
- Austin Independent School District, Texas, school board policy manual includes positive behavior support system for disciplinary action as well as the option for disciplinary alternative education program operations (e.g. positive behavior support)
  - See pdf

*Note: 1 = meets criteria for policy effectiveness (consistent, positive outcomes from at least two high-quality experimental or quasi-experimental trials using a comparison group or interrupted time series design)^3^; 2 = consistent evidence available linking policy with positive outcomes from high-quality observational studies only; 3 = insufficient evidence available for policy or policy components.

† Be sure to check with your state, county, and municipal governments regarding potential existing laws that may impede any new policy development.

References

^1^Wilson DB, Gottfredson DC, Najaka SS (2001). School-based prevention of problem behaviors: A meta-analysis. *Journal of Quantitative Criminology,* 17(3), 247-272.

^2^Safran SP, Oswald K (2003). Positive behavior supports: Can schools reshape disciplinary practices? *Exceptional Children,* 69(3), 361-373.

^3^Flay, BR, Biglan, A, Boruch, RF, Ganzalez Castro, F, Gottfredson, D, Kellam, S, Moscicki, EK, Schinke, S, Valentine, JC, & Ji, P (2005). Standards of evidence: Criteria for efficacy, effectiveness and dissemination. Prevention Science, 6(3), 151-175.

Quality Preschool/Early Childhood Education

Domain: School Influences

Reasons for Policy:

- Improved cognitive abilities among children.^1^
- Provide foundation for later learning and well-being^2^
- Decrease achievement gaps across race/ethnicity and socioeconomic status^2^

Community Group:

- State government
- Local government
- School district

Policy Components:

- “Center-based” programs (e.g. in a school or child development center)
- Provide an alternative physical or social environment compared to the home
- Possible home visitation component
- Full or half day programs, 9-12 months out of the year

Desired Outcomes:

- Higher rates of school readiness, IQ scores, and academic achievement
- Lower rates of grade retention and special education placement
- Lower rates of delinquency, teenage pregnancy, and welfare use
- Higher rates of high school graduation

Level of Evidence Available to Evaluate Effectiveness of Policy (1 = strong evidence to 3 = insufficient evidence*)^3^:

1 = Meets Criteria for Effectiveness

Achievable Results:

The following summary of achievable results is based on a published review of the scientific evidence.

- Moderate improvements in cognitive achievement (effect size 0.23), including academic (effect size 0.35), school readiness (effect size 0.38), and IQ test scores (effect size 0.43)^2, 4^
- Small improvements in school progress (effect size 0.14), including 13 to 14 percentage point reductions in grade retention and special education^2, 4^
- Small improvements (effect size 0.16) in socio/emotional and anti-social health^4^
- 17 to 27 percentage point increases in positive social outcomes, including high school graduation, employment and home ownership^2^
- 14 to 49 percentage point reductions in negative social outcomes, including teen pregnancy, teen arrests and welfare use^2^
- 44 to 61 percentage point increases in child health screenings^2^
- 3 to 22 percentage point increases in positive family outcomes, including parental high school graduation, income above the poverty line, parental employment, and not receiving public assistance^2^

Community Examples:

- Denver, Colorado, Denver Preschool Program provides tuition credits for parents to send their children to preschool and grants to improve existing preschools
  - <http://www.dpp.org/>
  - <http://earlyeducation.dpsk12.org/>
- El Dorado, California, First 5 program and Early Head Start
  - <http://www.edcoe.org/departments/child_development/early_head_start.html>
  - http://www.first5eldorado.com/Projects.html

Links to Policy Examples†:

- Denver, Colorado, preschool program is identified in this piece of legislation from the state
  - <http://www.cde.state.co.us/cpp/CPPHandbookOnline/programreq2.htm>
  - City Ordinance Chapter 11, Article III. Denver Preschool Program
    - See pdf
    - <http://library.municode.com/index.aspx?clientId=10257&stateId=6&stateName=Colorado> (There is not a link directly to the code, but I found the link in the municode database)
- El Dorado, California Municipal Code Chapter 8.65
  - See pdf in file
  - <http://www.sterlingcodifiers.com/CA/El%20Dorado%20County/index.htm>

*Note: 1 = meets criteria for policy effectiveness (consistent, positive outcomes from at least two high-quality experimental or quasi-experimental trials using a comparison group or interrupted time series design)^3^; 2 = consistent evidence available linking policy with positive outcomes from high-quality observational studies only; 3 = insufficient evidence available for policy or policy components.

† Be sure to check with your state, county, and municipal governments regarding potential existing laws that may impede any new policy development.

References

^1^ Gorey, KM (2001). Early childhood education: A meta-analytic affirmation of the short- and long-term benefits of educational opportunity. School Psychology Quarterly, 16(1), 9-30.

^2^ Anderson, LM, et al. (2003). The effectiveness of early childhood development programs: A systematic review. American Journal of Preventive Medicine, 24(3S), 32-46.

^3^ Flay, BR, Biglan, A, Boruch, RF, Ganzalez Castro, F, Gottfredson, D, Kellam, S, Moscicki, EK, Schinke, S, Valentine, JC, & Ji, P (2005). Standards of evidence: Criteria for efficacy, effectiveness and dissemination. Prevention Science, 6(3), 151-175.

^4^Camilli, G., Vargas, S., Ryan, S. & Barnett, W.S. (2010). Meta-analysis of the effects of early education interventions on cognitive and social development. Teachers College Record, 112(3), 579-620.

School-Based Physical Activity Programs

Domain: School Influences

Reasons for Policy:

- Childhood obesity is a major public health problem in the United States,^1^ and its prevalence has more than tripled since 1970.^2^
- These trends are likely to result in significant increases in the rates of heart disease, diabetes, and other diseases in young and middle-aged adults^3^ which could lead to a decline in life expectancy in the developed world.^4^
- Physical inactivity and obesity are shown to disproportionately impact low SES and minority children,^2^ and evidence suggests that school-based interventions are the best approach to reach this population.^5^
- Health and behavioral benefits are seen in children and adolescents who participate in 60 minutes or more of moderate to vigorous physical activity on a daily basis.^6^

Community Group:

- Local School District
- Local Government
- Parent Teacher Organization

Policy Components:

- Target kindergarten through 8^th^ grade students^7^
- Provide minimum of 30 minutes of daily moderate to vigorous physical activity (MVPA) during the school day via physical education, activity breaks, and recess^7^
- Teach skills to increase MVPA or reduce sedentary behavior^7^

Desired Outcomes:

- Increased physical activity for students
- Improved academic performance
- Improved mental health
- Improved fitness and physical health of students
- Establish lifelong patterns of healthy behavior
- Reduced BMI

Level of Evidence Available to Evaluate Effectiveness of Policy (1 = strong evidence to 3 = insufficient evidence*)^8^:

1 = Meets Criteria for Effectiveness

Achievable Results:

The following summary of achievable results is based on a published review of the scientific evidence.

On average school-based physical activity programs can achieve:

- 4.4% reductions in body fat (via skin-fold measurement)^9^
- 50.3% increase in class time spent in MVPA^9^
- 8.4% increase in aerobic capacity^9^
- Improved muscular endurance, flexibility, health related knowledge, and physical activity self-efficacy.^9^
- Small positive gains in academic performance^6^

Average improvements in BMI were not significant.^10^

Community Examples:

- Montgomery County, Maryland, Montgomery County Public Schools requires a Pre K– 12 physical education curriculum.
  - <http://www.montgomeryschoolsmd.org/curriculum/physed/>
- Charlotte-Mecklenburg School District, North Carolina requires elementary students to participate in physical activity for 30 minutes a day
  - <http://www.cms.k12.nc.us/cmsdepartments/ci/health-phys-ed/Pages/HealthyActiveChildren.aspx>
  - <http://www.cms.k12.nc.us/cmsdepartments/ci/health-phys-ed/Pages/default.aspx>

Links to Policy Examples†:

- Montgomery County, Maryland, Board of Education sets policy and regulations for Pre K- 12 physical education based on state Code of Maryland Regulations Requirements for Physical Education Instructional Programs for Grades K-12.
  - <http://www.montgomeryschoolsmd.org/departments/policy/pdf/jpg.pdf>
  - <http://www.montgomeryschoolsmd.org/departments/policy/pdf/jpgra.pdf>
  - <http://www.montgomeryschoolsmd.org/uploadedFiles/curriculum/physed/elementary/COMAR%20Requirements%20for%20PE%20K_12.pdf>
  - See pdfs
- Charlotte-Mecklenburg School District, North Carolina follows the state Department of Education policy (NC SBE Policy ID Number HSP-S-000) which requires 30 minutes of physical activity participation for elementary students daily
  - <http://www.ncpublicschools.org/docs/curriculum/healthfulliving/resources/policy/healthychildren/sbepolicymanual.pdf>
  - [http://policy.microscribepub.com/cgi-bin/om_isapi.dll?clientID=208862495&depth=8&infobase=charmeck.nfo&record={1C46}&softpage=PL_frame](http://policy.microscribepub.com/cgi-bin/om_isapi.dll?clientID=208862495&depth=8&infobase=charmeck.nfo&record=%7b1C46%7d&softpage=PL_frame)

*Note: 1 = meets criteria for policy effectiveness (consistent, positive outcomes from at least two high-quality experimental or quasi-experimental trials using a comparison group or interrupted time series design)^8^; 2 = consistent evidence available linking policy with positive outcomes from high-quality observational studies only; 3 = insufficient evidence available for policy or policy components.

† Be sure to check with your state, county, and municipal governments regarding potential existing laws that may impede any new policy development.

References

^1^Reilly JJ, Nethven, E, McDowell, ZC, et al. (2003). Health consequences of obesity. Archives of disease in childhood, 88, 748-752.

^2^ Ogden, CL, Carroll MD, Curtin LR, et al. (2006). Prevalence of overweight and obesity in the United States, 1999-2004. JAMA 295, 1549-1555.

^3^ Hayman, LL, Williams, CL, Daniels, SR, Steinberger, J, Paridon, S, Dennison, BA, McCrindle, BW (2004). Cardiovascular health promotion in the schools: A statement for health and education professionals and child health advocates from the committee on atherosclerosis, hypertension, and obesity in youth (AHOY) of the Council on Cardiovascular Disease in the Young, American Heart Association. Circulation, 110, 2266-2275.

^4^ Olshansky, SJ, Passaro, DJ, Hershow, RC, et al. (2005). A potential decline in life expectancy in the United States in the 21^st^ century. New England Journal of Medicine, 352, 1138-1145.

^5^ Gearhart, RF, Gruber, DM, & Vanata, DF. Obesity in the lower socio-economic status segments of American society. Forum on Public Policy, retrieved on February 18, 2010, from: <http://static.ashland.edu/academics/arts_sci/fcs//documents/oxfordarticle.pdf>.

^6^ Strong, WB, Malina, RM, Bumkie, CJR, et al. (2005). Evidence based physical activity for school-age youth. Journal of Pediatrics, 146: 732-737.

^7^ Centers for Disease Control and Prevention. Guidelines for school and community programs to promote lifelong physical activity among young people. MMWR Recomm Rep 1997 Mar 7;46(RR-6):1-36.

^8^ Flay, BR, Biglan, A, Boruch, RF, Ganzalez Castro, F, Gottfredson, D, Kellam, S, Moscicki, EK, Schinke, S, Valentine, JC, & Ji, P (2005). Standards of evidence: Criteria for efficacy, effectiveness and dissemination. Prevention Science, 6(3), 151-175.

^9^Kahn, EB, Ramsey, LT, Brownson, RC, et al. (2002). The effectiveness of interventions to increase physical activity: A systematic review. American Journal of Preventive Medicine, 22(4s), 73-107.

^10^Harris, KC, Kuramoto, LK, Schulzer, M & Retallack, JE (2009). Effect of school-based physical activity interventions on body mass index in children: a meta-analysis. Canadian Medical Association Journal, 180(7), 719-726.

School Nutrition Standards for School Lunch Programs

Domain: School Influences

Reasons for Policy:

- During the past 30 years childhood obesity rates have more than tripled and are disproportionately prevalent in low-income and ethnic minority children.^1,2^
- The school environment has the broad potential to impact students’ food choices and dietary quality, yet many school lunches do not meet nutrition standards.^3,4^

Community Group:

- School District
- Local Government

Policy Components:

- Nutrition standards for menu planning among school meal programs or at other meals/snacks sold in school environment consistent with the National Dietary Guidelines, CDC guidelines for school health program to promote lifelong healthy eating, or 5-A-Day program
- Implementation and compliance with nutrition guidelines in school lunch program
- Increased availability of fruit and vegetables and providing more lower fat school meals and snacks

Desired Outcomes:

- Improve the school food environment
- Improve student dietary intake
- Reduce rates of overweight and obesity in students

Level of Evidence Available to Evaluate Effectiveness of Policy (1 = strong evidence to 3 = insufficient evidence*):

1 = Meets Criteria for Effectiveness

Achievable Results:

The following summary of achievable results is based on a published review of the scientific evidence.

On average, school food and nutrition guidelines can achieve:

- Increased fruit and vegetable availability, with increases ranging from +0.28 servings/day to +0.48 servings/day^6^
- Significant decreases in total fat, with increases ranging from -2.0% to -10.9% of energy^6^
- Significant decreases in saturated fat intake, with increases ranging from -0.9% to -5.2% of energy^6^
- Positive impact on fruit and vegetable intake, with increases ranging from +0.30 to +0.37 servings/day^6^
- Significant decrease in total and saturated fat on the school menus (magnitude of effect unknown)^6^

There is insufficient scientific evidence available to determine impacts on body mass index (BMI).

Community Stories:

- Juneau School District (Alaska), provides a food service program that prescribes to the National and State nutritional guidelines.
  - <http://www.juneauschools.org/district/administrative_services/food_service_program>
- Beaver Dam Unified School District (Wisconsin) provides school lunches that comply with the 5-A-Day program and aims to educate its students on the importance of nutritional value.
  - http://www.beaverdam.k12.wi.us/bd/family/lunch

Policy Examples: †

- Juneau School District Policy # 8500
  - http://www.juneauschools.org/board/policies/view?policy=8500
- Beaver Dam Unified School District, Section 341.7
  - http://www.beaverdam.k12.wi.us/bd/content/Policy_Wellness_Nutrition.PDF

*Note: 1 = meets criteria for policy effectiveness (consistent, positive outcomes from at least two high-quality experimental or quasi-experimental trials using a comparison group or interrupted time series design)^5^; 2 = consistent evidence available linking policy with positive outcomes from high-quality observational studies only; 3 = insufficient evidence available for policy or policy components.

† Be sure to check with your state, county, and municipal governments regarding potential existing laws that may impede any new policy development.

References

^1^ U.S. Centers for Disease Control and Prevention, National Center for Health Statistics. Health, United States, 2003. Atlanta, GA: U.S. Department of Health and Human Services, 2003.

^2^ Kubik, MY, Lytle, LA, Hannan, PJ, Perry, CL, & Story, M (2003). The association of the school food environment with dietary behaviors of young adolescents. American Journal of Public Health, 93(7), 1168-1173.

^3^ Kumanyika, S & Grier, S (2006). Targeting interventions for ethnic minority and low-income populations. Childhood Obesity, 16(1), 187-207.

^4^ Levi, J, Turst for America’s Health, et. al. (2009). F as in fat: How obesity policies are failing in America. The Robert Wood Johnson Foundation. Retrieved from: <http://www.rwjf.org/files/research/20090701tfahfasinfat.pdf>

^5^ Flay, BR, Biglan, A, Boruch, RF, Ganzalez Castro, F, Gottfredson, D, Kellam, S, Moscicki, EK, Schinke, S, Valentine, JC, & Ji, P (2005). Standards of evidence: Criteria for efficacy, effectiveness and dissemination. Prevention Science, 6(3), 151-175.

^6^ Jaime, PC & Lock, K (2009). Do school based food and nutrition policies improve diet and reduce obesity? Preventive Medicine, 48(2009), 45-53.

School Vocational Training Programs

Domain: School Influences

Reasons for Policy:

- Fewer than half of American birth cohorts ever attain any postsecondary education,^1^ and for these individuals, traditional high school curricula do not provide occupational skills.^2^
- Improving the quality of public schools, the readiness of school leavers, and the competitiveness of the workforce are all high national priorities.^3^

Community Group:

- Local School Board
- Local Employers
- Local Government

Policy Components:

- School vocational training should include a variety of school-based and work-based learning opportunities throughout high school including:
  - career exploration and counseling
  - integrated academic and occupational instruction that is focused on high standards of achievement
  - a variety of structured work experiences that teach broad, transferable workplace skills

Desired Outcomes:

- Reduced dropout rates among high risk students
- Increased job placement and job satisfaction
- Better wages/job opportunities
- Individual growth in cognitive and affective areas

Level of Evidence Available to Evaluate Effectiveness of Policy (1 = strong evidence to 3 = insufficient evidence*):^4^

1 = Meets Criteria for Effectiveness

Achievable Results:

The following summary of achievable results is based on a published review of the scientific evidence.

On average, vocational education programs can:

- Decrease high school dropout by 6%^5^
- Improve job satisfaction by 7%^5^

Vocational education does not appear to have significant effects on basic academic skills or performance on

standardized tests^5^

Community Examples:

- Seattle Public Schools, Washington have a Career and Technical Education Department dedicated to helping students develop practical skills for industry-defined work
  - <http://www.seattleschools.org/area/cte/index.dxml>
- East Pennsboro Area School District, Pennsylvania partners with an area school to provide vocational education programs for students interested in technical fields
  - <http://www.cpavts.org/cperryavts/site/Directory_List.asp?byType=96>

Links to Policy Examples: †

- Seattle Public Schools, Washington School Policy C48.00
  - See pdf
- East Pennsboro Area School District Policy 115
  - <http://www.epasd.org/5598757139425/cwp/view.asp?A=3&Q=275047&C=50724>

*Note: 1 = meets criteria for policy effectiveness (consistent, positive outcomes from at least two high-quality experimental or quasi-experimental trials using a comparison group or interrupted time series design)^4^; 2 = consistent evidence available linking policy with positive outcomes from high-quality observational studies only; 3 = insufficient evidence available for policy or policy components.

† Be sure to check with your state, county, and municipal governments regarding potential existing laws that may impede any new policy development.

References

^1^ U.S. Bureau of the Census (1992). Statistical Abstract of the United States, (112^th^ ed.). Washington, DC: U.S. Government Printing Office.

^2^ U.S. Department of Labor (1991). What work requires of schools: A SCANS report for America 2000. Washington, DC.

^3^ National Center on Education and the Economy, (2006). Tough choices or tough times: The report of the new commission on the skills of the American workforce. Josey Bass, Washington, DC.

^4^ Flay, BR, Biglan, A, Boruch, RF, Ganzalez Castro, F, Gottfredson, D, Kellam, S, Moscicki, EK, Schinke, S, Valentine, JC, & Ji, P (2005). Standards of evidence: Criteria for efficacy, effectiveness and dissemination. Prevention Science, 6(3), 151-175.

^5^ Kulik, JA (1994). Curricular tracks and high school vocational education. 1994 National Assessment of Vocational Education, Washington, DC.

Sexual Health Education and Contraceptive Interventions

Domain: School Influences

Reasons for Policy:

- Adolescents who have an unintended pregnancy are less likely to complete school education.^1^
- Children born to adolescent mothers are more likely to have low birth weight and become victims of neglect and abuse.^2^
- 27% of pregnancies among 15–19-year-olds ended in abortion in 2006.^3^

Community Group:

- School district/ local school board
- Local government
- Parent-Teacher Association

Policy Components:

- Sexual health education curriculum in schools
- Educational and participatory programs to improve knowledge about risks and consequences of pregnancy and STDs
- Educational intervention and contraception promotion
- Abstinence promotion

Desired Outcomes:

- Reduce unintended pregnancy
- Improve adolescent knowledge about risks and consequences of pregnancy and STDs
- Reduce risky sexual behaviors
- Reduce STD infections
- Reduce abortions

Level of Evidence Available to Evaluate Effectiveness of Policy (1 = strong evidence to 3 = insufficient evidence*):^4^

1 = Meets Criteria for Effectiveness

Achievable Results:

The following summary of achievable results is based on a published review of the scientific evidence.

On average a combination of educational and contraceptive interventions can achieve:

- Large reductions in unintended pregnancy (RR 0.49, 95% CI 0.33 – 0.74)^1^

Abstinence-plus programs (promote sexual abstinence, but also encourage safer-sex strategies) can achieve:

- Significant improvements in knowledge of HIV/AIDS information^5^
- Significant reductions in sexual risk behavior (reduce incidence and frequency of sexual activity and unprotected sex)^5^

Community Examples:

- Lake Washington School District, Washington uses The Great Body Shop and The Family Life and Sexual Health Curriculum (F.L.A.S.H. ) for health education
  - <http://www.thegreatbodyshop.net/frame.asp?NT=undefined>
  - <http://www.kingcounty.gov/healthservices/health/personal/famplan/educators/flash.aspx>
- Chicago, Illinois public schools offer sexual health and reproductive education to students
  - <http://www.cps.edu/Programs/Wellness_and_transportation/School_health_services/Pages/FamilyLife.aspx>

Links to Policy Examples†:

- Lake Washington School District, Washington approved The Great Body Shop and F.L.A.S.H. curriculums for use in the public schools
  - <http://www.lwsd.org/SiteCollectionDocuments/For-Parents/Adopted-Curriculum-2009.pdf>
- Chicago, Illinois the Board of Education for the City of Chicago requires schools to provide appropriate sexual health education to students
  - <http://policy.cps.k12.il.us/documents/704.6.pdf>

*Note: 1 = meets criteria for policy effectiveness (consistent, positive outcomes from at least two high-quality experimental or quasi-experimental trials using a comparison group or interrupted time series design)^4^; 2 = consistent evidence available linking policy with positive outcomes from high-quality observational studies only; 3 = insufficient evidence available for policy or policy components.

† Be sure to check with your state, county, and municipal governments regarding potential existing laws that may impede any new policy development.

References

1 Oringanje C, Meremikwu MM, Eko H, Esu E, Meremikwu A, Ehiri JE. Interventions for preventing unintended pregnancies among adolescents. Cochrane Database of Systematic Reviews 2009, Issue 4. Art. No.: CD005215.

2 Elfebein DS, Felice ME (2003). Adolescent pregnancy. *Pediatr Clin North Am*, 50(4), 781-800.

3 Guttmacher Institute (2010). *U.S. Teenage Pregnancies, Births and Abortions: National and State Trends and Trends by Race and Ethnicity*, http://www.guttmacher.org/pubs/FB-ATSRH.html#9, accessed Jan. 26, 2010.

4 Flay, BR, Biglan, A, Boruch, RF, Ganzalez Castro, F, Gottfredson, D, Kellam, S, Moscicki, EK, Schinke, S, Valentine, JC, & Ji, P (2005). Standards of evidence: Criteria for efficacy, effectiveness and dissemination. Prevention Science, 6(3), 151-175.

5 Underhill K, Montgomery P, Operario D. Abstinence-plus programs for HIV infection prevention in high-income countries. Cochrane Database of Systematic Reviews 2009, Issue 1. Art. No.: CD007006.

Volunteer Tutoring Programs

Domain: School Influences

Reasons for Policy:

- The effectiveness of tutoring has been documented extensively in various strands of the educational literature.^1^
- Volunteer tutoring programs provide an affordable way of offering additional services to students at risk of not meeting annual academic goals.^2^

Community Group:

- Local School Board
- Parent Teacher Association (PTA)
- Community service groups
- Local Government and policymakers
- Universities

Policy Components:

- Regular tutoring sessions with an academic focus at least one month in duration
- Tutoring programs should be highly structured with specifications on time spent in different activities or with specific lessons and materials to be covered
- Non-professional adult volunteer tutors, typically community members or university students
- Programs should be offered in schools that have very high dropout rates and high concentrations of low SES students

Desired Outcomes:

- Academic improvement in low-achieving students

Level of Evidence Available to Evaluate Effectiveness of Policy (1 = strong evidence to 3 = insufficient evidence*):

1 = Meets Criteria for Effectiveness

Achievable Results:

The following summary of achievable results is based on a published review of the scientific evidence.

On average, highly structured volunteer tutoring programs can achieve:

- Moderate improvements in overall reading (effect size 0.30)^2^
- Small improvements in global domain reading (effect size 0.26)^2^
- Moderate improvement in reading letters and words (effect size 0.41)^2^
- Moderate improvements in reading oral fluency (effect size 0.30)^2^
- Moderate improvements in writing (effect size 0.45)^2^

Community Examples:

- Bellingham Public Schools (Washington) include volunteer tutoring as a part of their student services
  - http://bellinghamschools.org/department/student-services
- Memphis City Schools (Tennessee) have established a volunteer tutoring program for its student in mathematics and reading.

Links to Policy Examples: †

- Bellingham Public School, Bellingham Washington
  - http://bellinghamschools.org/department-owner/school-board/2185policy
- Memphis City Schools, Memphis Tennessee
  - http://www.mcsk12.net/policies/4.501%20School%20Volunteers.pdf

*Note: 1 = meets criteria for policy effectiveness (consistent, positive outcomes from at least two high-quality experimental or quasi-experimental trials using a comparison group or interrupted time series design)^3^; 2 = consistent evidence available linking policy with positive outcomes from high-quality observational studies only; 3 = insufficient evidence available for policy or policy components.

† Be sure to check with your state, county, and municipal governments regarding potential existing laws that may impede any new policy development.

References

^1^ Wasik, BA & Slavin, RE (1993). Preventing early reading failure with one-to-one tutoring: A review of five programs. Reading Research Quarterly, 28(2), 178-200.

^2^ Ritter, G, Denny, G, Albin, G, Barnett, J, & Blankenship, V (2006). The effectiveness of volunteer tutoring programs: A systematic review. Campbell Systematic Reviews, 2006:7.

^3^ Flay, BR, Biglan, A, Boruch, RF, Ganzalez Castro, F, Gottfredson, D, Kellam, S, Moscicki, EK, Schinke, S, Valentine, JC, & Ji, P (2005). Standards of evidence: Criteria for efficacy, effectiveness and dissemination. Prevention Science, 6(3), 151-175.

After-School Programs that Promote Personal and Social Skills

Domain: Peer Influences

Reasons for Policy:

- An estimated 15% of children aged 6-12 are regularly unsupervised after school.^1^
- More than two-thirds of low- and moderate-income youth do not have parental supervision available after school.^2^
- Unstructured, unsupervised after-school time is associated with increased violence, delinquency, sexual intercourse, smoking, alcohol and drug use, and poor academic outcomes.^3^
- There is extensive evidence that youth can be taught personal and social skills^4^

Community Group:

- Local School Board
- Local Government
- Community-based organizations
- Universities

Policy Components:

- Programs follow the SAFE principles: sequenced, active, focused and explicit
- Employ evidence-based training methods for the program that target at least one of the following: problem-solving, conflict resolution, self-control, leadership, responsible decision-making, and enhancement of self-efficacy and self-esteem to target positive youth development

Desired Outcomes:

- Reduced risk-taking behaviors
- Positive youth development
- Enhanced academic, social, emotional, and behavioral growth

Level of Evidence Available to Evaluate Effectiveness of Policy (1 = strong evidence to 3 = insufficient evidence*):

1 = Meets Criteria for Effectiveness

Achievable Results:

The following summary of achievable results is based on a published review of the scientific evidence.^5^

Significant improvements have been observed for:

- Child self-perceptions, including self-esteem, self-concept, self-efficacy (moderate effect sizes of 0.34-0.37)^4,6^
- School bonding (small to moderate effect sizes of 0.14-0.25)^4,6^
- Positive social behaviors (small effect size 0.19)^6^
- Appropriate behavioral control (moderate effect size of 0.29)^4^
- Performance on standardized school achievement tests (small effect sizes of 0.16-0.20)^4,6^
- Grades in school (small effect sizes of 0.11-0.14)^4,6^

Significant reductions have been observed for:

- Problem behaviors, including non-compliance, aggression, delinquent acts, disciplinary referrals, and rebelliousness (small to moderate effect sizes of 0.18-0.30)^4,6^
- Self-reported drug use (small effect sizes of 0.11-0.16)^4,6^

Community Examples:

- Berkeley, CA Unified School District offers multiple after school programs
  - <http://www.berkeley.net/after-school-programs/>
- Broward, Florida host before and after school programs for elementary and middle school children.
  - <http://www.broward.k12.fl.us/k12programs/bascc/>

Links to Policy Examples†:

- Berkeley, CA Unified School District Policy details supplemental instruction for students
  - See PDF
- Broward, FL, School board policy 6000.3 Before and After Care Student Programs outlines the rules for providers
  - See PDF

*Note: 1 = meets criteria for policy effectiveness (consistent, positive outcomes from at least two high-quality experimental or quasi-experimental trials using a comparison group or interrupted time series design)^5^; 2 = consistent evidence available linking policy with positive outcomes from high-quality observational studies only; 3 = insufficient evidence available for policy or policy components.

† Be sure to check with your state, county, and municipal governments regarding potential existing laws that may impede any new policy development.

References

^1^ Vandiere, S, Tout, K, Zaslow, M, Clakins, J, & Capizzano, J ( 2003). Unsupervised time: Family and child factors associated with self-care. Washington, DC: Urban Institute.

^2^ US Bureau of Labor Statistics (2000). Families with own children: Employment status of parents by age of youngest child and family type, 2001-2002 annual averages. Retrieved from <http://www.bls.gov/news.release/famee/t04.htm>.

- - ^3^ U.S. Department of Education & U.S. Department of Justice (2000). Working for children and families: Safe and smart after-school programs. Washington, DC: Government Printing Office. Retrieved from <http://www.ed.gov>.

^4^ Durlak, JA, Weissberg, RP, & Pachan, M (2010). A meta-analysis of after-school programs that seek to promote personal and social skills in children and adolescents. American Journal of Community Psychology, 45, 294-309.

^5^ Flay, BR, Biglan, A, Boruch, RF, Ganzalez Castro, F, Gottfredson, D, Kellam, S, Moscicki, EK, Schinke, S, Valentine, JC, & Ji, P (2005). Standards of evidence: Criteria for efficacy, effectiveness and dissemination. Prevention Science, 6(3), 151-175.

^6^Durlak, J.A. & Weissberg, R.P. (2007). *The impact of after-school programs that seek to promote personal and social skills*. Chicago, IL: The Collaborative for Academic, Social and Emotional Learning; 2007.

School-Based Efforts to Reduce Bullying

Domain: Peer Influences

Reasons for Policy:

- Bullying has serious short-term and long term effects on children’s physical and mental health.^1^
- Victimization from bullying is strongly associated with depression.^2^

Community Group:

- School district
- Local school board
- State department of education
- Local government

Policy Components:

- Evidence-based bullying prevention or intervention programs
- School wide anti-bullying policy, including sanctions and deprivation of privileges for bullies
- Improved playground supervision
- Educational and informational materials for teachers and/or parents
- Teacher trainings

Desired Outcomes:

- Reduction in bullying
- Decrease in victimization
- Prevention of bullying and victimization
- Encouragement of pro-social behavior

Level of Evidence Available to Evaluate Effectiveness of Policy (1 = strong evidence to 3 = insufficient evidence*):^3^

1 = Meets Criteria for Effectiveness

Achievable Results:

The following summary of achievable results is based on a published review of the scientific evidence.

On average, school-based efforts to reduce bullying can achieve:

- 20-23% decrease in bullying (Odds Ratio: 1.36, 95% CI: 1.26-1.47)^1^

On average, school-based efforts to reduce victimization can achieve:

- 17-20% decrease in victimization (Odds Ratio: 1.29, 95% CI: 1.18-1.42)^1^

Community Examples:

- Broward County, Florida, Broward County Public Schools has an anti-bullying policy
  - <http://www.browardschools.com/schools/bullying.htm>
- New York City Department of Education implements an anti-bullying program called “Respect for All”
  - <http://schools.nyc.gov/RulesPolicies/RespectforAll/default.htm>

Links to Policy Examples:

- School Board of Broward County, Florida Policy 5.9: Anti-Bullying
  - <http://www.browardschools.com/schools/pdf/bully/Anti-BullyPolicy%205.9.pdf>
  - Florida Statutes §1006.147 Bullying and harassment prohibited: <http://www.leg.state.fl.us/statutes/index.cfm?mode=View%20Statutes&SubMenu=1&App_mode=Display_Statute&Search_String=1006.147&URL=CH1006/Sec147.HTM>
- New York City Department of Education, Rules and Policies, Citywide Standards of Discipline and Intervention Measures
  - <http://schools.nyc.gov/NR/rdonlyres/B4C3EAD9-AA61-4430-A6C3-D389F6238700/66073/DiscCode200981109_Final_English.pdf>

*Note: 1 = meets criteria for policy effectiveness (consistent, positive outcomes from at least two high-quality experimental or quasi-experimental trials using a comparison group or interrupted time series design)^3^; 2 = consistent evidence available linking policy with positive outcomes from high-quality observational studies only; 3 = insufficient evidence available for policy or policy components.

† Be sure to check with your state, county, and municipal governments regarding potential existing laws that may impede any new policy development.

‡Local governments and organizations may check existing state statutes and administrative codes for the authority to implement local policies.

References

1 Farrington, DP, Ttofi, MM (2009). School-Based Programs to Reduce Bullying and Victimization. Campbell Systematic Reviews, 2009:6.

2 Hawker, DJ, Boulton, MJ (2000). Twenty years research on peer victimization and psychosocial maladjustment: A meta-analytic review of cross-sectional studies.  *Journal of Child Psychology and Psychiatry*, 41, 441-455.

3 Flay, BR, Biglan, A, Boruch, RF, Ganzalez Castro, F, Gottfredson, D, Kellam, S, Moscicki, EK, Schinke, S, Valentine, JC, & Ji, P (2005). Standards of evidence: Criteria for efficacy, effectiveness and dissemination. Prevention Science, 6(3), 151-175.

Additional file 2b. Policy briefs for strategies with evidence of positive outcomes from high-quality observational studies (Level 2).

Active Labor Market Policies

Domain: Income & Resources

Reasons for Policy:

- ALMPs are designed to combat widespread unemployment by helping the unemployed find work.^1^
- Moving people into regular employment can bring families out of poverty and off of government assistance.^2^
- The heaviest burden of the current recession is falling on African Americans and Hispanics, who are contending with much higher unemployment rates than whites nationally--about one-and-a-half times as high for Hispanics and twice as high for blacks.^3^
- Equal access to work skill development and job search assistance can help reduce this growing disparity.^4^

Community Group:

- Local/state government
- Local businesses
- Community service organizations
- Community colleges

Policy Components:

- Labor market employment training
- Subsidized public sector employment programs/direct job creation
- Job search assistance
- Classroom and on-the-job training
- Wage subsidies to the private sector

Desired Outcomes:

- Increase employability of the unemployed
- Transition unemployed off of government assistance
- Enhance labor supply
- Increase labor demand
- Reduce unemployment disparities in racial/ethnic minorities

Level of Evidence Available to Evaluate Effectiveness of Policy (1 = strong evidence to 3 = insufficient evidence*):^5^

2 = Consistent evidence from high-quality observational studies only

Achievable Results:

The following summary of achievable results is based on a published review of the scientific evidence.

On average, Subsidized Public Sector Employment Programs:

- Have little impact^6^

On average, Job Search Assistance Programs can achieve:

- Relatively favorable, but small, short-term impacts^6^

On average, Classroom and on-the-job training programs can achieve:

- Favorable, but small, outcomes two-years post-program^6^

Community Examples:

- Massachusetts Community Colleges in conjunction with state businesses provide workforce training.
  - <http://www.masscc.org/inside.asp?id=10>
  - <http://www.middlesex.mass.edu/bus_ind/>
- Sonoma County,California, Sonoma County Workforce Investment Board in conjunction with Sonoma County Human Services Department develops a local workforce plan and promotes workforce development and training.
  - <http://www.sonomawib.org/>
  - <http://www.socojoblink.org/>
  - <http://www.sonomaworks.org/>

Links to Policy Examples:

- Annotated Laws of Massachusetts, ALM GL Ch. 15A, §15F Community College Workforce Training Incentive Program
  - See pdf
- County of Sonoma, California, Workforce Investment Board given authority pursuant to the Workforce Investment Act of 1998; Governor’s Executive Order D-9-99; County of Sonoma Board of Supervisors Resolution 98-1409, 99-1621.
  - <http://supervisors.sonoma-county.org/boards_and_commissions.aspx?sid=1001&bid=88&view=v>
- 29 U.S.C.A §2832 Local workforce investment boards
  - See pdf

*Note: 1 = meets criteria for policy effectiveness (consistent, positive outcomes from at least two high-quality experimental or quasi-experimental trials using a comparison group or interrupted time series design)^5^; 2 = consistent evidence available linking policy with positive outcomes from high-quality observational studies only; 3 = insufficient evidence available for policy or policy components.

† Be sure to check with your state, county, and municipal governments regarding potential existing laws that may impede any new policy development.

‡Local governments and organizations may check existing state and federal statutes and administrative codes for the authority to implement local policies.

References

^1^ Kluve, J (2000). The effectiveness of the European Active Labor Market Policy. Institute for the Study of Labor. IZA Discussion Paper No. 2018.

Available at: <http://ideas.repec.org/p/iza/izadps/dp2018.html>

^2^ Robinson, P (2000). Active labour-market policies: A case of evidence-based policy-making? Oxford Review of Economic Policy, 16, 13-26.

^3^ Austin, A (2009). Unequal unemployment: Racial disparities in unemployment vary widely by state. Economic Policy Institute: Research and Ideas for Shared Propserity <http://www.epi.org/publications/entry/ib257/>

^4^ Moira, N (2006). Unionized workers and support for active labor market policies. Fifteenth International Conference of the Council for European Studies. Chicago, March 29- April 2, 2006.

^5^ Flay, BR, Biglan, A, Boruch, RF, Ganzalez Castro, F, Gottfredson, D, Kellam, S, Moscicki, EK, Schinke, S, Valentine, JC, & Ji, P (2005). Standards of evidence: Criteria for efficacy, effectiveness and dissemination. Prevention Science, 6(3), 151-175.

^6^ Card, D, Kluve, J, Weber, A (2009). Active labor market policy evaluations: A meta-analysis. CESIFO Working Paper Series No. 2570; Ruhr Economic Paper No. 86. Available at SSRN: http://ssrn.com/abstract=13565234.

Child Health-Care Access

Domain: Income & Resources

Reasons for Policy:

- 37% of Hispanic, 23% of African American, and 20% of white children do not have health insurance.^1^
- Fewer than half of eligible children for State Children’s Health Insurance Program (SCHIP) are enrolled.^1^
- There are low levels of quality health care for children in Medicaid and commercial programs alike.^1^
- Low-income areas have 44% fewer physicians than high-income areas.^2^

Community Group:

- State government (e.g. state licensing requirements)
- Local government
- Local public health department
- Non-profit/community organizations

Policy Components:

- Make the SCHIP application process faster and easier
- Include parents of low-income children in health care coverage
- Wider distribution of eligibility information in locations where eligible enrollees may be found (e.g. food stamp and WIC offices, Head Start programs, local community centers)
- Physician participation in cultural competency, development, and behavioral pediatrics training

Desired Outcomes:

- Primary prevention of disease and reduced cost of care
- Higher enrollment rates of children and low-income families in SCHIP and Medicaid programs
- Better quality and availability of health care services
- Reduced health disparities between children of low- and high-income families

Level of Evidence Available to Evaluate Effectiveness of Policy (1 = strong evidence to 3 = insufficient evidence*):

2 = consistent evidence available linking policy with positive outcomes from high-quality observational studies only

Achievable Results:

The following summary of achievable results is based on a published review of the scientific evidence.

- States are able to effectively reach eligible enrollees at provider locations, community health centers, schools and adult education centers, beneficiaries’ homes, and social service agencies.^2^
- 55% decrease in incomplete SCHIP applications after streamlining the verification process.^2^
- 28.6% increase in SCHIP enrollment after coverage was expanded to parents.^2^
- 30% increase in SCHIP enrollment after simplifying their application form.^2^

Community Examples:

- - El Dorado, California First 5 Program Children’s Health Initiative aims to help families utilize and maintain healthcare coverage
    - <http://www.first5eldorado.com/initiatives/healthychildren/childhealth.html>
    - <http://www.ccfc.ca.gov/>
  - San Bernardino, California Health Care Access for all Children Initiative-Children and Families Commission for San Bernardino County
    - <http://www.first5sanbernardino.org/programs/health_care_access.aspx>

Links to Policy Examples†:

- El Dorado, California
  - - Children and Families First Commission Chapter 8.65 added to Title 8
      - See pdf
    - <http://www.co.el-dorado.ca.us/first5/ordinance.pdf>
- San Bernardino, California
  - Title 1, Division 2, Chapter 29: County Children and Families First

*Note: 1 = meets criteria for policy effectiveness (consistent, positive outcomes from at least two high-quality experimental or quasi-experimental trials using a comparison group or interrupted time series design)^3^; 2 = consistent evidence available linking policy with positive outcomes from high-quality observational studies only; 3 = insufficient evidence available for policy or policy components.

† Be sure to check with your state, county, and municipal governments regarding potential existing laws that may impede any new policy development.

References

^1^Beal AC (2004). Policies to reduce racial and ethnic disparities in child health and health care. *Health Affairs,* 23(5), 171-179.

^2^Zambrana RE, Carter-Pokras O (2004). Improving health insurance coverage for Latino children: A review of barriers, challenges, and state strategies. *Journal of the National Medical Association,* 96(4), 508-523.

^3^Flay, BR, Biglan, A, Boruch, RF, Ganzalez Castro, F, Gottfredson, D, Kellam, S, Moscicki, EK, Schinke, S, Valentine, JC, & Ji, P (2005). Standards of evidence: Criteria for efficacy, effectiveness and dissemination. Prevention Science, 6(3), 151-175.

College Grants & Financial Aid

Domains: Income & Resources

Reasons for Policy:

- College tuition prices have grown at rates that have far outpaced the ability of students and their families to pay for college.^1^
- Need-based grants can potentially increase the access and choice in higher education for low-income students.^1^

Community Group:

- Local government
- Community colleges, universities, and other post-secondary schools
- Non-profit organizations

Policy Component:

- Provide need-based grants for students to attend college

Desired Outcomes:

- Expand and equalize student access to higher education
- Increase enrollment in higher education among low income and racial/ethnic minority students

Level of Evidence Available to Evaluate Effectiveness of Policy (1 = strong evidence to 3 = insufficient evidence*):^2^

2=Consistent evidence from high-quality observational studies only

Achievable Results:

The following summary of achievable results is based on a published review of the scientific evidence.

- 16% of all full-time students enrolled in college because of the existence of need-based grants^3^
- Between 20%-40% of enrollment of lower-income students, and 13% of middle-income students, is due to need-based grants^3^

Community Examples:

- University System of Ohio, Ohio Board of Regents funds grants for state resident students who attend Ohio colleges.
  - <http://www.regents.ohio.gov/sgs/index.php>
- Baltimore, Maryland, CollegeBound Foundation, a private non-profit program comprised of business and community leaders, awards Baltimore City public school graduates with the Last Dollar Grant.
  - <http://www.collegeboundfoundation.org/lastDollar/lastDollarForm.aspx>

Links to Policy Examples:

- Ohio Revised Code (ORC) §§ 3333.12 Instructional grants, 3333.122 College opportunity grant program
  - See pdfs
- Baltimore, Maryland, CollegeBound Foundation Governance Policies
  - <http://www.collegeboundfoundation.org/aboutus/index.html>
  - Requested from the organization 8/17/10

*Note: 1 = meets criteria for policy effectiveness (consistent, positive outcomes from at least two high-quality experimental or quasi-experimental trials using a comparison group or interrupted time series design)^2^; 2 = consistent evidence available linking policy with positive outcomes from high-quality observational studies only; 3 = insufficient evidence available for policy or policy components.

† Be sure to check with your state, county, and municipal governments regarding potential existing laws that may impede any new policy development.

‡Local governments and organizations may check existing state statutes and administrative codes for the authority to implement local policies.

References

1 Heller, DE (1997). Student price response in higher education: An update to Leslie and Brinkman. The Journal of Higher Education, 68(6), 624-659.

2 Flay, BR, Biglan, A, Boruch, RF, Ganzalez Castro, F, Gottfredson, D, Kellam, S, Moscicki, EK, Schinke, S, Valentine, JC, & Ji, P (2005). Standards of evidence: Criteria for efficacy, effectiveness and dissemination. Prevention Science, 6(3), 151-175.

3 Leslie, LL & Brinkman, PT. (1987). *The economic value of higher education*. Washington: American Council on Education.

Condom Subsidies and Social Marketing Programs

Domain: Income & Resources

Reasons for Policy:

- Widespread condom use can reduce the spread of AIDS and other sexually transmitted diseases (STDs).^1^
- Low income groups are at high risk for STDs and unwanted pregnancies, but are disproportionately burdened by high priced condoms and are less likely to purchase them.^1^
- Adverse health consequences from sexual behavior, such as STD infections, are estimated to be at least threefold higher in the United States than in any other developed country.^1^

Community Group:

- Local Businesses
- Local Universities
- Local Public Health Department

Policy Components:

- Reduce the price of condoms through subsidies
- Increase physical access by making condoms readily available in settings where high-risk sexual activity is common
- Provide reproductive health information to reduce social constraints and increase awareness

Desired Outcomes:

- Sustained improvements in sexual health outcomes at the population level
- Increased condom use among poor and vulnerable groups
- Reduce inequities in condom access in high risk groups
- Reduce unintentional pregnancies
- Reduce STD transmission

Level of Evidence Available to Evaluate Effectiveness of Policy (1 = strong evidence to 3 = insufficient evidence*):^2^

2= Consistent evidence available linking policy with positive outcomes from high-quality observational studies only

Achievable Results:

The following summary of achievable results is based on a published review of the scientific evidence.

- There is a consistent negative correlation between price and contraceptive sales. Condom prices set below 1% of per-capita gross national product for a year’s supply may achieve satisfactory prevalence for condoms in either a family planning or AIDS-prevention context ^3^

This evidence is based on studies conducted in developing countries and may not be generalizable to the United States, or other developed countries. More research is needed.

Community Examples:

- The New York City Department of Health and Mental Hygiene implements NYC Condom a free condom initiative through its website.
  - <http://www.nyc.gov/html/doh/html/condoms/condoms.shtml>
- The District of Columbia, Department of Health, HIV/AIDS Administration runs a free condom distribution program, Free Condoms for DC. Teachers and counselors may distribute condoms to students if they complete an online training course.
  - <http://doh.dc.gov/doh/cwp/view.asp?a=1371&q=602647>
  - <http://www.washingtonpost.com/wp-dyn/content/article/2010/05/20/AR2010052003980.html>

Policy Examples:

- Rules of the City of New York, Title 24 Department of Health and Mental Hygiene, §3.01 General powers of the Department
  - See pdf
- District of Columbia Official Code, §7-1604 AIDS Program Coordination Office
  - See pdf

*Note: 1 = meets criteria for policy effectiveness (consistent, positive outcomes from at least two high-quality experimental or quasi-experimental trials using a comparison group or interrupted time series design)^2^; 2 = consistent evidence available linking policy with positive outcomes from high-quality observational studies only; 3 = insufficient evidence available for policy or policy components.

† Be sure to check with your state, county, and municipal governments regarding potential existing laws that may impede any new policy development.

References

^1^ Ebrahim, SH, McKenna, MT, Marks, JS (2005). Sexual behavior: related adverse health burden in the United States. Sexually Transmitted Infections, 81, 38-40.

^2^ Flay, BR, Biglan, A, Boruch, RF, Ganzalez Castro, F, Gottfredson, D, Kellam, S, Moscicki, EK, Schinke, S, Valentine, JC, & Ji, P (2005). Standards of evidence: Criteria for efficacy, effectiveness and dissemination. Prevention Science, 6(3), 151-175.

^3^ Harvey, PD (1994). The impact of condom prices on sales in social marketing programs. Studies in Family Planning, 25(1), 52-58.

Title: Living Wage Ordinances

Domain: Income & Resources

Reasons for Policy:

- Federally or state set minimum wages are often insufficient to support a family, leaving low-wage workers stuck in a cycle of poverty.^1^
- Living wage ordinances are cited as the most striking progressive achievement in labor and employment policy in the past 25 years.^2^

Community Group:

- Local Government
- Businesses under contract to provide services to the local government

Policy Components:

- A city ordinance mandating businesses under contract with the city, or receiving assistance from the city, must pay their workers a wage sufficient to support a family financially.
- Living wage laws mandate a wage floor at a level that is intended to sustain an average household.
- A minimum wage requirement higher than that set by federal or state legislation, which does not take into account family size or household income level of workers.

Desired Outcomes:

- Increase wages of low-wage workers
- Increase financial security of workers and their family
- Reduce urban poverty

Level of Evidence Available to Evaluate Effectiveness of Policy (1 = strong evidence to 3 = insufficient evidence*):

2 = Consistent Evidence Available

Achievable Results*:*

The following summary of achievable results is based on published reviews of the scientific evidence.

On average, living wage ordinances can achieve:

- Positive and significant effects on the wages of low-wage workers^1^
- Increasing pay in low-skill jobs^2^
- Modest reductions in the likelihood that urban families live in poverty^1^
- Reduction in turnover^2^
- Very little to no employment loss^3^

Estimates of the costs of living wage ordinances find that contractor operating costs would increase by 1%-2%, which would be borne by the city as less than 1% of municipal revenue^4^

Community Examples:

- Los Angeles County, California requires all contract employers to pay workers $9.64 per hour if they are receiving health benefits and $11. 84 if they do not receive health benefits. The current state minimum wage is $8.00 per hour.
  - http://doingbusiness.lacounty.gov/living_wage.htm
- Bloomington, Indiana requires covered employers to pay a living wage of $11.25 in contrast to $7.25 that is mandated by state and federal law.
  - http://bloomington.in.gov/sections/viewSection.php?section_id=79

Links to Policy Examples: †

- Los Angeles County California County Code, Title 2 Chapter 2.201
  - <http://search.municode.com/html/16274/index.htmWebsites>
- Bloomington Indiana Municipal Code Chapter 2.28
  - <http://bloomington.in.gov/code/>

*Note: 1 = meets criteria for policy effectiveness (consistent, positive outcomes from at least two high-quality experimental or quasi-experimental trials using a comparison group or interrupted time series design)^5^; 2 = consistent evidence available linking policy with positive outcomes from high-quality observational studies only; 3 = insufficient evidence available for policy or policy components.

† Be sure to check with your state, county, and municipal governments regarding potential existing laws that may impede any new policy development.

References

^1^ Neumark, D, & Adams, S (2003). Do living wage ordinances reduce urban poverty? Journal of Human Resources, 38(3), 490-521.

^2^ Fairris, D, & Reich, M (2005). The impacts of living wage policies: introduction to the special issue. Industrial Relations, 44(1), 1-13.

^3^ Brown, C, (1999). Minimum wages, employment, and the distribution of income. Handbook of Labor Economics*,* vol. 3. New York: Elsevier.

^4^ Pollin, R (2005). Evaluating living wage laws in the United States. Economic Development Quarterly, 19(1), 3-24.

^5^ Flay, BR, Biglan, A, Boruch, RF, Ganzalez Castro, F, Gottfredson, D, Kellam, S, Moscicki, EK, Schinke, S, Valentine, JC, & Ji, P (2005). Standards of evidence: Criteria for efficacy, effectiveness and dissemination. Prevention Science, 6(3), 151-175.

Anti-Discrimination and Diversity Policies

Domain: Social Cohesion

Reasons for Policy:

- In 2008, among private industry in the U.S., women constituted 29% of executive or senior level managerial positions (Women constituted 48% of total employment in 2008).^1^
- In 2008, among private industry in the U.S., minorities constituted 12% of executive or senior level managerial positions (Minorities constituted 34% of total employment in 2008).^1^
- Although racial/ethnic minority women remain a relatively small percentage of all officials and managers, between 1990 and 2001 their rates of employment in these positions increased, ranging from 75% for African American women to 135% for Asian women.^2^
- However, the exclusion of women from management positions is more severe for racial/ethnic minority women.^2^
- Inequality at work may be rooted in managerial bias and the social isolation of women and racial/ethnic minorities.^3^

Community Group:

- Local businesses
- Corporations
- Local/state government

Policy Components:

- Organizational responsibility practices
- Affirmative action plans
- Diversity staff
- Diversity task force/committee

Desired Outcomes:

- Increase diversity in the workplace
- Increase diversity in private section management
- Increase employment of racial/ethnic minorities and women
- Establish responsibility for diversity

Level of Evidence Available to Evaluate Effectiveness of Policy (1 = strong evidence to 3 = insufficient evidence*):^4^

2 = Consistent evidence from high-quality observational studies

Achievable Results:

The following summary of achievable results is based on a published review of the scientific evidence.

On average, a diversity committee can achieve:

- 19% increase in odds that a white woman is in a managerial position.^3^
- 27% increase in odds that an African American woman is in a managerial position.^3^
- 12% increase in odds that an African American man is in a managerial position.^3^

On average, appointing full-time diversity staff can achieve:

- 11% increase in odds that a white woman is in a managerial position.^3^
- 13% increase in odds that an African American woman is in a managerial position.^3^
- 14% increase in odds that an African American man is in a managerial position.^3^
- Affirmative action plans, diversity committees and taskforces, diversity managers and departments are the most effective means of increasing the proportions of white women, African American women and African American men in private sector management.^3^

Community Examples:

- Connecticut State University System has an affirmative action plan and an affirmative action officer to advance social justice and equity.
  - <http://www.ct.edu/hr/affirmative-action/>
  - See pdf
- Torrance, California, American Honda Motor Co., Inc. is committed to maintaining a diverse workforce through its affirmative action and non-discrimination policies.
  - <http://corporate.honda.com/careers/diversity.aspx?id=diversity_overivew>
  - See pdf

Links to Policy Examples:

- Connecticut, C.G.S. § 10a-10, Office of Educational Opportunity
  - See pdf
- American Honda Motor Co., Inc., Affirmative Action Policy Statement
  - <http://corporate.honda.com/careers/diversity.aspx?id=policies#aaps>
  - See pdf

*Note: 1 = meets criteria for policy effectiveness (consistent, positive outcomes from at least two high-quality experimental or quasi-experimental trials using a comparison group or interrupted time series design)^4^; 2 = consistent evidence available linking policy with positive outcomes from high-quality observational studies only; 3 = insufficient evidence available for policy or policy components.

† Be sure to check with your state, county, and municipal governments regarding potential existing laws that may impede any new policy development.

‡Local governments and organizations may check existing state and federal statutes and administrative codes for the authority to implement local policies.

References

1 The U.S. Equal Employment Opportunity Commission (2008). Job Patterns For Minorities and Women in Private Industry. Retrieved from <http://www.eeoc.gov/eeoc/statistics/employment/jobpat-eeo1/index.cfm>.

2 The U.S. Equal Employment Opportunity Commission (2003). Women of Color: Their Employment in the Private Sector. Retrieved from <http://www.eeoc.gov/eeoc/statistics/reports/womenofcolor/index.html>.

3 Kalev, A, Dobbin, F, Kelly, E (2006). Best Practices or Best Guess? Assessing the Efficacy of Corporate Affirmative Action Diversity Policies. American Sociological Review, 71, 589-617.

4 Flay, BR, Biglan, A, Boruch, RF, Ganzalez Castro, F, Gottfredson, D, Kellam, S, Moscicki, EK, Schinke, S, Valentine, JC, & Ji, P (2005). Standards of evidence: Criteria for efficacy, effectiveness and dissemination. Prevention Science, 6(3), 151-175.

Community-based Arts Programs

Domain: Social Cohesion

Reasons for Policy:

- Supporting participation in the arts can help neighborhood renewal.^1^
- Participation in the arts can lead to increased performance on indicators of health, crime, employment, and education.^1^

Community Group:

- Local government
- Local community centers
- Local artists and businesses

Policy Components:

- Provision of community art programs, such as art classes and community art shows

Desired Outcomes:

- Increased social cohesion and social capital
- Increased performance on health and education indicators
- Improved mental health
- Improved neighborhood renewal

Level of Evidence Available to Evaluate Effectiveness of Policy (1 = strong evidence to 3 = insufficient evidence*):^2^

2 = consistent evidence from observational studies

Achievable Results:

The following summary of achievable results is based on a published review of the scientific evidence.

On average, community art programs can achieve:

- Positive effects on a personal level such as, making new friends, feelings of being happier, more creative and confident, and reduced feelings of isolation^1^
- Positive effects on a social level such as, greater community understanding, bringing different groups together, greater sense of community^1^
- Positive effects on an economic level such as, new jobs, increased sales of art work, greater inward investment in the community^1^
- Positive effects on an educational level such as, improved school performance^1^

Although results are generally positive, it is difficult to scientifically evaluate the effects of art programs and more research is needed.

Community Examples:

- Tacoma, Washington seeks to promote urban street art while aiming to reduce vandalism.
  - <http://www.cityoftacoma.org/Page.aspx?cid=12837>
- Pinellas County, Florida has a Public Art & Design Program.
  - <http://www.pinellasarts.org/public_art_and_design.htm>

Links to Policy Examples:

- Tacoma, Washington public art policy in city legislative policy manual (pg. 17)
  - <http://cms.cityoftacoma.org/cityclerk/files/documents/LegislativePolicyManual.pdf>
- Pinellas County, Florida, Code of Ordinances, Ch. 90, Art. III, §90-146 Public art and design
  - <http://library.municode.com/index.aspx?clientId=10274&stateId=9&stateName=Florida>
  - see pdf

*Note: 1 = meets criteria for policy effectiveness (consistent, positive outcomes from at least two high-quality experimental or quasi-experimental trials using a comparison group or interrupted time series design)^2^; 2 = consistent evidence available linking policy with positive outcomes from high-quality observational studies only; 3 = insufficient evidence available for policy or policy components.

† Be sure to check with your state, county, and municipal governments regarding potential existing laws that may impede any new policy development.

References

^1^Newman T, Curtis K, & Stephens J (2003). Do community-based arts projects result in social gains? A review of the literature. *Community Development Journal,* 38(4), 310-322.

^2^Flay, BR, Biglan, A, Boruch, RF, Ganzalez Castro, F, Gottfredson, D, Kellam, S, Moscicki, EK, Schinke, S, Valentine, JC, & Ji, P (2005). Standards of evidence: Criteria for efficacy, effectiveness and dissemination. Prevention Science, 6(3), 151-175.

Employee Share Ownership and Profit-sharing

Domain: Social Cohesion

Reasons for Policy:

- Group-based incentive systems have the potential to redistribute wealth and increase economic democracy.^1^
- Employee financial participation can lead to higher levels of trust and organizational commitment^2^ and improved relations among management and co-workers.^1^
- ESOP firms grow faster and generate more new jobs, thus promoting economic development in a community.^3^

Community Group:

- Local and state government
- Local businesses

Policy Components:

- Provide employees with a residual right to firm’s surplus through profit sharing (i.e., a portion of the profit is paid to employees in addition to their wage) or employee share ownership (i.e., employees own shares in the company in which they work).
- Provide sufficient information and communication to workers to have an impact on productivity
- Employee participation in governance of the firm
- Ensure all employees participate equally
- Local government subsidies to firms involved in financial participation and tax relief to financial institutions that lend to ESOPs
- Government support in outreach, education, loans and loan guarantees, and technical assistance for ESOPs

Desired Outcomes:

- Increased productivity
- Increase employee longevity
- Increased employment

Level of Evidence Available to Evaluate Effectiveness of Policy (1 = strong evidence to 3 = insufficient evidence*):^4^

2 = Consistent Evidence from Observational Studies Only

Achievable Results:

The following summary of achievable results is based on a published review of the scientific evidence.

On average, worker participation in decision making can achieve:

- Small improvements in worker productivity (r=0.06), with greater effects observed in labor-managed firms (r= 0.10) compared to participatory capitalist firms (r=0.04)^5^

On average, profit sharing can achieve:

- Small improvements in worker productivity (r=0.05), with larger effects observed in labor-managed firms (r=0.26) compared to participatory capitalist firms (r=0.04).^1,5^

On average, worker ownership can achieve:

- Small improvements in worker productivity (r=0.03), with larger effects observed in labor-managed firms (r=0.10) compared to participatory capitalist firms (r=0.02).^1,5^

Community Examples:

- Phelps County, Missouri, Phelps County Bank is a community bank owned by the employees through an Employee Stock Ownership Plan. (ESOP).
  - <http://www.phelpscountybank.com/history.cfm>
- Indianapolis, Indiana, AIT laboratories transferred ownership to employees through an ESOP.
  - <http://www.aitlabs.com/articles.aspx?id=2424>

Links to Policy Examples:

- Missouri Statutes, §362.067 Stock option plan for officers and employees—increase in shares—consideration
  - See pdf
- Indiana State Treasurer created Indiana’s ESOP Initiative (IEI)
  - <http://www.in.gov/tos/2343.htm>
  - <http://www.in.gov/tos/2360.htm>
  - Indiana Code 4-8.1-2 The Treasurer of State
    - <http://www.in.gov/legislative/ic/code/title4/ar8.1/ch2.html>

*Note: 1 = meets criteria for policy effectiveness (consistent, positive outcomes from at least two high-quality experimental or quasi-experimental trials using a comparison group or interrupted time series design)^4^; 2 = consistent evidence available linking policy with positive outcomes from high-quality observational studies only; 3 = insufficient evidence available for policy or policy components.

† Be sure to check with your state, county, and municipal governments regarding potential existing laws that may impede any new policy development.

‡Local governments and organizations may check existing state and federal statutes and administrative codes for the authority to implement local policies.

References

^1^ Perotin, V & Robinson, A (2002). Employee participation in profit and ownership: A review of the issues and evidence. Paper prepared for the European Parliament. Retrieved on June 24, 2010 at: <http://www.efesonline.org/LIBRARY/Employee%20Participation%20in%20Profit%20and%20Ownership%20P%E9rotin%2003.pdf>

^2^ Coyle-Shapiro, J, Morrow, PC, Richardson, R, & Dunn, SR (2002). Using profit sharing to enhance employee attitudes: A longitudinal examination of the effects on trust and commitment. Human Resource Management, 41(4), 423-439.

^3^ Thompson, JK (1993). Promotion of employee ownership through public policy: The British example. Journal of Economic Issues, 27(3), 825-847.

^4^ Flay, BR, Biglan, A, Boruch, RF, Ganzalez Castro, F, Gottfredson, D, Kellam, S, Moscicki, EK, Schinke, S, Valentine, JC, & Ji, P (2005). Standards of evidence: Criteria for efficacy, effectiveness and dissemination. Prevention Science, 6(3), 151-175.

^5^ Doucouliagos, C (1995). Worker participation and productivity in labor-managed and participatory capitalist firms: a meta-analysis. Industrial and Labor Relations Review, 49(1), 58-77.

Area-Wide Traffic Calming Devices

Domain: Physical Environment

Reasons for Policy:

- In the U.S., pedestrian deaths account for 15% of all road traffic fatalities.^1^
- In 2008, 4,378 pedestrians were killed and 69,000 pedestrians were injured in traffic crashes in the U.S.^2^
- In 2008, nearly one in five children between the ages of 5 and 9 killed in traffic crashes were pedestrians.^2^
- Area-wide traffic calming has the potential to reduce deaths and injuries.^1^

Community Group:

- Local government
- State government
- Transportation department
- Local planning council

Policy Components:

- Area-wide traffic calming measures designed to discourage the use of residential streets for through travel and create an environment where residential streets are safe. Strategies include:
  - Vertical and horizontal shifts in traffic (road humps, speed cushions, raised crosswalks, road narrowing)
  - Optical measures (chevron road signs, reflective surface treatment, shortened sightlines, alterations to road lighting)
  - Redistribution or alteration of traffic (permanent or temporary blocking of road, diagonal blocks, traffic circles, creation of one-way streets
  - Changes to road environment (increased vegetation along road)
  - Reduced speed limit zones

Desired Outcomes:

- Reduction in road traffic fatalities
- Reduction in road traffic injuries
- Reduction in traffic crashes
- Reduction in pedestrian-motor vehicle collisions

Level of Evidence Available to Evaluate Effectiveness of Policy (1 = strong evidence to 3 = insufficient evidence*):^3^

2 = Consistent evidence from high-quality observational studies only

Achievable Results:

The following summary of achievable results is based on a published review of the scientific evidence.

On average, area-wide traffic calming can achieve:

- Small reductions in road traffic injuries (RR = 0.85, 95% CI: 0.75-0.96)^1^

Note: Area-wide traffic calming appears to be a promising intervention for reducing traffic injuries and deaths (effects are in the hypothesized direction but not statistically significant). However, no studies with randomized controlled trials were found. Further research, which includes well-designed controlled studies, is needed to effectively evaluate traffic calming interventions.^1^

Community Examples:

- City of Lancaster, California, Public Works, Traffic Engineering Division implements a traffic calming policy.
  - <http://www.cityoflancasterca.org/Index.aspx?page=407>
  - See pdf
- City of Loveland, Colorado, Public Works implements a traffic calming program.
  - <http://www.ci.loveland.co.us/PublicWorks/Traffic/trafficcalming.htm>
  - See pdf

Links to Policy Examples:

- City of Lancaster Citywide Traffic Calming Policy
  - See pdf
- Loveland Municipal Code, Chapter 2.48 Department of Community Services
  - See pdf

*Note: 1 = meets criteria for policy effectiveness (consistent, positive outcomes from at least two high-quality experimental or quasi-experimental trials using a comparison group or interrupted time series design)^3^; 2 = consistent evidence available linking policy with positive outcomes from high-quality observational studies only; 3 = insufficient evidence available for policy or policy components.

† Be sure to check with your state, county, and municipal governments regarding potential existing laws that may impede any new policy development.

‡Local governments and organizations may check existing state and federal statutes and administrative codes for the authority to implement local policies.

References

1 Bunn, F, et al. (2003). Area-wide traffic calming for preventing traffic related injuries. *Cochrane Database of Systematic Reviews* 2003, 1, CD003110.

2 National Center for Statistics and Analysis, NHTSA (2009). Traffic Safety Facts: 2008 Data. National Highway and Traffic Safety Administration, Department of Transportation, Washington DC, DOT HS 811 163, retrieved from: <http://www-nrd.nhtsa.dot.gov/Pubs/811163.PDF>.

3 Flay, BR, Biglan, A, Boruch, RF, Ganzalez Castro, F, Gottfredson, D, Kellam, S, Moscicki, EK, Schinke, S, Valentine, JC, & Ji, P (2005). Standards of evidence: Criteria for efficacy, effectiveness and dissemination. Prevention Science, 6(3), 151-175.

Farm-to-School Programs

Domain: Physical Environment

Reasons for Policy:

- Farm to school programs create sustainable food systems in schools, which both support the local economy and deliver fresh, healthy foods to students.^1^
- Improved school food environment can help address dietary factors related to obesity and diabetes among youth.^1^
- The 2008 US Farm Bill allows schools to give preference to local agriculture, making farm to school programs easier to establish.^2^

Community Group:

- Local Farmers
- School District
- School Food Service Manager
- Parent Teacher Organization

Policy Components:

- Facilitate direct purchases between food service and farmers,
- Uses a forager to act as a go-between to facilitate purchasing,
- Arranges for purchase through a local farmer’s market,
- Enters into a “contract growing” arrangement with farmers, or
- Sources locally grown food through a distributor

Desired Outcomes:

- Improved health and nutrition of school-age children
- Strengthened capacity of local farmers
- Establishment of a community food systems approach which is independent of the global food system

Level of Evidence Available to Evaluate Effectiveness of Policy (1 = strong evidence to 3 = insufficient evidence*):^3^

2 = Consistent evidence available linking policy with positive outcomes from high-quality observational studies only.

Achievable Results:

The following summary of achievable results is based on a published review of the scientific evidence.

On average, farm to school programs can achieve:^4^

For schoolchildren:

- Increased access to fresh foods at school
- Experiential education for sustainability

For food service managers:

- Easier and less costly to provide healthy food to children

Local farmers:

- Steady income and convenient delivery
- Reductions in urban sprawl and protection of farmland

Community benefits:

- Infusion of money into the local economy
- Building relationships and community capacity
- Reductions in urban sprawl and protection of farmland

Community Examples:

- Auburn School District, Washington, is one of several school districts in Washington to partner with local farmers to provide fresh, local foods for students.
  - <http://www.farmtoschool.org/state-programs.php?action=detail&id=8&pid=298>
  - <http://agr.wa.gov/Marketing/Farmtoschool/>
- The New North Florida Cooperative Farm to School program partnered with Florida A&M University to provide local produce to several Florida school districts.
  - <http://www.farmtoschool.org/state-programs.php?action=detail&id=23&pid=32>
- University of Florida/Institute of Food and Agricultural Sciences Extension and Sarasota County cooperate to provide fresh food to local school districts.
  - <http://www.farmtoschool.org/state-programs.php?action=detail&id=23&pid=33>

Links to Policy Examples:

- Washington Revised Code §15.64.060
  - See pdf
- Florida Statutes, 1006.06(6) establishes the Florida Farm Fresh Schools Program
  - See pdf

*Note: 1 = meets criteria for policy effectiveness (consistent, positive outcomes from at least two high-quality experimental or quasi-experimental trials using a comparison group or interrupted time series design)^3^; 2 = consistent evidence available linking policy with positive outcomes from high-quality observational studies only; 3 = insufficient evidence available for policy or policy components.

† Be sure to check with your state, county, and municipal governments regarding potential existing laws that may impede any new policy development.

‡Local governments and organizations may check existing state statutes and administrative codes for the authority to implement local policies.

References

^1^ Vallianatos M, Gottlieb R, Haase MA. Farm-to-school: strategies for urban health, combating sprawl, and establishing a community food systems approach. *J Plann Educ Res*. 2004; 23:414–423.

^2^ Larsen, S, (2008). Farm bill timing. *Community Food Security Coalition Electronic Newsletter*. May 2008.

^3^ Flay, BR, Biglan, A, Boruch, RF, Ganzalez Castro, F, Gottfredson, D, Kellam, S, Moscicki, EK, Schinke, S, Valentine, JC, & Ji, P (2005). Standards of evidence: Criteria for efficacy, effectiveness and dissemination. Prevention Science, 6(3), 151-175.

^4^ Carlsson, L & Williams, PL, (2008). New approaches to the health promoting school: participation in sustainable food systems. *Journal of Hunger and Environmental Nutrition*, 3(4), 400-417

Point of Purchase Nutrition Strategies

Domain: Physical Environment

Reasons for Policy:

- Obesity is associated with increased risk for many common chronic diseases.^1^
- Prevalence of childhood obesity has risen sharply in the past three decades, and is closely associated with poor dietary choices.^2^
- Environmental interventions are a promising tool for shifting US dietary patterns.^3^

Community Group:

- Universities/Colleges
- School Board
- Health Department
- Local businesses/workplaces

Policy Components:

- Increased availability of healthy foods
- Nutritional content labels and signs
- Pricing and/or rebates encouraging purchase of healthy foods

Desired Outcomes:

- Increased purchase and consumption of healthier foods

Level of Evidence Available to Evaluate Effectiveness of Policy (1 = strong evidence to 3 = insufficient evidence*):^4^

2 = Consistent Evidence Available from High-quality Observational Studies Only

Achievable Results:

The following summary of achievable results is based on a published review of the scientific evidence.

- Point of purchase nutritional information may be associated with positive changes in sales of targeted food and dietary patterns within workplace and university settings. Evidence for other settings, including grocery stores and restaurants, is limited.^3^
- Increased availability of health food choices may be associated with increases in sales of healthy foods/snacks.^3^
- Incentives (decreased price/rebates) combined with information strategies may be associated with increased sales of targeted items.^3^

More research is needed to understand the impact of point of purchase nutrition strategies.

Community Examples:

- Baltimore, Maryland, Johns Hopkins Bloomberg School of Public Health and the Baltimore City Health Department work in partnership to lead the Baltimore Healthy Stores project.
  - <http://www.healthystores.org/BHS.html>
- City of Baltimore announced recommendations to increase demand and access to healthy food options.
  - <http://www.baltimorecity.gov/OfficeoftheMayor/NewsPressReleases/tabid/66/ID/365/Baltimore_City_Food_Policy_Task_Force_Makes_Citywide_Recommendations_for_a_Healthier_Baltimore.aspx>
- Minneapolis, Minnesota, City Council approved The Healthy Corner Store Program to increase access to healthy foods.
  - <http://www.mndaily.com/2010/04/12/minneapolis-project-aims-increase-access-healthy-foods>

Links to Policy Examples:

- Baltimore, Maryland; Baltimore City Charter, and Baltimore City Health Code
  - <http://www.baltimorecity.gov/Government/AgenciesDepartments/Planning/FoodPolicyTaskForce.aspx>
  - Baltimore City Charter, Article VII, §56 Department of Health: powers and duties.
  - Baltimore City Health Code, §§ 2-104 Commissioner’s Charter powers., 2-105 General duties.
- Minneapolis, Minnesota; Minneapolis Code of Ordinances, Title 10, Chapter 203 Grocery Stores and Specialty Food Stores
  - See pdf

*Note: 1 = meets criteria for policy effectiveness (consistent, positive outcomes from at least two high-quality experimental or quasi-experimental trials using a comparison group or interrupted time series design)^4^; 2 = consistent evidence available linking policy with positive outcomes from high-quality observational studies only; 3 = insufficient evidence available for policy or policy components.

† Be sure to check with your state, county, and municipal governments regarding potential existing laws that may impede any new policy development.

References

^1^ Centers for Disease Control and Prevention (2010). Health Consequences of Obesity. Retrieved July 15, 2010 from: <http://www.cdc.gov/obesity/causes/health.html>

^2^ Robert Wood Johnson Foundation (2009). F as in fat: How obesity policies are failing in America. Trust for America’s Health, Issue Report. Retrieved July 15, 2010 from: [www.healthyamericans.org/reports/obesity2010](http://www.healthyamericans.org/reports/obesity2010)

^3^ Seymour, JD, Yaroch, AL, Serdula, M, Blanck, HM & Khan, LK (2004). Impact of nutrition environmental interventions on point-of-purchase behavior in adults: a review. Preventive Medicine, 39, S108-S136.

^4^ Flay, BR, Biglan, A, Boruch, RF, Ganzalez Castro, F, Gottfredson, D, Kellam, S, Moscicki, EK, Schinke, S, Valentine, JC, & Ji, P (2005). Standards of evidence: Criteria for efficacy, effectiveness and dissemination. Prevention Science, 6(3), 151-175.

Pool Fencing for Preventing Drowning

Domain: Physical Environment

Reasons for Policy:

- Drowning is the third leading cause of unintentional injury to children under age 15.^1^
- Death rates from drowning are highest in children less than five years old.^1^
- For each childhood drowning fatality, four children are hospitalized and 14 are seen in the emergency department (ED) and released. For every child seen in the ED, there are 10 near misses (child immersed but was rapidly rescued).^1^
- Drowning rates are higher in locations where the weather is hot and exposure to swimming pools is greatest.^1^
- Swimming pools pose the greatest risk of drowning to toddlers.^1^

Community Group:

- Local government
- State government
- Health department

Policy Components:

- Isolation fencing (i.e., pool fencing that completely encloses a swimming pool and isolates it from the home).

Desired Outcomes:

- Prevention/reduction of drowning fatalities
- Prevention/reduction in near-drowning (non-fatal submersion resulting in treatment in a hospital or emergency department)

Level of Evidence Available to Evaluate Effectiveness of Policy (1 = strong evidence to 3 = insufficient evidence*):^2^

2 = Consistent evidence from high-quality observational studies only

Achievable Results:

The following summary of achievable results is based on a published review of the scientific evidence.

On average, isolation pool fencing can achieve:

- Large reductions in the risk of drowning (Odds Ratios: 0.17-0.29).^1*^

*Note: Recent studies evaluating pool-fencing ordinances indicate that the mere passage of legislation is not sufficient to reduce drowning. In those studies there was inadequate enforcement of the ordinance.

Legislation should include enforcement provisions and educational campaigns should accompany the

legislation. Legislation should also require fencing of both newly constructed and existing pools.

Additional studies are needed to provide a more precise estimate of the protective effect of fencing.^1^

Community Examples:

- Arizona Department of Health Services, Office of Environmental Safety, Residential Pool Safety Notice
  - <http://www.azdhs.gov/phs/oeh/pool_rules.htm>
  - See pdf
- Pinellas County, Florida, Pinellas County Building and Development Review Services indicate that all new private swimming pools provide a barrier around the pool.
  - <http://www.pinellascounty.org/build/swimpool.htm>
  - See pdf

Links to Policy Examples:

- Florida Building Code § 424.2.17 Residential swimming barrier requirement
  - See pdf
- Arizona Revised Statues § 36-1681 Pool enclosures; requirements; exceptions; enforcement
  - See pdf

*Note: 1 = meets criteria for policy effectiveness (consistent, positive outcomes from at least two high-quality experimental or quasi-experimental trials using a comparison group or interrupted time series design)^2^; 2 = consistent evidence available linking policy with positive outcomes from high-quality observational studies only; 3 = insufficient evidence available for policy or policy components.

† Be sure to check with your state, county, and municipal governments regarding potential existing laws that may impede any new policy development.

‡Local governments and organizations may check existing state and federal statutes and administrative codes for the authority to implement local policies.

References

1 Thompson, DC, Rivara, F (1998). Pool fencing for preventing drowning of children. *Cochrane Database of Systematic Reviews* 1998, 1, CD001047.

2 Flay, BR, Biglan, A, Boruch, RF, Ganzalez Castro, F, Gottfredson, D, Kellam, S, Moscicki, EK, Schinke, S, Valentine, JC, & Ji, P (2005). Standards of evidence: Criteria for efficacy, effectiveness and dissemination. Prevention Science, 6(3), 151-175.

Speed Enforcement Detection Devices

Domain: Physical Environment

Reasons for Policy:

- Speeding increases the risk of crashing and of injury.^1^
- It is estimated that by 2020, road traffic crashes will move from 9^th^ to 3^rd^ in the world ranking of burden of disease, as measured in disability adjusted life years.^1^
- In 2006 in the U.S., motor-vehicle traffic-related injuries accounted for 24.4% of all injury deaths.^2^
- Measures to reduce traffic speed are considered essential to reducing casualties on the road.^3^

Community Group:

- Local government
- Local law enforcement
- Transportation department
- State government

Policy Components:

- Automatic speed enforcement detection devices (camera)
- Fixed overt cameras
- Fixed covert cameras
- Mobile overt cameras
- Mobile covert cameras
- A combination of fixed or mobile and overt or covert cameras

Desired Outcomes:

- Reduction of traffic injuries
- Reduction of traffic deaths
- Reduction of traffic crashes
- Reduction in speeding drivers

Level of Evidence Available to Evaluate Effectiveness of Policy (1 = strong evidence to 3 = insufficient evidence*):^4^

2 = Consistent Evidence from High Quality Observational Studies Only

Achievable Results:

The following summary of achievable results is based on a published review of the scientific evidence.

On average, speed enforcement detection devices can achieve:

- 5-36% reduction of road traffic crashes.^1^
- 14%-34% reduction of injury crashes.^1^
- A reduction in average speeds.^1^

*Note: Speed detection enforcement devices reduce road traffic injuries and related road injuries and

deaths, however studies of a higher quality are needed.

Community Examples:

- Montgomery County, Maryland implements “Safe Speed”, an automated speed enforcement program. The program enforces speed limits of 35 mph or less in residential areas and in school zones with any speed.^‡^
  - <http://www.montgomerycountymd.gov/poltmpl.asp?url=/content/POL/districts/FSB/sod/speed/Speed.asp>
  - see pdf
- Baltimore County Council, MD authorizes the use of speed cameras in school zones
  - <http://www.baltimorecountymd.gov/Agencies/police/speedcameras/>
  - See pdf

Links to Policy Examples:

- Maryland Code, Transportation, §21-809 Speed monitoring systems
  - See pdf
- Montgomery County Code, § 31-9A Speed Monitoring Systems Authorized (Bill 7-10)
  - See pdf
- Baltimore County Code, Article 18 Transportation, Title 4 Speed Monitoring Systems (Bill 61-09, § 1, 10-1-2009)
  - See pdf

*Note: 1 = meets criteria for policy effectiveness (consistent, positive outcomes from at least two high-quality experimental or quasi-experimental trials using a comparison group or interrupted time series design)^4^; 2 = consistent evidence available linking policy with positive outcomes from high-quality observational studies only; 3 = insufficient evidence available for policy or policy components.

† Be sure to check with your state, county, and municipal governments regarding potential existing laws that may impede any new policy development.

‡Local governments and organizations should check existing state and federal statutes and administrative codes for the authority to implement local policies.

References

1 Wilson, C, Willis, C, Hendrikz, JK, Bellamy N (2006) Speed enforcement detection devices for preventing road traffic injuries. *Cochrane Database of Systematic Reviews* 2006, 2, CD004607.

2 Heron, HP, Hoyert, DL, Murphy, SL, Xu, JQ, Kochanek, KD, Tejada-Vera, B (2009). Deaths: Final data for 2006. National Vital Statistics Report, 57(14), National Center for Health Statistics.

3 Pilkington, P, Kinra, S (2005). Effectiveness of speed cameras in preventing road traffic collisions and related casualties: systematic review. *British Medical Journal*, 330, 331-334.

4 Flay, BR, Biglan, A, Boruch, RF, Ganzalez Castro, F, Gottfredson, D, Kellam, S, Moscicki, EK, Schinke, S, Valentine, JC, & Ji, P (2005). Standards of evidence: Criteria for efficacy, effectiveness and dissemination. Prevention Science, 6(3), 151-175.

Early Childhood Health Promotion

Domain: Family Influences

Reasons for Policy:

- The factors that shape child health and development have implications for health in adulthood.^1^
- Health promotion efforts targeting tobacco smoke exposure, unintentional injuries, obesity and/or mental health among preschool-aged children and their families can improve health across the life span and yield future economic returns to society.^1^

Community Group:

- Local/state government
- Local health department
- Local school board
- Department of education

Policy Components:

- Health promotion and interventions targeting preschool age children and their families addressing tobacco exposure, unintentional injury, obesity, and/or mental health.

Desired Outcomes:

- Reduce exposure to environmental tobacco smoke
- Prevention of unintentional injuries
- Prevention of childhood obesity
- Improved overall lifetime health and well being

Level of Evidence Available to Evaluate Effectiveness of Policy (1 = strong evidence to 3 = insufficient evidence*):^2^

1 = Meets Criteria for Effectiveness

Achievable Results:

The following summary of achievable results is based on a published review of the scientific evidence.

On average, smoking cessation interventions targeting pregnant women can achieve:

- 25% to 30% reductions in parent-reported smoking at home or household air nicotine measures.^1^

On average, unintentional injury prevention targeting children and parents can achieve:

- Improvements in parents’ and children’s knowledge, safety behaviors, and safety device usage.^1^

Because very few studies target preschool-age children, the overall effectiveness of interventions to prevent obesity in early childhood remains unclear. Additional evidence is needed for promotion interventions for children’s mental health. There is a need for targeted, specific, rigorous research to examine the longitudinal causal relationships between early childhood preventative interventions and health outcomes across the life span.^1^

Community Examples:

- Colorado, Boulder County Public Health Department administers child health promotion services.
  - <http://www.bouldercounty.org/help/family/pages/chpservices.aspx> (see pdf)
- Washington State Department of Early Learning administers child development, health and safety programs.
  - <http://www.del.wa.gov/development/Default.aspx> (see pdf)

Links to Policy Examples:

- Colorado Revised Statutes § 25-1-506. County or district public health agency
  - See pdf
- Colorado Revised Statutes Title 25 Health, Article 20.5 Prevention, Intervention, and Treatment Services for Children and Youth
  - See pdf
- Revised Code of Washington, Title 43, Chapter 43.215 Department of Early Learning
  - See pdf

*Note: 1 = meets criteria for policy effectiveness (consistent, positive outcomes from at least two high-quality experimental or quasi-experimental trials using a comparison group or interrupted time series design)^5^; 2 = consistent evidence available linking policy with positive outcomes from high-quality observational studies only; 3 = insufficient evidence available for policy or policy components.

† Be sure to check with your state, county, and municipal governments regarding potential existing laws that may impede any new policy development.

‡Local governments and organizations may check existing state and federal statutes and administrative codes for the authority to implement local policies.

References

1Guyer B, et al. (2009). Early Childhood Health Promotion and Its Life Course Health Consequences. Academic Pediatrics, 9, 142-149.

2 Flay, BR, Biglan, A, Boruch, RF, Ganzalez Castro, F, Gottfredson, D, Kellam, S, Moscicki, EK, Schinke, S, Valentine, JC, & Ji, P (2005). Standards of evidence: Criteria for efficacy, effectiveness and dissemination. Prevention Science, 6(3), 151-175.

Later School Day Start Time

Domain: School Influences

Reasons for Policy:

- Inadequate sleep may lead to poor school performance.^1^
- Excessive sleepiness in adolescents can have a negative effect on cognition and mood.^2^
- Excessive sleepiness in teenagers has been associated with an increased risk of automobile crashes.^2^

Community Group:

- Local school district
- Local school board
- Local government

Policy Components:

- Later school-day start time

Desired Outcomes:

- Increased school attendance rates
- Improved academic performance
- Decreased school dropout rates

Level of Evidence Available to Evaluate Effectiveness of Policy (1 = strong evidence to 3 = insufficient evidence*):^3^

2 = Consistent evidence available linking policy with positive outcomes from high-quality observational

studies only.

Achievable Results:

The following summary of achievable results is based on a published review of the scientific evidence.

On average, later school start times can achieve:

- More total sleep on school nights for students attending schools with earlier start times.^1^
- Improved attendance rates among high school students.^1^
- Improved student attention and concentration.^1,2^
- Decreased dropout rate among high school students.^2^

Results suggest that later school-day start times have beneficial effects on student attendance and academic performance. However, more rigorous studies are needed.

Community Examples:

- Minneapolis Public Schools, Minnesota, changed high school start time from 7:20 a.m. to 8:40 a.m.
  - <http://www.npr.org/templates/story/story.php?storyId=6896471>
- Anderson County School System, Tennessee, approved delayed school start times
  - <http://images.pcmac.org/Uploads/AndersonCounty/AndersonCounty/Sites/News/Documents/AC%20Schools%20prepare%20for%20delayed%20start%20of%20school%20day.pdf>
  - <http://www2.acs.ac/?PN=Pages&SubP=Level1Page&L=2&DivisionID=5289&DepartmentID=5216&PageID=9133&ToggleSideNav>=

Links to Policy Examples:

- Minneapolis Public Schools Policies, §6132 School Day
  - <http://policy.mpls.k12.mn.us/uploads/6132.pdf>
- Anderson County School System Rules and Policies, 1.101 Role of the Board
  - <http://www.tsba.net/production/type.asp?iType=1&iBoard=83>

*Note: 1 = meets criteria for policy effectiveness (consistent, positive outcomes from at least two high-quality experimental or quasi-experimental trials using a comparison group or interrupted time series design)^3^; 2 = consistent evidence available linking policy with positive outcomes from high-quality observational studies only; 3 = insufficient evidence available for policy or policy components.

† Be sure to check with your state, county, and municipal governments regarding potential existing laws that may impede any new policy development.

‡Local governments and organizations may check existing state statutes and administrative codes for the authority to implement local policies.

References

1Wolfson AR, Carskadon MA (2003). Understanding adolescents’ sleep patterns and school performance: a critical appraisal. *Sleep Med Rev*., 7(6), 491-506.

2 Millman RP, et al. (2005). Excessive Sleepiness in Adolescents and Young Adults: Causes, Consequences, and Treatment Strategies. *Pediatrics*, 115, 1774-1786.

3 Flay, BR, Biglan, A, Boruch, RF, Ganzalez Castro, F, Gottfredson, D, Kellam, S, Moscicki, EK, Schinke, S, Valentine, JC, & Ji, P (2005). Standards of evidence: Criteria for efficacy, effectiveness and dissemination. *Prevention Science*, 6(3), 151-175.

Modified School Calendars

Domain: School Influences

Reasons for Policy:

- Long summer vacation breaks may lead to poor information retention and lower academic scores.^1^
- Children that speak a language other than English at home may lose the English skills they learned during the school year while on a long summer break.^1^
- The United States has one of the lowest number of school days among industrialized nations.^1^

Community Group:

- Local government
- School district
- Parent-Teacher Association (PTA)

Policy Components:

- Modified calendar schedule (e.g. 9 weeks on 3 weeks off, 12 weeks on 4 weeks off)
- Educational activities offered during intersession

Desired Outcomes:

- Improved information retention
- Increased achievement scores
- Improved attitude toward school
- Improved accessibility to remedial and/or enrichment programs during breaks

Level of Evidence Available to Evaluate Effectiveness of Policy (1 = strong evidence to 3 = insufficient evidence*):^2^

2= Consistent Evidence from Observational Studies Only

Achievable Results:

The following summary of achievable results is based on a published review of the scientific evidence.

On average, a modified school calendar can achieve:

- Small improvements in academic achievement scores (effect size: 0.11).^1^

More than 80% of teachers, parents, students, administrators and staff favor a modified school calendar.^1^

Community Examples:

- Fairfax County Public Schools (Virginia) instituted a modified school calendar in seven of its elementary schools.
  - http://www.fcps.edu/about/modifiedcal.htm
- Salt Lake City School District (Utah) allows schools to create a modified school calendar.
  - http://www.slc.k12.ut.us/schools/#special

Links to Policy Examples: †

- Fairfax County Public Schools Regulations 1345.1 and 1345.2
  - http://www.boarddocs.com/vsba/fairfax/Board.nsf/0/eaebbfbbda0e379985256fcd005aadd3/$FILE/P1345.pdf
  - http://www.boarddocs.com/vsba/fairfax/Board.nsf/0/99dd7a6a94e44572852571fd00659bd5/$FILE/R1345.pdf
- Salt Lake City School District Board Policy I-6
  - http://www.slc.k12.ut.us/board/policies/I/pdfs/i6.pdf
  - http://www.boarddocs.com/vsba/fairfax/Board.nsf/0/eaebbfbbda0e379985256fcd005aadd3/$FILE/P1345.pdf

*Note: 1 = meets criteria for policy effectiveness (consistent, positive outcomes from at least two high-quality experimental or quasi-experimental trials using a comparison group or interrupted time series design)^2^; 2 = consistent evidence available linking policy with positive outcomes from high-quality observational studies only; 3 = insufficient evidence available for policy or policy components.

† Be sure to check with your state, county, and municipal governments regarding potential existing laws that may impede any new policy development.

References

^1^Cooper H, Valentine JC, Charlton K, Melson A (2003). The effects of modified school calendars on student achievement and on school and community attitudes. *Review of Educational Research,* 73(1), 1-52.

^2^Flay, BR, Biglan, A, Boruch, RF, Ganzalez Castro, F, Gottfredson, D, Kellam, S, Moscicki, EK, Schinke, S, Valentine, JC, & Ji, P (2005). Standards of evidence: Criteria for efficacy, effectiveness and dissemination. Prevention Science, 6(3), 151-175.

School-Based Health Centers

Domain: School Influences

Reasons for Policy:

- Young people today face worse health than their parents did and meeting the health care needs of school-aged children remains a challenge.^1^
- More than two-thirds of the 11.2 million uninsured children and adolescents in the United States who were eligible for Medicaid or the SCHIP were not enrolled in 2000.^2^

Community Group:

- Local medical community (physicians, nurses, mental health counselors)
- Parent Teacher Association
- Local Health Department
- Private sector organizations
- Youth service organizations

Policy Components:

- Employ a combination of physicians, physician assistants, and nurse practitioners as well as mental health professionals and nursing staff.
- Provide a variety of primary preventive services, physical health services, and mental health and counseling services in a manner that is culturally sensitive, confidential, comfortable, and safe.
- Form advisory boards to involve the community in planning and advising.

Desired Outcomes:

- Provide health services to students with unmet health needs and inadequate health resources.

Level of Evidence Available to Evaluate Effectiveness of Policy (1 = strong evidence to 3 = insufficient evidence*):^3^

2= Consistent Evidence

Achievable Results:

The following summary of achievable results is based on a published review of the scientific evidence.

- SBHC’s effectively increases accessibility to health services by reaching a broad scope of eligible students. Between 58% and 75% of students eligible to receive services at SBHCs enroll and 72% of those enrolled use SBHC services.^4^
- More research is needed on the health outcomes achieved with SBHCs.^4^

Community Examples:

- Seattle Washington passed a levy that funds school health clinics in all high school and selected middle schools in the city.
  - <http://www.seattle.gov/neighborhoods/education/edlevy.htm>
  - <http://www.seattle.gov/neighborhoods/education/implementation&evaluation.pdf#page=12>
  - <http://www.kingcounty.gov/healthservices/health/child/yhs.aspx>
- New York City New York has 122 school-based health centers providing primary, preventive, first aid, and emergency care.
  - <http://schools.nyc.gov/Offices/Health/SBHC/SBHC.htm>

Links to Policy Examples:

- Seattle Washington, Ordinance # 121529 allocates $2,605,000 for school-based health clinics
  - <http://www.seattle.gov/neighborhoods/education/Ord121529FN.pdf>
- New York City New York: Regulation of the Chancellor Number A- 701 Section VII.
  - <http://docs.nycenet.edu/docushare/dsweb/Get/Document-313/A-701%20%206-19-08%20%20Formatted.pdf>

*Note: 1 = meets criteria for policy effectiveness (consistent, positive outcomes from at least two high-quality experimental or quasi-experimental trials using a comparison group or interrupted time series design)^5^; 2 = consistent evidence available linking policy with positive outcomes from high-quality observational studies only; 3 = insufficient evidence available for policy or policy components.

† Be sure to check with your state, county, and municipal governments regarding potential existing laws that may impede any new policy development.

References

^1^  Tylee, PA, Haller, DM, Graham, T, Churchill, R, & Sanci, LA (2007). Youth-friendly primary-care services: how are we doing and what more needs to be done? The Lancet, 369(9572), 1565-1573.

^2^ American Academy of Pediatrics. Children’s Health Insurance Advocacy Fact Sheets. Available at: http://www.aap.org/advocacy/facts00.htm.2000. Accessed October 12, 2002.

^3^ Flay, BR, Biglan, A, Boruch, RF, Ganzalez Castro, F, Gottfredson, D, Kellam, S, Moscicki, EK, Schinke, S, Valentine, JC, & Ji, P (2005). Standards of evidence: Criteria for efficacy, effectiveness and dissemination. Prevention Science, 6(3), 151-175.

^4^ Santelli, J,Morreale, M, Wigton, A, & Grason, H (1996). School health centers and primary care for adolescents: A review of the literature. Journal of Adolescent Health, 18, 357-366.

School Funding

Domain: School Influences

Reasons for Policy:

- Low-income students consistently fall behind high-income students in academic areas, such as test scores, graduation rates, and college enrollment.^1^
- Findings show that an increase in funding for low-income school districts can substantially benefit children in those areas.^1^
- Studies show that although increases in funding have occurred, only 25% of those funds have been used on regular instruction.^1^

Community Group:

- State government
- Local government
- Local school board

Policy Components:

- Have a low-income weight of 110-159% in educational funding equations (i.e. 2-2.5 times the cost for students who are not low-income)
- Use additional funds to establish/enhance programs that have been shown to improve achievement (e.g. class-size reduction, teacher training and education, recruitment and hiring of high-quality teachers, and early childhood education)

Desired Outcomes:

- Improved achievement and standardized test scores
- Improved achievement in reading and math
- Improved graduation rates
- Improved college enrollment rates

Level of Evidence Available to Evaluate Effectiveness of Policy (1 = strong evidence to 3 = insufficient evidence*):^3^

2 = Consistent Evidence Available

Achievable Results:

The following summary of achievable results is based on a published review of the scientific evidence.

On average, a $500 (10% of the national average) increase in per pupil expenditure can achieve:

- Large improvements student academic achievement (Effect Size: 0.70).^2^ However, results should be interpreted with caution, as most studies have been cross-sectional.

Community Examples:

- New York City Department of Education seeks to provide schools with adequate funding in order for students to achieve
  - See “Fair Funding“ pdf
- New York City Department of Education handbook on “fair funding”
  - See pdf

Links to Policy Examples: †

- Minneapolis Public School website describes how property tax levies work for the district
  - <http://www.mpls.k12.mn.us/228210129163622130/site/default.asp>
- Albany Unified School District, California
  - See PDF

*Note: 1 = meets criteria for policy effectiveness (consistent, positive outcomes from at least two high-quality experimental or quasi-experimental trials using a comparison group or interrupted time series design)^3^; 2 = consistent evidence available linking policy with positive outcomes from high-quality observational studies only; 3 = insufficient evidence available for policy or policy components.

† Be sure to check with your state, county, and municipal governments regarding potential existing laws that may impede any new policy development.

References

^1^Carey K (2002). Education funding and low-income children: A review of current research. *Center on Budget and Policy Priorities.*

^2^Hedges LV, Laine RD, Greenwald R (1994). An exchange: Part I: Does money matter? A meta-analysis of studies of the effects of differential school inputs on student outcomes. *Educational Researcher,* 23(3), 5-14.

^3^Flay, BR, Biglan, A, Boruch, RF, Ganzalez Castro, F, Gottfredson, D, Kellam, S, Moscicki, EK, Schinke, S, Valentine, JC, & Ji, P (2005). Standards of evidence: Criteria for efficacy, effectiveness and dissemination. Prevention Science, 6(3), 151-175.

School Gardens

Domain: School Influences

Reasons for Policy:

- The United States food environment often does not promote health.^1^
- Fewer than 10% of children age 4-13 years meet recommendations^2^ for daily fruit and vegetable consumption.^3^
- School gardens can create a more healthful food environment and educational opportunities.^4^
- School gardens teach through experience,^5^ and experiential learning stimulates higher orders of cognition.^6^
- School gardens can be socially inclusive of all ethnicity/income groups, allow parents who are less educated to become active in the school through aiding in the garden, and increase community involvement in the school.^4^

Community Group:

- School District
- City horticultural societies
- Community ecological experts
- Local farmers
- Health department
- University agricultural departments

Policy Components:

- Integrate hands-on garden experiences into classroom learning and dietary choices

Desired Outcomes:

- Increased academic achievement and engagement
- Improved attitudes and behaviors for healthy eating

Level of Evidence Available to Evaluate Effectiveness of Policy (1 = strong evidence to 3 = insufficient evidence*):^7^

2 = Consistent Evidence from Observational Studies Only

Achievable Results:

The following summary of achievable results is based on a published review of the scientific evidence.

On average, school garden programs may be associated with:

- Improved science scores^4^
- Improved elementary student preference for vegetables as snacks^4^
- Improved school attitude and pride^4^
- Improved teamwork, student bonding, interaction with adults and their community^4^

Findings are limited by few high-quality, experimental studies. More research is needed.

Community Examples:

- Lopez Island School District, Washington implements the L.I.F.E. (Lopez Island Farm Education) Garden program.
  - <http://www.lopezislandschool.org/programs/garden>
  - <http://agr.wa.gov/marketing/farmtoschool/schoolgardens.aspx>
- Carmel, California, Carmel Middle School supports a school garden program and garden science curriculum.
  - <http://www.csgn.org/page.php?id=130>
  - <http://www.csgn.org/>

Links to Policy Examples:

- Lopez Island School District, School Board Wellness Policy 6701
  - See pdf
- California Code, Education Code, Ch.10, Art 2, §9000-9004 Garden Programs
  - See pdf

*Note: 1 = meets criteria for policy effectiveness (consistent, positive outcomes from at least two high-quality experimental or quasi-experimental trials using a comparison group or interrupted time series design)^7^; 2 = consistent evidence available linking policy with positive outcomes from high-quality observational studies only; 3 = insufficient evidence available for policy or policy components.

† Be sure to check with your state, county, and municipal governments regarding potential existing laws that may impede any new policy development.

References

^1^ U.S. Department of Health and Human Services (2006). Healthy people 2010 midcourse review. Washington, DC: U.S. Government Printing Office.

^2^ US Department of Agriculture, Center for Nutrition Policy and Promotion. *MyPyramid Food Guidance System*. Washington, DC: Center for Nutrition Policy and Promotion; 2005.

^3^ Guenther PM, Dodd KW, Reedy J, Krebs-Smith SM. Most Americans eat much less than recommended amounts of fruits and vegetables. *J Am Diet Assoc*. 2006;106:1371-1379.

^4^ Blair, D (2009). The child in the garden: An evaluative review of the benefits of school gardening. The Journal of Environmental Education, 40(2), 15-38.

^5^ Bundschu-Mooney, E. (2003). *School garden investigation: Environmental awareness and education.* San Rafael, CA: Division

of Education, School of Business, Education and Leadership, Dominican University of California. (ERIC Document

Reproduction Service No. ED480981). Retrieved August 2, 2006, from <http://edres.org/eric/ED480981.htm>

^6^ Waliczek, T. M., Logan, P., & Zajicek, J. M. (2003). Exploring the impact of outdoor environmental activities on children

using a qualitative text data analysis system. *HortTechnology, 13*, 684–688.

^7^ Flay, BR, Biglan, A, Boruch, RF, Ganzalez Castro, F, Gottfredson, D, Kellam, S, Moscicki, EK, Schinke, S, Valentine, JC, & Ji, P (2005). Standards of evidence: Criteria for efficacy, effectiveness and dissemination. Prevention Science, 6(3), 151-175.

School Recess

Domain: School Influences

Reasons for Policy:

- There is a trend toward minimizing or eliminating recess breaks during the school day.^1^
- Recess may have a positive effect on children’s cognitive development.^1,2^
- Peer interaction and socialization may improve school performance and adjustment.^1^
- Recess breaks may foster greater attention to class work.^1,2^

Community Group:

- Individual school
- Local school district
- Local school board
- Local/state government

Policy Components:

- Recess breaks in primary school curriculum
- Outdoor or indoor recess breaks
- Game facility during recess breaks (ball games, chase, jumping and singing games)

Desired Outcomes:

- Greater attention to classroom tasks
- Improvements in school performance and achievement

Level of Evidence Available to Evaluate Effectiveness of Policy (1 = strong evidence to 3 = insufficient evidence*):^3^

2 = Consistent Evidence Available for High-quality Observational Studies Only

Achievable Results:

The following summary of achievable results is based on a published review of the scientific evidence.

- On average, children are more attentive to classroom tasks after recess.^1^ However, more research is needed.

Community Examples:

- DeKalb County Board of Education, Georgia instituted mandatory recess for grades K-5.
  - <http://beforeitsnews.com/story/130/589/DeKalb_County,_Georgia,_USA,_Joins_Other_School_Districts_Nationwide_That_Are_Restoring_Childrens_Right_to_Recess.html>
- Chicago, Illinois, McCormick Elementary School, implemented recess policy.
  - <http://healthyschoolscampaign.typepad.com/healthy_schools_campaign/2010/03/mccormick-elementary-school-success-with-recess.html>

Links to Policy Examples:

- DeKalb County Board of Education Policy, Unstructured Break Time
  - <https://eboard.eboardsolutions.com/ePolicy/Policy.aspx?Sch=4054&S=4054&PC=IEDA&revNo=1.17&srch=unstructured+break+time&ktype=Exact>
  - Official Code of Georgia Annotated, O.C.G.A. §20-2-323 (2010) Unstructured break time for students in kindergarten through grade eight
    - See pdf
- Chicago public Schools Policy Manual, §704.7, III, (C)
  - <http://policy.cps.k12.il.us/documents/704.7.pdf>

*Note: 1 = meets criteria for policy effectiveness (consistent, positive outcomes from at least two high-quality experimental or quasi-experimental trials using a comparison group or interrupted time series design)^3^; 2 = consistent evidence available linking policy with positive outcomes from high-quality observational studies only; 3 = insufficient evidence available for policy or policy components.

† Be sure to check with your state, county, and municipal governments regarding potential existing laws that may impede any new policy development.

References

1 Pellegrini AD, Bohn CM (2005). The Role of Recess in Children’s Cognitive Performance and School Adjustment. *Educational Researcher*, 34, 13-19.

2 Pellegrini AD, Bjorklund DF (1997). The role of recess in children’s cognitive performance. *Educational Psychologist*, 32:1, 35-40.

3 Flay, BR, Biglan, A, Boruch, RF, Ganzalez Castro, F, Gottfredson, D, Kellam, S, Moscicki, EK, Schinke, S, Valentine, JC, & Ji, P (2005). Standards of evidence: Criteria for efficacy, effectiveness and dissemination. Prevention Science, 6(3), 151-175.

Teacher Merit Pay

Domain: School Influences

Reasons for Policy:

- Close to 100% of public school teachers are paid by salary schedules.^1^
- Salary schedules pay by years of experience and education level, two variables which are weakly correlated with student outcomes.^2^

Community Group:

- Parent Teacher’s Association
- Local School Board
- Local Government
- Teacher’s Union

Policy Components:

- Policy approach varies by school district. In general, teacher pay plans should be linked to student achievement, professional evaluations, and professional growth activities.

Desired Outcomes:

- Improve teacher and administrator productivity
- Recruit more qualified teachers
- Improve student academic achievement

Level of Evidence Available to Evaluate Effectiveness of Policy (1 = strong evidence to 3 = insufficient evidence*):^3^

2= Inconsistent Evidence

Achievable Results:

The following summary of achievable results is based on a published review of the scientific evidence.

Incentive programs can have positive effects on teacher behavior.^4^ However, the scientific evidence is limited by a preponderance of observational studies. More research is needed to determine the magnitude of potential effects and identify the optimal configuration of performance incentives.

Community Examples:

- Houston Independent School District (Texas) implemented the ASPIRE program to recruit and retain high performing teachers.
  - http://portal.battelleforkids.org/ASPIRE/aspire/about_aspire.html?sflang=en
- Guilford County Schools (North Carolina) financed Mission possible, a teacher incentive program through a grant from the federal government.
  - http://www.guilford.k12.nc.us/depts/mission_possible/background.htm

Links to Policy Examples: †

- Houston Independent School District Policy # 01912
  - http://www.tasb.org/policy/pol/private/101912/pol.cfm?DisplayPage=DEAA(LOCAL).pdf
- Guilford County Schools
  - <http://www.gcsnc.com/policies/pdf/DD.pdf>
  - http://www.gcsnc.com/policies/pdf/DD_P.pdf

*Note: 1 = meets criteria for policy effectiveness (consistent, positive outcomes from at least two high-quality experimental or quasi-experimental trials using a comparison group or interrupted time series design)^3^; 2 = consistent evidence available linking policy with positive outcomes from high-quality observational studies only; 3 = insufficient evidence available for policy or policy components.

† Be sure to check with your state, county, and municipal governments regarding potential existing laws that may impede any new policy development.

References

1 Podgursky, M (2007). Teams versus bureaucracies: Personnel policy, wage-setting, and teacher quality in traditional public, charter, and private schools. In M. Berends, M.G. Springer, & H. Walberg (Eds.), Charter school outcomes. Mahwah, NJ: Lawrence Erlbaum Associates.

2 Hanuschek, E.A. (2003). The failure of input-based resource policies. Economic Journal, 113, F64-F68.

3 Flay, BR, Biglan, A, Boruch, RF, Ganzalez Castro, F, Gottfredson, D, Kellam, S, Moscicki, EK, Schinke, S, Valentine, JC, & Ji, P (2005). Standards of evidence: Criteria for efficacy, effectiveness and dissemination. Prevention Science, 6(3), 151-175.

4 Podgursky, M & Springer, MG (2007). Teacher performance pay: A review. Journal of Policy Analysis and Management, 26(4) 909-949.

Additional file 3b. Policy briefs for strategies with insufficient evidence to assess efficacy based on published reviews (Level 3).

Conditional Cash Transfer

Domain: Income & Resources

Reasons for Policy:

- Nationwide, 18% of all children live in poverty and African American and Latino children are disproportionately affected.^1^
- As low-income children can face the greatest resource disparities, CCT programs can help redistribute resources to reduce health inequities.^2^

Community Group:

- Neighborhood-based non-profit organizations (NBO’s)
- Local government
- Social Service Organizations
- Local school board
- Local health clinics

Policy Components:

- Monetary incentives given to very low income families who participate in specified education, health, and workforce activities that are focused on the well-being of children
- Family rewards given to parents for efforts and accomplishments in children’s education, family preventive health care practices, and parents’ employment
- Education rewards given directly to students for activities such as good attendance and passing standardized exams

Desired Outcomes:

- Short term reductions in poverty through immediate cash payments
- Long-term reductions in poverty by building human capital
- Increased use of preventive health services
- Improved school attendance and academic achievement

Level of Evidence Available to Evaluate Effectiveness of Policy (1 = strong evidence to 3 = insufficient evidence*):^3^

3=Insufficient Evidence

Achievable Results:

The following summary of achievable results is based on a published review of the scientific evidence.

CCT’s have a positive impact on the use of health services^2,4^

CCT’s can improve growth and nutritional status in children^2,4^

Studies assessing CCT programs have occurred predominantly in low- to middle-income countries. More

research is needed to determine the efficacy of CCT’s in high-income countries, such as the U.S. and the U.K.

Community Examples:**

- New York City, NY recently ended its Opportunity NYC program which offered various CCT programs to individuals in the community.
  - <http://www.opportunitynyc.net/whatsnext>
- Greensboro, NC, College Bound Sisters is a nonprofit program administered through the University of North Carolina. The program awards young women with a dollar a day in a college savings account as long as they stay pregnancy free
  - <http://nursing.uncg.edu/cbs/index.html>
  - see pdf
- The Mexican Federal Government administers, Opotunidades, a direct cash transfer program to help support families living in extreme poverty.
  - <http://www.oportunidades.gob.mx/Portal/wb/Web/oportunidades_a_human_development_program>

Links to Policy Examples†:

None available.

*Note: 1 = meets criteria for policy effectiveness (consistent, positive outcomes from at least two high-quality experimental or quasi-experimental trials using a comparison group or interrupted time series design)^3^; 2 = consistent evidence available linking policy with positive outcomes from high-quality observational studies only; 3 = insufficient evidence available for policy or policy components.

**Note: In the United States, CCT programs are usually privately funded nonprofit 501(c)(3) tax exempt organizations. CCT programs also seem to be more prevalent in developing countries.

† Be sure to check with your state, county, and municipal governments regarding potential existing laws that may impede any new policy development.

References

^1^ Aber, LJ (2009). A big new investment in America’s poorest and youngest children: Conditional cash transfers. Big Ideas for Children: Investing in our Nation’s Future, First Focus. Retrieved from: <http://www.firstfocus.net/Download/18-Aber.pdf>.

^2^ Lagarde, M, Haines, A, & Palmer, N (2007). Conditional cash transfers for improving uptake of health interventions in low- and middle-income countries: A systematic review. JAMA, 298 (16), 1899-1910.

^3^ Flay, BR, Biglan, A, Boruch, RF, Ganzalez Castro, F, Gottfredson, D, Kellam, S, Moscicki, EK, Schinke, S, Valentine, JC, & Ji, P (2005). Standards of evidence: Criteria for efficacy, effectiveness and dissemination. Prevention Science, 6(3), 151-175.

^4^ Lagarde, M, Haines, A, & Palmer, N (2009). The impact of conditional cash transfers on health outcomes and use of health services in low and middle income countries. Cochrane Database, Issue, 4.

Home Nursing Services for Children

Domain: Income & Resources

Reasons for Policy:

- Hospitalization of a child can be very stressful for the child and the parents.^1^
- Many times, children in the ER present with non-urgent illnesses and do not have primary care providers.^1^

Community Group:

- Local government
- Local public health department
- Local non-profit organizations

Policy Components:

- Specialized home-based nursing services
- Target children with acute or chronic illnesses

Desired Outcomes:

- Reduction in hospitalization of children
- Reduction in visits to ER
- Reduced health care costs
- Increased health and health care satisfaction

Level of Evidence Available to Evaluate Effectiveness of Policy (1 = strong evidence to 3 = insufficient evidence*):^2^

3 = insufficient evidence available

Achievable Results:

The following summary of achievable results is based on a published review of the scientific evidence.

- The evidence of effectiveness for these programs is mixed. Some studies suggest that home-based nursing services provided to children with acute and/or chronic illness are not effective in reducing hospital admissions or presentations to the emergency department, while others suggest that these services can improve parental satisfaction, reduce anxiety, and improve family functioning, psychological adjustment and school attendance. More research is needed.^1^

*Note: 1 = meets criteria for policy effectiveness (consistent, positive outcomes from at least two high-quality experimental or quasi-experimental trials using a comparison group or interrupted time series design)^2^; 2 = consistent evidence available linking policy with positive outcomes from high-quality observational studies only; 3 = insufficient evidence available for policy or policy components.

References

^1^Cooper C, Wheeler DM, Woolfenden S, Boss T, Piper S. Specialist home-based nursing services for children with acute and chronic illnesses. Cochrane Database of Systematic Reviews 2006, Issue 4. Art. No.: CD004383.

^2^Flay, BR, Biglan, A, Boruch, RF, Ganzalez Castro, F, Gottfredson, D, Kellam, S, Moscicki, EK, Schinke, S, Valentine, JC, & Ji, P (2005). Standards of evidence: Criteria for efficacy, effectiveness and dissemination. Prevention Science, 6(3), 151-175.

Title: Housing Improvement

Domain: Income & Resources

Reasons for Policy:

- A diversity of factors associated with housing conditions are found to produce a range of effects upon the health of the public, including physical, mental and social realms.^1^
- Poor housing conditions disproportionately impact low-income families.^1^
- Health consequences from low quality housing can include increased allergies, respiratory disease,^2,3^ traumatic injuries^1^, heat/cold related mortality^4^, impaired neurological development,^5^ anxiety and depression.^6^
- Childhood housing conditions have been linked to subsequent adult mortality.^7^

Community Group:

- Local Housing Authorities
- Code Enforcement
- Local Government
- Public Health Department
- Local Builders/Contractors

Policy Components:

- Building code addressing relevant areas to health and safety
- Screening low-income housing for toxins (radon, lead, carbon monoxide)
- Installing proper heating and insulation in housing for temperature and mold control
- Inspecting housing for a variety of safety measures, such as railings, fire-rated materials, etc.

Desired Outcomes:

- Eliminate health hazards in homes
- Stop growth of mold in homes
- Reduce indoor heat/cold exposures
- Reduce exposures to household toxins
- Reduce prevalence and severity of childhood asthma
- Reduce traumatic injuries in the home from falls or fires

Level of Evidence Available to Evaluate Effectiveness of Policy (1 = strong evidence to 3 = insufficient evidence*):

3 = Insufficient Evidence

Achievable Results:

The following summary of achievable results is based on a published review of the scientific evidence.^8^

Housing Improvements can achieve:

- Significant improvements in overall mental and physical health^9^
- Reductions in respiratory disease and improved lung function ^1,7^

Please note that these results are based on studies limited by small study populations, poor research designs, and lack of controlling for confounders. They should be interpreted with caution.

Community Examples:

- Lapeer Michigan has instituted a Housing Improvement Department that oversees housing quality and improvements pursuant to the city housing code.
  - http://www.ci.lapeer.mi.us/housing.htm
- Fresno California provides funds to low-income citizens who need to make repairs and improvements on their home.
  - http://www.fresno.gov/Government/DepartmentDirectory/PlanningandDevelopment/Housing/HousingRehabilitation/HomeImprovementProgram.htm

Links to Policy Examples:

- Lapeer Michigan City Ordinance: Chapter 9: Housing
  - http://www.ci.lapeer.mi.us/documents/Chapter%2009%20-%20Housing.pdf
- Fresno California Municipal Code: Chapter 8, Article V, section 8-2-502
  - [www.municode.com](http://www.municode.com)

Be sure to check with your state, county, and municipal governments regarding potential existing laws that may impede any new policy development.

*Note: 1 = meets criteria for policy effectiveness (consistent, positive outcomes from at least two high-quality experimental or quasi-experimental trials using a comparison group or interrupted time series design)^5^; 2 = consistent evidence available linking policy with positive outcomes from high-quality observational studies only; 3 = insufficient evidence available for policy or policy components.

References

^1^Shaw, M (2004). Housing and Public Health. Annual Reviews in Public Health, 25: 379-418.

^2^ Peat, J, Dickerson, J, & Li, J (1998). Effects of damp and mould in the home respiratory health: A review of the literature. Allergy, 53:120-128.

^3^ Blane, D, Mitchell, R, & Bartley, M (2000). The “inverse housing law” and respiratory health. Journal of Epidemiology and Community Health, 54: 745-749.

^4^ Gemell, I (2001). Indoor heating, house conditions, and health. Journal of Epidemiology and Community Health, 55: 928-929.

^5^ Meyer, P, NcGeehin, M, & Falk, H (2003). A global approach to childhood lead poisoning prevention. International Journal of Hygiene and Environmental Health, 206:363-369.

^6^ Hopton, J (1996). Housing conditions and mental health in a disadvantaged area in Scotland. Journal of Epidemiology and Community Health, 50: 56-61.

^7^ Dedman , D, Gunnell, D, Davey Smith, G, Frankel, S (2001). Childhood housing conditions and later mortality in the Boyd Orr cohort. Journal of Epidemiology and Community Health, 55: 10-15.

^8^ Flay, BR, Biglan, A, Boruch, RF, Ganzalez Castro, F, Gottfredson, D, Kellam, S, Moscicki, EK, Schinke, S, Valentine, JC, & Ji, P (2005). Standards of evidence: Criteria for efficacy, effectiveness and dissemination. Prevention Science, 6(3), 151-175.

^9^ Thomson, H, Petticrew, M, Morrison, D (2001). Health effects of housing improvement: Systematic review of intervention studies. British Medical Journal, 323:187-190.

In-community Alcohol and Drug Abuse Treatment Centers

Domain: Income & Resources

Reasons for Policy:

- There is a high correlation between criminal activity and drug use; more than 50% of those arrested tested positive for use of at least one illegal drug.^1^
- Offenders who were part of a therapeutic community drug treatment program showed less drug use and criminal activity compared to those that did not.^1^

Community Group:

- Local government
- Law enforcement
- Justice system
- Local public health departments

Policy Components:

- 6-12 month in-prison treatment program
- Community aftercare program after release
- Include individual and group therapy, 12-step meetings, addiction education, life skills training, and vocational counseling and placement both in-prison and after release

Desired Outcomes:

- Lower rates of recidivism
- Lower rates of re-arrest

Level of Evidence Available to Evaluate Effectiveness of Policy (1 = strong evidence to 3 = insufficient evidence*):

3 = insufficient evidence available

Achievable Results:

The following summary of achievable results is based on a published review of the scientific evidence.

- The evidence of effectiveness for these programs is mixed and limited by studies with poor scientific merit. More research is needed.

*Note: 1 = meets criteria for policy effectiveness (consistent, positive outcomes from at least two high-quality experimental or quasi-experimental trials using a comparison group or interrupted time series design)^2^; 2 = consistent evidence available linking policy with positive outcomes from high-quality observational studies only; 3 = insufficient evidence available for policy or policy components.

References

^1^Chanhatasilpa C, MacKenzie DL, Hickman LJ (2000). The effectiveness of community-based programs for chemically dependent offenders: A review and assessment of the research. *Journal of Substance Abuse Treatment,* 19, 383-393.

^2^Flay, BR, Biglan, A, Boruch, RF, Ganzalez Castro, F, Gottfredson, D, Kellam, S, Moscicki, EK, Schinke, S, Valentine, JC, & Ji, P (2005). Standards of evidence: Criteria for efficacy, effectiveness and dissemination. Prevention Science, 6(3), 151-175.

Title: Mixed-Income Housing

Domain: Income & Resources

Reasons for Policy:

- Spatial segregation of housing by income, race, or social class often results in unsafe neighborhoods of concentrated poverty^1^
- High poverty neighborhoods can have serious negative consequences for the well-being and life chances of children^2^
- The recent spread of socioeconomic segregation has lead to the physical and social deterioration of neighborhoods, resulting in reduced safety and health of the residents^1^

Community Group:

- Local government
- Local rental property owners/landlords
- Local housing authorities

Policy Components:

- “Mixed Income Housing” is a publicly subsidized multifamily rental housing development that exists within poverty neighborhoods, with a deliberate mixing of income groups
- Development may be new construction or conversion of existing buildings

Desired Outcomes:

- Decreased socioeconomic residential housing segregation
- Decreased exposure to crimes against person and property
- Decreased neighborhood social disorder
- Improved child and adolescent mental and physical health

Level of Evidence Available to Evaluate Effectiveness of Policy (1 = strong evidence to 3 = insufficient evidence*):^3^

3= Insufficient Evidence^1^

Community Stories:

- Community example (Note: Emilia will search for communities that have implemented policy; if any examples are provided in the article, provide a note here)
- Community example

Policy Examples:

- Websites (Emilia will search for examples of exact wording of policies)
- Websites

*Note: 1 = meets criteria for policy effectiveness (consistent, positive outcomes from at least two high-quality experimental or quasi-experimental trials using a comparison group or interrupted time series design)^3^; 2 = consistent evidence available linking policy with positive outcomes from high-quality observational studies only; 3 = insufficient evidence available for policy or policy components.

References

^1^ Anderson, LM, St. Charles, J, Fullilove, MT, Scrimshaw, SC, Fielding, JE, Normand, J, & the Task Force on Community Preventive Services (2003). Providing affordable family housing and reducing residential segregation by income: A systematic review. American Journal of Preventive Medicine, 24(3S), S47-67.

^2^ Ellen, IG & Turner, MA (2003). Do neighborhoods matter and why? : Choosing a better life? Evaluating the moving to opportunity social experiment. (pg 313-338). Washington, DC: Urban Institute Press.

^3^ Flay, BR, Biglan, A, Boruch, RF, Ganzalez Castro, F, Gottfredson, D, Kellam, S, Moscicki, EK, Schinke, S, Valentine, JC, & Ji, P (2005). Standards of evidence: Criteria for efficacy, effectiveness and dissemination. Prevention Science, 6(3), 151-175.

Transportation Policies

Domain: Income & Resources

Reasons for Policy:

- Only 27% of students in grades 9-12 get the recommended amount of physical activity per week.^1^
- The direct costs of inactivity each year in the US are approximately $24 billion.^1^
- Approximately 200,000-300,000 premature deaths per year are due to physical inactivity.^1^
- Approximately 40% of children are inhibited from walking or cycling to school because of perceived traffic dangers.^2^

Community Group:

- Local government
- Local transportation departments

Policy Components:

- Making/improving bicycle lanes
- Adding bicycle racks on buses
- Require sidewalks in neighborhoods
- Subsidized transit passes
- Incentives for car pooling
- Increase cost of parking

Desired Outcomes:

- Improved air quality due to the reduced use of cars
- More participation in physical activity
- Lower rates of obesity and other related health concerns
- Lower rates of mental health problems

Level of Evidence Available to Evaluate Effectiveness of Policy (1 = strong evidence to 3 = insufficient evidence*):^3^

3 = insufficient evidence available

Achievable Results:

The following summary of achievable results is based on a published review of the scientific evidence.

- There is insufficient evidence on the effectiveness of transportation policies in increasing physical activity. More research is needed.^1^

*Note: 1 = meets criteria for policy effectiveness (consistent, positive outcomes from at least two high-quality experimental or quasi-experimental trials using a comparison group or interrupted time series design)^3^; 2 = consistent evidence available linking policy with positive outcomes from high-quality observational studies only; 3 = insufficient evidence available for policy or policy components.

References

^1^Heath G, et al. The effectiveness of urban design and land use and transport policies and practices to increase physical activity: a systematic review. Journal of Physical Activity and Health. 2006;3(Suppl 1):S55-S76.

^2^Giles-Corti B, Kelty SF, Zubrick SR, Villanueva KP (2009). Encouraging walking for transport and physical activity in children and adolescents: How important is built environment? Sports Medicine, 39(12), 995-1009.

^3^Flay, BR, Biglan, A, Boruch, RF, Ganzalez Castro, F, Gottfredson, D, Kellam, S, Moscicki, EK, Schinke, S, Valentine, JC, & Ji, P (2005). Standards of evidence: Criteria for efficacy, effectiveness and dissemination. Prevention Science, 6(3), 151-175.

Welfare-to-Work

Domain: Income & Resources

Reasons for Policy:

- Welfare Reform Act of 1996 converted the welfare system into a finite program built to provide short-term cash assistance and steer people quickly into jobs^1^
- Psychologists believe parental employment may benefit children by providing them with family role models who work and are self-sufficient and by introducing a regular schedule into the family routine^2^

Community Group:

- Local Welfare Agency
- Local Employers
- Local Government

Policy Components:

- Mandate a minimum 30 hours per week of work for mothers to receive welfare checks
- Provide community service work for those unable to find employment
- Supplement welfare income with work earnings to boost family income

Desired Outcomes:

- Improved academic, cognitive and behavioral development of children
- Increased family income
- Promote family self-sufficiency and break the intergenerational cycle of poverty

Level of Evidence Available to Evaluate Effectiveness of Policy (1 = meets criteria for effectiveness to 3 = insufficient evidence*):^3^

3 = Insufficient evidence

Achievable Results:

The following summary of achievable results is based on a published review of the scientific evidence.

On average, welfare-to-work programs show:

- Small effects on improved likelihood of participant employment (RR = 1.10, 95% CI: 1.01-1.20).^5^
- Small, positive effects on participants’ earnings (Hedges g = 0.043, 95% CI: 0.01-0.08).^5^
- Small reductions in welfare payments to participants (Hedges g = 0.04, 95% CI: 0.03-0.05).^5^

However, deleterious effects on children’s academic outcomes have also been found:

- Work-to-welfare programs show small, but significant, deleterious effects on children’s school performance (Effect Size: -0.11, p-value < .01), including increased likelihood of repeating a grade (Effect Size: 0.06, p-value < .05) and decreased likelihood of performing above average in school (Effect Size: -0.08, p-value < .01). Effects may be more pronounced if a child has a younger sibling.^4^

More research is needed to determine if the benefits outweigh the negative effects of these programs.

Community Examples:

- New Jersey’s “Work First” program provides comprehensive assistance to those applying for welfare while also promoting independence through self sufficiency.
  - <http://www.state.nj.us/humanservices/dfd/programs/workfirstnj/>
  - <http://www.state.nj.us/humanservices/dfd/programs/workfirstnj/wfnjhandbk_09.pdf>
- The Housing Authority of the City of Austin, Texas, Community Development Department’s Family Self-Sufficiency Program provides residents with the skills and services necessary to become economically self-sufficient.
  - <http://www.hacanet.org/services/>
  - <http://www.hacanet.org/services/self_sufficiency.php>

Links to Policy Examples†:

- New Jersey Administrative Code, Title 10, Chapter 90 establishes the Work First New Jersey program, which emphasizes personal responsibility and promotes self -sufficiency through work. §§(1)(g) of Chapter 90 charges county or municipal agencies with the responsibility of administering the program^‡^.
- The Housing Authority of the City of Austin was created under Texas Local Government Code 392. The Family Self-Sufficiency program was implemented by resolution #1611.

*Note: 1 = meets criteria for policy effectiveness (consistent, positive outcomes from at least two high-quality experimental or quasi-experimental trials using a comparison group or interrupted time series design)^3^; 2 = consistent evidence available linking policy with positive outcomes from high-quality observational studies only; 3 = insufficient evidence available for policy or policy components.

† Be sure to check with your state, county, and municipal governments regarding potential existing laws that may impede any new policy development.

‡Local governments and organizations may check existing state statutes and administrative codes for the authority to implement local policies.

References

^1^ Zaslow, MJ, Moore, KA, Brooks, JL, Morris, PA, Tout, K, Redd, ZA, & Emig, CA (2002). Experimental studies of welfare reform and children. The Future of Children in Children and Welfare Reform, 12(1), 79-95. Princeton University.

^2^ Morris, PA, Duncan, GJ, & Clark-Kauffman, EC (2005). Child well-being in an era of welfare reform: The sensitivity of transitions in development to policy change. Developmental psychology, 41(6), 919-932.

^3^ Flay, BR, Biglan, A, Boruch, RF, Ganzalez Castro, F, Gottfredson, D, Kellam, S, Moscicki, EK, Schinke, S, Valentine, JC, & Ji, P (2005). Standards of evidence: Criteria for efficacy, effectiveness and dissemination. Prevention Science, 6(3), 151-175.

^4^ Gennetian, LA, Duncan, G, Knox, V, Vargas, W, Clark-Kauffman, E, & London, AS (2002). How welfare policies affect adolescents’ school outcomes: A synthesis of evidence from experimental studies. Journal of Research on Adolescence, 14(4), 399-423.

^5^ Smeslund, G, Hagen, KB, Steiro, A, Johme, T, Dalsbo, K, & Rud, G (2006). Work programs for welfare recipients. Campbell Collaboration Library. Retreived April 1, 2010 from <http://www.ncchta.org/fullmono/mon219.pdf>

Collective or Community Kitchens

Domain: Social Cohesion

Reasons for Policy:

- In 2003, 36 million people lived in food insecurity in the United States, 13 million of those were children.^1^
- 14% of low-income groups have insufficient food.^1^
- Food insecurity is associated with a number of factors, including poverty and area of residence.^1^

Community Group:

- Local government
- Local non-profit organizations
- Local community centers

Policy Components:

- Providing large kitchens for community use
- Cooking classes
- Communal meal programs

Desired Outcomes:

- Improved social cohesion
- Improved diet
- Lower rates of obesity
- Improved access to safe, affordable, and nutritionally sound food

Level of Evidence Available to Evaluate Effectiveness of Policy (1 = strong evidence to 3 = insufficient evidence*)^2^:

3 = Insufficient evidence

Achievable Results:

The following summary of achievable results is based on a published review of the scientific evidence.

- There is insufficient evidence on the effects of collective kitchens on social cohesion and other health outcomes. More research is needed.^3^

*Note: 1 = meets criteria for policy effectiveness (consistent, positive outcomes from at least two high-quality experimental or quasi-experimental trials using a comparison group or interrupted time series design)^3^; 2 = consistent evidence available linking policy with positive outcomes from high-quality observational studies only; 3 = insufficient evidence available for policy or policy components.

† Be sure to check with your state, county, and municipal governments regarding potential existing laws that may impede any new policy development.

References

^1^Dubois, L, Farmer, A, Girard, M, Porcherie, M (2006). Family food insufficiency is related to overweight among preschoolers. *Social Science & Medicine,* 63, 1503-1516.

^2^Flay, BR, Biglan, A, Boruch, RF, Ganzalez Castro, F, Gottfredson, D, Kellam, S, Moscicki, EK, Schinke, S, Valentine, JC, & Ji, P (2005). Standards of evidence: Criteria for efficacy, effectiveness and dissemination. Prevention Science, 6(3), 151-175.

^3^Engler-Stringer, R, & Berenbaum, S (2005). Collective kitchens in Canada: A review of literature. *Canadian Journal of Dietetic Practice and Research,* 66(4), 246-251.

Community–Driven Development

Domain: Social Cohesion

Reasons for Policy:

- Community-driven development (CDD) is an important form of development assistance that gives community groups control of decisions and resources over assistance.^1^
- CDD may empower low-income people, enhance sustainability, improve efficiency and effectiveness, make development more inclusive, build social capital, and strengthen governance.^2^
- The World Bank has lent approximately $7 billion dollars for such projects.^1^

Community Group:

- Local government
- Community organizations
- State government

Policy Components:

- Active involvement of members of a defined community in aspects of project design and implementation.
- Community has direct control over key project decisions, including management of investment funds.
- Community is a beneficiary in the design and management of a development project.

Desired Outcomes:

- Improve project quality and performance.
- Improve the targeting of poverty programs.
- Better designed projects and targeted benefits.
- Improve project sustainability.

Level of Evidence Available to Evaluate Effectiveness of Policy (1 = strong evidence to 3 = insufficient evidence*):^3^

3= insufficient evidence

Achievable Results:

The following summary of achievable results is based on a published review of the scientific evidence.

- Further research is needed to evaluate the effects of community–driven development on the desired outcomes.^1^
- Because many such projects lack careful evaluations with good treatment and control groups, and with baseline and follow-up data, little is known about the impact of community-based projects.^1^
- Evidence suggests project targeting is not always effective, especially for development projects targeting low-income individuals within communities.^1^
- There is some evidence that community-based development projects create effective community infrastructure and improve welfare outcomes, but the evidence does not support that it is the actual participatory elements that are responsible for improved project outcomes.^1^
- Community-based development seems likely to be more effective in more cohesive and better managed communities.^1^
- The sustainability of community-based initiatives depends on an enabling institutional environment.^1^
- Because the success of community-based development is conditioned by local cultural and social systems, projects are best done with careful learning by doing.^1^
- More research is needed.

*Note: 1 = meets criteria for policy effectiveness (consistent, positive outcomes from at least two high-quality experimental or quasi-experimental trials using a comparison group or interrupted time series design)^3^; 2 = consistent evidence available linking policy with positive outcomes from high-quality observational studies only; 3 = insufficient evidence available for policy or policy components.

† Be sure to check with your state, county, and municipal governments regarding potential existing laws that may impede any new policy development.

References

1 Mansuri, G, Vijayendra, R (2004). Community-Based and –Driven Development: A Critical Review. *The World Bank Observer*, 19(1), 1-39.

2 Doniger, P, et al. (2001). Community Driven Development. In World Bank, *Poverty Reduction Strategy Paper Sourcebook*, Vol. 1.

3 Flay, BR, Biglan, A, Boruch, RF, Ganzalez Castro, F, Gottfredson, D, Kellam, S, Moscicki, EK, Schinke, S, Valentine, JC, & Ji, P (2005). Standards of evidence: Criteria for efficacy, effectiveness and dissemination. Prevention Science, 6(3), 151-175.

Enterprise Zones

Domain: Social Cohesion

Reasons for Policy:

- Originally, enterprise zones were designed to attract businesses to low-income or underdeveloped areas.^1^
- In 2008, there were approximately 3,000 enterprise zones.^1^

Community Group:

- State government
- Local government

Policy Components:

- Property and income tax abatements
- Capital and labor subsidies
- Job training assistance
- Infrastructure improvements

Desired Outcomes:

- Increased employment opportunities for people living in the distressed areas
- Increased number of businesses in economically distressed areas
- Higher income and lower poverty levels for families residing within the enterprise zone

Level of Evidence Available to Evaluate Effectiveness of Policy (1 = strong evidence to 3 = insufficient evidence*):

3 = insufficient evidence

Achievable Results:

The following summary of achievable results is based on a published review of the scientific evidence.

- The effectiveness of these programs is mixed. However, generally studies have found that enterprise zones are not effective in helping distressed areas economically.^1^ More research is needed.

*Note: 1 = meets criteria for policy effectiveness (consistent, positive outcomes from at least two high-quality experimental or quasi-experimental trials using a comparison group or interrupted time series design)^2^; 2 = consistent evidence available linking policy with positive outcomes from high-quality observational studies only; 3 = insufficient evidence available for policy or policy components.

References

^1^Greenbaum RT, Landers J (2009). Why are state policy makers still proponents of enterprise zones? What explains their action in the face of a preponderance of the research? *International Regional Science Review,* 32, 466-479.

^2^Flay, BR, Biglan, A, Boruch, RF, Ganzalez Castro, F, Gottfredson, D, Kellam, S, Moscicki, EK, Schinke, S, Valentine, JC, & Ji, P (2005). Standards of evidence: Criteria for efficacy, effectiveness and dissemination. Prevention Science, 6(3), 151-175.

Independent Living Programs for Young People Leaving the Care System

Domain: Social Cohesion

Reasons for Policy:

- In 2003, there were 523,000 children in public care in the United States.^1^
- In the U.S., public (foster) care placement types include homes of family members, homes of nonfamily members, group homes or institutions, pre-adoptive homes, and other placements.^1^
- In the U.S., about 20,000 young people leave the public care system each year. A significant number of young people leaving care are disadvantaged and ill-prepared for adult life.^1^

Community Group:

- Local government
- Local non-profit organization
- State government
- State child and family agency
- Federal government

Policy Components:

- Independent living programs containing training and/or support for personal development

Desired Outcomes:

- Improved educational attainment (high school diploma, vocational diploma, higher education)
- Increased employment
- Improved health status
- Stable housing

Level of Evidence Available to Evaluate Effectiveness of Policy (1 = strong evidence to 3 = insufficient evidence*):^2^

3 = Insufficient Evidence

Achievable Results:

The following summary of achievable results is based on a published review of the scientific evidence.

- No randomized or quasi-randomized controlled studies met the inclusion criteria for this review. Other studies utilizing other designs had weak methodology and were insufficient to draw conclusions for policy. Further research is needed regarding individual living programs to determine the effectiveness of such programs on health outcomes.^1^

*Note: 1 = meets criteria for policy effectiveness (consistent, positive outcomes from at least two high-quality experimental or quasi-experimental trials using a comparison group or interrupted time series design)^2^; 2 = consistent evidence available linking policy with positive outcomes from high-quality observational studies only; 3 = insufficient evidence available for policy or policy components.

† Be sure to check with your state, county, and municipal governments regarding potential existing laws that may impede any new policy development.

‡Local governments and organizations may check existing state and federal statutes and administrative codes for the authority to implement local policies.

References

1 Donkoh C, Underhill K, Montgomery P (2006). Independent living programmes for improving outcomes for young people leaving the care system. Campbell Systematic Reviews 2006, 8, DOI: 10.4073/csr.2006.8.

2 Flay, BR, Biglan, A, Boruch, RF, Ganzalez Castro, F, Gottfredson, D, Kellam, S, Moscicki, EK, Schinke, S, Valentine, JC, & Ji, P (2005). Standards of evidence: Criteria for efficacy, effectiveness and dissemination. Prevention Science, 6(3), 151-175.

Complete Streets

Domain: Physical Environment

Reasons for Policy:

- In the last 15 years, more than 76,000 Americans have been killed while crossing or walking along a street.^1^
- In the U.S., an estimated 200,000 to 300,000 premature deaths occur each year due to physical inactivity.^2^
- A complete streets policy ensures that streets and roadways are designed with all users in mind, including bicyclists, pedestrians, and public transportation vehicles.^3^
- Complete streets reduce crashes and improve pedestrian safety through comprehensive safety improvements.^3^
- Complete streets provide opportunities for increased physical activity by incorporating features that promote walking and cycling.^3^

Community Group:

- Local government
- Local planning boards
- State government
- Transportation agencies

Policy Components:

- Features include streets and roadways that include sidewalks, bike lanes, special bus lanes, accessible transit stops, safe crossing opportunities, median islands, accessible pedestrian signals, and curb extensions
- Street-scale urban design which include redesigned streets, improved street lighting, features that increase ease and safety of street crossing, sidewalk continuity, enhanced aesthetics of the street area, and traffic calming features (enter islands, mini-roundabouts, raised crosswalks)

Desired Outcomes:

- Improved pedestrian safety
- Decreased pedestrian and cyclist morbidity and mortality
- Increased physical activity
- Decreased transportation costs
- Reduction in pollution and automobile emissions

Level of Evidence Available to Evaluate Effectiveness of Policy (1 = strong evidence to 3 = insufficient evidence*):^4^

3 = Insufficient evidence available

Achievable Results:

The following summary of achievable results is based on a published review of the scientific evidence.

- There is evidence linking complete streets to improved traffic safety.^2^ However, there are no systematic

reviews or meta-analyses available. Additional research is needed to evaluate the effects of complete

streets on other desired health outcomes.

*Note: 1 = meets criteria for policy effectiveness (consistent, positive outcomes from at least two high-quality experimental or quasi-experimental trials using a comparison group or interrupted time series design)^4^; 2 = consistent evidence available linking policy with positive outcomes from high-quality observational studies only; 3 = insufficient evidence available for policy or policy components.

† Be sure to check with your state, county, and municipal governments regarding potential existing laws that may impede any new policy development.

‡Local governments and organizations may check existing state and federal statutes and administrative codes for the authority to implement local policies.

References

1 Ernst, M, Shoup, L (2009). Dangerous by Design: Solving the Epidemic of Preventable Pedestrian Deaths (and Making Great Neighborhoods). Surface Transportation Policy Partnership and Transportation for America. Retrieved September 27, 2010, from <http://t4america.org/resources/dangerousbydesign/>.

2 Heath, GW, Brownson RC, Kruger, J, Miles, R, Powell KE, Ramsey LT, and the Task Force on Community Preventive Services (2006). The Effectiveness of Urban Design and Land Use and Transport Policies and Practices to Increase Physical Activity: A Systematic Review. *Journal of Physical Activity and Health*, 3, Spp 1, S55-S76.

3 National Complete Streets Coalition. (2010). In Complete Streets Fundamentals. Retrieved September 27, 2010, from <http://www.completestreets.org/complete-streets-fundamentals/>.

4 Flay, BR, Biglan, A, Boruch, RF, Ganzalez Castro, F, Gottfredson, D, Kellam, S, Moscicki, EK, Schinke, S, Valentine, JC, & Ji, P (2005). Standards of evidence: Criteria for efficacy, effectiveness and dissemination. Prevention Science, 6(3), 151-175.

Condom Availability in Schools

Domain: Physical Environment

Reasons for Policy:

- In 2003, 34.3% of adolescents were sexually active^1^
- In 2003, 46.7% of adolescents had sexual intercourse at least once^1^
- In 2003, 14.4% of adolescents had 4 or more sexual partners^1^
- Schools are one of the best ways to reach adolescents because nearly 95% of youth aged 5-17 years are enrolled.^2^

Community Group:

- Local government
- Local school district

Policy Components:

- Have condoms available in schools without restrictions (e.g. in an open basket in school health center)

Desired Outcomes:

- Lower rates of teenage STIs and pregnancy
- Lower rates of sexual risk behaviors
- Higher rates of safer sex practices

Level of Evidence Available to Evaluate Effectiveness of Policy (1 = strong evidence to 3 = insufficient evidence*):^3^

3 = insufficient evidence available

Achievable Results:

The following summary of achievable results is based on a published review of the scientific evidence.

- The evidence of effectiveness for these programs is mixed and limited by studies with poor scientific merit. More research is needed.^2^

*Note: 1 = meets criteria for policy effectiveness (consistent, positive outcomes from at least two high-quality experimental or quasi-experimental trials using a comparison group or interrupted time series design); 2 = consistent evidence available linking policy with positive outcomes from high-quality observational studies only; 3 = insufficient evidence available for policy or policy components.

References

^1^Ahern NR & Kiehl EM (2006). Adolescent sexual health & practice-A review of the literature: Implications for healthcare providers, educators, and policy makers. *Family Community Health,* 29(4), 299-313.

^2^Kirby D (2002). The impact of schools and school programs upon adolescent sexual behavior. *The Journal of Sex Research,* 39(1), 27-33.

^3^Flay, BR, Biglan, A, Boruch, RF, Ganzalez Castro, F, Gottfredson, D, Kellam, S, Moscicki, EK, Schinke, S, Valentine, JC, & Ji, P (2005). Standards of evidence: Criteria for efficacy, effectiveness and dissemination. Prevention Science, 6(3), 151-175.

Enhanced Enforcement Gun Laws

Domain: Physical Environment

Reasons for Policy:

- Approximately 70% of reported homicides in the United States involve the use of firearms.^1^
- Among the identified firearms used in homicides, handguns comprised 88.3%. ^1^
- The total cost of gun violence in the U.S. ranges from at least $6-12 billion per year, but could be as high as $80 billion per year.^2^

Community Group:

- Local law enforcement
- State law enforcement
- Federal law enforcement

Policy Components:

- Gun detection patrols in high crime areas (directed or saturation patrols and roadblock checkpoints)
- Enhanced surveillance of probationers and parolees
- Weapon reporting hotlines
- Consent searches
- Searches of school lockers

Desired Outcomes:

- Reduction of illegal gun possession and carrying
- Reduction and prevention of gun related crime

Level of Evidence Available to Evaluate Effectiveness of Policy (1 = strong evidence to 3 = insufficient evidence*):^3^

3 = insufficient evidence

Achievable Results:

The following summary of achievable results is based on a published review of the scientific evidence.

- Directed patrols focused on illegal gun carrying may reduce gun violence at high-risk places and times. However, evidence is limited by the small number of available studies, variability in study design and analysis, and the absence of randomized trials. More research is needed.^2^
- Directed patrol is an aggressive policing strategy that raises concerns about legality, racial profiling, and community relations.^2^
- There is insufficient evidence to support other policy components and strategies of enhanced enforcement.^2^

*Note: 1 = meets criteria for policy effectiveness (consistent, positive outcomes from at least two high-quality experimental or quasi-experimental trials using a comparison group or interrupted time series design)^3^; 2 = consistent evidence available linking policy with positive outcomes from high-quality observational studies only; 3 = insufficient evidence available for policy or policy components.

† Be sure to check with your state, county, and municipal governments regarding potential existing laws that may impede any new policy development.

References

1 United States Department of Justice, Federal Bureau of Investigation. (2009). *Crime in the United States, 2008.* Retrieved August 3, 2010, from <http://www.fbi.gov/ucr/cius2008>.

2 Koper, CS, Mayo-Wilson, E, (2006). Police crackdowns on illegal gun carrying: a systematic review of their impact on gun crime. *Journal of Experimental Criminology,* 2, 227-261.

3 Flay, BR, Biglan, A, Boruch, RF, Ganzalez Castro, F, Gottfredson, D, Kellam, S, Moscicki, EK, Schinke, S, Valentine, JC, & Ji, P (2005). Standards of evidence: Criteria for efficacy, effectiveness and dissemination. Prevention Science, 6(3), 151-175.

Firearm Policies

Domain: Physical Environment

Reasons for Policy:

- In 2007, 31,224 persons died from firearm injuries in the United States.^1^
- In the U.S., firearm injuries account for over 17% of all injury deaths.^1^
- In the U.S., the cost of firearm related violence is approximately $100 billion per year.^2^
- Among 26 high-income nations, U.S. firearm death rates exceeded those of all other nations.^2^

Community Group:

- Local government
- State government
- Federal government

Policy Components:

- Prohibition of certain categories of firearms or ammunition that are particularly dangerous or not well suited for hunting or self-defense.
- Child access prevention laws (CAP)
- Firearm registration and licensing
- Firearm waiting periods / background checks

Desired Outcomes:

- Reduction of violent crimes (homicide, aggravated assault, robbery and rape)
- Reduction of unintentional firearm related injury
- Reduction in suicide
- Prevention of firearm related injury and death

Level of Evidence Available to Evaluate Effectiveness of Policy (1 = strong evidence to 3 = insufficient evidence*):^3^

3= insufficient evidence available for policy or policy components

Achievable Results:

The following summary of achievable results is based on a published review of the scientific evidence.

- Evidence is insufficient to determine whether the degree of firearms regulation is associated with decreased violence.^2^
- Further research is needed to determine the relationship between specific types and degree of firearm regulation and the rates of violence within a given jurisdiction.^2^
- Conclusions that evidence for the effectiveness of a firearms law is insufficient does not imply the law has no effect, rather, what effect, if any, the law has on an outcome is not yet known.^2^

*Note: 1 = meets criteria for policy effectiveness (consistent, positive outcomes from at least two high-quality experimental or quasi-experimental trials using a comparison group or interrupted time series design)^3^; 2 = consistent evidence available linking policy with positive outcomes from high-quality observational studies only; 3 = insufficient evidence available for policy or policy components.

† Be sure to check with your state, county, and municipal governments regarding potential existing laws that may impede any new policy development.

References

1 Xu, J, Kochanek KD, Murphy, SL, Tejada-Vera, B (2010). Deaths: Final Data for 2007. National Vital Statistics Reports web release, National Center for Health Statistics, 58(19), retrieved from <http://www.cdc.gov/nchs/deaths.htm>.

2 Hahn RA, et al. (2005). Firearms Laws and the Reduction of Violence: A Systematic Review. *American Journal of Preventive Medicine*, 28(2S1), 40-71.

3 Flay, BR, Biglan, A, Boruch, RF, Ganzalez Castro, F, Gottfredson, D, Kellam, S, Moscicki, EK, Schinke, S, Valentine, JC, & Ji, P (2005). Standards of evidence: Criteria for efficacy, effectiveness and dissemination. Prevention Science, 6(3), 151-175.

Food Taxes and Subsidies

Domain: Physical Environment

Reasons for Policy:

- Americans consume too much dietary fat and sugar and not enough fruits and vegetables, which is associated with higher body mass index (BMI) and obesity rates.^1^
- Obesity-related medical expenditures were estimated to be $92.6 billion (in 2002 dollars), with taxpayers covering approximately one-half the costs.^2^
- Good taste, high convenience, and the low cost of energy-dense foods, in conjunction with large portions and low satiating power, may be the principal reasons for overeating and weight gain.^3^
- Financial disparities in access to healthier diets may help explain why the highest rates of obesity and diabetes are found among racial/ethnic minorities and the working poor.^3^

Community Group:

- Local Government
- Local food outlets (restaurants, fast food, convenience stores, supermarkets)

Policy Components:

- Add substantial taxes to certain foods and beverages high in calories, fat, salt, or sugars, and/or low in nutrients
- Use taxes generated to subsidize the purchase of healthy foods (fruits and vegetables), especially for low income populations, and enhance the health-promoting environment in low-income communities
- Implement strategies at all levels of food access including the school, workplace, restaurant, supermarkets, and corner stores

Desired Outcomes:

- Decrease consumption of unhealthy, energy dense foods and increase consumption of healthy, less-dense foods
- Reduce BMI/obesity
- Reduce chronic disease incidence

Level of Evidence Available to Evaluate Effectiveness of Policy (1 = strong evidence to 3 = insufficient evidence*):^4^

3= Insufficient Evidence

Achievable Results:

The following summary of achievable results is based on a published review of the scientific evidence.

- Taxes and subsidies on food have the potential to influence consumption considerably and improve health, particularly when they are large.^5^
- Imposing substantial taxes on fattening foods may improve health outcomes, such as body weight and chronic disease risk, and may be particularly effective among overweight or low-income children.^1,5^
- More, scientifically rigorous, research is needed.

*Note: 1 = meets criteria for policy effectiveness (consistent, positive outcomes from at least two high-quality experimental or quasi-experimental trials using a comparison group or interrupted time series design)^4^; 2 = consistent evidence available linking policy with positive outcomes from high-quality observational studies only; 3 = insufficient evidence available for policy or policy components.

† Be sure to check with your state, county, and municipal governments regarding potential existing laws that may impede any new policy development.

References

^1^ Powell, LM & Chaloupka, FJ (2009). Food prices and obesity: Evidence and policy implications for taxes and subsidies. Milbank Quarterly, 87(1), 229-257.

^2^ Finkelstein, EA, Fiebelkorn, IC, & Wang, G (2003). National medical spending attributable to overweight and obesity: How much, and who’s paying? Health Affairs, W3-219-W3-226.

^3^ Drewnowski, A & Darmon, N (2005). Food choices and diet costs: An economic analysis. Journal of Nutrition, 135, 900-904.

^4^ Flay, BR, Biglan, A, Boruch, RF, Ganzalez Castro, F, Gottfredson, D, Kellam, S, Moscicki, EK, Schinke, S, Valentine, JC, & Ji, P (2005). Standards of evidence: Criteria for efficacy, effectiveness and dissemination. Prevention Science, 6(3), 151-175.

^5^ Thow, AM, Jan, S, Leeder, S, & Swinburn, B (2010). The effect of fiscal policy on diet, obesity and chronic disease: a systematic review. Bulletin of the World Health Organization, 88, 609-614.

Limiting Alcohol Advertising

Domain: Physical Environment

Reasons for Policy:

- In 2009, 67% of high school students who drank reported binge drinking in the last 30 days. ^1^
- Heavy alcohol consumption in adolescence is linked to heavier consumption, alcohol dependence, and alcohol-related problems in early adulthood.^2^

Community Group:

- Local government
- Local consumer protection agencies
- Local district attorney

Policy Components:

- Restricting, or completely banning, alcohol advertising or marketing that may be seen by large populations of underage youth

Desired Outcomes:

- Delay onset of drinking
- Reduced alcohol use among youth
- Fewer alcohol-related problems (e.g. liver cirrhosis, traffic crashes and fatalities)

Level of Evidence Available to Evaluate Effectiveness of Policy (1 = strong evidence to 3 = insufficient evidence*):^2^

1 = strong evidence for effects of exposure to alcohol advertising on onset and frequency of drinking

3 = insufficient evidence for effects of alcohol advertising bans on drinking behavior and related problems

Achievable Results:

The following summary of achievable results is based on a published review of the scientific evidence.

- Exposure to alcohol advertising is significantly associated with earlier initiation of alcohol use and increased levels of alcohol consumption among drinking youth.^3, 4^
- A number of high-quality, longitudinal studies have shown a dose-response relationship between exposure to alcohol advertising and both alcohol use initiation and consumption.^3, 4^
- The data are insufficient to draw clear conclusions about the net effects of bans and/or restrictions of alcohol advertising on drinking behavior and related problems among youth. More research is needed.^5^

Community Examples:

- San Diego, California restricts the amount of alcoholic beverage billboards in areas highly concentrated by children.
  - <http://www.sandiego.gov/nccd/zoning/billboards.shtml>
- San Francisco, California restricts alcoholic beverage advertising on property owned by the City of San Francisco
  - <http://www.sfexaminer.com/local/bart-reverses-policy-rejects-alcohol-ads>

Links to Policy Examples†:

- San Diego, California Municipal Code §58.0501-58.0504
  - http://docs.sandiego.gov/municode/MuniCodeChapter05/Ch05Art08Division05.pdf
- San Francisco, California Municipal Code §4.20
  - See pdf

*Note: 1 = meets criteria for policy effectiveness (consistent, positive outcomes from at least two high-quality experimental or quasi-experimental trials using a comparison group or interrupted time series design)^5^; 2 = consistent evidence available linking policy with positive outcomes from high-quality observational studies only; 3 = insufficient evidence available for policy or policy components.

† Be sure to check with your state, county, and municipal governments regarding potential existing laws that may impede any new policy development.

References

^1^Campbell CA, et al. (2009). The effectiveness of limiting alcohol outlet density as a means of reducing excessive alcohol consumption and alcohol-related harms. *American Journal of Preventive Medicine,* 37(6). 556-569.

^2^Flay, BR, Biglan, A, Boruch, RF, Ganzalez Castro, F, Gottfredson, D, Kellam, S, Moscicki, EK, Schinke, S, Valentine, JC, & Ji, P (2005). Standards of evidence: Criteria for efficacy, effectiveness and dissemination. Prevention Science, 6(3), 151-175.

^3^Anderson P, de Bruijn A, Angus K, Gordon R, & Hastings G (2009). Impact of alcohol advertising and media exposure on adolescent alcohol use: A systematic review of longitudinal studies. *Alcohol & Alcoholism,* 44(3), 229-243.

^4^Smith LA, & Foxcroft DR (2009). The effect of alcohol advertising, marketing, and portrayal on drinking behaviour in young people: Systematic review of prospective cohort studies. *BMC Public Health,* 9.

^5^Grube J (2005). Preventing alcohol-related problems: Public policy strategies. *Transportation Research Circular: Implementing Impaired Driving Countermeasures: Putting Research into Action,* E-C072, 97-126.

Limiting Pesticide Exposure in Workers

Domain: Physical Environment

Reasons for Policy:

- In the U.S., over 16,000 pesticide products are marketed and approximately 1 billion pounds of pesticide active ingredient are used annually.^1^
- It is estimated that 10,000-20,000 physician-diagnosed pesticide poisonings occur each year among the approximately 2 million U.S. agricultural workers.^1^
- Among all occupational poisonings, it is estimated that between 20,000 and 40,000 poisonings may occur each year.^2^
- The health effects of pesticide exposure depend on the type of pesticide, some affect the nervous system, some are carcinogens, some may irritate the skin and eyes and others affect the endocrine system.^3^

Community Group:

- Local government
- State health or environmental agency
- Federal government
- Local businesses

Policy Components:

- Pesticide handling safety training
- Applicator training and licensure
- Mixing, loading, or application interventions
- Field re-entry restrictions
- Use of personal protective equipment (PPE) and isolation techniques
- Bio-monitoring programs (Cholinesterase testing)

Desired Outcomes:

- Reduction in pesticide exposure
- Reduction in pesticide poisonings

Level of Evidence Available to Evaluate Effectiveness of Policy (1 = strong evidence to 3 = insufficient evidence*):^4^

2 = consistent evidence available linking policy with positive outcomes from high-quality observational

studies only

Achievable Results:

The following summary of achievable results is based on a published review of the scientific evidence.

On average, use of appropriate PPE and different mixing and application procedures, as opposed to open

pouring and dumping can achieve:

- Reductions in pesticide exposure under controlled conditions.^2^

Note: No studies examined the effects of prevention programs on pesticide poisonings. Many of the studies had a small number of subjects. Further research with larger experimental or quasi-experimental studies is needed.

Community Example

- California Environmental Protection Agency, Office of Environmental Health Hazard Assessment overseas a cholinesterase monitoring program.
  - <http://www.oehha.ca.gov/pesticides/programs/Helpdocs1.html> (see pdf)
- Colorado State University has a Colorado Environmental Pesticide Education Program and the Colorado Department of Agriculture administers the Pesticide Applicator Program
  - <http://www.cepep.colostate.edu/>
  - <http://www.colorado.gov/cs/Satellite/Agriculture-Main/CDAG/1167928159784>

Links to Policy Examples:

- California Food and Agricultural Code, D. 7, Ch.2, Art. 10.5, Pesticides and Worker Safety
  - See pdf
- California Code of Regulations, 3 CCR § 6728 Medical Supervision
  - See pdf
- Colorado Revised Statutes, Title 35, Article 10. Pesticide Applicator’s Act
  - C.R.S. § 35-10-118 Powers and duties of the commissioner (see pdf)

Note: Most states regulate pesticide use through the state’s agricultural agency and follow the Environmental Protection Agency’s Worker Protection Standard for Agricultural Pesticides. See <http://www.epa.gov/oecaagct/twor.html>

- 40 C.F.R Part 170 Worker Protection Standard

*Note: 1 = meets criteria for policy effectiveness (consistent, positive outcomes from at least two high-quality experimental or quasi-experimental trials using a comparison group or interrupted time series design)^4^; 2 = consistent evidence available linking policy with positive outcomes from high-quality observational studies only; 3 = insufficient evidence available for policy or policy components.

† Be sure to check with your state, county, and municipal governments regarding potential existing laws that may impede any new policy development.

‡Local governments and organizations may check existing state and federal statutes and administrative codes for the authority to implement local policies.

References

1National Institute for Occupational Safety and Health, Centers for Disease Control and Prevention. NIOSH Safety and Health Topic: Pesticide Illness and Injury Surveillance. U.S. Department of Health and Human Services, Retrieved February 3, 2011, from <http://www.cdc.gov/niosh/topics/pesticides/>.

2 Keifer, MC (2000). Effectiveness of Interventions in Reducing Pesticide Overexposure and Poisonings. American Journal of Preventative Medicine, 18(4S), 80-89.

3 U.S. Environmental Protection Agency. Pesticides: Health and Safety, Human Health Issues. Retrieved February 3, 2011, from <http://www.epa.gov/pesticides/health/human.htm>.

4 Flay, BR, Biglan, A, Boruch, RF, Ganzalez Castro, F, Gottfredson, D, Kellam, S, Moscicki, EK, Schinke, S, Valentine, JC, & Ji, P (2005). Standards of evidence: Criteria for efficacy, effectiveness and dissemination. Prevention Science, 6(3), 151-175.

Social Host Liability and Keg Registration

Domain: Physical Environment

Reasons for Policy:

- In 2009, 67% of high school students who drank reported binge drinking in the last 30 days. ^1^
- Heavy alcohol consumption in adolescence is linked to heavier consumption, alcohol dependence, and alcohol-related problems in early adulthood.^2^
- The costs of alcohol use by underage drinkers was estimated to be $52 billion annually in 1999.^3^

Community Group:

- Local government
- Local law enforcement
- Local businesses that sell alcohol

Policy Components:

- Require beer keg purchasers to complete forms that link their name to the keg’s number (keg registration)
- Enforcement of social host liability laws in which adults that give alcohol to minors or intoxicated adults can be sued for damages or injuries caused by the minor or intoxicated adult
- Campaigns to ensure social hosts are aware of social host liability laws

Desired Outcomes:

- Reduced alcohol use among underage youth
- Reduce blood alcohol concentrations (BACs) among adults
- Reduce alcohol-related traffic crashes and fatalities
- Reduce alcohol-related unintentional injuries

Level of Evidence Available to Evaluate Effectiveness of Policy (1 = strong evidence to 3 = insufficient evidence*):^4^

3 = Insufficient Evidence

Achievable Results:

The following summary of achievable results is based on a published review of the scientific evidence.

- The evidence of effects of keg registration and social host liability laws is mixed. However, favorable trends in impact on traffic crash fatalities, heavy alcohol consumption and drink-driving have been observed. More research is needed.^3^

*Note: 1 = meets criteria for policy effectiveness (consistent, positive outcomes from at least two high-quality experimental or quasi-experimental trials using a comparison group or interrupted time series design)^4^; 2 = consistent evidence available linking policy with positive outcomes from high-quality observational studies only; 3 = insufficient evidence available for policy or policy components.

References

^1^Campbell CA, et al. (2009). The effectiveness of limiting alcohol outlet density as a means of reducing excessive alcohol consumption and alcohol-related harms. *American Journal of Preventive Medicine,* 37(6). 556-569.

^2^Anderson P, de Bruijn A, Angus K, Gordon R, & Hastings G (2009). Impact of alcohol advertising and media exposure on adolescent alcohol use: A systematic review of longitudinal studies. *Alcohol & Alcoholism,* 44(3), 229-243.

^3^Grube J (2005). Preventing alcohol-related problems: Public policy strategies. *Transportation Research E-Circular: Implementing Impaired Driving Countermeasures: Putting Research into Action,* E-C072, 93-118.

^4^Flay, BR, Biglan, A, Boruch, RF, Ganzalez Castro, F, Gottfredson, D, Kellam, S, Moscicki, EK, Schinke, S, Valentine, JC, & Ji, P (2005). Standards of evidence: Criteria for efficacy, effectiveness and dissemination. Prevention Science, 6(3), 151-175.

Supermarket Access

Domain: Physical Environment

Reasons for Policy:

- Prevalence of overweight among children and adolescents in the United States has almost tripled since 1980. ^1^
- Significant disparities exist among socioeconomically disadvantaged and racial/ethnic minority populations.^2^
- Neighborhood built environments (including the retail food environment) are believed to influence eating behaviors and potentially contribute to the observed health disparities.^3^

Community Group:

- Local Government
- Local Businesses
- Local Public Health Department

Policy Components:

- Increase access to supermarkets in low-income neighborhoods

Desired Outcomes:

- Decreased obesity rates in socioeconomically disadvantaged and racial/ethnic minority populations
- Increased accessibility to healthy foods

Level of Evidence Available to Evaluate Effectiveness of Policy (1 = strong evidence to 3 = insufficient evidence*):^4^

3= Insufficient Evidence

Achievable Results:

The following summary of achievable results is based on a published review of the scientific evidence.

- Neighborhood residents who have better access to supermarkets and limited access to convenience stores may have healthier diets and lower levels of obesity.^5,6,7^
- More, scientifically rigorous, research is needed.

*Note: 1 = meets criteria for policy effectiveness (consistent, positive outcomes from at least two high-quality experimental or quasi-experimental trials using a comparison group or interrupted time series design)^4^; 2 = consistent evidence available linking policy with positive outcomes from high-quality observational studies only; 3 = insufficient evidence available for policy or policy components.

† Be sure to check with your state, county, and municipal governments regarding potential existing laws that may impede any new policy development.

References

^1^ Flegal K. Epidemiological aspects of overweight and obesity in the United States. Physiology & Behavior, 86, 599-602.

^2^ Crawford, PB, Wang, M, Krathwohl, S, & Ritchie, LD (2006). Disparities in obesity: Prevalence, causes, and solutions. Journal of Hunger and Environmental Nutrition, 1, 27-48.

^3^ Glanz, K, Sallis, J, Saelens, BE, & Frank, LD (2005). Healthy nutrition environments: Concepts and measures. American Journal of Health Promotion, 19, 330-333.

^4^ Flay, BR, Biglan, A, Boruch, RF, Ganzalez Castro, F, Gottfredson, D, Kellam, S, Moscicki, EK, Schinke, S, Valentine, JC, & Ji, P (2005). Standards of evidence: Criteria for efficacy, effectiveness and dissemination. Prevention Science, 6(3), 151-175.

^5^ Ford, PB & Dzewaltowski, DA (2008). Disparities in obesity prevalence due to variation in the retail food environment: Three testable hypotheses. Nutrition Reviews, 66(4), 216-228.

^6^ Larson, NI, Story, MT, & Nelson, MC (2009). Neighborhood environments: Disparities in access to healthy foods in the U.S. American Journal of Preventive Medicine, 36(1), 74-81.

^7^ Giskes, K, van Lenthe, F, Avendano-Pabon, M, Brug, J (2010). A systematic review of environmental factors and obesogenic dietary intakes among adults: Are we getting closer to understanding obesogenic environments? Obesity Reviews, no.doi: 10.1111/j.1467-789X.2010.00769.x.

Tobacco Sales Laws Enforcement

Domain: Physical Environment

Reasons for Policy:

- Controlling access to tobacco is an established strategy for reducing consumption.^1^
- Poor compliance with tobacco sales laws is well documented.^1^
- Despite declining trends, 78% of 10^th^ graders say they find it ‘fairly easy’ or ‘very easy’ to get cigarettes.^1^
- In 2006, of those underage students who tried to buy cigarettes in a store in the previous 30 days, approximately half had been asked for proof of age.^1^

Community Group:

- Local government
- State government
- Local law enforcement
- Local/state health officials

Policy Components:

- Active enforcement of sales laws directed at retailers
- Periodic unannounced compliance checks
- Citations for retailers that sell tobacco to minors

Desired Outcomes:

- Reduce illegal sales of tobacco to minors
- Reduce access to tobacco by minors
- Reduce tobacco consumption

Level of Evidence Available to Evaluate Effectiveness of Policy (1 = strong evidence to 3 = insufficient evidence*):^3^

3= Insufficient evidence

Achievable Results:

The following summary of achievable results is based on a published review of the scientific evidence.

- There is evidence linking active enforcement of laws prohibiting sales of tobacco to minors to reductions in the sale of cigarettes to youth^2^
- There is evidence linking enforcement or warnings to retailer behavior, but compliance requires regular enforcement and there is reduced effectiveness with less occurrences of enforcement.^1^
- Additional research and systematic reviews or meta-analyses are needed.

*Note: 1 = meets criteria for policy effectiveness (consistent, positive outcomes from at least two high-quality experimental or quasi-experimental trials using a comparison group or interrupted time series design)^3^; 2 = consistent evidence available linking policy with positive outcomes from high-quality observational studies only; 3 = insufficient evidence available for policy or policy components.

† Be sure to check with your state, county, and municipal governments regarding potential existing laws that may impede any new policy development.

‡Local governments and organizations may check existing state and federal statutes and administrative codes for the authority to implement local policies.

References

1 Stead, LF, Lancaster, T (2005). Interventions for preventing tobacco sales to minors. Cochrane Database of Systematic Reviews, 1, CD001497.

2 Forster, JL, Widome, R, & Bernat, DH (2007). Policy interventions and surveillance as strategies to prevent tobacco use in adolescents and young adults. American Journal of Preventive Medicine, 33(6S), S35-S339.

3 Flay, BR, Biglan, A, Boruch, RF, Ganzalez Castro, F, Gottfredson, D, Kellam, S, Moscicki, EK, Schinke, S, Valentine, JC, & Ji, P (2005). Standards of evidence: Criteria for efficacy, effectiveness and dissemination. Prevention Science, 6(3), 151-175.

Mental Health Services for Parents

Domain: Family Influences

Reasons for Policy:

- Up to 23% of all families have at least one parent with mental illness.^1^
- Mental illness can greatly impact parenting skills (e.g. less sensitive, poorer communication, and less affectionate).^1^
- Parental mental illness is associated with negative child outcomes such as, mental illness in the child, higher infant mortality rate, higher likelihood to be placed in foster care, and insecure attachment.^1^

Community Group:

- Local government
- Local public health department
- Local non-profit organizations

Policy Components:

- Proper identification of parenting status of mental health services users
- Parenting skills training for parents with mental illnesses
- Family-focused practices and interventions
- Child care provided while the parent is using services
- Psychoeducation for parents and children about mental illnesses
- Use of interagency collaboration

Desired Outcomes:

- Positive parent-child relationships
- Increased use of positive parenting skills
- Increased mental health in parents
- Increased mental health and social adjustment in children
- Secure/positive attachment styles in children

Level of Evidence Available to Evaluate Effectiveness of Policy (1 = strong evidence to 3 = insufficient evidence*):^3^

3 = insufficient evidence available

Achievable Results:

The following summary of achievable results is based on a published review of the scientific evidence.

- The evidence of effectiveness for these programs is insufficient. More research is needed.^1,2^

*Note: 1 = meets criteria for policy effectiveness (consistent, positive outcomes from at least two high-quality experimental or quasi-experimental trials using a comparison group or interrupted time series design)^3^; 2 = consistent evidence available linking policy with positive outcomes from high-quality observational studies only; 3 = insufficient evidence available for policy or policy components.

References

^1^Maybery, D, & Reupert, A (2009). Parental mental illness: A review of barriers and issues for working with families and children. *Journal of Psychiatric and Mental Health Nursing,* 16, 784-791.

^2^Craig, EA (2004). Parenting programs for women with mental illness who have young children: A review. *Australian and New Zealand Journal of Psychiatry,* 38, 923-928.

^3^Flay, BR, Biglan, A, Boruch, RF, Ganzalez Castro, F, Gottfredson, D, Kellam, S, Moscicki, EK, Schinke, S, Valentine, JC, & Ji, P (2005). Standards of evidence: Criteria for efficacy, effectiveness and dissemination. Prevention Science, 6(3), 151-175.

Adequate Yearly Progress

Domain: School Influences

Reasons for Policy:

- The achievement gap on standardized tests is viewed as the most significant educational challenge facing American society in the 21^st^ century.^1^
- The 2001 No Child Left Behind Act (NCLB) aims to close the achievement gap between high- and low-performing children, especially minority and low-income students.^2^
- Adequate yearly progress (i.e., standards-based accountability) requirements are the central mechanism for improving school performance and the academic achievement of different subgroups of students.^1^

Community Group:

- This policy is implemented at a national and state level.

Policy Components:

- Standards apply only to schools that receive federal funds
- Schools must meet an absolute level of performance in reading and mathematics with a defined minimum number of students who must meet the proficiency level
- Each state establishes its own performance goals
- Schools that fail to make AYP for two or more consecutive years are subject to a series of sanctions

Desired Outcomes:

- Improve teaching quality within schools
- Improve reading and mathematics proficiency in low-performing subgroups
- Close the achievement gap between high- and low-performing children

Level of Evidence Available to Evaluate Effectiveness of Policy (1 = strong evidence to 3 = insufficient evidence*):^3^

3 = Insufficient Evidence

Achievable Results:

The following summary of achievable results is based on published reviews of the scientific evidence.

- Qualitative studies suggest that standards-based accountability may be associated with reductions in the curricular content covered in school. Classroom learning is narrowed to tested subjects, and subject area knowledge is fragmented into test-related pieces.^4^ However, more high-quality research is needed to understand the effects.

*Note: 1 = meets criteria for policy effectiveness (consistent, positive outcomes from at least two high-quality experimental or quasi-experimental trials using a comparison group or interrupted time series design)^3^; 2 = consistent evidence available linking policy with positive outcomes from high-quality observational studies only; 3 = insufficient evidence available for policy or policy components.

References

^1^ Kim, J & Sunderman, GL (2005). Measuring academic proficiency under the No Child Left Behind Act: Implications for educational equity. Educational Researcher, 34(8), 3-13.

^2^ No Child Left Behind Act of 2001, Pub. L. No. 107-110, 115 Stat. 1425.

^3^ Flay, BR, Biglan, A, Boruch, RF, Ganzalez Castro, F, Gottfredson, D, Kellam, S, Moscicki, EK, Schinke, S, Valentine, JC, & Ji, P (2005). Standards of evidence: Criteria for efficacy, effectiveness and dissemination. Prevention Science, 6(3), 151-175.

^4^ Au, W (2007). High-stakes testing and curricular control: A qualitative metasynthesis. Educational Researcher, 36, 258-268.

Charter Schools

Domain: School Influences

Reasons for Policy:

- Charter schools expand the variety of schools available and provide unique educational options to students in the community.^1^
- Charter schools have increased autonomy and flexibility, allowing for innovation and adaptive education while promoting educational reform.^2^
- Nationwide, charter schools serve a greater number of minority and low-income students.^3^

Community Group:

- Local School Board
- Public University
- Local Government

Policy Components:

- Charter schools are more accountable than traditional schools and must report standardized academic test scores in order to renew charter contracts.
- Charter schools close if they fail to uphold contract with the local school board^4^

Desired Outcomes:

- Improved student achievement^5^
- Improved teacher, parent, and student satisfaction^5^
- Increased parental involvement
- Improved system of public education^5^

Level of Evidence Available to Evaluate Effectiveness of Policy (1 = strong evidence to 3 = insufficient evidence*):^6^

3= Insufficient Evidence

Achievable Results:

The following summary of achievable results is based on a published review of the scientific evidence.

- The evidence on effects of charter schools on student achievement is mixed, some high-quality studies showing positive effects and others showing negative. More research is needed. ^7^
- Positive effects have been observed for parental involvement and satisfaction among parents, students and teachers. ^5^

*Note: 1 = meets criteria for policy effectiveness (consistent, positive outcomes from at least two high-quality experimental or quasi-experimental trials using a comparison group or interrupted time series design)^5^; 2 = consistent evidence available linking policy with positive outcomes from high-quality observational studies only; 3 = insufficient evidence available for policy or policy components.

References

^1^ Nathan, J. (1996). Charter schools: Creating hope and opportunity for American education. San Francisco: Jossey-Bass.

^2^ Arsen, D, Plank, D, & Sykes, G (1999). School choice policies in Michigan: The rules matter. East Lansing: Michigan State University.

^3^ RPP International (2000). The state of charter schools: 2000. Washington, DC: U.S. Department of Education, Office of Educational Research and Improvement.

^4^ Renzulli, LA & Roscigno, VJ (2005). Charter school policy, implementation, and diffusion across the United States. Sociology of Education, 78(4), 344-365.

^5^ Bulkley, K & Fisler, J (2003). A decade of charter schools: From theory to practice. Educational Policy, 17(3), 317-342.

^6^ Flay, BR, Biglan, A, Boruch, RF, Ganzalez Castro, F, Gottfredson, D, Kellam, S, Moscicki, EK, Schinke, S, Valentine, JC, & Ji, P (2005). Standards of evidence: Criteria for efficacy, effectiveness and dissemination. Prevention Science, 6(3), 151-175.

^7^ Miron, G & Nelson, C (2001). Student academic achievement in charter schools: what we know and why we know so little. Occasional Paper No. 41. National Center for the Study of Privatization in Education, Teacher’s College, Columbia University.

Early College Programs

Domain: School Influences

Reasons for Policy:

- The strongest predictor of bachelor’s degree completion is the intensity and quality of students’ high school curriculum^1^
- Exposing traditionally non-college-bound students to college while still in high school can demystify college and build motivation^2^
- Earning college credit in high school lowers the long-term cost of a college degree^3^

Community Group:

- Local Public School District
- Local Community Colleges
- Local Colleges & Universities

Policy Components:

- Offer opportunities to earn college credit for coursework completed during high school, such as enrolling in classes at the college directly or taking college-level classes in high school and scoring high on standardized exams.
- Help students at risk for dropping out meet graduation requirements and transition to postsecondary education through Middle College High School programs.

Desired Outcomes:

- Increase college enrollment and success in otherwise non-college bound students
- Give advanced students an opportunity to enter college more prepared

Level of Evidence Available to Evaluate Effectiveness of Policy (1 = strong evidence to 3 = insufficient evidence*):^4^

3= Insufficient Evidence

Achievable Results:

The following summary of achievable results is based on a published review of the scientific evidence.

- Little is known of the overall characteristics and effects of these programs. More rigorous research is needed.^2^

*Note: 1 = meets criteria for policy effectiveness (consistent, positive outcomes from at least two high-quality experimental or quasi-experimental trials using a comparison group or interrupted time series design)^4^; 2 = consistent evidence available linking policy with positive outcomes from high-quality observational studies only; 3 = insufficient evidence available for policy or policy components.

References

^1^ Adelman, C (1999). Answers in the tool box: Academic intensity, attendance patterns, and Bachelor’s degree attainment. Washington, DC: U.S. Department of Education, Office of Educational Research and Improvement.

^2^ Bailey, T & Karp, MM (2003). Promoting college access and success: a review of credit-based transition programs. New York: Teachers College, Columbia University, Community College Research Center.

^3^ Orr, MT (1998). Integrating secondary schools and community colleges through school-to-work transition and education reform. Journal of Vocational Education Research, 23(2), 93-113.

^4^ Flay, BR, Biglan, A, Boruch, RF, Ganzalez Castro, F, Gottfredson, D, Kellam, S, Moscicki, EK, Schinke, S, Valentine, JC, & Ji, P (2005). Standards of evidence: Criteria for efficacy, effectiveness and dissemination. Prevention Science, 6(3), 151-175.

K-8 School Grade Configuration

Domain: School Influences

Reasons for Policy:

- School grade configuration is an important issue in public education.^1^
- Grade configuration influences academic achievement, school attendance, and social development.^1^
- In a K-8 grade configuration, the absence of school to school transition and continuity of experience may lead to higher academic achievement.^1^

Community Group:

- Local school district
- Local school board
- State department of education

Policy Components:

- K-8 grade configuration (elementary-wide) as opposed to a 6-8 grade configuration (middle school).

Desired Outcomes:

- Improved academic achievement
- Improved social development and functioning
- Improved school attendance
- Decreased drop-out rates

Level of Evidence Available to Evaluate Effectiveness of Policy (1 = strong evidence to 3 = insufficient evidence*):^2^

3 = Insufficient Evidence Available

Achievable Results:

The following summary of achievable results is based on a published review of the scientific evidence.

- Additional research is needed to conclude that K-8 grade configuration has positive effects on the desired outcomes. Available evidence generally suggests that achievement in grades 6-8 is higher in schools having a K-8 grade configuration than a middle school configuration. However, more research is needed.^1^

*Note: 1 = meets criteria for policy effectiveness (consistent, positive outcomes from at least two high-quality experimental or quasi-experimental trials using a comparison group or interrupted time series design)^2^; 2 = consistent evidence available linking policy with positive outcomes from high-quality observational studies only; 3 = insufficient evidence available for policy or policy components.

† Be sure to check with your state, county, and municipal governments regarding potential existing laws that may impede any new policy development.

References

1 Coladarci T, Hancock J (2002). Grade-span configuration. *Journal of Research in Rural Education*, 17(3), 189-192.

2 Flay, BR, Biglan, A, Boruch, RF, Ganzalez Castro, F, Gottfredson, D, Kellam, S, Moscicki, EK, Schinke, S, Valentine, JC, & Ji, P (2005). Standards of evidence: Criteria for efficacy, effectiveness and dissemination. Prevention Science, 6(3), 151-175.

School Music Programs

Domain: School Influences

Reasons for Policy:

- Music is an important dimension of academic development.^1^
- Youth music participation is associated with higher matriculation rates.^2^
- Ninety-five percent of Americans believe that music is a key component in children’s education.³
- Over seventy-five percent of Americans feel schools should mandate music education.³

Community Group:

- School district/ local school board
- Local government
- State government
- Parent-Teacher Association (PTA)

Policy Components:

- Weekly, in-school music class participation
- Elementary school music program
- High school music program

Desired Outcomes:

- Improved school attendance
- Increased achievement scores
- Positive classroom behavior
- Decreased rates of current and lifetime alcohol, tobacco or drug abuse

Level of Evidence Available to Evaluate Effectiveness of Policy (1 = strong evidence to 3 = insufficient evidence*)^4^:

3= insufficient evidence

Achievable Results:

The following summary of achievable results is based on a published review of the scientific evidence.

- There is insufficient evidence to support conclusions related to the nonmusical outcomes (academic achievement, social and emotional growth) of music education.^5^
- Although research has produced positive results, the conclusions are generally unconvincing due to inadequacies in experimental designs.^5^
- Further research and better designed studies are needed to assess the effects of school based music education on nonmusical outcomes.^5^

*Note: 1 = meets criteria for policy effectiveness (consistent, positive outcomes from at least two high-quality experimental or quasi-experimental trials using a comparison group or interrupted time series design)^5^; 2 = consistent evidence available linking policy with positive outcomes from high-quality observational studies only; 3 = insufficient evidence available for policy or policy components.

† Be sure to check with your state, county, and municipal governments regarding potential existing laws that may impede any new policy development.

References

1 Southgate DE, Roscigno VJ (2009). The impact of music on childhood and adolescent achievement. *Social Science Quarterly*, 90(1), 4-21.

2 Aschaffenburg K, Maas I (1997). Cultural and Educational Careers: The Dynamics of Social Reproduction. *American Sociological Review,* 62, 573-87.

3 American Music Conference. (2003). *Press Releases.* Carlsbad, CA: American Music Conference.

4 Flay, BR, Biglan, A, Boruch, RF, Ganzalez Castro, F, Gottfredson, D, Kellam, S, Moscicki, EK, Schinke, S, Valentine, JC, & Ji, P (2005). Standards of evidence: Criteria for efficacy, effectiveness and dissemination. Prevention Science, 6(3), 151-175.

5 Wolff KL, (2004). The Nonmusical Outcomes of Music Education: A Review of the Literature. *Bulletin of the Council for the Research in Music Education.* 159, 74-91.

Vaccination for Daycare

Domain: School Influences

Reasons for Policy:

- In the United States, more than 400,000 cases of illness and 30,000 deaths caused by vaccine-preventable diseases occur each year.^1^
- The effectiveness of universally recommended vaccinations in preventing disease for children is well-established.^2^
- Enactment and enforcement of state immunization laws during the 1970s–1980s led to more than 95% of school–aged children now being appropriately vaccinated with recommended doses of vaccine. ^3^

Community Group:

- State and Local Government
- Public Health Department
- Day Care Programs

Policy Components:

- Requiring vaccinations or documentation of immunity as a condition of day care attendance
- Availability of free or reduced-price vaccinations to low income children through the health department

Desired Outcomes:

- Increase in the vaccination coverage levels in young children
- Decrease in the prevalence of vaccine-preventable illnesses and deaths in young children.

Level of Evidence Available to Evaluate Effectiveness of Policy (1 = strong evidence to 3 = insufficient evidence*):^4^

3 = Insufficient Evidence

Achievable Results:

There is insufficient evidence available on effects of vaccination requirements for day care attendance. However, among school- and college-aged youth, vaccination requirements may increase vaccine coverage levels by 15%.^5^ More research is needed.

*Note: 1 = meets criteria for policy effectiveness (consistent, positive outcomes from at least two high-quality experimental or quasi-experimental trials using a comparison group or interrupted time series design)^4^; 2 = consistent evidence available linking policy with positive outcomes from high-quality observational studies only; 3 = insufficient evidence available for policy or policy components.

† Be sure to check with your state, county, and municipal governments regarding potential existing laws that may impede any new policy development.

References

^1^ Fedson, DS (1994). Adult immunization: Summary of the National Vaccine Advisory Committee. JAMA, 91:320S-324S.

^2^ Centers for Disease Control and Prevention: Recommended childhood immunization schedule-United States. MMWR 1998;47, 8-12.

^3^ Briss, PA, Rodewald, LE, Hinman, AR, Shefer, AM, Strikas, RA, Bernier, RR, Carande-Kulis, VG, Yusuf, HR, Ndiaye, SM, Williams, SM, & The Task Force on Community Preventive Services (2000). Reviews of evidence regarding interventions to improve vaccination coverage in children, adolescents, and adults. American Journal of Preventive Medicine, 18(1S):97-140.

^4^ Flay, BR, Biglan, A, Boruch, RF, Ganzalez Castro, F, Gottfredson, D, Kellam, S, Moscicki, EK, Schinke, S, Valentine, JC, & Ji, P (2005). Standards of evidence: Criteria for efficacy, effectiveness and dissemination. Prevention Science, 6(3), 151-175.

^5^ Shefer, A, Briss, P, Rodewald, L, Bernier, R, Strikas, R, Yusuf, H, Ndiaye, S, Williams, S, Pappaioanou, M, & Hinman, AR (1999). Improving immunization coverage rates: A review of the literature. Epidemiological Reviews, 21(1), 96-142.
